# Supplementary material for: ‘If I am on ART, my new-born baby should be put on treatment immediately’: Exploring the acceptability, and appropriateness of Cepheid Xpert HIV-1 Qual assay for early infant diagnosis of HIV in Malawi
Source: PLOS Glob Public Health. 2023 Mar 10;3(3):e0001135. doi: 10.1371/journal.pgph.0001135 (PMC10021387; doi:10.1371/journal.pgph.0001135)
Supplement: S1 Text — (DOCX) [file pgph.0001135.s002.docx]

**Interview guide for caregivers**

*A Questionnaire to validate new HIV test called Cepheid Xpert HIV -1 Quay assay (Cepheid) in your hospital*

1. How would you as a parent/guardian feel if your child was to undergo HIV testing with **Cepheid Xpert HIV -1 Quay assay using whole blood (Cepheid)?**

**Malingana ndi mmene tafotokozera za kayezedwe ka** **Cepheid Xpert HIV -1 Quay assay using whole blood (Cepheid), mwana ayenera kutengedwa magazi pachara kapena pa nsempha, inu monga kholo mungamve bwanji kuti mwana wanu ayezedwe magazi kuzera njira zimezi?**

2. What are your thoughts about this new strategy **Cepheid Xpert HIV -1 Quay assay using whole blood (Cepheid)** for testing HIV in children and giving results promptly?

**Kwainu monga kholo la mwana wa chichepere, maganizo anu ndi otani pokhuzana ndi mayezedwe a magazi kuti tidziwe kuti mwana ali ndi HIV kapena ayi malingana ndi mmene tafotokozera za kayezedwe ka Cepheid Xpert HIV -1 Quay assay using whole blood (Cepheid), malingana ndi nthawi yimene zosatira zimatuluka ?**

3. How should this **Cepheid Xpert HIV -1 Quay assay using whole blood (Cepheid)** approach be implemented in a hospital? (Probe who should be targeted, why should they be targeted and why?)

**Kodi njira zimenezi tingazikhazikise bwanji mu zipatala? (tatiwuzani, tiyambe ndi gulu liti la anthu ndipo nchifukwa chani mukuganiza kuti tiyambe ndi gulu limeneli chifukwa chain?**

4. How should issues of privacy of both children and their guardians be maintained?

**Kodi tingapange bwanji kuti kuyezesa magazi kwa ana ndi makolo awo kapena anthu owayang’ira zikhale za chinsinsi?**

5a.What should be the role of parents/guardians in the implementations of these approaches?

**Kodi makolo angatengepo gawo lanji kuti njira zoyezesera magazi za Cepheid Xpert HIV -1 Quay assay using whole blood (Cepheid), zikhazikisidwe mu chipatala chathu chino cha Mulanje?**

b.What information should be provided to ensure that guardians understand the procedures involved?

**Kodi makolo awuzidwe zotani ndi uphungu wotani kuti amvesese za njira zoyezesera magazi za Cepheid Xpert HIV -1 Quay assay using whole blood (Cepheid) ?**

6. What should be the role of male partners in the implementation of **Cepheid Xpert HIV -1 Quay assay using whole blood (Cepheid)?** (Probe: How should male partners be encouraged to take active role in this approach?)

**Kodi azibambo angatengepo gawo lanji kuti njira zoyezesera magazi za Cepheid Xpert HIV -1 Quay assay using whole blood (Cepheid) zikhazikisidwe mu chipatala chathu chino cha Mulanje? Tingawalimbikise bwanji azibambo kuti azitenga nawo gawo mukuyezedwa magazi mu njira za Cepheid**

7. How would your community feel if **Cepheid Xpert HIV -1 Quay assay using whole blood (Cepheid)** was to be implemented in your nearest health facility? (What could be done to encourage community members to participate in this intervention?)

**Kodi anthu a mmudzi mwanu angamve bwanji njira zoyezesera magazi za Cepheid Xpert HIV -1 Quay assay using whole blood (Cepheid) zitakhazikisidwa pa chipatala chanu chaching’ono mmudzi mwanu? Tingatani kuti anthu a mmudzi muno alimbikisidwe kutenga nawo mbali mu njira zoyezetsera magazi za Cepheid Xpert HIV -1 Quay assay using whole blood (Cepheid)?**

8. What are some concerns that you and some members in the community might have related to receiving HIV test results of a child?

**Kodi inu ndi anthu ena mma midzi mu mumakhala ndi nkhawa zanji zokhuzana ndi kulandila zosatira za magazi mwana akayezedwa kuti tiziwe kuti mwana ali ndi HIV kapena ayi?**

Ena amadandawula kuti ukapezeka ndi HIV uzimwa ma ARV moyo wako wonse.

9. Do you have suggestions or ideas for addressing possible community concerns about **Cepheid Xpert HIV -1 Quay assay using whole blood (Cepheid) as an**  HIV testing strategy?

**Kodi mungakhale ndi njira kapena maganizo a momwe tingathandizire kuchepesa nkhawa zokhuzana ndikulandila zotsatira za magazi mwana wayezedwa kuti tidziwe kuti mwana ali ndi HIV kapena ayi?**

B. Perceptions about time to receive test results

10. From the time that your child is tested, how long would you be patient enough to know results from the blood tests? (Same day, after three, after three months?)

**Kuchokera pa nthawi yomwe mwana wanu wayezedwa magazi kuti tidziwe kuti mwana ali ndi HIV kapena ayi, mungapilile nthawi yayitali bwanji kuti mudziwe zosatira**

Tsiku Lomwelo □

Patatha masiku □

Miyezi iwiri kapena itatu □

Fotokozani zifukwa zomwe mwasankhira Yankho limeneli

the tests are HIV positive? (same day, after three, after three months?)Explain why you would prefer your chosen answer.

**Mwana wanu atayezedwa magazi, mungafune kudikila nthawi yayitali bwanji kuti mudziwe kuti mwana ali ndi HIV yomwe yimayambitsa matenda a AIDS?**

Tsiku Lomwelo □

Patatha masiku □

Miyezi iwiri kapena itatu □

Fotokozani zifukwa zomwe mwasankhira Yankho limeneli

12. If your child test for HIV, how long would you want to wait before you are told that results from the test are HIV negative? (Same day, after three, after three months?) Explain why you would prefer your chosen answer.

**Mwana wanu atayezedwa magazi mungafune kudikila nthawi yayitali bwanji kuti muziwe kuti mwana alibe HIV yomwe imayambitsa matenda a AIDS**

Tsiku Lomwelo □√

Patatha masiku □

Miyezi iwiri kapena itatu □

Fotokozani zifukwa zomwe mwasankhira Yankho limeneli

Chifukwa kamatha kubisala ka chilombo nde Patatha Miyezi itatu

C.Acceptability and decision making

13. What information would you want to be given to make an informed decision to accept that your child should get an HIV test or not? Explain

**kodi mungafune muwuzidwe zotani ndi uphungu otani kuti inu mupange chisankho choti mwana wanu ayezedwe magazi kuti mudziwe kuti mwana ali ndi HIV yomwe imayambitsa matenda a AIDS kapena ayi? Fotokozani bwino lomwe.**

14. How would you want to be approached and given information about these two HIV testing strategies? Explain

**Mungafune kuti tikufikileni mu njira yotani kuti tikuwuzeni zimezi ndikukupasani uphungu umenewu wa njira zoyezesera magazi za Cepheid Xpert HIV -1 Quay assay using whole blood (Cepheid)?**

D.Potential Social Harms/Concerns etc.

15. Would you encourage other parents/guardians to allow their children to test for HIV using these the **Cepheid Xpert HIV -1 Quay assay using whole blood (Cepheid)** approach? Yes □ No □

What would be your main concerns and worries towards this approach?

**Kodi mungathe kuwalimbikisa makolo anzanu kapena owasamalira ana kuti alore ana Awo ayezedwwe magazi kuti aziwe ngati ali ndi HIV yoyambitsa matenda a AIDS kugwilitsa ntchito Cepheid Xpert HIV -1 Quay assay using whole blood (Cepheid)?**

Alibepo Nkhawa ina iliyonse

16. How would you personally feel if someone from your community learns about HIV test results for your child?

**Kodi mungamve bwanji ngati munthu wina wa mmudzi mwanu ataziwa zotsatira za magazi a mwana wanu atayezedwa kufufuza ngati ali ndi HIV kapena ayi?**

17. Do you have any other thoughts you wish to share on this topic?

**Kodi muli ndi maganizo kapena nkhawa zina zomwe mungafune kutidziwisa pa nkhani imeneyi**

Alibepo Nkhawa ina iliyonse koma maganizo awo ndiwoti njilazi zipitilile

*The Research Team*
