# Supplementary material for: ‘If I am on ART, my new-born baby should be put on treatment immediately’: Exploring the acceptability, and appropriateness of Cepheid Xpert HIV-1 Qual assay for early infant diagnosis of HIV in Malawi
Source: PLOS Glob Public Health. 2023 Mar 10;3(3):e0001135. doi: 10.1371/journal.pgph.0001135 (PMC10021387; doi:10.1371/journal.pgph.0001135)
Supplement: S2 Text — (DOCX) [file pgph.0001135.s003.docx]

**Interview guide for health workers**

1. As a health professional, how do you feel as you deliver this Cepheid Xpert HIV -1 Quay assay using whole blood (Cepheid) which involves taking blood.
2. As a health professional, how do you feel as you interact with a care giver where you are taking blood from their infant or child.
3. If this way of HIV testing using XpertHIV whole blood is scaled above, do you feel other health workers will be interested in this method?
4. Will it add any extra demand on the health services?
5. Do you feel you need a lot of time when performing this test?
6. Are the procedures involved easy to follow?
7. As you deliver this service, what is the general impression of parents and care givers as their children are having blood taken?
8. EID results using DBS and PCR turn- around time of results is 2-3 months, do you think the ministry of health would be interested in Cepheid whole blood protocol which takes 2 hours?
9. Do you think the government can afford HIV testing with Cepheid ?
10. Can Cepheid whole blood protocol be scaled up?
11. If yes, what would be the barriers?
12. If yes, what would be the selling points?
13. Why do women have a lot more confidence in hospital staff?
14. Why is it that caregivers especially women do not have anything to say when asked questions?
15. Why is that caregivers hardly explain answerers, their answers are very short? Eg Anxiety about the window period?
16. What is your opinion about testing for HIV among mothers whose partners are HIV positive?

The research team
