# Supplementary material for: ‘If I am on ART, my new-born baby should be put on treatment immediately’: Exploring the acceptability, and appropriateness of Cepheid Xpert HIV-1 Qual assay for early infant diagnosis of HIV in Malawi
Source: PLOS Glob Public Health. 2023 Mar 10;3(3):e0001135. doi: 10.1371/journal.pgph.0001135 (PMC10021387; doi:10.1371/journal.pgph.0001135)
Supplement: S1 File — (ZIP) [file pgph.0001135.s004.zip › transcripts/0040.docx]

*A Questionnaire to validate new HIV tests called Cepheid Xpert HIV -1 Quay assay (Cepheid) in your hospital*

DET 0040

*Participant number 0040 analibe maganizo ena aliwonse okhudzana ndi kafukufuku makamaka wanjira zimenezi za Cepheid. (PARTICIPANT ONLY ANSWERED QUESTION 1 THEREAFETER HAD NO THOUGHTS*

1. How would you as a parent/guardian feel if your child was to undergo HIV testing with Cepheid ?

CG-Atha kumva bwino chifukwa aziwa mene nthupi mwa mwana mulili

2. What are your thoughts about this new strategy **Cepheid Xpert HIV -1 Quay assay using whole blood (Cepheid)**  for testing HIV in children and giving results promptly?

CG-Alibe maganizo alionse

3. How should this approach of **Cepheid Xpert HIV -1 Quay assay using whole blood (Cepheid)**

be implemented in a hospital? (Probe who should be targeted, why should they be targeted and why?)

4. How should issues of privacy of both children and their guardians be maintained?

5a.What should be the role of parents/guardians in the implementations of these approaches?

b.What information should be provided to ensure that guardians understand the procedures involved?

6. What should be the role of male partners in the implementation of these approaches? (Probe: How should male partners be encouraged to take active role in this approach of **Cepheid Xpert HIV -1 Quay assay using whole blood (Cepheid)**?

7. How would your community feel if this approach of **Cepheid Xpert HIV -1 Quay assay using whole blood (Cepheid)**  were to be implemented in your nearest health facility? (What could be done to encourage community members to participate in these interventions?)

8. What are some concerns that you and some members in the community might have related to receiving HIV test results of a child?

9. Do you have suggestions or ideas for addressing possible community concerns about these HIV testing strategies?

B. Perceptions about time to receive test results

10. From the time that your child is tested, how long would you be patient enough to know results from the blood tests? (Same day, after three, after three months?)

Tsiku Lomwelo □

Patatha masiku □

Miyezi iwiri kapena itatu □

Fotokozani zifukwa zomwe mwasankhira Yankho limeneli

11. If your child is tested for HIV, how long would you want to wait before you are told that results from the tests are HIV positive? (same day, after three, after three months?)Explain why you would prefer your chosen answer.

Tsiku Lomwelo □

Patatha masiku □

Miyezi iwiri kapena itatu □

Fotokozani zifukwa zomwe mwasankhira Yankho limeneli

Malingana ndikafukufukuyo kuti aziwe bwino ngati ali nako kapena ayi

12. If your child test for HIV, how long would you want to wait before you are told that results from the test are HIV negative? (Same day, after three, after three months?)Explain why you would prefer your chosen answer.

Tsiku Lomwelo □

Patatha masiku □

Miyezi iwiri kapena itatu □

Fotokozani zifukwa zomwe mwasankhira Yankho limeneli

C.Acceptability and decision making

13. What information would you want to be given to make an informed decision to accept that your child should get an HIV test or not? Explain

14. How would you want to be approached and given information about these two HIV testing strategies? Explain

D.Potential Social Harms/Concerns etc.

15. Would you encourage other parents/guardians to allow their children to test for HIV using this approach of **Cepheid Xpert HIV -1 Quay assay using whole blood (Cepheid)**? What would be your main concerns and worries towards **Cepheid Xpert HIV -1 Quay assay using whole blood (Cepheid)**?

Yes □ No □

16. How would you personally feel is someone from your community learns about HIV test results for your child?

17. Do you have any other thoughts you wish to share on this topic?

*The Research Team*
