# Supplementary material for: ‘If I am on ART, my new-born baby should be put on treatment immediately’: Exploring the acceptability, and appropriateness of Cepheid Xpert HIV-1 Qual assay for early infant diagnosis of HIV in Malawi
Source: PLOS Glob Public Health. 2023 Mar 10;3(3):e0001135. doi: 10.1371/journal.pgph.0001135 (PMC10021387; doi:10.1371/journal.pgph.0001135)
Supplement: S1 File — (ZIP) [file pgph.0001135.s004.zip › transcripts/0041.docx]

*A Questionnaire to validate new HIV tests called Cepheid Xpert HIV -1 Quay assay (Cepheid) in your hospital*

DET 0041

1. How would you as a parent/guardian feel if your child was to undergo HIV testing with Cepheid ?

CG-Atha kumva bwino chifukwa akufuna kuziwa choonadi cha mene mwana ali nthupi

2. What are your thoughts about the Cepheid Xpert HIV -1 Quay assay using whole blood (Cepheid) for testing HIV in children and giving results promptly?

CG-Maganizo awo ndiwokondwela chifukwa njirazi kunalibe

3. How should this approach **Cepheid Xpert HIV -1 Quay assay using whole blood (Cepheid)** be implemented in a hospital? (Probe who should be targeted, why should they be targeted and why?)

CG-Amene wabwela kuchipatala akuyenela kukapeleka uphungu kwa nzawo,komanso akufunika kuyambila ndi ana chifukwa alipo makolo omwe amathawa ku scale akakhala ndi mimba

4. How should issues of privacy of both children and their guardians be maintained?

CG-Chinsinsi chiyenela kukhala ndi mwini osatilayo

5a.What should be the role of parents/guardians in the implementation of **Cepheid Xpert HIV -1 Quay assay using whole blood (Cepheid)**?

CG-Atengepo udindo kumapita koyezesa

b.What information should be provided to ensure that guardians understand the procedures involved?

CG-Auzidwe za ndondomeko zimenezi

6. What should be the role of male partners in the implementation of this approach of **Cepheid Xpert HIV -1 Quay assay using whole blood (Cepheid)** ? (Probe: How should male partners be encouraged to take active role in these approaches?)

CG-Azibambo azibwela kuchipatala ndi mabanja awo kuzayezesa, komanso tiwalimbikise powanyengelela iwowo monga mayi wapakhomo

7. How would your community feel if these approaches were to be implemented in your nearest health facility? (What could be done to encourage community members to participate in these interventions?)

CG-Atha kuchimva bwino chifukwa aziziwa staus yawo koma tifalise uthenga pochitisa msonkhano kuti aliyense azimvele yekha

8. What are some concerns that you and some members in the community might have related to receiving HIV test results of a child?

CG-Nkhawa imakhalapo yoti mwana afa kwa osachimvesa koma kwa ochimvesa amatsatila njila ndikuona mene mwana angamusamalire

9. Do you have suggestions or ideas for addressing possible community concerns about these HIV testing strategies?

CG-Pemphelo ndilomwe lingathese nkhawa komaso kumwa makhwala mwa ndondomeko

B. Perceptions about time to receive test results

10. From the time that your child is tested, how long would you be patient enough to know results from the blood tests? (Same day, after three, after three months?)

Tsiku Lomwelo □√

Patatha masiku □

Miyezi iwiri kapena itatu □

Fotokozani zifukwa zomwe mwasankhira Yankho limeneli

Kuti akamapita kwawo aziwe status ya mwana kuti ngati kuli kolandila chithandizo alandile pompo

11. If your child is tested for HIV, how long would you want to wait before you are told that results from the tests are HIV positive? (same day, after three, after three months?)Explain why you would prefer your chosen answer.

Tsiku Lomwelo □√

Patatha masiku □

Miyezi iwiri kapena itatu □

Fotokozani zifukwa zomwe mwasankhira Yankho limeneli

CG-Amafuna adziwe tsiku lomwelo kuti akhale omasuka

CG-Malingana ndikafukufukuyo kuti aziwe bwino ngati ali nako kapena ayi

12. If your child test for HIV, how long would you want to wait before you are told that results from the test are HIV negative? (Same day, after three, after three months?) Explain why you would prefer your chosen answer.

Tsiku Lomwelo □√

Patatha masiku □

Miyezi iwiri kapena itatu □

Fotokozani zifukwa zomwe mwasankhira Yankho limeneli

CG-Chifukwa akhala osangalala ndi nyengo yomwe mwana wawo ali

C.Acceptability and decision making

13. What information would you want to be given to make an informed decision to accept that your child should get an HIV test or not? Explain

14. How would you want to be approached and given information about these two HIV testing strategies? Explain

CG-Madokotala akuyenela kuuza anthu akabwela kuchipatala

D.Potential Social Harms/Concerns etc.

15. Would you encourage other parents/guardians to allow their children to test for HIV using **Cepheid Xpert HIV -1 Quay assay using whole blood (Cepheid)**? What would be your main concerns and worries towards this approach?

CG-Alibepo nkhawa inailiyonse Kamba kanjilazi

16. How would you personally feel is someone from your community learns about HIV test results for your child?

CG-Sadandaula chifukwa akhala wava zam’maluwa choona akhala sakuchiziwa

17. Do you have any other thoughts you wish to share on this topic?

CG-Ganizo lawo ndiloti njilazi zifike ma health centre kuti omwe sanave amve kuti nawo athe kuwayezetsa ana awo

*The Research Team*
