# Supplementary material for: ‘If I am on ART, my new-born baby should be put on treatment immediately’: Exploring the acceptability, and appropriateness of Cepheid Xpert HIV-1 Qual assay for early infant diagnosis of HIV in Malawi
Source: PLOS Glob Public Health. 2023 Mar 10;3(3):e0001135. doi: 10.1371/journal.pgph.0001135 (PMC10021387; doi:10.1371/journal.pgph.0001135)
Supplement: S1 File — (ZIP) [file pgph.0001135.s004.zip › transcripts/DET 0038.docx]

*A Questionnaire to validate new HIV tests called Cepheid Xpert HIV -1 Quay assay (Cepheid) in your hospital*

DET 0038

1. How would you as a parent/guardian feel if your child was to undergo HIV testing with Cepheid Xpert HIV -1 Quay assay using whole blood (Cepheid) ?

CG-Ine ndingamve bwino chifukwa ndikamva mwana mene alili ndizaziwa mene alili

2. What are your thoughts about this new strategy **Cepheid Xpert HIV -1 Quay assay using whole blood (Cepheid)** for testing HIV in children and giving results promptly?

CG-Ineyo ndikuona kuti njira imeneyi ndiyabwino chifukwa itithandizira kuziwa za nthupi la mwana

3. How should this **Cepheid Xpert HIV -1 Quay assay using whole blood (Cepheid)** approach be implemented in a hospital? (Probe who should be targeted, why should they be targeted and why?)

CG-Pozera msokhano

CG-Tiyambire ana chifukwa njira zimenezi akulu alinazo kale

4. How should issues of privacy of both children and their guardians be maintained?

CG-Makolo ndi madotoo akuyenera kusunga chinsisi

5a.What should be the role of parents/guardians in the implementations of these approaches?

CG-Gawo limene tikatenge ndikutengapo gawo poyezesa magazi

b.What information should be provided to ensure that guardians understand the procedures involved?

CG-Auzidwe masamalidwe ndi matengedwe amatenda amenewa

6. What should be the role of male partners in the implementation of these approaches? (Probe: How should male partners be encouraged to take active role in these approaches?)

CG-Azibambo akungoyenera kubwera kuzayezesa ndipo kuwalangiza ubwino wake wowayezetsa ana kuti akhale ndi umoyo wabwino

7. How would your community feel if these approaches were to be implemented in your nearest health facility? (What could be done to encourage community members to participate in these interventions?)

CG-Ena atha kuchimva koma ena sangachimve

-pakuyenera kuwauza afumu kuti apange msonkhano wokhuzana ndi njirazi

8. What are some concerns that you and some members in the community might have related to receiving HIV test results of a child?

CG-Nkhawa imakhalapo kuti chifukwa suziwa mene mwana alili nthupi

9. Do you have suggestions or ideas for addressing possible community concerns about these HIV testing strategies?

CG-Madakotala apeleke malangizo othesa nkhawa akamadikira Zotsatira

B. Perceptions about time to receive test results

10. From the time that your child is tested, how long would you be patient enough to know results from the blood tests? (Same day, after three, after three months?)

Tsiku Lomwelo □√

Patatha masiku □

Miyezi iwiri kapena itatu □

Fotokozani zifukwa zomwe mwasankhira Yankho limeneli

CG-Kuti uziwe mene thupi mulili ndikuziwa chomwe ungachite malingana ndizotsatilazo

11. If your child is tested for HIV, how long would you want to wait before you are told that results from the tests are HIV positive? (same day, after three, after three months?)Explain why you would prefer your chosen answer.

Tsiku Lomwelo □√

Patatha masiku □

Miyezi iwiri kapena itatu □

Fotokozani zifukwa zomwe mwasankhira Yankho limeneli

Ndi masiku abwino kuti ukamve zenizeni za mene mwana alili

12. If your child test for HIV, how long would you want to wait before you are told that results from the test are HIV negative? (Same day, after three, after three months?)Explain why you would prefer your chosen answer.

Tsiku Lomwelo □√

Patatha masiku □

Miyezi iwiri kapena itatu □

Fotokozani zifukwa zomwe mwasankhira Yankho limeneli

CG-Kuti ulimbe mtima ndi zosatilazo

C.Acceptability and decision making

13. What information would you want to be given to make an informed decision to accept that your child should get an HIV test or not? Explain

CG-Alandire uphungu wachilimbikiso Kamba ka zotsatila

14. How would you want to be approached and given information about these two HIV testing strategies? Explain

CG-Akabwera uphungu wachilimbikiso Kamba ka zotsatila

D.Potential Social Harms/Concerns etc.

15. Would you encourage other parents/guardians to allow their children to test for HIV using these two approaches?

Yes □ √No □

What would be your main concerns and worries towards these approaches?

CG-Nkhawa palibepo ndimayezedwawa

16. How would you personally feel is someone from your community learns about HIV test results for your child?

CG-Sangadandaule chifukwa mwana wawo akhala wathandizidwa

17. Do you have any other thoughts you wish to share on this topic?

CG-Alibe maganizo kapena nkhawa inailiyonse yokhuza mayezedwe a Cephid

*The Research Team*

Participant 0038 akuti akumva bwino chifukwa cha njilazi zomwe zithandizire kuziwa mene mwana wawo alili komanso anali omasuka mukuthandiza kuyankha mafunso onse omwe anali muchipatala chakafukufuku ameneyu

(Participant was very happy to take part in this study and to test her child with **Cepheid Xpert HIV -1 Quay assay using whole blood (Cepheid) and is willing to answer questions and participate freely in this research study if we need to ask more questions.**
