# Supplementary material for: ‘If I am on ART, my new-born baby should be put on treatment immediately’: Exploring the acceptability, and appropriateness of Cepheid Xpert HIV-1 Qual assay for early infant diagnosis of HIV in Malawi
Source: PLOS Glob Public Health. 2023 Mar 10;3(3):e0001135. doi: 10.1371/journal.pgph.0001135 (PMC10021387; doi:10.1371/journal.pgph.0001135)
Supplement: S1 File — (ZIP) [file pgph.0001135.s004.zip › transcripts/DET 0043.docx]

*A Questionnaire to validate new HIV tests called Cepheid Xpert HIV -1 Quay assay using whole blood (Cepheid) in your hospital*

DET 0043

1. How would you as a parent/guardian feel if your child was to undergo HIV testing with Cepheid *Xpert HIV -1 Quay assay using whole blood (Cepheid)*

Atha kukhala osangala chifukwa afuna kuziwa nthupi mwa mwana kuti muli bwanji

2. What are your thoughts about these new strategies of Cepheid *Xpert HIV -1 Quay assay using whole blood (Cepheid)* for testing HIV in children and the time it takes to receive results?

Maganizo awo ndikukondwa ndi njilazi kuti alandila chithandizo malingana ndi ma results

3. How should these approaches of Cepheid *Xpert HIV -1 Quay assay using whole blood (Cepheid)* be implemented in a hospital? (Probe who should be targeted, why should they be targeted and why?)

Tizikhazikitse powuzana kuti tikayezetese komanso tiyambile kuyeza ana chifukwa mwana sangathe kupanga chisankho paiye yekha kuti akayezetse

4. How should issues of privacy of both children and their guardians whilst testing with Cepheid *Xpert HIV -1 Quay assay using whole blood (Cepheid)* be maintained?

A dotolo ndi amene akuyenzetsayo ndi amene akuyenela kusunga chinsinsi

5a.What should be the role of parents/guardians in the implementations of these approaches of Cepheid *Xpert HIV -1 Quay assay using whole blood (Cepheid)*?

Makolo atenge ana awo ndikukayezetsa

b.What information should be provided to ensure that guardians understand the procedures involved?

A chipata adziwe kufunika kwa Cepheid

6. What should be the role of male partners in the implementation of these approaches of Cepheid *Xpert HIV -1 Quay assay using whole blood (Cepheid)*? (Probe: How should male partners be encouraged to take active role in these approaches?)

Awuzidwe kuti nawonso akayezetse pakubwela nawo ku chipatala kuno

7. How would your community feel if these approaches of Cepheid *Xpert HIV -1 Quay assay using whole blood (Cepheid)* were to be implemented in your nearest health facility? (What could be done to encourage community members to participate in these interventions?)

Atha kukhala osangalala chifukwa zawafupikila kusiyana mtunda wa utali kwambiri komanso a chipatala ndi a mfumu akuyenela kulimbitsa anthu mtima kuti akayezetse

8. What are some concerns that you and some members in the community might have related to receiving HIV test results of a child?

Ena amadandawula kuti ukapezeka ndi H.I.V uzimwa ma ARV moyo wako wonse.

9. Do you have suggestions or ideas for addressing possible community concerns about these HIV testing strategies of Cepheid *Xpert HIV -1 Quay assay using whole blood (Cepheid)*?

Kuwalangiza kuti asamadandawule chifukwa ayamba kulandila chithandizo akapezeka ndi H.I.V

B. Perceptions about time to receive test results

10. From the time that your child is tested, how long would you be patient enough to know results from the blood tests? (Same day, after three, after three months?)

Tsiku Lomwelo □

Patatha masiku □

Miyezi iwiri kapena itatu □

Fotokozani zifukwa zomwe mwasankhira Yankho limeneli

Kuti aziwe m’mene thupi mwa mwana mulili

11. If your child is tested for HIV, how long would you want to wait before you are told that results from the tests are HIV positive? (same day, after three, after three months?)Explain why you would prefer your chosen answer.

Tsiku Lomwelo □

Patatha masiku □

Miyezi iwiri kapena itatu □

Fotokozani zifukwa zomwe mwasankhira Yankho limeneli

Chifukwa kwawo ndikutali kuti abwele kuzangomva zosatila

12. If your child test for HIV, how long would you want to wait before you are told that results from the test are HIV negative? (Same day, after three, after three months?)Explain why you would prefer your chosen answer.

Tsiku Lomwelo □

Patatha masiku □

Miyezi iwiri kapena itatu □

Fotokozani zifukwa zomwe mwasankhira Yankho limeneli

Chifukwa kamatha kubisala ka chilombo nde Patatha Miyezi itatu

C.Acceptability and decision making

13. What information would you want to be given to make an informed decision to accept that your child should get an HIV test or not? Explain

Awuzidwe zowalimbitsa mtima kuti ngati mwana alibe H.I.V amusamale komanso ngati ali ndi H.I.V ayamba kulandila chithandizo

14. How would you want to be approached and given information about these two HIV testing strategies of Cepheid *Xpert HIV -1 Quay assay using whole blood (Cepheid)*? Explain

Akabwela ku chipatala afotokozeledwe za njilazi

D.Potential Social Harms/Concerns etc.

15. Would you encourage other parents/guardians to allow their children to test for HIV using Cepheid *Xpert HIV -1 Quay assay using whole blood (Cepheid)*? What would be your main concerns and worries towards this approach?

Yes □ No □

Alibepo Nkhawa ina iliyonse

16. How would you personally feel is someone from your community learns about HIV test results for your child?

Atha kukhala okhumudwa chifukwa iwowo monga mokhala kholo akuyenela kuziwa zotsatilazo

17. Do you have any other thoughts you wish to share on this topic of testing with Cepheid *Xpert HIV -1 Quay assay using whole blood (Cepheid)*?

Alibepo Nkhawa ina iliyonse koma maganizo awo ndiwoti njilazi zipitilile

*The Research Team*
