# Supplementary material for: ‘If I am on ART, my new-born baby should be put on treatment immediately’: Exploring the acceptability, and appropriateness of Cepheid Xpert HIV-1 Qual assay for early infant diagnosis of HIV in Malawi
Source: PLOS Glob Public Health. 2023 Mar 10;3(3):e0001135. doi: 10.1371/journal.pgph.0001135 (PMC10021387; doi:10.1371/journal.pgph.0001135)
Supplement: S1 File — (ZIP) [file pgph.0001135.s004.zip › transcripts/DET 0045.docx]

*A Questionnaire to validate new HIV tests called Cepheid Xpert HIV -1 Quay assay (Cepheid) in your hospital*

DET 0038

1. How would you as a parent/guardian feel if your child was to undergo HIV testing with Cepheid?

Atha kumva bwino chifukwa aziw amene angatumizire akava zotsatilazo

CG- I would feel good because how to act after hearing the results

2. What are your thoughts about these new strategies for testing HIV in children and giving results promptly?

Alibepo maganizo aliwonse.

CG- No thoughts on this

3. How should these approaches be implemented in a hospital? (Probe who should be targeted, why should they be targeted and why?)

-Aziuzidwa akabwera kuchipatala kuno za Cepheid

CG- They should be taught after coming here to the hospital

-tiyambile ana chifukwa ndondomeko yakuyeza ana pakadali pano palibe koma ya akulu ilipo

CG - we should start with children because we already have adult testing methods

4. How should issues of privacy of both children and their guardians be maintained?

Chinsinsi chikuyenela kukhala ndi kholo la mwana

CG- The secret should be with the child’s parents

5a.What should be the role of parents/guardians in the implementations of these approaches?

Gawo lomwe angatengepo ndikubwelesa mwana kuti azayezedwe

CG- They should take part by bringing their child to the hospital for testing.

b.What information should be provided to ensure that guardians understand the procedures involved?

Mene wachimvera chifukwa ena olo auzidwe uphungu saugwilisaso ntchito ayi

CG- It depends on how you receive it because some receive counselling and don’t use it.

6. What should be the role of male partners in the implementation of these approaches? (Probe: How should male partners be encouraged to take active role in these approaches?)

-Azimayi akuyenela kuzawauza azimuna awo zaubwio wanjirazi

CG- Women must tell their husband’s the importance of these strategies.

7. How would your community feel if these approaches were to be implemented in your nearest health facility? (What could be done to encourage community members to participate in these interventions?)

-Ena atha kuchimva koma ena sangachimve

CG- some might accept it, some can not

-pakuyenera kuwauza afumu kuti apange msonkhano wokhuzana ndi njirazi

CG- The village chief must hold a convention concerning the strategies

8. What are some concerns that you and some members in the community might have related to receiving HIV test results of a child?

Nkhawa imakhalapo kuti chifukwa suziwa mene mwana alili nthupi

CG- Concern is there because you don’t know the child’s status

9. Do you have suggestions or ideas for addressing possible community concerns about these HIV testing strategies?

Madakotala apelike malangiso athesa nkhawa akamadikira Zotsatira

Doctors should offer counselling to reduce the persons stress when waiting for the results.

B. Perceptions about time to receive test results

10. From the time that your child is tested, how long would you be patient enough to know results from the blood tests? (Same day, after three, after three months?)

Tsiku Lomwelo □

Patatha masiku □

Miyezi iwiri kapena itatu □

Fotokozani zifukwa zomwe mwasankhira Yankho limeneli

Ndiziwe kuti mwana wanga ali bwanji ndikudziwa momutetedzera

CG- To know my child’s status and how I should care for him/her

11. If your child is tested for HIV, how long would you want to wait before you are told that results from the tests are HIV positive? (same day, after three, after three months?)Explain why you would prefer your chosen answer.

Tsiku Lomwelo □

Patatha masiku □

Miyezi iwiri kapena itatu □

Fotokozani zifukwa zomwe mwasankhira Yankho limeneli

Powona ulendo wamayendedwe anga Ndikuwona kuti bola Patatha masiku

CG- With consideration on transport I would prefer 3days

12. If your child test for HIV, how long would you want to wait before you are told that results from the test are HIV negative? (Same day, after three, after three months?)Explain why you would prefer your chosen answer.

Tsiku Lomwelo □

Patatha masiku □

Miyezi iwiri kapena itatu □

Fotokozani zifukwa zomwe mwasankhira Yankho limeneli

Pamenepa ndilibe ganizo lililonse

CG- No thoughts on this

C.Acceptability and decision making

13. What information would you want to be given to make an informed decision to accept that your child should get an HIV test or not? Explain

Ndikuyenera kupatsidwa uphungu woyenera posawona nkhope ndikutilangiza bwino.

CG- I need to receive good counselling without discrimination

14. How would you want to be approached and given information about these two HIV testing strategies? Explain

Ine ndasankha kundifikila kuno ku chipatala kundilangiza za Cepheid

CG- I choose finding me here in the hospital and telling me about Cepheid

D.Potential Social Harms/Concerns etc.

15. Would you encourage other parents/guardians to allow their children to test for HIV using these two approaches? What would be your main concerns and worries towards these approaches?

Yes □ No □

Nkhawa yanga ili poti mwana atatengera matenda ndikhonza kudandawula ndithu

CG- My concern is that if my child has the virus, I would be extremely sad.

16. How would you personally feel is someone from your community learns about HIV test results for your child?

Ndikuwona kuti zingavute chifukwa anthufe ndiwosiyana mitima chifukwa Akhonza kumalalika m’mudzi

CG- It would be hard because we have different hearts and someone might preach my results to the community.

17. Do you have any other thoughts you wish to share on this topic?

Ine ndilibe Nkhawa kapena ganizo lililonse

CG- No problem with this

*The Research Team*
