# Supplementary material for: ‘If I am on ART, my new-born baby should be put on treatment immediately’: Exploring the acceptability, and appropriateness of Cepheid Xpert HIV-1 Qual assay for early infant diagnosis of HIV in Malawi
Source: PLOS Glob Public Health. 2023 Mar 10;3(3):e0001135. doi: 10.1371/journal.pgph.0001135 (PMC10021387; doi:10.1371/journal.pgph.0001135)
Supplement: S1 File — (ZIP) [file pgph.0001135.s004.zip › transcripts/DET 0047.docx]

*A Questionnaire to validate new HIV tests called Cepheid Xpert HIV -1 Quay assay (Cepheid) and in your hospital*

DET 0047

1. How would you as a parent/guardian feel if your child was to undergo HIV testing with Cepheid & ?

Atha kumva bwino chifukwa akamva zotsatila amuteteza kamba zotsatilanzo

CG- I would feel good because after hearing the results I will care for the child based from that

2. What are your thoughts about these new strategies for testing HIV in children and giving results promptly?

Alibepo ganizo lililonse

CG- No thoughts on this

3. How should these approaches be implemented in a hospital? (Probe who should be targeted, why should they be targeted and why?)

Munthu akabwela ku chipatala mpamene aziwuzidwa ndi njira imodzi yomwe tingakhazikitsile makamaka tiyambe ndi mayi chifukwa mayi ndi amene amayamwitsa mwana nde atha kumupatsila

CG- People should be told about testing after coming to the hospital is one way of establishing this and we should start we women because they breast feed children.

4. How should issues of privacy of both children and their guardians be maintained?

Ukakhala wayezetsa usamangowuza anthu zotsatila ukuyenela kusunga chinsinsi

CG- After getting tested you shouldn’t just tell everyone about your results.

5a.What should be the role of parents/guardians in the implementations of these approaches?

Azimayi akuyenela kuzipeleka pakayezedwe ka Cepheid ndi

CG-Women should take part in this testing method of Cepheid.

b.What information should be provided to ensure that guardians understand the procedures involved?

Alibe ganizo

CG-No thoughts

6. What should be the role of male partners in the implementation of these approaches? (Probe: How should male partners be encouraged to take active role in these approaches?)

Azibambo azibwela nawonso kuzayezetsa komanso Azimayi alimbikitse azibambo amene sali willing ndikuyezetsa

CG- Men should also becoming for testing and women should also encourage men who are not willing to get tested.

7. How would your community feel if these approaches were to be implemented in your nearest health facility? (What could be done to encourage community members to participate in these interventions)

Angachimve bwino chifukwa aliyense amafuna kudziwa zotsatila a chipatala akufunika kuwalimbikitsa ndi mayezedwe a Cepheid ndi

CG- I would feel good because everyone wants to know their results and the hospital must encourage people about testing using Cepheid.

8. What are some concerns that you and some members in the community might have related to receiving HIV test results of a child?

Alibe nkhawa chifukwa amakhala akufuna mwana athandizidwe

CG- No concern because it is all for helping the child.

9. Do you have suggestions or ideas for addressing possible community concerns about these HIV testing strategies?

Komanso anthu amene amakhala ndi nkhawa alimbikitsidwe kuti ngati akupeza nako ka chirombo simathelo azonse

CG- Those with fear should be told that If found infected it is not the end of everything.

B. Perceptions about time to receive test results

10. From the time that your child is tested, how long would you be patient enough to know results from the blood tests? (Same day, after three, after three months?)

Tsiku Lomwelo □

Patatha masiku □

Miyezi iwiri kapena itatu □

Fotokozani zifukwa zomwe mwasankhira Yankho limeneli

Kuti ngati wapezeka nako ka chirombo ka alandile chithandizo

CG- So that if found with the virus he/she should receive medical help.

11. If your child is tested for HIV, how long would you want to wait before you are told that results from the tests are HIV positive? (same day, after three, after three months?)Explain why you would prefer your chosen answer.

Tsiku Lomwelo ●□

Patatha masiku □

Miyezi iwiri kapena itatu □

Fotokozani zifukwa zomwe mwasankhira Yankho limeneli

Alibepo ganizo lomwe anenela Tsiku Lomwelo

CG- No reason for choosing same day

12. If your child test for HIV, how long would you want to wait before you are told that results from the test are HIV negative? (Same day, after three, after three months?)Explain why you would prefer your chosen answer.

Tsiku Lomwelo □

Patatha masiku □

Miyezi iwiri kapena itatu □

Fotokozani zifukwa zomwe mwasankhira Yankho limeneli

Mulimose momwe a chipatala angatulutsile zotsatilanzo

CG- Any time the hospital chooses to release the results

C.Acceptability and decision making

13. What information would you want to be given to make an informed decision to accept that your child should get an HIV test or not? Explain

Zili ndiyiwowa kupanga chisankho sakudikilanso uphungu waku chipatala ayi

CG- It is up to me, without need for the hospitals counsel.

14. How would you want to be approached and given information about these two HIV testing strategies? Explain

Akabwela ku chipatala mpamene Awuzidwe za mayezedwe a Cepheid ndi

CG- I should be told about testing after coming to the hospital

D.Potential Social Harms/Concerns etc.

15. Would you encourage other parents/guardians to allow their children to test for HIV using these two approaches? What would be your main concerns and worries towards these approaches?

Yes □ No □

Alibepo nkhawa yina iliyonse ndinjilazi

CG- No worries about this.

16. How would you personally feel is someone from your community learns about HIV test results for your child?

Sangamve bwino chifukwa chikuyenela kukhalapo

CG- I Would not feel good because privacy is necessary

17. Do you have any other thoughts you wish to share on this topic?

Alibepo nkhawa kapena Maganizo aliwonse okhudzana ndi njilazi

CG- No further thoughts.

*The Research Team*
