# Supplementary material for: ‘If I am on ART, my new-born baby should be put on treatment immediately’: Exploring the acceptability, and appropriateness of Cepheid Xpert HIV-1 Qual assay for early infant diagnosis of HIV in Malawi
Source: PLOS Glob Public Health. 2023 Mar 10;3(3):e0001135. doi: 10.1371/journal.pgph.0001135 (PMC10021387; doi:10.1371/journal.pgph.0001135)
Supplement: S1 File — (ZIP) [file pgph.0001135.s004.zip › transcripts/DET 0048.docx]

*A Questionnaire to validate new HIV tests called Cepheid Xpert HIV -1 Quay assay (Cepheid) in your hospital*

DET 0048

1. How would you as a parent/guardian feel if your child was to undergo HIV testing with Cepheid?

Ine ndingamve bwino chifukwa ndikufuna ndimve m’mene alili ndipo ndikudziwa m’mene ndingamusamalire

CG- I would feel good because I would want to know how my child is and how to take care of her.

2. What are your thoughts about these new strategies for testing HIV in children and giving results promptly?

Maganizo anga ndi abwino chifukwa choti ndizaziwa mwachangu m’mene mwana alili kuti apatsidwe chithandizo choyenela

CG- It is a good idea because I will know early on how my child is and how to help.

3. How should these approaches be implemented in a hospital? (Probe who should be targeted, why should they be targeted and why?)

Muyambire kutiwuza n’chipatala momuno ndipo ndasankha kuti ana ayambilire chifukwa iwo ndiwofunika

CG- It should start here in the hospital and I choose children because they are important

4. How should issues of privacy of both children and their guardians be maintained?

Chinsinsi chikuyenera kukhalapo pakati pa dotolo ndi kholo la mwana

CG- The secret should be between the doctor and parent

5a. What should be the role of parents/guardians in the implementations of these approaches?

Ndikhonza kuwafotokozera anzanga ku nyumba kuti kwabwera njira zatsopano zoyezera ana magazi zotchedwa Cepheid ndi kuti titezele ana athu

CG- I would explain to my friends at home about the new method of HIV testing called Cepheid and how it can help our children.

b.What information should be provided to ensure that guardians understand the procedures involved?

6. What should be the role of male partners in the implementation of these approaches? (Probe: How should male partners be encouraged to take active role in these approaches?)

-Azibambo akungoyenera kutenga gawo pozayezetsa ndikudziwa kuti thupi mwawo muli bwanji ndi kuzitetezera

CG -Men should be taking part by getting tested to know their HIV status

-Tikungoyenera kuwawunikira za ubwino wayezetsa

CG- we should enlighten the importance of testing

7. How would your community feel if these approaches were to be implemented in your nearest health facility? (What could be done to encourage community members to participate in these interventions)

Angamwe bwino ngati tawona kuti mwana wathu salibwino tikuyenera kuthamangira ku chipatala ndikulandira chithandizo

CG- They would feel good knowing that the help they need for their child is readily available

8. What are some concerns that you and some members in the community might have related to receiving HIV test results of a child?

Ine nkhawa sindingakhale nayo chifukwa choti ndikufuna kuthandizidwa

CG- I wouldn’t have any concerns because I want to be helped

9. Do you have suggestions or ideas for addressing possible community concerns about these HIV testing strategies?

Powalimbikitsa anzathu kuti simathero azonse akapezeka ndikachirombo ndikukhala opanda sankho

CG- By encouraging our friends that its not the end of everything if they are found positive and not discriminating them.

B. Perceptions about time to receive test results

10. From the time that your child is tested, how long would you be patient enough to know results from the blood tests? (Same day, after three, after three months?)

Tsiku Lomwelo □

Patatha masiku □

Miyezi iwiri kapena itatu □

Fotokozani zifukwa zomwe mwasankhira Yankho limeneli

Pamenepa ndilibe Yankho

CG- No idea

11. If your child is tested for HIV, how long would you want to wait before you are told that results from the tests are HIV positive? (same day, after three, after three months?)Explain why you would prefer your chosen answer.

Tsiku Lomwelo ●□

Patatha masiku ●□

Miyezi iwiri kapena itatu □

Fotokozani zifukwa zomwe mwasankhira Yankho limeneli

Chifukwa choti ndiziwa kuti mwana wanga alibwanji ndikuwalimbikitsa anzanga zawubwino woyezetsa kuzera njira zimenezi

CG- Because I will know how my child is and encourage my friends on the importance of testing using Cepheid methods

12. If your child test for HIV, how long would you want to wait before you are told that results from the test are HIV negative? (Same day, after three, after three months?)Explain why you would prefer your chosen answer.

Tsiku Lomwelo □

Patatha masiku □

Miyezi iwiri kapena itatu □

Fotokozani zifukwa zomwe mwasankhira Yankho limeneli

Apa ndilibe ganizo

CG- No thoughts here

C.Acceptability and decision making

13. What information would you want to be given to make an informed decision to accept that your child should get an HIV test or not? Explain

Tingoyenera kupatsidwa uphungu kuchokera kwa inu a dotolo

CG- We should get counselling from the doctor

14. How would you want to be approached and given information about these two HIV testing strategies? Explain

Mukhonza kundifukira kuzera kunyumba kwanga

CG- You can reach me at my house

D.Potential Social Harms/Concerns etc.

15. Would you encourage other parents/guardians to allow their children to test for HIV using these two approaches? What would be your main concerns and worries towards these approaches?

Yes □ No □

Ndilibe vuto koma nkhawa yanga ndiyomutenga mwana magazi pamsempha chifukwa choti ndimawona ngati akhala ndi bala

CG- I have no problem but my fear is the venous blood draw. I am afraid of it leaving a wound on the puncture site.

16. How would you personally feel is someone from your community learns about HIV test results for your child?

17. Do you have any other thoughts you wish to share on this topic?

Nkhawa yanga ndiyokuti mwina magazi amene mwamutengawo akhonza kudwala kuti mwachotsa magazi ndiye atadwala ndikuyenera kumutani kuti akhalenso bwino

CG- My worry is on the fact that the blood you have taken might make the child sick and I wouldn’t know what to do then

*The Research Team*
