# Supplementary material for: ‘If I am on ART, my new-born baby should be put on treatment immediately’: Exploring the acceptability, and appropriateness of Cepheid Xpert HIV-1 Qual assay for early infant diagnosis of HIV in Malawi
Source: PLOS Glob Public Health. 2023 Mar 10;3(3):e0001135. doi: 10.1371/journal.pgph.0001135 (PMC10021387; doi:10.1371/journal.pgph.0001135)
Supplement: S1 File — (ZIP) [file pgph.0001135.s004.zip › transcripts/DET 0050.docx]

*A Questionnaire to validate new HIV tests called Cepheid Xpert HIV -1 Quay assay (Cepheid) in your hospital*

DET 0050

1. How would you as a parent/guardian feel if your child was to undergo HIV testing with Cepheid?

Ine ndingamve kukoma chifukwa choti ndikufuna kudziwa m’mene mwana wanga alili ndikudziwa m’mene ndingamuthandizile ngati pakufunika chithandizo t

CG- I would like it so much because I want to know my child’s status and how I may help him/her if need arises

2. What are your thoughts about these new strategies for testing HIV in children and giving results promptly?

Maganizo anga ndiwokuti ndingodikila kuti zotsatira m’mene tingawathandizire komanso ndikuwona kuti njirazi zachita bwino zabwera

I think we should just wait for the results and how see how we can help and I am also thankful for these methods

3. How should these approaches be implemented in a hospital? (Probe who should be targeted, why should they be targeted and why?)

-Kuzera muchipatala

- Tiyambire ana chifukwa anasamaziwa kanthu ndiye ndi bwino kuwawongolera anawa

CG- Using Hospitals

CG- We should start with children because they don’t know anything

4. How should issues of privacy of both children and their guardians be maintained?

Zimatengera oyezawo kuti asunge chinsinsi chifukwa chimakhala chagona m’manja mwawo

CG- It depends on the one doing the test because it is in their hands

5a.What should be the role of parents/guardians in the implementations of these approaches?

Powafotokozera anzathu kuti agwilitse ntchito njirazi kuti ndizabwino

CG- By explaining to their friends the importance of this method

b.What information should be provided to ensure that guardians understand the procedures involved?

Awuzidwe ubwino wa njirazi kuti atsatire

CG- They should be taught the importance of this method.

6. What should be the role of male partners in the implementation of these approaches? (Probe: How should male partners be encouraged to take active role in these approaches?)

-azibambonso ndiwudindo wawo wotenga gawo kuyezetsa ana awo

CG- It is a man’s duty to take part in this

- Tikungoyenera kuwafotokozera za ubwino woyezetsa magaz

CG- We should be taught the importance of blood testing

7. How would your community feel if these approaches were to be implemented in your nearest health facility? (What could be done to encourage community members to participate in these interventions)

Angamve bwino chifukwa mayendedwe m’mudzi amakhala wovuta kuwafotokozera za ubwino oyezetsa magazi

CG- They would feel good because transport is difficult in rural areas. And explaining the importance of blood testing.

8. What are some concerns that you and some members in the community might have related to receiving HIV test results of a child?

Ndikhonza kuchilandira bwino chifukwa choti ndiziwa momutetezera mwana wanga

CG- I would receive it well because I would know ways of taking care for my child.

9. Do you have suggestions or ideas for addressing possible community concerns about these HIV testing strategies?

Kuwalimbikitsa ndi njira yabwino komanso kuwalangiza kuti akayezetse mwana ndikudziwa m’mene mwana alili

CG- Motivating them that it is a good way and counselling them into getting their child tested.

B. Perceptions about time to receive test results

10. From the time that your child is tested, how long would you be patient enough to know results from the blood tests? (Same day, after three, after three months?)

Tsiku Lomwelo □●

Patatha masiku □●

Miyezi iwiri kapena itatu □

Fotokozani zifukwa zomwe mwasankhira Yankho limeneli

Ndasankha chonchi chifukwa choti ndi kufuna ndiziwa m’mene alili

I chose this because I want to know how he is

11. If your child is tested for HIV, how long would you want to wait before you are told that results from the tests are HIV positive? (same day, after three, after three months?)Explain why you would prefer your chosen answer.

Tsiku Lomwelo □

Patatha masiku □●

Miyezi iwiri kapena itatu □

Fotokozani zifukwa zomwe mwasankhira Yankho limeneli

Ndasankha Patatha masiku chifukwa choti ndikungoyenera kuziwa chotsatira za mwana ndikulandira malangizo omutetezera

CG- I choose 3 days because I need to Know the results and receive correct guidance.

12. If your child test for HIV, how long would you want to wait before you are told that results from the test are HIV negative? (Same day, after three, after three months?)Explain why you would prefer your chosen answer.

Tsiku Lomwelo □

Patatha masiku □

Miyezi iwiri kapena itatu □

Fotokozani zifukwa zomwe mwasankhira Yankho limeneli

Chifukwa choti ndikuyenera kuziwa momwe ndingasamalire mwana ndikumuteteza

CG- because I have to know how I will take care of my child.

C.Acceptability and decision making

13. What information would you want to be given to make an informed decision to accept that your child should get an HIV test or not? Explain

Sindingayembekezere uphungu wina uliwonse chifukwa ndikuyenera kuchimva ndekha kuti ndikuyenera kuyedzetsa mwana wanga

CG- I would not expect any counselling because it is up to me to get my child tested

14. How would you want to be approached and given information about these two HIV testing strategies? Explain

Zitengera inuyo nokha a chipatala chisankho chanu

CG- It will depend on the choice of the hospital

D.Potential Social Harms/Concerns etc.

15. Would you encourage other parents/guardians to allow their children to test for HIV using these two approaches? What would be your main concerns and worries towards these approaches?

Yes □ No □

Palibe nkhawa iliyonse, basi Tingoyenera kuchilandira chifukwa zithuzi ndizothandiza ife tomwe m’maka maka tikaziwa m’mene thupi lathu lili

CG- I do not have any concerns or worry because this method is there to how our community

16. How would you personally feel is someone from your community learns about HIV test results for your child?

Ndingamve kuwawa chifukwa pamayenera kukhala chinsinsi poyezetsa

CG- I would be feel hurt because there is a need for privacy.

17. Do you have any other thoughts you wish to share on this topic?

Nkhawa kapena Maganizo ndilibe

CG- No problem or concerns

*The Research Team*

Kafukufuku wa DET0050

Iwo akuwona kuti njira zimene zikufuna kukhazikitsidwazi zilo bwino chifukwa kale kunalibe njira zimenezi zoyezera magazi mwachangu

She thinks that this method is very welcome and she is pleased because with this method, they don’t have to wait for too long to hear the results
