# Supplementary material for: ‘If I am on ART, my new-born baby should be put on treatment immediately’: Exploring the acceptability, and appropriateness of Cepheid Xpert HIV-1 Qual assay for early infant diagnosis of HIV in Malawi
Source: PLOS Glob Public Health. 2023 Mar 10;3(3):e0001135. doi: 10.1371/journal.pgph.0001135 (PMC10021387; doi:10.1371/journal.pgph.0001135)
Supplement: S1 File — (ZIP) [file pgph.0001135.s004.zip › transcripts/DET 0051.docx]

*A Questionnaire to validate new HIV tests called Cepheid Xpert HIV -1 Quay assay (Cepheid) a in your hospital*

DET 0051

1. How would you as a parent/guardian feel if your child was to undergo HIV testing with Cepheid?

Atha kumva bwino chifukwa afuna aziwe m’mene mwana alili nthupi

CG- She would feel good because she would know the child’s status

2. What are your thoughts about these new strategies for testing HIV in children and giving results promptly?

Akuwona Kuti njirazi chifukwa ndizachangu

CG- Because it is a faster way

3. How should these approaches be implemented in a hospital? (Probe who should be targeted, why should they be targeted and why?)

Kwa amene ayezetsako azitha kuwuza anzawo mukutelo zikakhazikitsidwa, komanso tiyambile ana chifukwa njira zoyezera akulu akulu zilipo kale

CG- For those who are done with testing they should also tell their friends and we should start with children because methods of testing adults are already there.

4. How should issues of privacy of both children and their guardians be maintained?

Makolo akuyeneleka kusunga chinsinsi mwana akayezedwa

CG- Parents need to keep the results of their child to themselves

5a.What should be the role of parents/guardians in the implementations of these approaches?

Atha kuzayezetsa magazi kuzera njira ya Cepheid

CG- They should come to get tested using Cepheid

B. What information should be provided to ensure that guardians understand the procedures involved?

Afuna uphungu wachilimbikitso kuchokera kwa a chipatala kamba ka Cepheid

CG- They need to be counselled by the hospital stuff

6. What should be the role of male partners in the implementation of these approaches? (Probe: How should male partners be encouraged to take active role in these approaches?)

Azibambo nawonso azayezetse komanso Azimayi alimbikitse azimuna awo

CG- Men should also get tested and women must motivate their husbands.

7. How would your community feel if these approaches were to be implemented in your nearest health facility? (What could be done to encourage community members to participate in these interventions)

Atha kuchilandira bwino chifukwa zawafupikila

CG- They would welcome it

8. What are some concerns that you and some members in the community might have related to receiving HIV test results of a child?

Sakhala ndi nkhawa ina ili yonse chifukwa anakhonzekera kuti china chili chonse chibwele achilandila

CG- I would not have any concerns because I was prepared for any result

9. Do you have suggestions or ideas for addressing possible community concerns about these HIV testing strategies?

Kuwalimbikitsa kuti asadandawule ngati atapezeka nako chifukwa masiku ano nkhani ya AIDS siyobisanso ayi

CG- Encouraging them that not to be worried if found positive

B. Perceptions about time to receive test results

10. From the time that your child is tested, how long would you be patient enough to know results from the blood tests? (Same day, after three, after three months?)

Tsiku Lomwelo □

Patatha masiku □

Miyezi iwiri kapena itatu □

Fotokozani zifukwa zomwe mwasankhira Yankho limeneli

Chifukwa aziwe ngati mwana ali ndi H.I.V kuti ayambe kulandila chithandizo

CG- Because I want to know fast and for my child to start getting assisted in good time

11. If your child is tested for HIV, how long would you want to wait before you are told that results from the tests are HIV positive? (same day, after three, after three months?)Explain why you would prefer your chosen answer.

Tsiku Lomwelo □

Patatha masiku □

Miyezi iwiri kapena itatu □

Fotokozani zifukwa zomwe mwasankhira Yankho limeneli

Chifukwa choti mwana ayambe kulandila chithandizo

CG- For my child to start getting assisted

12. If your child test for HIV, how long would you want to wait before you are told that results from the test are HIV negative? (Same day, after three, after three months?)Explain why you would prefer your chosen answer.

Tsiku Lomwelo □

Patatha masiku □

Miyezi iwiri kapena itatu □

Fotokozani zifukwa zomwe mwasankhira Yankho limeneli

Chifukwa ndi m’mene a chipatala awawuzila

CG- Because that is what the medical personnel said

C.Acceptability and decision making

13. What information would you want to be given to make an informed decision to accept that your child should get an HIV test or not? Explain

Alibe ganizo lililonse

CG- No thoughts on this

14. How would you want to be approached and given information about these two HIV testing strategies? Explain

Akabwela ku chipatala kuno auzidwe malangizo a Cepheid

CG- Get advice about Cepheid after coming to the hospital

D.Potential Social Harms/Concerns etc.

15. Would you encourage other parents/guardians to allow their children to test for HIV using these two approaches? What would be your main concerns and worries towards these approaches?

Yes □ No □

Alibepo nkhawa ina iliyonse ndi njilazi

CG- No worries here.

16. How would you personally feel is someone from your community learns about HIV test results for your child?

Chifukwa panopa nkhani ya AIDS siyobisanso ayi ndipo munthu sangadandawule olo atamva

CG- I would not feel bad because right now issues of HIV/AIDS are everywhere.

17. Do you have any other thoughts you wish to share on this topic?

Kwawo ndikuthokoza chifukwa njira zoyezera kunalibeko ndi kale lonse

CG- I am grateful because we never used to have this methods in the past

*The Research Team*

Participant DET 0051
