# Supplementary material for: ‘If I am on ART, my new-born baby should be put on treatment immediately’: Exploring the acceptability, and appropriateness of Cepheid Xpert HIV-1 Qual assay for early infant diagnosis of HIV in Malawi
Source: PLOS Glob Public Health. 2023 Mar 10;3(3):e0001135. doi: 10.1371/journal.pgph.0001135 (PMC10021387; doi:10.1371/journal.pgph.0001135)
Supplement: S1 File — (ZIP) [file pgph.0001135.s004.zip › transcripts/DET 0052.docx]

*A Questionnaire to validate new HIV tests called Cepheid Xpert HIV -1 Quay assay (Cepheid) in your hospital*

DET 0052

1. How would you as a parent/guardian feel if your child was to undergo HIV testing with Cepheid?

Ine ndingamve bwino kuti mwana ayezedwe kuti aziwe za mthupi mwake, ndikuziwa momutetezera

CG- I would feel good because I would know how my child health is.

2. What are your thoughts about these new strategies for testing HIV in children and giving results promptly?

Ine ndili okondwera chifukwa choti ukaziwa za mwana kuti thupi muli bwanji chifukwa uziwa momutetezera kusiyana kuti anakakhala osayezetsa

CG- I am happy because when you know your child’s status you will know how you care for him/her.

3. How should these approaches be implemented in a hospital? (Probe who should be targeted, why should they be targeted and why?)

Tikabwera ku chipatala mukuyenera kutiwuzila ku chitseko ndipo mukuyenera kuyambira ana chifukwa mwa iwo wokha sangathe ndiye ndikuwona kuti tiyambe kuteteza ana

CG- When we come for testing you need to tell us in a closed room and it should start with children because they cannot do it by themselves.

4. How should issues of privacy of both children and their guardians be maintained?

Munthu ukungoyenera kuziwa wekha ndi a chipatala

CG- Only you and the hospital must know

5a.What should be the role of parents/guardians in the implementations of these approaches?

Gawo limene ndingatenge ndikulimbikitsa makolo anzanga kutengapo ngawo poyezetsa magazi

CG- I would help in explaining this test to my friends so that they can also come to get tested

b.What information should be provided to ensure that guardians understand the procedures involved?

Kutiwuza zakuwopysa kwa matendawa ndikapewedwe kake

CG- Telling us the dangers and prevention of the virus

6. What should be the role of male partners in the implementation of these approaches? (Probe: How should male partners be encouraged to take active role in these approaches?)

Kuwalimbikitsa kuti azayezetse ana awo

CG- Motivating them into getting their children tested

7. How would your community feel if these approaches were to be implemented in your nearest health facility? (What could be done to encourage community members to participate in these interventions)

Atha kumva bwino chifukwa choti njira zimenezi kale kunalibe

CG- I would feel good because this method did not exist before

8. What are some concerns that you and some members in the community might have related to receiving HIV test results of a child?

Nkhawa imakhalapo kuti atamupeza nako inde azilandira koma vuto ndiloti ayambe kumwa adakali wachichepele

CG- The concerns come about that if found positive the child will indeed receive assistance but she is too young

9. Do you have suggestions or ideas for addressing possible community concerns about these HIV testing strategies?

Pamenepo ndilibe ganizo

CG- No idea

B. Perceptions about time to receive test results

10. From the time that your child is tested, how long would you be patient enough to know results from the blood tests? (Same day, after three, after three months?)

Tsiku Lomwelo □

Patatha masiku □

Miyezi iwiri kapena itatu □

Fotokozani zifukwa zomwe mwasankhira Yankho limeneli

Chifukwa choti ndikufuna ndikalingalire bwino bwino ndisanamve zotsatira

CG- Because I need to go home to think about it properly before I receive the results

11. If your child is tested for HIV, how long would you want to wait before you are told that results from the tests are HIV positive? (same day, after three, after three months?)Explain why you would prefer your chosen answer.

Tsiku Lomwelo □

Patatha masiku □

Miyezi iwiri kapena itatu □

Fotokozani zifukwa zomwe mwasankhira Yankho limeneli

Ndasankha choncho chifukwa ndikuyenera ndikakambilane kaye ndi abambo akunyumba

CG- I choose like that because I need to discuss with my husband first.

12. If your child test for HIV, how long would you want to wait before you are told that results from the test are HIV negative? (Same day, after three, after three months?)Explain why you would prefer your chosen answer.

Tsiku Lomwelo □

Patatha masiku □

Miyezi iwiri kapena itatu □

Fotokozani zifukwa zomwe mwasankhira Yankho limeneli

Pamenepo ndilibe ganizo

CG- I have no idea

C.Acceptability and decision making

13. What information would you want to be given to make an informed decision to accept that your child should get an HIV test or not? Explain

Potifotokozera mapewedwe ndi katetezedwe kake kamatendawa

CG- By explaining how we can protect ourselves and prevent this virus

14. How would you want to be approached and given information about these two HIV testing strategies? Explain

Mukuyenera kutifikira kuzera muzipatala ndi kutiyikila zikwagwani muzipatala

CG- You should reach us by using the hospitals and posters in hospitals.

D. Potential Social Harms/Concerns etc.

15. Would you encourage other parents/guardians to allow their children to test for HIV using these two approaches? What would be your main concerns and worries towards these approaches?

Yes □ No □

Ine nkhawa yanga inali poti anakati amupeza nako ndinakakhala okhumudwa

CG- My concern is on the fact if found positive, I would be stressed.

16. How would you personally feel is someone from your community learns about HIV test results for your child?

Sindingamve bwino chifukwa choti nkhani imeneyi imayenera kukhala yachinsisi

CG- I would not be happy because this is supposed to be kept secret

17. Do you have any other thoughts you wish to share on this topic?

Ine ndilibe ganizo kapena nkhawa iliyonse pa nkhaniyi

I have questions

*The Research Team*
