# Supplementary material for: ‘If I am on ART, my new-born baby should be put on treatment immediately’: Exploring the acceptability, and appropriateness of Cepheid Xpert HIV-1 Qual assay for early infant diagnosis of HIV in Malawi
Source: PLOS Glob Public Health. 2023 Mar 10;3(3):e0001135. doi: 10.1371/journal.pgph.0001135 (PMC10021387; doi:10.1371/journal.pgph.0001135)
Supplement: S1 File — (ZIP) [file pgph.0001135.s004.zip › transcripts/DET 0053.docx]

*A Questionnaire to validate new HIV tests called Cepheid Xpert HIV -1 Quay assay (Cepheid) in your hospital*

DET 0053

1. How would you as a parent/guardian feel if your child was to undergo HIV testing with Cepheid?

Atha kumva bwino chifukwa akufuna kuziwa mwana wawo m’mene alili nthupi

CG- I would feel good because I want to know the status of my child.

2. What are your thoughts about these new strategies for testing HIV in children and giving results promptly?

Maganizo

3. How should these approaches be implemented in a hospital? (Probe who should be targeted, why should they be targeted and why?)

Kuwalimbikitsa kuti ana akayezetse komanso tiyambile a chinyamata pokhazikitsa mayezetsedwawa

CG- Motivating them to get children tested and we should start with the youths

4. How should issues of privacy of both children and their guardians be maintained?

Munthu uzipita wekha kukayezetsa osati ndi anthu ambili

CG- Everyone should go for the test alone and not as a group

5a.What should be the role of parents/guardians in the implementations of these approaches?

Gawo lomwe angatengepo ndikuwawuza ena kuti azayezedwe munjila zimenezi za Cepheid komanso

CG- Take part by telling others to get tested using this method of Cepheid

b.What information should be provided to ensure that guardians understand the procedures involved?

Osachita phuma koma malangizo Awuzidwe mwachifatsi komanso awuzidwe za ubwino wakuyezetsa kudzera munjilazi

CG- Giving the counselling slowly without pressure and should be taught the importance of using this test.

6. What should be the role of male partners in the implementation of these approaches? (Probe: How should male partners be encouraged to take active role in these approaches?)

A zibambo awalimbikitse a chinyamata komanso azikazi awo kuti azayezetse.

CG- Men should encourage the youth and their wives to get tested

7. How would your community feel if these approaches were to be implemented in your nearest health facility? (What could be done to encourage community members to participate in these interventions?)

Ena atha kumva bwino koma ena zangamve bwino komanso awuzidwe wanjiladzi kuti alimbikitsidwe

CG- Some would like this but other would not and they need to encouraged to use this method

8. What are some concerns that you and some members in the community might have related to receiving HIV test results of a child?

Amakhala ndi Nkhawa polingalila zotsatila kuti zituluka zotani

CG- Stress over the upcoming results

9. Do you have suggestions or ideas for addressing possible community concerns about these HIV testing strategies?

Anthu amene ali ndi Nkhawa akufunika ndikuwalimbikitsa kuti kukhala ndi ka chirombo sikofunika

CG- Those with fear and concerns must be taught that having the virus is not the end

B. Perceptions about time to receive test results

10. From the time that your child is tested, how long would you be patient enough to know results from the blood tests? (Same day, after three, after three months?)

Tsiku Lomwelo □

Patatha masiku □

Miyezi iwiri kapena itatu □

Fotokozani zifukwa zomwe mwasankhira Yankho limeneli

Chifukwa kudikila ndikopweteka kwambili

CG- It’s because waiting is very painful

11. If your child is tested for HIV, how long would you want to wait before you are told that results from the tests are HIV positive? (same day, after three, after three months?)Explain why you would prefer your chosen answer.

Tsiku Lomwelo □

Patatha masiku □

Miyezi iwiri kapena itatu □

Fotokozani zifukwa zomwe mwasankhira Yankho limeneli

Chifukwa ukayezetsa ndikupita nthawi umayiwalako

CG- Because when you get tested and time passes by you might forget.

12. If your child test for HIV, how long would you want to wait before you are told that results from the test are HIV negative? (Same day, after three, after three months?)Explain why you would prefer your chosen answer.

Tsiku Lomwelo □

Patatha masiku □

Miyezi iwiri kapena itatu □

Fotokozani zifukwa zomwe mwasankhira Yankho limeneli

Alibe ganizo chifukwa chomwe anenela Tsiku lomwelo

CG- No reason as to why I have chosen the same day

C.Acceptability and decision making

13. What information would you want to be given to make an informed decision to accept that your child should get an HIV test or not? Explain

Awawuze ophungu oti akapezeka nako ka chirombo ayamba kulandila thandizo

CG- Counselling of what to do if found with the virus

14. How would you want to be approached and given information about these two HIV testing strategies? Explain

Alibe ganizo lili lonse

CG- No idea on this

D.Potential Social Harms/Concerns etc.

15. Would you encourage other parents/guardians to allow their children to test for HIV using these two approaches? What would be your main concerns and worries towards these approaches?

Yes □ No □

Ngati msempha suwukupezeka Nkhawa imakhalapo kuti ampweteka mwanayo

CG- When they cannot find the vein, there is fear that they might hurt the child.

16. How would you personally feel is someone from your community learns about HIV test results for your child?

Sangamve bwino chifukwa Munthu wamvayo atha kumakafunsila za ma results amwana wawoyo

CG- I would not be happy because the person might spread rumors

17. Do you have any other thoughts you wish to share on this topic?

Maganizo awo ndiwokuti tilimbikitse njilazi chifukwa zithandiza kuti anthu aziwe m’mene ana awo alili ndithu

CG- I think that we should encourage these ways because it is helping people know their children’s health Status.

*The Research Team*
