# Supplementary material for: ‘If I am on ART, my new-born baby should be put on treatment immediately’: Exploring the acceptability, and appropriateness of Cepheid Xpert HIV-1 Qual assay for early infant diagnosis of HIV in Malawi
Source: PLOS Glob Public Health. 2023 Mar 10;3(3):e0001135. doi: 10.1371/journal.pgph.0001135 (PMC10021387; doi:10.1371/journal.pgph.0001135)
Supplement: S1 File — (ZIP) [file pgph.0001135.s004.zip › transcripts/DET 0054.docx]

*A Questionnaire to validate new HIV tests called Cepheid Xpert HIV -1 Quay assay (Cepheid) in your hospital*

DET 0054

1. How would you as a parent/guardian feel if your child was to undergo HIV testing with Cepheid?

Ndikuganiza kuti za bwino chifukwa zitithandiza kudziwa kuti mwana ali bwanji

CG- I think its good because it will help me know my child’s status

2. What are your thoughts about these new strategies for testing HIV in children and giving results promptly?

Ndikuganiza kuti zakhala bwino kuti titiziwa mwachangu kuti mwana ali bwanji

CG- I think it is good that we will know earlier

3. How should these approaches be implemented in a hospital? (Probe who should be targeted, why should they be targeted and why?)

-Muchipatala momuno muzitiwuza

-CG- Telling us here in the hospital

-Ndikuwona kuti muyambire akulu ndi ana chifukwa choti tonse ndi ofunikira

4. How should issues of privacy of both children and their guardians be maintained?

Ukuyenera kumva wekha ndikuzisungira chinsinsi

CG- You need to hear this alone and keep it private

5a.What should be the role of parents/guardians in the implementations of these approaches?

Pamenepo ndilibe ganizo

CG- No idea

b.What information should be provided to ensure that guardians understand the procedures involved?

Akuyenera kuwudzidwa ubwino woyezetsa magazi ndikapewedwe kake

CG- The importance of the test and prevention of the virus

6. What should be the role of male partners in the implementation of these approaches? (Probe: How should male partners be encouraged to take active role in these approaches?)

-Azibambo akuyenera kuzayedzetsabe

CG- Men also need to come for the test

-Tikuyenera tikafikile ku chipatala pompano chifukwa Azibambo amathawa

CG- Men run away from the test so they need to be reached when they come to the hospital

7. How would your community feel if these approaches were to be implemented in your nearest health facility? (What could be done to encourage community members to participate in these interventions?)

-Atha kumva bwino komabe kumva ndikosiyana ena sangachimve

- I would receive it well but others might not understand

-Pakupangisa msonkhano akhonza kutithandiza

- conventions might help

8. What are some concerns that you and some members in the community might have related to receiving HIV test results of a child?

Nkhawa imakhala poti mwana akayezetsa mwina apezeka ndi matenda poti mwana ndi mwana akhonza kuvetsa chisoni kuti azimwa mankhwala tsiku ndi tsiku

CG- Its sad to watch a child take daily ART that is where the concern comes in.

9. Do you have suggestions or ideas for addressing possible community concerns about these HIV testing strategies?

Kuwalimbikitsa kuti asakhale ndi Nkhawa

CG- Encourage them to have no be worried

B. Perceptions about time to receive test results

10. From the time that your child is tested, how long would you be patient enough to know results from the blood tests? (Same day, after three, after three months?)

Tsiku Lomwelo □

Patatha masiku □

Miyezi iwiri kapena itatu □

Fotokozani zifukwa zomwe mwasankhira Yankho limeneli

Tiziwe ndi kukhala womasuka

CG- To know and be free

11. If your child is tested for HIV, how long would you want to wait before you are told that results from the tests are HIV positive? (same day, after three, after three months?)Explain why you would prefer your chosen answer.

Tsiku Lomwelo □

Patatha masiku □

Miyezi iwiri kapena itatu □

Fotokozani zifukwa zomwe mwasankhira Yankho limeneli

12. If your child test for HIV, how long would you want to wait before you are told that results from the test are HIV negative? (Same day, after three, after three months?)Explain why you would prefer your chosen answer.

Tsiku Lomwelo □

Patatha masiku □

Miyezi iwiri kapena itatu □

Fotokozani zifukwa zomwe mwasankhira Yankho limeneli

C.Acceptability and decision making

13. What information would you want to be given to make an informed decision to accept that your child should get an HIV test or not? Explain

Zitengera inu a chipatala kutilangiza zamayezedwewa ndikutipatsa uphungu wabwino

CG- Medical personnel should advise us about the testing and give us counselling

14. How would you want to be approached and given information about these two HIV testing strategies? Explain

Zikutengeranso inu ma dotolo kutiwuza za ubwino wakayezedweka

CG- It will depend on the doctor telling us the importance of testing.

D.Potential Social Harms/Concerns etc.

15. Would you encourage other parents/guardians to allow their children to test for HIV using these two approaches? What would be your main concerns and worries towards these approaches?

Yes □ No □

Nkhawa ndilibe ili yonse, ine kwanga ndikusangalala kuti njira zifunika zikhazikitsidwe

I have no concerns but joy because this needs to established.

16. How would you personally feel is someone from your community learns about HIV test results for your child?

Sindingadandawule koma kungowalimbikitsa kuti nawonso akayezetse mwana wawo

I wouldn’t be sad but rather tell them to get their child tested too.

17. Do you have any other thoughts you wish to share on this topic?

Ine ganizo kapena Nkhawa ndilibe

I don’t have any more concerns

*The Research Team*
