# Supplementary material for: ‘If I am on ART, my new-born baby should be put on treatment immediately’: Exploring the acceptability, and appropriateness of Cepheid Xpert HIV-1 Qual assay for early infant diagnosis of HIV in Malawi
Source: PLOS Glob Public Health. 2023 Mar 10;3(3):e0001135. doi: 10.1371/journal.pgph.0001135 (PMC10021387; doi:10.1371/journal.pgph.0001135)
Supplement: S1 File — (ZIP) [file pgph.0001135.s004.zip › transcripts/DET 0055.docx]

*A Questionnaire to validate new HIV tests called Cepheid Xpert HIV -1 Quay assay (Cepheid) in your hospital*

DET 0055

1. How would you as a parent/guardian feel if your child was to undergo HIV testing with Cepheid?

Atha kumva bwino chifukwa akayezedwa aziwa m’mene alili kuti ali ndi tsogolo labwino kapena ayi

CG- I would feel good because when tested I will know if the child has bright future or not.

2. What are your thoughts about these new strategies for testing HIV in children and giving results promptly?

Ndizabwino njirazi chifukwa aziwa ana m’mene alili chifukwa utha kumangomusunga mwana kumati ali bwino bwino pamene ali ndi H.I.V

CG- It’s a good strategy because I will know how their child is because without testing you may just keep an HIV positive child without knowing.

3. How should these approaches be implemented in a hospital? (Probe who should be targeted, why should they be targeted and why?)

Komanso tifikile m’midzi chifukwa anthu a m’midzi amavutika mayendedwe komanso tiyambe ana kuti tidizwe za tsogolo la mwanayo

CG- We should target rural areas because people in the village areas have difficulties with travelling and we should start with children to know their future

4. How should issues of privacy of both children and their guardians be maintained?

Akabwera kuzayezetsa uzingozisungila mwawekha komanso ma dotolo asunge chinsinsi

CG- When we come for testing keep the results should be kept only by us parents and the doctors.

5a. What should be the role of parents/guardians in the implementations of these approaches?

Alibe ganizo lililonse

CG- No idea

b.What information should be provided to ensure that guardians understand the procedures involved?

Akabwera ku scale afotokozeledwe za ubwino za njira za Cepheid

CG- When they come for antenatal clinic, Cepheid should be explained

6. What should be the role of male partners in the implementation of these approaches? (Probe: How should male partners be encouraged to take active role in these approaches?)

Azibambo afotokozeledwe kuti azibwera kuzayezetsa kuti aziwe m’mene alili

CG-Men should be told the importance of testing so they can know their statuses

7. How would your community feel if these approaches were to be implemented in your nearest health facility? (What could be done to encourage community members to participate in these interventions)

Atha kumva bwino chifukwa aziwa m’mene nthupi mwawo mulili komanso afotokozeledwe za ubwino za Cepheid

CG- they would like it because they would know the status of their children and explain to them the importance of ceiphed

8. What are some concerns that you and some members in the community might have related to receiving HIV test results of a child?

Sakhala ndi nkhawa chifukwa amakhala akukhonza tsogolo la mwana

CG- I would not have concerns because it is for fixing my child’s future

9. Do you have suggestions or ideas for addressing possible community concerns about these HIV testing strategies?

Komanso kwa amene amakhala ndi nkhawa awuzidwe kuti sangafe Tsiku lomwelo koma angotsatila zomwe a chipatala awawuza.

CG- Those with concerns should be told it cannot kill them on the same day but they should follow the doctor’s instruction

B. Perceptions about time to receive test results

10. From the time that your child is tested, how long would you be patient enough to know results from the blood tests? (Same day, after three, after three months?)

Tsiku Lomwelo □

Patatha masiku □

Miyezi iwiri kapena itatu □

Fotokozani zifukwa zomwe mwasankhira Yankho limeneli

Chifukwa tsiku lomwelo amakhala akukapanga zinthu zina ku mudzi nde bola Patatha masiku

CG- she has other things to do so she can not wait to get the results the same day

11. If your child is tested for HIV, how long would you want to wait before you are told that results from the tests are HIV positive? (same day, after three, after three months?)Explain why you would prefer your chosen answer.

Tsiku Lomwelo □

Patatha masiku □

Miyezi iwiri kapena itatu □

Fotokozani zifukwa zomwe mwasankhira Yankho limeneli

Chifukwa amakhala kuti achipatala ndi womwe awafotokozela kuti abwele Patatha masiku

CG- because it is usually the hospital personnel that tells us to wait for a few days

12. If your child test for HIV, how long would you want to wait before you are told that results from the test are HIV negative? (Same day, after three, after three months?)Explain why you would prefer your chosen answer.

Tsiku Lomwelo □

Patatha masiku □

Miyezi iwiri kapena itatu □

Fotokozani zifukwa zomwe mwasankhira Yankho limeneli

Chifukwa uzisangalala kuti mwana ali bwino bwino komanso kumutetedza kuti asatenge AIDS

CG- Because it will nice to know the child is alright and protected from contracting HIV

C.Acceptability and decision making

13. What information would you want to be given to make an informed decision to accept that your child should get an HIV test or not? Explain

Awuzidwe uphungu woyenera momwe angamutetezele mwana kuti asatenge matenda

CG- Should Receive the collect counselling on how her child can be protected from contracting HIV and AIDS

14. How would you want to be approached and given information about these two HIV testing strategies? Explain

Kuwafikila ku mudzi ndikuwawuza uphungu umenewu

CG- Reaching communities and telling them about this

D.Potential Social Harms/Concerns etc.

15. Would you encourage other parents/guardians to allow their children to test for HIV using these two approaches? What would be your main concerns and worries towards these approaches?

Yes □ No □

Alibepo nkhawa chifukwa akufuna kuti aziwe m’mene mwana alili nthupi

No concern because it is for the child’s wellbeing

16. How would you personally feel is someone from your community learns about HIV test results for your child?

Sangamve bwino chifukwa munthuyo azifalitsa za m’mene mwanayo alili komanso kuyezetsa magazi kumafunika kukhala kwa chinsinsi

CG- I would not feel good because the person would be spreading news about my child and it supposed to a private

17. Do you have any other thoughts you wish to share on this topic?

Alibepo maganizo kapena nkhawa pa nkhaniyi

No thoughts on this

*The Research Team*
