# Supplementary material for: ‘If I am on ART, my new-born baby should be put on treatment immediately’: Exploring the acceptability, and appropriateness of Cepheid Xpert HIV-1 Qual assay for early infant diagnosis of HIV in Malawi
Source: PLOS Glob Public Health. 2023 Mar 10;3(3):e0001135. doi: 10.1371/journal.pgph.0001135 (PMC10021387; doi:10.1371/journal.pgph.0001135)
Supplement: S1 File — (ZIP) [file pgph.0001135.s004.zip › transcripts/DET 0056.docx]

*A Questionnaire to validate new HIV tests called Cepheid Xpert HIV -1 Quay assay (Cepheid) in your hospital*

DET 0056

1. How would you as a parent/guardian feel if your child was to undergo HIV testing with Cepheid?

Ndikuwona kuti ndi njira yabwino zithandiza kuchepetsa imfa za ana zomwe zimabwera kamba kamatendawa

CG- I think this is a good way and it will help reduce child death which comes about due to the virus.

2. What are your thoughts about these new strategies for testing HIV in children and giving results promptly?

Ndikuwona kuti njirazi ndizothandiza

CG- I think it is helpful

3. How should these approaches be implemented in a hospital? (Probe who should be targeted, why should they be targeted and why?)

Ndikuwona kuti mupeze nthawi yoyenda m’mudzi ndikulangiza za ubwino woyezetsa ndikuwona kuti Muyambire ana chifukwa choti nthawi yonseyi njira za ana zoyezetera magazi kunalibe

CG- Find time and spread the news about the test to our communities and start with children because all this time, there was no method for testing children.

4. How should issues of privacy of both children and their guardians be maintained?

Chinsinsi chikuyenera kukhala ndi inu a chipatala ndife tomwe

CG- The secret should be between the hospital and parents

5a.What should be the role of parents/guardians in the implementations of these approaches?

Ndikuyenera kuwafotokozera anzanga m’mudzi za ubwino woyezetsa kuti nawonso azayezetse nawonso ana awo

CG- I need to explain to my friend at the village the importance of this test so that can also get their children tested.

b.What information should be provided to ensure that guardians understand the procedures involved?

Mukuyenera kuwawunikira anthu kuti njira zoyezetera anazi zikuyenera kutithandiza ife tomwe

CG- You need to explain to people that this method is here for us.

6. What should be the role of male partners in the implementation of these approaches? (Probe: How should male partners be encouraged to take active role in these approaches?)

Azibambo akuyenera kuzayezetsa komanso inu a chipatala mukuyenera kufika m’midzi ndikuwaziwitsa azibambo za ubwinozi

CG- Men need to also come for the test. You also need to reach out to them at the village.

7. How would your community feel if these approaches were to be implemented in your nearest health facility? (What could be done to encourage community members to participate in these interventions)

Angamve bwino chifukwa choti ndizothandiza ife tomwe mukuyenera inu a chipatala kuwawunikira

CG- They can welcome it because this method is here to help us, and you need to elaborate to them the importance of this.

8. What are some concerns that you and some members in the community might have related to receiving HIV test results of a child?

Nkhawa zimakhalapo chifukwa choti mwana akapezeka nako umakhala wodandawula

CG- The worry comes in because when a child is found positive, as a parent you become sad.

9. Do you have suggestions or ideas for addressing possible community concerns about these HIV testing strategies?

Munthu akuyenera kulimbikitsidwa kuti kukhala ndi ka chirombo simathero azonse koma apitilize kumwa mankhwala ndikukhala ndi thupi la thanzi

CG- They need to be encouraged that being positive is not the end, they can take medicine and live a healthy life.

B. Perceptions about time to receive test results

10. From the time that your child is tested, how long would you be patient enough to know results from the blood tests? (Same day, after three, after three months?)

Tsiku Lomwelo □

Patatha masiku □

Miyezi iwiri kapena itatu □

Fotokozani zifukwa zomwe mwasankhira Yankho limeneli

Chifukwa ndikufuna kudziwa kuti mwana wanga ali bwanji ndikumuthandiza mwachangu

CG- Because I need to know the health status of my child and help him promptly

11. If your child is tested for HIV, how long would you want to wait before you are told that results from the tests are HIV positive? (same day, after three, after three months?)Explain why you would prefer your chosen answer.

Tsiku Lomwelo □

Patatha masiku □

Miyezi iwiri kapena itatu □

Fotokozani zifukwa zomwe mwasankhira Yankho limeneli

Basi Tikungoyenera kumva kuti mwana ali bwanji

CG- I just need to know how my child is

12. If your child test for HIV, how long would you want to wait before you are told that results from the test are HIV negative? (Same day, after three, after three months?)Explain why you would prefer your chosen answer.

Tsiku Lomwelo □

Patatha masiku □

Miyezi iwiri kapena itatu □

Fotokozani zifukwa zomwe mwasankhira Yankho limeneli

C.Acceptability and decision making

13. What information would you want to be given to make an informed decision to accept that your child should get an HIV test or not? Explain

Mukuyenera kutipatsa uphungu wanga wabwino komanso ngati mwana akudwaladwala tikuyenera kuzamuyezetsa

CG- You need to counsel us and we need to get our child tested if he gets sick regularly

14. How would you want to be approached and given information about these two HIV testing strategies? Explain

Mukuyenera kutifikira m’mudzi koma potilalikila kuzera m’ma radio ndi u chipatala

CG- Reaching us in our villages ,radios even hospitals.

D.Potential Social Harms/Concerns etc.

15. Would you encourage other parents/guardians to allow their children to test for HIV using these two approaches? What would be your main concerns and worries towards these approaches?

Yes □ No □

Nkhawa yanga ili potenga magazi kuti magazi amakhala ochuluka ndiye tikumadabwa kuti akumapita kuti

CG- My problem is with the amount of blood taken and what they do with it

16. How would you personally feel is someone from your community learns about HIV test results for your child?

Sindingamve bwino chifukwa aliyense amafuna chinsinsi

CG- I would not feel good because everyone needs their privacy.

17. Do you have any other thoughts you wish to share on this topic?

Ndikuwona kuti njirazi zili bwino chifukwa choti nthawi yonseyi kunalibe njirazi chandikomera chifukwa choti tiziziwa mwansanga ngati mwana ali ndi ka chirombo ndikudziwa m’mene tingamuthandizile

I think this is a very good method because it was not there before, We will be able to know very fast the status of our children and they will be getting the help they need fast.

*The Research Team*
