# Supplementary material for: ‘If I am on ART, my new-born baby should be put on treatment immediately’: Exploring the acceptability, and appropriateness of Cepheid Xpert HIV-1 Qual assay for early infant diagnosis of HIV in Malawi
Source: PLOS Glob Public Health. 2023 Mar 10;3(3):e0001135. doi: 10.1371/journal.pgph.0001135 (PMC10021387; doi:10.1371/journal.pgph.0001135)
Supplement: S1 File — (ZIP) [file pgph.0001135.s004.zip › transcripts/DET 0060.docx]

*A Questionnaire to validate new HIV tests called Cepheid Xpert HIV -1 Quay assay (Cepheid) in your hospital*

DET 0060

1. How would you as a parent/guardian feel if your child was to undergo HIV testing with Cepheid?

Ine Ndingamve bwino chifukwa ndi m’mene dziko likuyendera aliyense akuyenera kudziwa m’mene alili

CG- I would feel good because in the world these days everyone needs to know their status

2. What are your thoughts about these new strategies for testing HIV in children and giving results promptly?

Ndichinthu chabwino chifukwa tiziwa m’mene alili mwana ndiamene tingamuthandidzire

CG- It’s a good thing because we know the child’s status and how the child can be helped

3. How should these approaches be implemented in a hospital? (Probe who should be targeted, why should they be targeted and why?)

Mukuyenera kutiwuza inu a chipatala mukuyenera muyambire ana chifukwa iwo ndi atsogoleri amawa

CG- The hospital personnel are the ones who should tell us and testing should start with kids because they are our future leaders

4. How should issues of privacy of both children and their guardians be maintained?

Mukuyenera inu a chipatala kutisungila chinsinsi

CG- It must be kept private by the hospital

5a.What should be the role of parents/guardians in the implementations of these approaches?

Gawo limene ndingatenge monga ine kholo ndikuyezetsa mwana wanga

CG- As a parent my role is to get my child tested

b. What information should be provided to ensure that guardians understand the procedures involved?

A chipatala mukuyenera kutiwuza ubwino woyezetsa ndikapewedwe kake

CG- We need to be told the importance of the test and the prevention measures

6. What should be the role of male partners in the implementation of these approaches? (Probe: How should male partners be encouraged to take active role in these approaches?)

-azibambo ndiwokanika

CG- Men are hard to deal with

-Mukuyenera Kuwalimbikitsa a zibambo kuzayedzetsa kuzela nchipatala

CG- You need to encourage men to get tested

7. How would your community feel if these approaches were to be implemented in your nearest health facility? (What could be done to encourage community members to participate in these interventions)

-Atha kukhala osangalala

-They would be happy

-Titha kuwafotokozera za ubwino oyezetsa

- we can tell them the importance of testing

8. What are some concerns that you and some members in the community might have related to receiving HIV test results of a child?

Kwa ine sindikhala ndi Nkhawa koma ndimakhala osangala chifukwa ndiziwa momusamalira mwana

CG- I would only be grateful because I would know how to take care of my child

9. Do you have suggestions or ideas for addressing possible community concerns about these HIV testing strategies?

Kwa Munthu amene amakhala ndi Nkhawa tikuyenera Kuwalimbikitsa kuti simathero amoyo chifukwa choti mankhwala amene timapasidwa amatiwonjezera moyo

CG- They need to be encouraged that being found with the virus is not the end of the world sicne the ART adds more days to life

B. Perceptions about time to receive test results

10. From the time that your child is tested, how long would you be patient enough to know results from the blood tests? (Same day, after three, after three months?)

Tsiku Lomwelo □

Patatha masiku □

Miyezi iwiri kapena itatu □

Fotokozani zifukwa zomwe mwasankhira Yankho limeneli

Chifukwa choti ndikufuna kuziwa momutetedzera mwana

CG- Because I want to know how to take Care of my child

11. If your child is tested for HIV, how long would you want to wait before you are told that results from the tests are HIV positive? (same day, after three, after three months?)Explain why you would prefer your chosen answer.

Tsiku Lomwelo □

Patatha masiku □

Miyezi iwiri kapena itatu □

Fotokozani zifukwa zomwe mwasankhira Yankho limeneli

Ndikufuna kuziwa mwana m’mene alili ngati atapezeka nako ndikhonza kusamala motani

CG- I want to know how my child is and how I can help her

12. If your child test for HIV, how long would you want to wait before you are told that results from the test are HIV negative? (Same day, after three, after three months?)Explain why you would prefer your chosen answer.

Tsiku Lomwelo □

Patatha masiku □

Miyezi iwiri kapena itatu □

Fotokozani zifukwa zomwe mwasankhira Yankho limeneli

Ndichonyadisa kumva ngati mwana alibe matenda ndipo tingathe kuziwa momutetedzera kuti astenge matenda

CG- it is excting to hear when the child is negative and to learn how to protect her

C.Acceptability and decision making

13. What information would you want to be given to make an informed decision to accept that your child should get an HIV test or not? Explain

Mukuyenera kundiwuza inu maganizo osatira kuti ndipange chisankho choyezetsa mwana wanga

CG- You need counsel me what I need to know so that I can make a decision to get my child tested

14. How would you want to be approached and given information about these two HIV testing strategies? Explain

Ine ndingafune mundifikire ku chipatala konkuno Sinanga ndi konkuno

I would prefer if you reach out to me when I come to the hospital

D.Potential Social Harms/Concerns etc.

15. Would you encourage other parents/guardians to allow their children to test for HIV using these two approaches? What would be your main concerns and worries towards these approaches?

Yes □ No □

Sindingakhale ndi Nkhawa chifukwa choti mukutenga kwanu magazi mukufuna kunditetedzera mwana wanga

CG- I would not be worried because you are drawing the blood with intentionsof helping my child

16. How would you personally feel is someone from your community learns about HIV test results for your child?

I would not feel bad because there would be no time for worries but thinking about the future

17. Do you have any other thoughts you wish to share on this topic?

Ine ndilibe ganizo kapena dandawulo lili lonse pa nkhaniyi

CG- I have no concerns

*The Research Team*
