# Supplementary material for: ‘If I am on ART, my new-born baby should be put on treatment immediately’: Exploring the acceptability, and appropriateness of Cepheid Xpert HIV-1 Qual assay for early infant diagnosis of HIV in Malawi
Source: PLOS Glob Public Health. 2023 Mar 10;3(3):e0001135. doi: 10.1371/journal.pgph.0001135 (PMC10021387; doi:10.1371/journal.pgph.0001135)
Supplement: S1 File — (ZIP) [file pgph.0001135.s004.zip › transcripts/DET 045.docx]

*A Questionnaire to validate new HIV tests called Cepheid Xpert HIV -1 Quay assay (Cepheid) in your hospital*

DET 0045

1. How would you as a parent/guardian feel if your child was to undergo HIV testing with Cepheid?

CG-Atha kumva bwino chifukwa aziwa amene angatumizire akava zotsatilazo

2. What are your thoughts about these Cepheid Xpert HIV -1 Quay assay using whole blood (Cepheid) for testing HIV in children and giving results promptly?

CG-Alibepo maganizo aliwonse.

3. How should this approach of **Cepheid Xpert HIV -1 Quay assay using whole blood (Cepheid)**  be implemented in a hospital? (Probe who should be targeted, why should they be targeted and why?)

CG-Aziuzidwa akabwera kuchipatala kuno za Cepheid

CG-tiyambile ana chifukwa ndondomeko yakuyeza ana pakadali pano palibe koma ya akulu ilipo

4. How should issues of privacy of both children and their guardians be maintained?

CG-Chinsinsi chikuyenela kukhala ndi kholo la mwana

5a.What should be the role of parents/guardians in the implementation of this approach of **Cepheid Xpert HIV -1 Quay assay using whole blood (Cepheid)**?

CG-Gawo lomwe angatengepo ndikubwelesa mwana kuti azayezedwe

b.What information should be provided to ensure that guardians understand the procedures involved?

CG-M’mene wachimvera chifukwa ena olo auzidwe uphungu saugwilisaso ntchito ayi

6. What should be the role of male partners in the implementation of this approach of **Cepheid Xpert HIV -1 Quay assay using whole blood (Cepheid)**? (Probe: How should male partners be encouraged to take active role in these approaches?)

CG-Azimayi akuyenela kuzawauza azimuna awo zaubwio wanjirazi

7. How would your community feel if **Cepheid Xpert HIV -1 Quay assay using whole blood (Cepheid)**

were to be implemented in your nearest health facility? (What could be done to encourage community members to participate in these interventions?)

CG-Ena atha kuchimva koma ena sangachimve

CG--pakuyenera kuwauza afumu kuti apange msonkhano wokhuzana ndi njirazi

8. What are some concerns that you and some members in the community might have related to receiving HIV test results of a child?

CG-Nkhawa imakhalapo kuti chifukwa suziwa mene mwana alili nthupi

9. Do you have suggestions or ideas for addressing possible community concerns about these HIV testing strategies?

CG-Madakotala apelike malangiso athesa nkhawa akamadikira Zotsatira

B. Perceptions about time to receive test results

10. From the time that your child is tested, how long would you be patient enough to know results from the blood tests? (Same day, after three, after three months?)

Tsiku Lomwelo □√

Patatha masiku □

Miyezi iwiri kapena itatu □

Fotokozani zifukwa zomwe mwasankhira Yankho limeneli

CG-Ndiziwe kuti mwana wanga ali bwanji ndikudziwa momutetedzera

11. If your child is tested for HIV, how long would you want to wait before you are told that results from the tests are HIV positive? (same day, after three, after three months?)Explain why you would prefer your chosen answer.

Tsiku Lomwelo □√

Patatha masiku □

Miyezi iwiri kapena itatu □

Fotokozani zifukwa zomwe mwasankhira Yankho limeneli

CG-Powona ulendo wamayendedwe anga Ndikuwona kuti bola Patatha masiku

12. If your child test for HIV, how long would you want to wait before you are told that results from the test are HIV negative? (Same day, after three, after three months?)Explain why you would prefer your chosen answer.

Tsiku Lomwelo □√

Patatha masiku □

Miyezi iwiri kapena itatu □

Fotokozani zifukwa zomwe mwasankhira Yankho limeneli

CG-Pamenepa ndilibe ganizo lililonse

C.Acceptability and decision making

13. What information would you want to be given to make an informed decision to accept that your child should get an HIV test or not? Explain

CG-Ndikuyenera kupatsidwa uphungu woyenera posawona nkhope ndikutilangiza bwino.

14. How would you want to be approached and given information about these two HIV testing strategies? Explain

CG-Ine ndasankha kundifikila kuno ku chipatala kundilangiza za Cepheid

D.Potential Social Harms/Concerns etc.

15. Would you encourage other parents/guardians to allow their children to test for HIV using **Cepheid Xpert HIV -1 Quay assay using whole blood (Cepheid)**?

Yes □ No □

What would be your main concerns and worries towards this approach?

CG-Nkhawa yanga ili poti mwana atatengera matenda ndikhonza kudandawula ndithu

16. How would you personally feel is someone from your community learns about HIV test results for your child?

CG-Ndikuwona kuti zingavute chifukwa anthufe ndiwosiyana mitima chifukwa Akhonza kumalalika m’mudzi

17. Do you have any other thoughts you wish to share on this topic?

Ine ndilibe Nkhawa kapena ganizo lililonse (PARTICIPANT HAS NO DOUBT NOR OTHER THOUGHTS)

*The Research Team*
