# Supplementary material for: ‘If I am on ART, my new-born baby should be put on treatment immediately’: Exploring the acceptability, and appropriateness of Cepheid Xpert HIV-1 Qual assay for early infant diagnosis of HIV in Malawi
Source: PLOS Glob Public Health. 2023 Mar 10;3(3):e0001135. doi: 10.1371/journal.pgph.0001135 (PMC10021387; doi:10.1371/journal.pgph.0001135)
Supplement: S1 File — (ZIP) [file pgph.0001135.s004.zip › transcripts/DET006.docx]

**DET006_CG_F_24.7.18**

1. **Malingana ndi mmene tafotokozera za kayezedwe ka Cepheid Xpert HIV -1 Quay assay using whole blood (Cepheid), mwana ayenera kutengedwa magazi pachara kapena pa nsempha, inu monga kholo mungamve bwanji kuti mwana wanu ayezedwe magazi kuzera njira zimezi?**

- **CG-** NJira zimenezi zikuwoneka kuti ndizothandiza.

1. **Kwainu monga kholo la mwana wa chichepere, maganizo anu ndi otani pokhuzana ndi mayezedwe a magazi kuti tidziwe kuti mwana ali ndi HIV kapena ayi malingana ndi mmene tafotokozera za kayezedwe ka Cepheid Xpert HIV -1 Quay assay using whole blood (Cepheid) malingana ndi nthawi yimene zosatira zimatuluka?**

- **CG-**  Ineyo ndiwochilandira kwambiri chifukwa njira zimenezi zitithandiza kuziwa msanga mmene mmatupi a mwana wathu aliri.

1. **Kodi njira zimenezi tingazikhazikise bwanji mu zipatala? (tatiwuzani, tiyambe ndi gulu liti la anthu ndipo nchifukwa chani mukuganiza kuti tiyambe ndi gulu limeneli chifukwa chain?**

- **CG-**  Tikuyenera kufikila mafumu ndikuwafotokozera kuti awuze anthu a m’mudzi za mayezedwe amenewa, muyambire ana chifukwa njira zimenezi kunalibe ndi kale lonse.

1. **Kodi tingapange bwanji kuti kuyezesa magazi kwa ana ndi makolo awo kapena anthu owayang’ira zikhale za chinsinsi?**

- **CG-** Popeleka uphungu wabwino kwa owayang’anira komanso kuwaziwitsa kuti ndi ufulu wawo kusunga chinsinsi.

1. **Kodi makolo angatengepo gawo lanji kuti njira zoyezesera magazi za Cepheid Xpert HIV -1 Quay assay using whole blood (Cepheid) zikhazikisidwe mu chipatala chathu chino cha Mulanje?**

- **CG-** Makolo akuyenera kuchilandira ndikumvesesa pamayezedwe amenewa.

b). **Kodi makolo awuzidwe zotani ndi uphungu wotani kuti amvesese za njira zoyezesera magazi za Cepheid Xpert HIV -1 Quay assay using whole blood (Cepheid)?**

- **CG-** Tikunera kuwafotokozera kuti uphungu umenewu ndiwonera kwa ana athu.

1. **Kodi azibambo angatengepo gawo lanji kuti njira zoyezesera magazi za Cepheid Xpert HIV -1 Quay assay using whole blood (Cepheid) zikhazikisidwe mu chipatala chathu chino cha Mulanje? Tingawalimbikise bwanji azibambo kuti azitenga nawo gawo mukuyezedwa magazi mu njira za Cepheid Xpert HIV -1 Quay assay using whole blood (Cepheid)?**

- **CG-**  Ndilibe ganizo lililonse.

1. **Kodi anthu a mmudzi mwanu angamve bwanji njira zoyezesera magazi za Cepheid Xpert HIV -1 Quay assay using whole blood (Cepheid) zitakhazikisidwa pa chipatala chanu chaching’ono mmudzi mwanu. Tingatani kuti anthu a mmudzi muno alimbikisidwe kutenga nawo mbali mu njira zoyezetsera magazi za Cepheid Xpert HIV -1 Quay assay using whole blood (Cepheid)?**

- **CG-** Zingakhale zonyaditsa ndi zokoma chifukwa mayendedwe angakhale osavuta komanso tingakhale olimbikitsana m’mudzi pakayezedwe kasopanoka.

1. **Kodi inu ndi anthu ena mma midzi mu mumakhala ndi nkhwa zanji zokhuzana ndi kulandila zosatira za magazi mwana akayezedwa kuti tiziwe kuti mwana ali ndi HIV kapena ayi?**

- **CG-**  Aliyense pamene akuyezedwa amakhala ndi nkhawa makamaka pamene ukamamvesera zotsatira za magazi ako chifukwa umatha kuganiza kuti sindifa ndikapezeka nako.

1. **Kodi mungakhale ndi njira kapena maganizo a momwe tingathandizire kuchepesa nkhawa zokhuzana ndikulandila zotsatira za magazi mwana wayezedwa kuti tidziwe kuti mwana ali ndi HIV kapena ayi?**

- **CG-**  Kupemphera ndichiyambi chochotsa nkhawa komanso kulimbikitsana.

1. **Kuchokera pa nthawi yomwe mwana wanu wayezedwa magazi kuti tidziwe kuti mwana ali ndi HIV kapena ayi, mungapilile nthawi yayitali bwanji kuti mudziwe zosatira**

- **Tsiku lomwelo**

**Patatha masiku**

**Miyezi iwiri kapena itatu**

**Fotokozani zifukwa zomwe mungasankhile yankho limeneli**

- **CG-**  Chifukwa zizatithandiza kuziwa m’mene tingamusamalilire mwana wanga ndikumuteteza moyenera

1. **Mwana wanu atayezedwa magazi, mungafune kudikila nthawi yayitali bwanji kuti mudziwe kuti mwana ali ndi HIV yomwe yimayambitsa matenda a AIDS?**

- **TSiku lomwelo**

**Patatha masiku**

**Miyezi iwiri kapena itatu**

**Fotokozani zifukwa zimene mwasankhila yankho limenelo**

- **CG-** Nthawi ina iliyonse imene a dokotala anenere kuti tidikire.

1. **Mwana wanu atayezedwa magazi mungafune kudikila nthaawi yayitali bwanji kuti muziwe kuti mwana alibe HIV yomwe imayambitsa matenda a AIDS**

- **Tsiku lomwelo**

**Patatha masiku**

**Miyezi iwiri kapena itatu**

**Fotokozani zifukwa zomwe mungasankhile yankho limenelo**

- **CG-**  Chifukwa choti ndidziwa nthawi yoyenera komanso ngati mwana wanga akuyenera kumwa mankhwala kapena ayi.

1. **kodi mungafune muwuzidwe zotani ndi uphungu otani kuti inu mupange chisankho choti mwana wanu ayezedwe magazi kuti mudziwe kuti mwana ali ndi HIV yomwe imayambitsa matenda a AIDS kapena ayi? Fotokozani bwino lomwe.**

- **CG-**  ife tikuyenera kulandila uphungu monga makolo, ndipo otofikila potiwuza uphunguwu akuyenera kutiwuza mwa ndondomeko.

1. **Mungafune kuti tikufikileni mu njira yotani kuti tikuwuzeni zimezi ndikukupasani uphungu umenewu wa njira zoyezesera magazi za Cepheid Xpert HIV -1 Quay assay using whole blood (Cepheid)?**

- **CG-**  Potiphunzitsa kuzera ma wailesi komanso kumata zithunzi mzipatala zophunzitsa njirazi.

1. **Kodi mungathe kuwalimbikisa makolo anzanu kapena owasamalira ana kuti alore ana Awo ayezedwwe magazi kuti aziwe ngati ali ndi HIV yoyambitsa matenda a AIDS kugwilitsa ntchito Cepheid Xpert HIV -1 Quay assay using whole blood (Cepheid)?**

- **CG-**  Eya

**15b) Nkhawa zanu zingakhale zotani ndi mayezedwe amenewa a Cepheid Xpert HIV -1 Quay assay using whole blood (Cepheid)?**

- **CG-** Ine sindikuwonapi nkhawa iliyonse chifukwa njirayi itichitira ubwino kwabasi.

1. **Kodi mungamve bwanji ngati munthu wina wa mmudzi mwanu ataziwa zotsatira za magazi a mwana wanu atayezedwa kufufuza ngati ali ndi HIV kapena ayi?**

- **CG-** Tikungoyenera kudziwa ndi ufulu wathu kuteteza moyo wa mwana wathu chifukwa sikoyenera kulola munthu wina kudziwa zosatila za mwana wathu, monga ine kholo ndikuyenera kutenga gawo ndikuteteza mwana wanga.

1. **Kodi muli ndi maganizo kapena nkhawa zina zomwe mungafune kutidziwisa pa nkhani imeneyi**

- **CG-**  Ine nkhawa yanga ndiyapotenga magazi basi chifukwa ndimadabwa kuti magazi a mwana wanga akupita kuti.

*The Research Team*
