# Supplementary material for: ‘If I am on ART, my new-born baby should be put on treatment immediately’: Exploring the acceptability, and appropriateness of Cepheid Xpert HIV-1 Qual assay for early infant diagnosis of HIV in Malawi
Source: PLOS Glob Public Health. 2023 Mar 10;3(3):e0001135. doi: 10.1371/journal.pgph.0001135 (PMC10021387; doi:10.1371/journal.pgph.0001135)
Supplement: S1 File — (ZIP) [file pgph.0001135.s004.zip › transcripts/DET013.docx]

**DET013_CG_F_26.7.18**

1. **Malingana ndi mmene tafotokozera za kayezedwe ka Cepheid ndi Lamp, mwana ayenera kutengedwa magazi pachara kapena pa nsempha, inu monga kholo mungamve bwanji kuti mwana wanu ayezedwe magazi kuzera njira zimezi?**

- **CG-** Ndingamve bwino chifukwa ndizimene ndimayembekezera ndicholinga choti mwana athandizidwe mwachangu ngati alinako kapena ayi.

1. **Kwainu monga kholo la mwana wa chichepere, maganizo anu ndi otani pokhuzana ndi mayezedwe a magazi kuti tidziwe kuti mwana ali ndi HIV kapena ayi malingana ndi mmene tafotokozera za kayezedwe ka Cepheid ndi Lamp malingana ndi nthawi yimene zosatira zimatuluka ?**

- **CG-**  Ndili onyadira chifukwa nthawi yonseyi njira ngati imeneyi kunalibe ndiye m’mene abwelesa izi ndakondwera zedi.

1. **Kodi njira zimenezi tingazikhazikise bwanji mu zipatala? (tatiwuzani, tiyambe ndi gulu liti la anthu ndipo nchifukwa chani mukuganiza kuti tiyambe ndi gulu limeneli chifukwa chain?**

- **CG-** Pamenepo ndilibe ganizo, Muyambire ndi ana ndi akulu omwe.

1. **Kodi tingapange bwanji kuti kuyezesa magazi kwa ana ndi makolo awo kapena anthu owayang’ira zikhale za chinsinsi?**

- **CG-** Zimatengera munthu mwini wake kusunga chinsinsi.

1. **Kodi makolo angatengepo gawo lanji kuti njira zoyezesera magazi za Cepheid ndi Lamp zikhazikisidwe mu chipatala chathu chino cha Mulanje?**

- **CG-**  Zitengera munthu maganizo ako kuti ine ndikugwilizana nazo kapena ayi.Ndilibe ganizo lililonse.

b). **Kodi makolo awuzidwe zotani ndi uphungu wotani kuti amvesese za njira zoyezesera magazi za Cepheid ndi Lamp?**

- **CG-** Ndilibe ganizo lililonse.

1. **Kodi azibambo angatengepo gawo lanji kuti njira zoyezesera magazi za Cepheid ndi Lamp zikhazikisidwe mu chipatala chathu chino cha Mulanje? Tingawalimbikise bwanji azibambo kuti azitenga nawo gawo mukuyezedwa magazi mu njira za Cepheid ndi Lamp?**

- **CG-**  Ndingawuze kuti kwabwera njira zatsopano zomwe zikuthandiza ana kuyezedwa magazi ndikuziwa tsiku lomwe kungowatsimikizira kuti njirazi ndi zabwino ndizonyadisa.

1. **Kodi anthu a mmudzi mwanu angamve bwanji njira zoyezesera magazi za Cepheid ndi Lamp zitakhazikisidwa pa chipatala chanu chaching’ono mmudzi mwanu. Tingatani kuti anthu a mmudzi muno alimbikisidwe kutenga nawo mbali mu njira zoyezetsera magazi za Cepheid ndi Lamp?**

- **CG-** Atha kukhala onyadira chifukwa kayezedwe aka ka ana tinalibe mbuyomu.

1. **Kodi inu ndi anthu ena mma midzi mu mumakhala ndi nkhwa zanji zokhuzana ndi kulandila zosatira za magazi mwana akayezedwa kuti tiziwe kuti mwana ali ndi HIV kapena ayi?**

- **CG-** Ndilibe ganizo.

1. **Kodi mungakhale ndi njira kapena maganizo a momwe tingathandizire kuchepesa nkhawa zokhuzana ndikulandila zotsatira za magazi mwana wayezedwa kuti tidziwe kuti mwana ali ndi HIV kapena ayi?**

- **CG-**  Kwa ine ndingochivomereza ndipo nditapezeka ndilibe ndikhoza kuyamika ambuye.

1. **Kuchokera pa nthawi yomwe mwana wanu wayezedwa magazi kuti tidziwe kuti mwana ali ndi HIV kapena ayi, mungapilile nthawi yayitali bwanji kuti mudziwe zosatira**

**Tsiku lomwelo**

- **Patatha masiku**

**Miyezi iwiri kapena itatu**

**Fotokozani zifukwa zomwe mungasankhile yankho limeneli**

- **CG-**  Chifukwa ndingafune kuziwa ngati mwana wanga ali ndi kachilombo kapena ayi.

1. **Mwana wanu atayezedwa magazi, mungafune kudikila nthawi yayitali bwanji kuti mudziwe kuti mwana ali ndi HIV yomwe yimayambitsa matenda a AIDS?**

- **TSiku lomwelo**

**Patatha masiku**

**Miyezi iwiri kapena itatu**

**Fotokozani zifukwa zimene mwasankhila yankho limenelo**

- **CG-**  Ndasankha tsiku limodzi chifukwa ndili ndi mwana wina ndimwina a khonza kumandivuta.

1. **Mwana wanu atayezedwa magazi mungafune kudikila nthaawi yayitali bwanji kuti muziwe kuti mwana alibe HIV yomwe imayambitsa matenda a AIDS**

- **Tsiku lomwelo**

**Patatha masiku**

**Miyezi iwiri kapena itatu**

**Fotokozani zifukwa zomwe mungasankhile yankho limenelo**

- **CG-** Yankho ndilibe.

1. **kodi mungafune muwuzidwe zotani ndi uphungu otani kuti inu mupange chisankho choti mwana wanu ayezedwe magazi kuti mudziwe kuti mwana ali ndi HIV yomwe imayambitsa matenda a AIDS kapena ayi? Fotokozani bwino lomwe.**

- **CG-** Tiwuzidwe kuti pano tikutenga magazi ndipo zitengera inu nomwe kuti mwana wanu athandizidwe.

1. **Mungafune kuti tikufikileni mu njira yotani kuti tikuwuzeni zimezi ndikukupasani uphungu umenewu wa njira zoyezesera magazi za Cepheid ndi Lamp?**

- **CG-** Mutifikire munjira iliyonse kaya kundikira kunyumba.

1. **Kodi mungathe kuwalimbikisa makolo anzanu kapena owasamalira ana kuti alore ana Awo ayezedwwe magazi kuti aziwe ngati ali ndi HIV yoyambitsa matenda a AIDS kugwilitsa ntchito Cepheid ndi Lamp?**

- **CG-**  Eya

**15b) Nkhawa zanu zingakhale zotani ndi mayezedwe amenewa a** **Cepheid Xpert HIV -1 Quay assay using whole blood (Cepheid)?**

- **CG-** Ndilibe nkhawa.

1. **Kodi mungamve bwanji ngati munthu wina wa mmudzi mwanu ataziwa zotsatira za magazi a mwana wanu atayezedwa kufufuza ngati ali ndi HIV kapena ayi?**

- **CG-** Ndikhonza kumva bwino.

1. **Kodi muli ndi maganizo kapena nkhawa zina zomwe mungafune kutidziwisa pa nkhani imeneyi**

- **CG-**  Ndilibe nkhawa kapena ganizo.

*The Research Team*
