# Supplementary material for: ‘If I am on ART, my new-born baby should be put on treatment immediately’: Exploring the acceptability, and appropriateness of Cepheid Xpert HIV-1 Qual assay for early infant diagnosis of HIV in Malawi
Source: PLOS Glob Public Health. 2023 Mar 10;3(3):e0001135. doi: 10.1371/journal.pgph.0001135 (PMC10021387; doi:10.1371/journal.pgph.0001135)
Supplement: S1 File — (ZIP) [file pgph.0001135.s004.zip › transcripts/DET022.docx]

**DET022_CG_F_27.7.18**

1. **Malingana ndi mmene tafotokozera za kayezedwe ka Cepheid, mwana ayenera kutengedwa magazi pachara kapena pa nsempha, inu monga kholo mungamve bwanji kuti mwana wanu ayezedwe magazi kuzera njira zimezi?**

- **CG** Kumva bwino chifukwa akufuna kumva za nthupi komaso kutsatila malangizo omwe achipatala anganene
- **CG-** I would feel good because I would want to know the results

1. **Kwainu monga kholo la mwana wa chichepere, maganizo anu ndi otani pokhuzana ndi mayezedwe a magazi kuti tidziwe kuti mwana ali ndi HIV kapena ayi malingana ndi mmene tafotokozera za kayezedwe ka Cepheid kuti zosatira zimatuluka kwa minitsi 92?**

- **CG-**  akuona kuti njilazi zilibwino chifukwa aziwa zotsatila mwachangu
- **CG-** it is a good method because you hear the results in short time

1. **Kodi njira zimenezi tingazikhazikise bwanji mu zipatala? (tatiwuzani, tiyambe ndi gulu liti la anthu ndipo nchifukwa chani mukuganiza kuti tiyambe ndi gulu limeneli chifukwa chain?**

- **CG-** Kukhazikitsa kwake ndikosavuta chifukwa kwa amene amvetsetsa ngati achitilamu atha kuzitsatila makamaka akuti zimbile ndi gulu la ana chifukwa pakali pano zimavuta kuti aziwe zotsatila zawo
- **CG-** Start with the children because it is hard to get them tested

1. **Kodi tingapange bwanji kuti kuyezesa magazi kwa ana ndi makolo awo kapena anthu owayang’ira zikhale za chinsinsi?**

CG- Kupita nawo ku chipinda ndikwauzila komko zotsatila zikhale kwa mwini wakeyo kuti auze anthu kapena ayi

CG- Telling them the results in a closed room so that it should be up to the owner to tell people.

1. **Kodi makolo angatengepo gawo lanji kuti njira zoyezesera magazi za Cepheid zikhazikisidwe mu chipatala chathu chino cha Mulanje?**

- **CG-** kholo lokonda mwana wake likuyenela kuchinvetsa zokhudzana ndi mayezedwe a Cepheid komaso atha kuwauza anzawo zabwino wa njilazi
- **CG-** For every parent that loves their children it is easy to understand this method and for them to tell other people

b). **Kodi makolo awuzidwe zotani ndi uphungu wotani kuti amvesese za njira zoyezesera magazi za Cepheid?**

- **CG-** kuwauza mene angasamalile mwanayo chifukwa akaziwa status ya mwana wawo aziwa tsogolo lake komaso mene angamusamalilire
- **CG-** Advise them how they can take care of their child after they get the results

1. **Kodi azibambo angatengepo gawo lanji kuti njira zoyezesera magazi za Cepheid zikhazikisidwe mu chipatala chathu chino cha Mulanje? Tingawalimbikise bwanji azibambo kuti azitenga nawo gawo mukuyezedwa magazi mu njira za Cepheid?**

Mene azimayi akufotokozeledwa ndi momwemoso azibambo akuyenela kuuzidwila chifukwa mwana ndiwa onse siwa mayi ekha ayi

CG- The same way this issue is being handled with women because the child is for them both

1. **Kodi anthu a mmudzi mwanu angamve bwanji njira zoyezesera magazi za Cepheid zitakhazikisidwa pa chipatala chanu chaching’ono mmudzi mwanu. Tingatani kuti anthu a mmudzi muno alimbikisidwe kutenga nawo mbali mu njira zoyezetsera magazi za Cepheid ndi?**

- **CG-** atha kuchilandiLA bwino chifukwa zawafikila pafupi komaso zitha kundathindaza kuti aziwe mene ana wo alili komaso alimbikitsidwe polandila uphungu waubwino wa njilazi
- **CG-** They would welcome it because everyone wants to know the status of their child.

1. **Kodi inu ndi anthu ena mma midzi mu mumakhala ndi nkhwa zanji zokhuzana ndi kulandila zosatira za magazi mwana akayezedwa kuti tiziwe kuti mwana ali ndi HIV kapena ayi?**

- **CG- Palibe nkhawa chiufkwa ngati pali chithandizo alandila mwachangu chifukwa olo iwowo sakhala ndi nkhawa akayezedwa**
- **CG-** I do not have any concerns because I know we will be assisted

1. **Kodi mungakhale ndi njira kapena maganizo a momwe tingathandizire kuchepesa nkhawa zokhuzana ndikulandila zotsatira za magazi mwana wayezedwa kuti tidziwe kuti mwana ali ndi HIV kapena ayi?**

**CG-**  Akuyenela kuuzidwa malangizo komaso kuwalimbikitsa kuti mwana akapezeka ndi kachilombo simathelo azonse koma chiyambi cha moyo

**CG-** They need to be counselled that even if the child is positive, it is not the end of the world

1. **Kuchokera pa nthawi yomwe mwana wanu wayezedwa magazi kuti tidziwe kuti mwana ali ndi HIV kapena ayi, mungapilile nthawi yayitali bwanji kuti mudziwe zosatira**

**Tsiku lomwelo**

**Patatha masiku**

**Miyezi iwiri kapena itatu**

**Fotokozani zifukwa zomwe mungasankhile yankho limeneli**

- **CG-** chifukwa akufuna mwana alandile chithandizo pompo ndikutiso aziwe mwachangu kuti nthupi mwake mene mulili kusiyana ndikudikila nthawi yaitali
- **CG-** Because I need to know how my child is and how to she can get assisted

1. **Mwana wanu atayezedwa magazi, mungafune kudikila nthawi yayitali bwanji kuti mudziwe kuti mwana ali ndi HIV yomwe yimayambitsa matenda a AIDS?**

**TSiku lomwelo**

**Patatha masiku**

**Miyezi iwiri kapena itatu**

**Fotokozani zifukwa zimene mwasankhila yankho limenelo**

- **CG-** Chifukwa ngati afuna kuti mwana ayezedwe ndekuti zotsatilaso afuna adziwe tsiku lomwelo ena asanawapotoze maganizo
- **CG-** Because I want to get the results before someone manipulates my mind

1. **Mwana wanu atayezedwa magazi mungafune kudikila nthaawi yayitali bwanji kuti muziwe kuti mwana alibe HIV yomwe imayambitsa matenda a AIDS**

**Tsiku lomwelo**

**Patatha masiku**

**Miyezi iwiri kapena itatu**

**Fotokozani zifukwa zomwe mungasankhile yankho limenelo**

1. **kodi mungafune muwuzidwe zotani ndi uphungu otani kuti inu mupange chisankho choti mwana wanu ayezedwe magazi kuti mudziwe kuti mwana ali ndi HIV yomwe imayambitsa matenda a AIDS kapena ayi? Fotokozani bwino lomwe.**

- **CG-** Awafotokozzele mene mwana atati wapezeka ndikachilombo kuti amusamale bwanji komaso mene angamusamalile ngati alibe kachilombo
- **CG-** How to take care of a child if found positive or negative

1. **Mungafune kuti tikufikileni mu njira yotani kuti tikuwuzeni zimezi ndikukupasani uphungu umenewu wa njira zoyezesera magazi za Cepheid?**

- **CG- Njila zofikila zambiri monga akabwela ku scale chifukwa akuganiza kuti sitingakwanise kufikila aliense**
- **CG-** When we come for antenatal clinic because you can not manage to reach us in our homes

1. **Kodi mungathe kuwalimbikisa makolo anzanu kapena owasamalira ana kuti alore ana Awo ayezedwwe magazi kuti aziwe ngati ali ndi HIV yoyambitsa matenda a AIDS kugwilitsa ntchito Cepheid?**

- **CG-**  Eya
- **CG-** Yes

**15b) Nkhawa zanu zingakhale zotani ndi mayezedwe amenewa a ndi Cepheid?**

**CG-** Palibe nkwawa inailiyonse chifukwa akuona kuti njilazi zabwino kwambiri

**CG-** I have not concern

1. **Kodi mungamve bwanji ngati munthu wina wa mmudzi mwanu ataziwa zotsatira za magazi a mwana wanu atayezedwa kufufuza ngati ali ndi HIV kapena ayi?**

- **CG-** Alibe vuto linalililonse chifukwa matenda masiku ano siomabisaso ayi
- **CG-**no problem because there is no reason to hide these days

1. **Kodi muli ndi maganizo kapena nkhawa zina zomwe mungafune kutidziwisa pa nkhani imeneyi**

- **CG-** Alibepo nkhawa koma kwawo ndikupempha kuti zipitilile kuti enaso afikilidweso
- **CG-** No question, it should just continue so that it can reach others
