# Supplementary material for: ‘If I am on ART, my new-born baby should be put on treatment immediately’: Exploring the acceptability, and appropriateness of Cepheid Xpert HIV-1 Qual assay for early infant diagnosis of HIV in Malawi
Source: PLOS Glob Public Health. 2023 Mar 10;3(3):e0001135. doi: 10.1371/journal.pgph.0001135 (PMC10021387; doi:10.1371/journal.pgph.0001135)
Supplement: S1 File — (ZIP) [file pgph.0001135.s004.zip › transcripts/DET062 CG.docx]

**DET062_CG_F_16_08_18**

1. Why do caregivers have a lot of trust in hospital staff?

**CG-** Iwowo chifukwa ndiwoziwwa chilichonse ndiye ndiyenera kuwakhulupilira.

**CG-** because they know everything so I need to trust them

1. Why is that most caregivers do not have anything to say when asked question?

**CG-**  Palibe chifukwa.

**CG-** No reason

1. Why do mothers think their children should be tested if they themselves are HIV negative?

**CG-**  Amafuna uziwe kuti magazi ake alibwanji chifukwa mwana amasewera ndizinthu zambiri monga maleza ndi zina.

**CG-** They want to know the child’s status because children sometime play with sharp objects

1. Do women understand the role of ART as the preventative measure if partners are HIV positive?

**CG-** Amamvetsetsa ndipo akungoyenera kuziteteza pogonana ndiwokondedwa wawo

**CG-** They understand and they just need to protect themselves during sexual intercourse
