# Supplementary material for: ‘If I am on ART, my new-born baby should be put on treatment immediately’: Exploring the acceptability, and appropriateness of Cepheid Xpert HIV-1 Qual assay for early infant diagnosis of HIV in Malawi
Source: PLOS Glob Public Health. 2023 Mar 10;3(3):e0001135. doi: 10.1371/journal.pgph.0001135 (PMC10021387; doi:10.1371/journal.pgph.0001135)
Supplement: S1 File — (ZIP) [file pgph.0001135.s004.zip › transcripts/DET064 CG.docx]

**DET064_CG_F_16_08_18**

1. Why do caregivers have a lot of trust in hospital staff?

**CG-** Chifukwa choti timaziwa kuti akhonza kutithandiza pamavuto athu.

**CG-** Because they are the ones that can help us with our problems

1. Why is that most caregivers do not have anything to say when asked question?

**CG-** Awo amakhala mantha koma choyankha amakhala alinacho.

**CG-** I usually have something to say but I don’t because of fear

1. Why do mothers think their children should be tested if they themselves are HIV negative?

**CG-**  Mwana ndi mwana atha kutengera matenda njira zosiyanasiyana posewera ndi azinzake.

**CG-** A child might have contracted the virus from friends while playing

1. Do women understand the role of ART as the preventative measure if partners are HIV positive?

**CG-** I don’t have any idea
