# Supplementary material for: ‘If I am on ART, my new-born baby should be put on treatment immediately’: Exploring the acceptability, and appropriateness of Cepheid Xpert HIV-1 Qual assay for early infant diagnosis of HIV in Malawi
Source: PLOS Glob Public Health. 2023 Mar 10;3(3):e0001135. doi: 10.1371/journal.pgph.0001135 (PMC10021387; doi:10.1371/journal.pgph.0001135)
Supplement: S1 File — (ZIP) [file pgph.0001135.s004.zip › transcripts/DET065 CG.docx]

**DET065_CG_F_16_08_18**

1. Why do caregivers have a lot of trust in hospital staff?

**CG-** Malingana ndimmene iwowo akugwilira ntchito ine ndimakhala ndi chikhulupiliro.

**CG-** I trust them depending on how they are perfoming their job

1. Why is that most caregivers do not have anything to say when asked question?

**CG-** Ena amakhala ndi amanyazi ena osamasuka chifukwa cha m’mene mutnhu alili.

**CG-** Some are just ashamed because of their status so they do know want to say anything

1. Why do mothers think their children should be tested if they themselves are HIV negative?

**CG-**  Mwina munthu umakhala uli pa window period osaziwa ndiye umafunika kuyezetsa mwana komanso mwana atha kutengera posewera.

**CG-** Maybe the mother could be in window period and transmit to the child and the child might also have contracted while playing

1. Do women understand the role of ART as the preventative measure if partners are HIV positive?

**CG-**  I don’t have any idea.
