# Supplementary material for: ‘If I am on ART, my new-born baby should be put on treatment immediately’: Exploring the acceptability, and appropriateness of Cepheid Xpert HIV-1 Qual assay for early infant diagnosis of HIV in Malawi
Source: PLOS Glob Public Health. 2023 Mar 10;3(3):e0001135. doi: 10.1371/journal.pgph.0001135 (PMC10021387; doi:10.1371/journal.pgph.0001135)
Supplement: S1 File — (ZIP) [file pgph.0001135.s004.zip › transcripts/DET066 CG.docx]

**DET066_CG_F_16_08_18**

1. Why do caregivers have a lot of trust in hospital staff?

**CG-**  Chifukwa choti tikabwera kuzalandira chithandizo amatilandira bwinondi kutithandiza m’mene tikufunira.

**CG-** because when we come to the hospital, they welcome us and help us as required

1. Why is that most caregivers do not have anything to say when asked question?

**CG-**  Chifukwa choti amakhala sakuziwa kapena mfuso salimvetsetsa.

**CG-** Because they either have no idea or they didn’t understand the question

1. Why do mothers think their children should be tested if they themselves are HIV negative?

**CG-** Timafuna tiziwe m’mene nthupi mwa mwana alili.

**CG-** We just want to know they status of the child

1. Do women understand the role of ART as the preventative measure if partners are HIV positive?

**CG-** Eya timaziwa ndipo timazitsata

**CG-** Yes we understand and follow it
