# Supplementary material for: ‘If I am on ART, my new-born baby should be put on treatment immediately’: Exploring the acceptability, and appropriateness of Cepheid Xpert HIV-1 Qual assay for early infant diagnosis of HIV in Malawi
Source: PLOS Glob Public Health. 2023 Mar 10;3(3):e0001135. doi: 10.1371/journal.pgph.0001135 (PMC10021387; doi:10.1371/journal.pgph.0001135)
Supplement: S1 File — (ZIP) [file pgph.0001135.s004.zip › transcripts/DET067 CG.docx]

**DET067_CG_F_16_08_18**

1. Why do caregivers have a lot of trust in hospital staff?

**CG-** Chifukwa choti tikadwara akatipatsa mankhwala timachira.

**CG-** Beucase when they give us medication when we are sick, we get better

1. Why is that most caregivers do not have anything to say when asked question?

**CG-**  Chifukwa choti amakhala sakuziwa yankho lake pena amakhala mantha kuti akayankha alakwisa.

**CG-** They either don’t know the answer or they are afraid of getting it wrong

1. Why do mothers think their children should be tested if they themselves are HIV negative?

**CG-**  Chifukwa choti ana amakhala ndi anzawo m’masewero osiyanasiyana ndiye choncho akhonza kusewera zinthu zoti wina wazibaya iwonso kuzibaya potero akhonza kutengera.

**CG-** Because children sometimes would play with sharp objects that were unknowingly used by an infected person

1. Do women understand the role of ART as the preventative measure if partners are HIV positive?

**CG-**  Eya ndimamvetsetsa.

**CG-** Yes I understand
