# Supplementary material for: ‘If I am on ART, my new-born baby should be put on treatment immediately’: Exploring the acceptability, and appropriateness of Cepheid Xpert HIV-1 Qual assay for early infant diagnosis of HIV in Malawi
Source: PLOS Glob Public Health. 2023 Mar 10;3(3):e0001135. doi: 10.1371/journal.pgph.0001135 (PMC10021387; doi:10.1371/journal.pgph.0001135)
Supplement: S1 File — (ZIP) [file pgph.0001135.s004.zip › transcripts/DET068 CG.docx]

**DET068_CG_F_16_08_18**

1. Why do caregivers have a lot of trust in hospital staff?

**CG-** Amakhala ndi faith chifukwa achipatala ndi amene amathandiza munthu akudwara.

**CG-** Because they help us whenever we are sick

1. Why is that most caregivers do not have anything to say when asked question?

**CG-**  Kuganiza kumakhala kosiyana chifukwa ena amakhalaa ndi mantha kuti mwina ndemanga yawo simveka.

**CG-** Because of fear

1. Why do mothers think their children should be tested if they themselves are HIV negative?

**CG-** Ndi mmene ziyenera kukhala mwina kachilombo salinako koma mwana ali nako chifukwa mwana amasewere masewera osiyanasiyana nde atha kukhala nako iwe osaziwa.

**CG-** That is how it is supposed to be because children might have contracted it somewhere without parents knowing

1. Do women understand the role of ART as the preventative measure if partners are HIV positive?

**CG-**  She doesn’t have any idea.
