# Supplementary material for: ‘If I am on ART, my new-born baby should be put on treatment immediately’: Exploring the acceptability, and appropriateness of Cepheid Xpert HIV-1 Qual assay for early infant diagnosis of HIV in Malawi
Source: PLOS Glob Public Health. 2023 Mar 10;3(3):e0001135. doi: 10.1371/journal.pgph.0001135 (PMC10021387; doi:10.1371/journal.pgph.0001135)
Supplement: S1 File — (ZIP) [file pgph.0001135.s004.zip › transcripts/DET069 CG.docx]

**DET069_CG_F_16_08_18**

1. Why do caregivers have a lot of trust in hospital staff?

**CG-** Chifukwa amawathandiza muzambiri nde amakhala ndi chikhulupililo kuti awathandizaso pa vuto lililonse.

**CG-** **CG-** because they help them in so many things hence, they have faith that they can help out with anything.

1. Why is that most caregivers do not have anything to say when asked question?

**CG-** Chifukwa amafuna kuti asatalikise nkhani.

**CG-** Because they do not want to prolong the discussion

1. Why do mothers think their children should be tested if they themselves are HIV negative?

**CG-** Amafuna aaziwe mmene nthupi mwa mwana mulili.

**CG-** They want to know the status of the child

1. Do women understand the role of ART as the preventative measure if partners are HIV positive?

**CG-** She doesn’t have any idea.
