# Supplementary material for: ‘If I am on ART, my new-born baby should be put on treatment immediately’: Exploring the acceptability, and appropriateness of Cepheid Xpert HIV-1 Qual assay for early infant diagnosis of HIV in Malawi
Source: PLOS Glob Public Health. 2023 Mar 10;3(3):e0001135. doi: 10.1371/journal.pgph.0001135 (PMC10021387; doi:10.1371/journal.pgph.0001135)
Supplement: S1 File — (ZIP) [file pgph.0001135.s004.zip › transcripts/DET29.docx]

**DET029_CG_F_30.7.18**

1. **Malingana ndi mmene tafotokozera za kayezedwe ka Cepheid, mwana ayenera kutengedwa magazi pachara kapena pa nsempha, inu monga kholo mungamve bwanji kuti mwana wanu ayezedwe magazi kuzera njira zimezi?**

- **CG-** Angamve bwino chifukwa akupanga tsogolo la mwanayo
- **CG-** I would like it because it is for the future of my child

1. **Kwainu monga kholo la mwana wa chichepere, maganizo anu ndi otani pokhuzana ndi mayezedwe a magazi kuti tidziwe kuti mwana ali ndi HIV kapena ayi malingana ndi mmene tafotokozera za kayezedwe ka Cepheid kuti zosatira zimatuluka kwa minitai 92?**

- **CG-**  Maganizo awo ndi abwino chifukwa zithandiza kuziwa ngati ali ndi matenda kapena ayi
- **CG-** I like this because I want to know the status of my child

1. **Kodi njira zimenezi tingazikhazikise bwanji mu zipatala? (tatiwuzani, tiyambe ndi gulu liti la anthu ndipo nchifukwa chani mukuganiza kuti tiyambe ndi gulu limeneli chifukwa chain?**

- **CG-** Ziwathandiza kudzela kuma ward ya ana ndikufotokoza ubwino wanjilazi komaso tiyambire ana chifukwa mwana atha kutengela munjila zosiyanasiyana mwana ndi mwana
- **CG-** Reach us through the peadatrics wards and start with children

1. **Kodi tingapange bwanji kuti kuyezesa magazi kwa ana ndi makolo awo kapena anthu owayang’ira zikhale za chinsinsi?**

- Chinsinsi chili ndi mwini mwana
- CG- The child should keep this confidential

1. **Kodi makolo angatengepo gawo lanji kuti njira zoyezesera magazi za Cepheid zikhazikisidwe mu chipatala chathu chino cha Mulanje?**

- **CG-**  atenge gawo powayezetsela anawa kudwzela munjila za Cepheid
- **CG-** I will take part by having my children tested

b). **Kodi makolo awuzidwe zotani ndi uphungu wotani kuti amvesese za njira zoyezesera magazi za Cepheid?**

- **CG-** Alandile uphungu owalimbikitsa zaubwino wanjilazi
- **CG-** Be counselled on the importance of this method

1. **Kodi azibambo angatengepo gawo lanji kuti njira zoyezesera magazi za Cepheid zikhazikisidwe mu chipatala chathu chino cha Mulanje? Tingawalimbikise bwanji azibambo kuti azitenga nawo gawo mukuyezedwa magazi mu njira za Cepheid?**

- **CG-**  Akafuna kuyedzesa azibwela kuchipatala kuzaziwa mene alili nthupi
- **CG-** They should come to the hospital for testing

1. **Kodi anthu a mmudzi mwanu angamve bwanji njira zoyezesera magazi za Cepheid zitakhazikisidwa pa chipatala chanu chaching’ono mmudzi mwanu. Tingatani kuti anthu a mmudzi muno alimbikisidwe kutenga nawo mbali mu njira zoyezetsera magazi za Cepheid?**

- CG - Amva bwino chifukwa iwowoso akuyenela kuyezetsa sinanga zafika pafupi kuwauza mmisonkhani kuti akayezedwe
- CG- They would like it and be encouraged to get tested. Reach us through conventions

1. **Kodi inu ndi anthu ena mma midzi mu mumakhala ndi nkhwa zanji zokhuzana ndi kulandila zosatira za magazi mwana akayezedwa kuti tiziwe kuti mwana ali ndi HIV kapena ayi?**

- CG – Nkhawa imakhalapo makamaka kuti zotsatila zibwela zotani
- CG- I am worried of what the results will be

1. **Kodi mungakhale ndi njira kapena maganizo a momwe tingathandizire kuchepesa nkhawa zokhuzana ndikulandila zotsatira za magazi mwana wayezedwa kuti tidziwe kuti mwana ali ndi HIV kapena ayi?**

- CG- Kulimba mtima kuti ngati zotsatila zikuonesa kuti alinako amusamalile mwanayo
- CG- Be strong and if the child is positive learn how to take care of her

1. **Kuchokera pa nthawi yomwe mwana wanu wayezedwa magazi kuti tidziwe kuti mwana ali ndi HIV kapena ayi, mungapilile nthawi yayitali bwanji kuti mudziwe zosatira**

- **Tsiku lomwelo**

**Patatha masiku**

**Miyezi iwiri kapena itatu**

**Fotokozani zifukwa zomwe mungasankhile yankho limeneli**

- **CG-** Chifukwa choti azilimba mtima ndizotsatilazo
- **CG-** Because they will only be comfortable after hearing the results

1. **Mwana wanu atayezedwa magazi, mungafune kudikila nthawi yayitali bwanji kuti mudziwe kuti mwana ali ndi HIV yomwe yimayambitsa matenda a AIDS?**

- **TSiku lomwelo**

**Patatha masiku**

**Miyezi iwiri kapena itatu**

**Fotokozani zifukwa zimene mwasankhila yankho limenelo**

- **CG-** Chifukwa choti sinanga zinthuzi zipita ku Chipinda choyezera(Lab) ndiye zikhonza kutengako nthawi yochepa.
- **CG-** Because the test is taking place in the laboratory I think it cannot take a lot of time

1. **Mwana wanu atayezedwa magazi mungafune kudikila nthaawi yayitali bwanji kuti muziwe kuti mwana alibe HIV yomwe imayambitsa matenda a AIDS**

- **Tsiku lomwelo**

**Patatha masiku**

**Miyezi iwiri kapena itatu**

**Fotokozani zifukwa zomwe mungasankhile yankho limenelo**

**CG-** Kupangila mayendedwe nde ndibwino kuti aziwe tsiku lomwelo.

**CG-** Because I stay far and transportation is difficult

1. **kodi mungafune muwuzidwe zotani ndi uphungu otani kuti inu mupange chisankho choti mwana wanu ayezedwe magazi kuti mudziwe kuti mwana ali ndi HIV yomwe imayambitsa matenda a AIDS kapena ayi? Fotokozani bwino lomwe.**

- **CG-** Kuwalimbikitsa ktui alandila chithandizo akapezeka ndi matenda olo kuwathandiza ndimalangizo ngati alibe kuti akamusamale bwanji mwana
- **CG-** Teach them how they can take care of the child if found positive or negative

1. **Mungafune kuti tikufikileni mu njira yotani kuti tikuwuzeni zimezi ndikukupasani uphungu umenewu wa njira zoyezesera magazi za Cepheid ndi ?**

- **CG-** Njila inailiyonse yomwe achipatala akhonza.
- **CG-** How ever the hospital is comfortable with

1. **Kodi mungathe kuwalimbikisa makolo anzanu kapena owasamalira ana kuti alore ana Awo ayezedwwe magazi kuti aziwe ngati ali ndi HIV yoyambitsa matenda a AIDS kugwilitsa ntchito Cepheid ndi ?**

- **CG-**  Eya
- **CG-** Yes

**15b) Nkhawa zanu zingakhale zotani ndi mayezedwe amenewa a ndi Cepheid?**

- **CG-** Nkhawa zibwela kuti potenga pa msempha kuti sapwetekedwa mwanayo nid dotoloyo
- **CG-** I feel like the venous puncture would hurt my child

1. **Kodi mungamve bwanji ngati munthu wina wa mmudzi mwanu ataziwa zotsatira za magazi a mwana wanu atayezedwa kufufuza ngati ali ndi HIV kapena ayi?**

- **CG-** Sangamve bwino kuti munthu winayo akamva azilengeza zamene mwana wawoyo alili
- **CG-** I would not like it because I do not want my childs status to be talked of by everyone

1. **Kodi muli ndi maganizo kapena nkhawa zina zomwe mungafune kutidziwisa pa nkhani imeneyi**

- **CG-** nkhawa ndiyoti sakudziwa mene mwana wawo alili nthupi makamaka zotsatila za mwanayo ndizomwe zikuwapasa nkhawa.
- **CG-** I am only worried because I do not know the status of my child
