# Supplementary material for: ‘If I am on ART, my new-born baby should be put on treatment immediately’: Exploring the acceptability, and appropriateness of Cepheid Xpert HIV-1 Qual assay for early infant diagnosis of HIV in Malawi
Source: PLOS Glob Public Health. 2023 Mar 10;3(3):e0001135. doi: 10.1371/journal.pgph.0001135 (PMC10021387; doi:10.1371/journal.pgph.0001135)
Supplement: S1 File — (ZIP) [file pgph.0001135.s004.zip › transcripts/MORE CAREGIVER_RESPONSES (1).docx]

1. Why do caregivers have a lot of trust in hospital staff?

**DET01CG** because they are helped in many ways so they have faith they will be helped again without any problems

**DET02CG** Because the hospital stuff know everything so they must be trusted.

**DET03CG** They have faith because medical stuff are the ones who help sick people

**DET04CG** because we know they might help us with our problems.

**DET05CG** in accordance to how they work I have faith in them

**DET06CG** because when we come to get help they receive us well and help us depending on what we need.

**DET07CG** because when we get sick they give us medicine which helps us get well.

**DET08CG** They have faith because the hospital is the institution that helps sick people

**DET09CG** Because they are helped in many ways so they have faith that they will be helped with any problem.

**DET10CG** because we know they can help us in time of need.

1. Why is that most caregivers do not have anything to say when asked question?

**DET01CG-** To prevent dragging the story

**DET02CG** no reason

**DET03CG** Thoughts are different because some are afraid that their views won’t be heard

**DET04CG** because of fear but they always have something to say

**DET05CG** Some people are just shy so they can’t open up because that’s the way they are.

**DET06CG** because they don’t know or they haven’t understood the question.

**DET07CG** because sometimes they don’t know the answer and afraid if they answer they will make a mistake

**DET08CG** Thoughts are different and some are afraid their view won’t be heard.

**DET09CG** To make a long story short

**DET10CG** just because of fear but they have replies.

1. Why do mothers think their children should be tested if they themselves are HIV negative?

**DET01CG-** To know the child’s status

**DET02CG** To know how her blood is because children play with things like razors and needles.

**DET03CG** because that’s how it should be since children play with many things so they may contract it.

**DET04CG** a child is a child and they may contract diseases in different ways while playing with their friiends.

**DET05CG** because they might have been on their window period when getting tested do they

**DET06CG** we want to know our child’s status**.**

**DET07CG** because children play different types of games with their friends and they might play something which involves two or more people contacting blood.

**DET08CG** it’s how it is supposed to be because the parent might not have it but the child may take it from playing around

**DET09CG** To know the status of child

**DET10CG** a child might contract the virus in different ways while playing with their friends

1. Do women understand the role of ART as the preventative measure if partners are HIV positive?

**DET01CG-** She doesn’t have any idea.

**DET02CG** They understand and they must protect themselves when making love to their loved ones.

**DET03CG** To her it’s a new experience

**DET04CG** She doesn’t have any idea.

**DET05CG** must get their child tested and the child may get it while playing.

**DET06CG** Yes we know and understand**.**

**DET07CG** yes I understand

**DET08CG** She doesn’t have any idea

**DET09CG** She doesn’t have any idea.

**DET10CG** She doesn’t have any idea.
