# Supplementary material for: ‘If I am on ART, my new-born baby should be put on treatment immediately’: Exploring the acceptability, and appropriateness of Cepheid Xpert HIV-1 Qual assay for early infant diagnosis of HIV in Malawi
Source: PLOS Glob Public Health. 2023 Mar 10;3(3):e0001135. doi: 10.1371/journal.pgph.0001135 (PMC10021387; doi:10.1371/journal.pgph.0001135)
Supplement: S2 File — (ZIP) [file pgph.0001135.s005.zip › Transcipts _Health _workers/ANSWERS for HW _INTERVIEWS_DET 1-5 HW.docx]

1. Why do women have a lot more confidence in hospital staff?

HW1- Because when they come with a problem they are helped

HW2 Because we are medical personnel and they have faith that we will help them

HW3 it depends on how you have welcomed the person

HW4 They are received well and counselled calmly and we keep their secrets.

HW5 - its how they are welcomed

1. Why is it that caregivers especially women do not have anything to say when asked questions?

HW1- An uneducated woman will have difficulties answering

HW2 think because some may have not gone to school and cultures which state that a man is the one who is supposed to make decisions make them unable to answer questions.

HW3 I think it is because they didn’t go to school so it is difficult for them to answer questions

HW4 because some have fear and others are just born like that

HW5 depends on how you have received the person

1. Why is that caregivers hardly explain answerers, their answers are very short? Eg Anxiety about the window period?

HW1- because they don’t understand what window period is and they think they may have it while there is a 50/50 chance

HW2 I think they have fear and concerns when we explain what window period is and they may not understand it properly

HW3 I think it is mostly because of shyness

HW4 people are secretive and shy

HW5 people are born different and some have troubles because of school

1. What is your opinion about testing for HIV among mothers whose partners are HIV positive?

HW1- For a negative woman it is never safe because men are difficult every time

HW2 I think they are not safe especially to men who resist using condoms and since a woman is a woman and they might not say no and say yes to everything.

HW3 - We might not be sure if they are negative and if they continue being negative we need to advise them to how they can protect themselves.

HW4 They must be tested regularly to get protected

HW5 There you would want to know about the mother because they have high risk of getting the virus because of their partner who is HIV positive
