# Supplementary material for: ‘If I am on ART, my new-born baby should be put on treatment immediately’: Exploring the acceptability, and appropriateness of Cepheid Xpert HIV-1 Qual assay for early infant diagnosis of HIV in Malawi
Source: PLOS Glob Public Health. 2023 Mar 10;3(3):e0001135. doi: 10.1371/journal.pgph.0001135 (PMC10021387; doi:10.1371/journal.pgph.0001135)
Supplement: S2 File — (ZIP) [file pgph.0001135.s005.zip › Transcipts _Health _workers/DET001 HP.docx]

**DET001_HP_16_08_18**

As a healthy professional how do you feel

1. As you deliver this service of Cepheid Xpert HIV -1 Quay assay using whole blood (Cepheid) which involves taking blood.

**HP-** I feel good because despite that we using this method it seems this method is fast and it will help us to save lives in time.

1. As you interact with a care giver where you are taking blood.

**HP-**  It depends ndi m’mene wamufikilira munthu and a kachimvetsetsa bwinobwino.

**HP-** it depends on how you have interacted with the person and how they have understood it.

1. If this way of HIV testing using whole blood is scaled above, do you feel other healthy workers will be interested in this method?

**HP-** Kutenga whole blood kwa ifeyo tinazolowera kuti timatenga pa nthawi yomwe tikufuna kupanga full blood count ndiye ndikuwona kuti zivuta anthu kuti achilandire mwachangu.

**HP-** We are used that we only draw whole blood when conducting full blood count so I think it would be hard for them to accept it.

1. Will it add any extra demand on the healthy services?

**HP-** We will need more teams and more equipment

1. Do you feel you need a lot of time?

**HP-** Yes we need a lot of items of orientation, chifukwa kwa munthu wa nyuwani akuyenera aphunzire bwinobwino.

**HP-** Yes we need a lot of time for orientation because a new person must appropriately learn everything.

1. Are the procedures involved easy to follow?

**HP-** Not easy to follow as such because you need to focus if you don’t focus you can give wrong results.

1. As you deliver this service, what is the general impression of parents and care givers as their children are having blood taken?

**HP-** There expecting kuti azatenga ma results tsiku lomwero rather kuzatengaso in 5days time.

**HP-** They are expecting to get their results on the same day or in 5days time

1. EID results using DBS and PCR turn around time of results is 2-3 months, do you think the ministry of healthy would be interested in Cepheid whole blood protocol which takes 2hours?

**HP-** It is good and atha kuvomereza chifukwa choti turn around time yatsika imene imatitengera nthawi yaitali kuziwa pano aziziwa in few hours.

**HP-** They would like it because of the reduced turn-around time.

1. Do you think the government can afford HIV testing with Cepheid ?

**HP-** Ndi m’mene ndalama yavutilamu ndikuwona ngati zikhoza kuvutirapo kuti agule zipangizo.

**HP-** With the economic problems we have I think it would be hard for them to buy the equipment

1. Can Cepheid whole blood protocol be scaled up?

**HP-**  It can be scaled up kuti anthu apite pa training.

**HP-** it can be scaled up so people can go for training

1. If yes what would be the barriers?

**HP-** Barriers ingakhalepo ngati alibe ma Lab personnel zikhonza kuvutilapo makamaka ma healthy center chifukwa simukhala ma Lab.

**HP-** barriers would be there because if there are no lab personnel it would be difficult especially In the health centers since they are no labs.

1. If yes what would be the selling points?

**HP-** Kumene kuli ma Lab ndizotheka koma kumene kulibe ndizovutilapo.

**HP-** it is possible in places where labs are available.
