# Supplementary material for: ‘If I am on ART, my new-born baby should be put on treatment immediately’: Exploring the acceptability, and appropriateness of Cepheid Xpert HIV-1 Qual assay for early infant diagnosis of HIV in Malawi
Source: PLOS Glob Public Health. 2023 Mar 10;3(3):e0001135. doi: 10.1371/journal.pgph.0001135 (PMC10021387; doi:10.1371/journal.pgph.0001135)
Supplement: S2 File — (ZIP) [file pgph.0001135.s005.zip › Transcipts _Health _workers/DET001 HW.docx]

**DET001_HW_16_08_18**

1. Why do women have a lot more confidence in hospital staff?

**HW-** Chifukwa choti akabwera ndivuto ku chipatala amathandizidwa.

**HW-** Because when they come with a problem they are helped

1. Why is it that caregivers especially women do not have anything to say when asked questions?

**HW-** M’mene ndimawonera kwa nzimayi oti sanapite ku sukulu chimakhala chomuvuta kuyankha mafunso.

**HW-** An uneducated woman will have difficulties answering

1. Why is that caregivers hardly explain answerers, their answers are very short? Eg Anxiety about the window period?

**HW-** Kwambiri pa nkhani ya window period amakhala sakumvetsetsa chifukwa amawona ngati atha kupezeka nako pamene imakhala 50-50 chance.

**HW-** because they don’t understand what window period is and they think they may have it while there is a 50/50 chance

1. What is your opinion about testing for HIV among mothers whose partners are HIV positive?

**HW-** Kwa nzimayi oti alibe sakhala safe chifukwa azibambo amakhala ovuta nthawi zonse.

**HW-** For a negative woman it is never safe because men are difficult every time
