# Supplementary material for: ‘If I am on ART, my new-born baby should be put on treatment immediately’: Exploring the acceptability, and appropriateness of Cepheid Xpert HIV-1 Qual assay for early infant diagnosis of HIV in Malawi
Source: PLOS Glob Public Health. 2023 Mar 10;3(3):e0001135. doi: 10.1371/journal.pgph.0001135 (PMC10021387; doi:10.1371/journal.pgph.0001135)
Supplement: S2 File — (ZIP) [file pgph.0001135.s005.zip › Transcipts _Health _workers/DET002 HP.docx]

**DET002_HP_16_08_18**

As a healthy professional how do you feel

1. As you deliver this service of **Cepheid Xpert HIV -1 Quay assay using whole blood (Cepheid)** which involves taking blood.

**HP-** He feels okay because it is part of his job.

1. As you interact with a care giver where you are taking blood.

**HP-** Other workers will be happy with the process because the results do not take long time to be ready.

1. If this way of HIV testing using whole blood is scaled above, do you feel other healthy workers will be interested in this method?

**HP-** Yes they will be interested because of the quick results.

1. Will it add any extra demand on the healthy services?

**HP-**  It will need a lot of demand because of shortage of nurses.

1. Do you feel you need a lot of time?

**HP-** Not that much time as long as the equipments are available.

1. Are the procedures involved easy to follow?

**HP-** Yes they are easy to follow.

1. As you deliver this service, what is the general impression of parents and care givers as their children are having blood taken?

**HP-** Some are scared to the procedures need to be taught properly.

1. EID results using DBS and PCR turn around time of results is 2-3 months, do you think the ministry of healthy would be interested in Cepheid whole blood protocol which takes 2hours?

**HP-**  The ministry of healthy will be interested because the whole process is very fast which will help.

1. Do you think the government can afford HIV testing with Cepheid ?

**HP-** Yes the government can afford.

1. Can Cepheid whole blood protocol be scaled up?

**HP-** Yes.

1. If yes what would be the barriers?

**HP-** The barriers will be there because of few staff and also each Lab will require the machines.

1. If yes what would be the selling points?

**HP-**  People will welcome it because of the quick results.

**The research team**
