# Supplementary material for: ‘If I am on ART, my new-born baby should be put on treatment immediately’: Exploring the acceptability, and appropriateness of Cepheid Xpert HIV-1 Qual assay for early infant diagnosis of HIV in Malawi
Source: PLOS Glob Public Health. 2023 Mar 10;3(3):e0001135. doi: 10.1371/journal.pgph.0001135 (PMC10021387; doi:10.1371/journal.pgph.0001135)
Supplement: S2 File — (ZIP) [file pgph.0001135.s005.zip › Transcipts _Health _workers/DET002 HW .docx]

**DET002_HW_16_08_18**

1. Why do women have a lot more confidence in hospital staff?

**HW-** Chifukwa choti ndife achipatala ndipo amakhulupilira kuti athandizidwa.

**HW-B**ecause we are medical personnel and they have faith that we will help them

1. Why is it that caregivers especially women do not have anything to say when asked questions?

**HW-** Ndimawona ngati chifukwa choti ena sanapite ku sukulu, komanso ndimawona ngati chifukwa cha chikhalidwe kuti decision making imakhala ya mamuna ndipo zimenezi zimapangitsa kusayankha mafuso.

**HW-** I think because some may have not gone to school and cultures which state that a man is the one who is supposed to make decisions make them unable to answer questions.

1. Why is that caregivers hardly explain answers, their answers are very short? Eg on Anxiety about the window period?

**HW-** Ndimawona ngati amakhala ndi nkhawa tikawofokozera za window period chifukwa iwowo samachimvesetsa nchikana amavutika kuyankha.

**HW-** I think they have fear and concerns when we explain what window period is and they may not understand it properly

1. What is your opinion about testing for HIV among mothers whose partners are HIV positive?

**HW-** Ndimawona ngati sakhala safe makamaka kwa nzibambo amakhala ovuta pa nkhani yogwilitsa ntchito ma condom ndiye poti mzimayi ndi mzimayi amakanika ku kana amangovomera china chili chonse.

**HW-** I think they are not safe especially to men who resist using condoms and since a woman is a woman and they might not say no and say yes to everything.
