# Supplementary material for: ‘If I am on ART, my new-born baby should be put on treatment immediately’: Exploring the acceptability, and appropriateness of Cepheid Xpert HIV-1 Qual assay for early infant diagnosis of HIV in Malawi
Source: PLOS Glob Public Health. 2023 Mar 10;3(3):e0001135. doi: 10.1371/journal.pgph.0001135 (PMC10021387; doi:10.1371/journal.pgph.0001135)
Supplement: S2 File — (ZIP) [file pgph.0001135.s005.zip › Transcipts _Health _workers/DET003 HP.docx]

**DET003_HP_16_08_18**

As a healthy professional how do you feel

1. As you deliver this service of **Cepheid Xpert HIV -1 Quay assay using whole blood (Cepheid)** which involves taking blood.

**HP-** I feel good because it is my duty, and I can easily identify.

1. As you interact with a care giver where you are taking blood.

**HP-** It depends on the caregiver ngati ali ndi attitude zimakhala zovuta.

**HP-** it depends on the care givers attitude and if they have a negative one it is difficult.

1. If this way of HIV testing using whole blood is scaled above, do you feel other healthy workers will be interested in this method?

**HP-** Yes I think so, they would be interested because we are screening a lot of diseases.

1. Will it add any extra demand on the healthy services?

**HP-**  Zifunika kuwonjezera zipangizo ndi staff ikuyenera kukhala yochuluka.

**HP-** There is need for more equipment and enough medical staff

1. Do you feel you need a lot of time?

**HP-** Zikufunika kutengako nthawi kuti ziyende bwino.

**HP-** it needs time so it can proceed smoothly

1. Are the procedures involved easy to follow?

**HP-**  It easy to follow.

1. As you deliver this service, what is the general impression of parents and care givers as their children are having blood taken?

**HP-** Anthu amawoneka kuti alinazo chidwi kuti aziwe mmene mwana alili.

**HP-** People seem interested to know how their child is

1. EID results using DBS and PCR turn around time of results is 2-3 months, do you think the ministry of healthy would be interested in Cepheid whole blood protocol which takes 2hours?

**HP-**  Likhonza kusangalasidwa nawo chifukwa choti boma silisangalasidwa kuti ma results ena achedwe.

**HP-** They would be happy too because the government would not be happy with delayed results.

1. Do you think the government can afford HIV testing with Cepheid ?

**HP-** Yes

1. Can Cepheid whole blood protocol be scaled up?

**HP-** Yes

1. If yes what would be the barriers?

**HP-** Barriers can be on stuff and resources.

1. If yes what would be the selling points?

**HP-**  People will welcome it because of the quick results.

**The Research Team**
