# Supplementary material for: ‘If I am on ART, my new-born baby should be put on treatment immediately’: Exploring the acceptability, and appropriateness of Cepheid Xpert HIV-1 Qual assay for early infant diagnosis of HIV in Malawi
Source: PLOS Glob Public Health. 2023 Mar 10;3(3):e0001135. doi: 10.1371/journal.pgph.0001135 (PMC10021387; doi:10.1371/journal.pgph.0001135)
Supplement: S2 File — (ZIP) [file pgph.0001135.s005.zip › Transcipts _Health _workers/DET003 HW.docx]

**DET003_HW_16_08_18**

1. Why do women have a lot more confidence in hospital staff?

**HW-** Zimatengera ndimmene wamulandilira muntuyo kuti akhale omasuka.

**HW-** it depends on how you have welcomed the person

1. Why is it that caregivers especially women do not have anything to say when asked questions?

**HW-** Ndimawona ngati chifukwa choti sanapite ku sukulu ndiye zimakhala zovutilapo kuyankha mafuso.

**HW-** I think it is because they didn’t go to school so it is difficult for them to answer questions

1. Why is that caregivers hardly explain answerers, their answers are very short?eg Anxiety about the window period?

**HW-** Kwambiri ndimawona ngati chifukwa cha manyazi.

**HW-** I think it is mostly because of shyness

1. What is your opinion about testing for HIV among mothers whose partners are HIV positive?

**HW-**Athu amenewa sitingaziwe bwinobwino ngati angakhaledi negative komanso ngati akupitilira kukhala negative tikuyenera kuwalangiza mozitetezera.

**HW-** We might not be sure if they are negative and if they continue being negative we need to advise them to how they can protect themselves.
