# Supplementary material for: ‘If I am on ART, my new-born baby should be put on treatment immediately’: Exploring the acceptability, and appropriateness of Cepheid Xpert HIV-1 Qual assay for early infant diagnosis of HIV in Malawi
Source: PLOS Glob Public Health. 2023 Mar 10;3(3):e0001135. doi: 10.1371/journal.pgph.0001135 (PMC10021387; doi:10.1371/journal.pgph.0001135)
Supplement: S2 File — (ZIP) [file pgph.0001135.s005.zip › Transcipts _Health _workers/DET004 HP.docx]

**DET004_HP_16_08_18**

As a healthy professional how do you feel

1. As you deliver this service of **Cepheid Xpert HIV -1 Quay assay using whole blood (Cepheid)?** which involves taking blood.

**HP-** I feel good, more especially people are coming and they are willing to follow this method.

1. As you interact with a care giver where you are taking blood.

**HP-** Zikuwoneka kuti zilibwino chifukwa sitinapezepo vuto.

**HP-** It looks like it is okay because we didn’t find any problems

1. If this way of HIV testing using whole blood is scaled above, do you feel other healthy workers will be interested in this method?

**HP-** Yes they will because it is fast and reliable.

1. Will it add any extra demand on the healthy services?

**HP-**  Yes it can add up demand chifukwa anthu azibwera ambiri.

**HP-** it can add demand because people will be coming in large numbers

1. Do you feel you need a lot of time?

**HP-** We don’t need a lot of time kuti ma samples asabwereso ku Blantyre it takes a lot of time.]

**HP-** We do not need a lot of time

1. Are the procedures involved easy to follow?

**HP-** Yes

1. As you deliver this service, what is the general impression of parents and care givers as their children are having blood taken?

**HP-** Ena amakhala ndimantha koma tikawafotokozera bwinobwino akumachimvetsetsa.

**HP-** some have fear but when we clearly explain to them they understand.

1. EID results using DBS and PCR turn around time of results is 2-3 months, do you think the ministry of healthy would be interested in Cepheid whole blood protocol which takes 2hours?

**HP-**  Yes, chifukwa choti imapanga reduse work nzipatala zakutali zomwe zili ndi zipangizo.

**HP-** Yes because work is reduced in hospitals which have the equipment but are further from the people.

1. Do you think the government can afford HIV testing with Cepheid ?

**HP-** Yes if they have resources.

1. Can Cepheid whole blood protocol be scaled up?

**HP-**  Yes

1. If yes what would be the barriers?

**HP-** Understand ya anthu ingakhalae yovutilapo kuti atsatire njirazi koma with time.

**HP-** people’s understanding maybe hard but they may follow the strategies with time

1. If yes what would be the selling points?

**HP-**  It can be accesable and easy.

**HP-** it can be easily accessible

The Research Team
