# Supplementary material for: ‘If I am on ART, my new-born baby should be put on treatment immediately’: Exploring the acceptability, and appropriateness of Cepheid Xpert HIV-1 Qual assay for early infant diagnosis of HIV in Malawi
Source: PLOS Glob Public Health. 2023 Mar 10;3(3):e0001135. doi: 10.1371/journal.pgph.0001135 (PMC10021387; doi:10.1371/journal.pgph.0001135)
Supplement: S2 File — (ZIP) [file pgph.0001135.s005.zip › Transcipts _Health _workers/DET005 HP.docx]

**DET005_HP_16_08_18**

As a healthy professional how do you feel

1. As you deliver this service of **Cepheid Xpert HIV -1 Quay assay using whole blood (Cepheid)** which involves taking blood.

**HP-**  I feel good but the only problems is when taking blood from the vein.

1. As you interact with a care giver where you are taking blood.

**HP-** It depends on the caregiver ngati ali ndi attitude zimakhala zovuta.

**HP-** it depends on the attitude of the care giver

1. If this way of HIV testing using whole blood is scaled above, do you feel other healthy workers will be interested in this method?

**HP-**  Some will be interested some can not be and it will depend on counseling.

1. Will it add any extra demand on the healthy services?

**HP-**  We need to have extra stuff to shift up because this testing need a break.

1. Do you feel you need a lot of time?

**HP-** We don’t need a lot of time because it’s the easy way to find out your results.

1. Are the procedures involved easy to follow?

**HP-** Of course its not difficult but it has a challenge to find a vein in children under 6years.

1. As you deliver this service, what is the general impression of parents and care givers as their children are having blood taken?

**HP-** It depends on how the healthy center is counseling the caregiver.

1. EID results using DBS and PCR turn around time of results is 2-3 months, do you think the ministry of healthy would be interested in Cepheid whole blood protocol which takes 2hours?

**HP-**  Of course the minister can be interested.

1. Do you think the government can afford HIV testing with Cepheid ?

**HP-** The government can’t afford on its own.

1. Can Cepheid whole blood protocol be scaled up?

**HP-** Yes

1. If yes what would be the barriers?

**HP-** The barriers can be on the government, because the government on its own can not do this they will rely on funds.

1. If yes what would be the selling points?

**HP-**  People will welcome it because of the quick results.

**The Research Team**
