# Supplementary material for: ‘If I am on ART, my new-born baby should be put on treatment immediately’: Exploring the acceptability, and appropriateness of Cepheid Xpert HIV-1 Qual assay for early infant diagnosis of HIV in Malawi
Source: PLOS Glob Public Health. 2023 Mar 10;3(3):e0001135. doi: 10.1371/journal.pgph.0001135 (PMC10021387; doi:10.1371/journal.pgph.0001135)
Supplement: S2 File — (ZIP) [file pgph.0001135.s005.zip › Transcipts _Health _workers/DET005 HW.docx]

**DET005_HW_16_08_18**

1. Why do women have a lot more confidence in hospital staff?

**HW-** Ndi mmene wamulandilira munthuyo.

**HW-** its how they are welcomed

1. Why is it that caregivers especially women do not have anything to say when asked questions?

**HW-** Zimatengera ndi iweyo mmene wamulandilira muntuyo.

**HW-** depends on how you have received the person

1. Why is that caregivers hardly explain answerers, their answers are very short? eg Anxiety about the window period?

**HW-**Anthu amabadwa mosiya ena amavutika kamba ka sukulu.

**HW-** people are born different and some have troubles explain because they did not go far with school

1. What is your opinion about testing for HIV among mothers whose partners are HIV positive?

**HW-**Pamenepo umafu uziwe za mzimayiyo chifukwa ali pa high risk pokhala ndi mamuna oti ali ndi kachilombo ka HIV.

**HW-** There you would want to know about the mother because they have high risk of getting the virus because of their partner who is HIV positive
