# Supplementary material for: ‘If I am on ART, my new-born baby should be put on treatment immediately’: Exploring the acceptability, and appropriateness of Cepheid Xpert HIV-1 Qual assay for early infant diagnosis of HIV in Malawi
Source: PLOS Glob Public Health. 2023 Mar 10;3(3):e0001135. doi: 10.1371/journal.pgph.0001135 (PMC10021387; doi:10.1371/journal.pgph.0001135)
Supplement: S2 File — (ZIP) [file pgph.0001135.s005.zip › Transcipts _Health _workers/SUMMARY OF HEALTH WORKHER RESPONSES_ DET HP_1-5 .docx]

As a health professional how do you feel

1. As you deliver this Cepheid Xpert HIV -1 Quay assay using whole blood (Cepheid) which involves taking blood.

HP1- I feel good because despite that we using this method it seems this method is fast and it will help us to save lives in time.

HP2 He feels okay because it is part of his job.

HP3 - I feel good because it is my duty, and I can easily identify.

HP4 - I feel good, more especially people are coming and they are willing to follow this method.

HP5 - I feel good but the only problems is when taking blood from the vein.

1. As you interact with a care giver where you are taking blood.

HP1- it depends on how you have interacted with the person and how they have understood it.

HP2 Other workers will be happy with the process because the results do not take long time to be ready.

HP3 it depends on the care givers attitude and if they have a negative one it is difficult.

HP4 It looks like it is okay because we didn’t find any problems

HP5 it depends on the attitude of the care giver

1. If this way of HIV testing using whole blood is scaled above, do you feel other healthy workers will be interested in this method?

HP- We are used that we only draw whole blood when conducting full blood count so I think it would be hard for them to accept it.

HP2 Yes they will be interested because of the quick results.

HP3 Yes I think so, they would be interested because we are screening a lot of diseases.

HP4 Yes they will because it is fast and reliable.

HP5 Some will be interested some can not be and it will depend on counseling.

1. Will it add any extra demand on the healthy services?

HP1- We will need more teams and more equipment

HP2 It will need a lot of demand because of shortage of nurses.

HP3 There is need for more equipment and enough medical staff

HP4 it can add demand because people will be coming in large numbers

HP5 We need to have extra stuff to shift up because this testing need a break.

1. Do you feel you need a lot of time?

HP1- Yes we need a lot of time for orientation because a new person must appropriately learn everything.

HP2 Not that much time as long as the equipments are available.

HP3 it needs time so it can proceed smoothly

HP4 We do not need a lot of time

HP5 We don’t need a lot of time because it’s the easy way to find out your results.

1. Are the procedures involved easy to follow?

HP1- Not easy to follow as such because you need to focus if you don’t focus you can give wrong results.

HP2 Yes they are easy to follow.

HP3 It easy to follow

HP4 - Yes

HP5 - Of course its not difficult but it has a challenge to find a vein in children under 6years.

1. As you deliver this service, what is the general impression of parents and care givers as their children are having blood taken?.

HP1- They are expecting to get their results on the same day or in 5days time

HP2 Some are scared to the procedures need to be taught properly.

HP3 People seem interested to know how their child is

HP4 some have fear but when we clearly explain to them they understand.

HP5 It depends on how the healthy center is counseling the caregiver.

1. EID results using DBS and PCR turn around time of results is 2-3 months, do you think the ministry of healthy would be interested in Cepheid whole blood protocol which takes 2hours?

HP1- They would like it because of the reduced turn-around time.

HP2 The ministry of healthy will be interested because the whole process is very fast which will help.

HP3 They would be happy too because the government would not be happy with delayed results.

HP4 Yes because work is reduced in hospitals which have the equipment but are further from the people.

HP5 Of course the minister can be interested.

1. Do you think the government can afford HIV testing with Cepheid ?

HP1- With the economic problems we have I think it would be hard for them to buy the equipment

HP2 Yes the government can afford.

HP3 Yes

HP4 Yes if they have resources.

HP5 The government can’t afford on its own.

1. Can Cepheid whole blood protocol be scaled up?

HP1- it can be scaled up so people can go for training

HP2 Yes.

HP3 Yes

HP4 Yes

HP5 Yes

1. If yes what would be the barriers?

HP1- barriers would be there because if there are no lab personnel it would be difficult especially In the health centers since they are no labs.

HP2 The barriers will be there because of few staff and also each Lab will require the machines.

HP3 Barriers can be on stuff and resources.

HP4 people’s understanding maybe hard but they may follow the strategies with time

HP5 The barriers can be on the government, because the government on its own can not do this they will rely on funds

1. If yes what would be the selling points?

HP- it is possible in places where labs are available.

HP2 People will welcome it because of the quick results

HP3 People will welcome it because of the quick results.

HP4 it can be easily accessible

HP5 People will welcome it because of the quick results.

The research team
