# Supplementary material for: ‘If I am on ART, my new-born baby should be put on treatment immediately’: Exploring the acceptability, and appropriateness of Cepheid Xpert HIV-1 Qual assay for early infant diagnosis of HIV in Malawi
Source: PLOS Glob Public Health. 2023 Mar 10;3(3):e0001135. doi: 10.1371/journal.pgph.0001135 (PMC10021387; doi:10.1371/journal.pgph.0001135)
Supplement: S2 File — (ZIP) [file pgph.0001135.s005.zip › transcripts responses chichewa& english/ANSWERS 31-40.docx]

*A Questionnaire to validate new HIV tests called Cepheid Xpert HIV -1 Quay assay (Cepheid) in your hospital*

1. How would you as a parent/guardian feel if your child was to undergo HIV testing with Cepheid?

DET031 I would feel good because I will know how the child is.

DET032 I would feel good knowing how my child

DET033**-** I would feel okay with it because if found positive my child will be helped.

DET034 I would feel good knowing the HIV status of my child

DET035 I would feel good because after being tested I will know how my child is

DET036 I would feel good because I will know the child’s status

DET037 I would feel good because I would know the status of my child.

DET038- I would feel good when I know the results, I will know my child’s status

DET039- I would feel good because I will know the health status of my child

DET040 I would feel good about knowing my child’s status

1. What are your thoughts about these new strategies for testing HIV in children and giving results promptly?

DET031**-** It is a good idea to know the child’s status within a short time.

DET032 It is a good idea

DET033 I am happy because this new method will help us all

DET034 it’s a good method since all they want to know is their status

DET035 I am happy because this method has been established and I am excited because it is benefiting our wellbeing.

DET037**-** I am happy with it because this method wasn’t available before

DET038- I think this method is good because it will help us know the status of the child.

DET039- It is a good method because it will help us know status of our children

DET040 No thoughts on this

1. How should these approaches be implemented in a hospital? (Probe who should be targeted, why should they be targeted and why?)

DET031 The nurses needs to explain to the women when they come to the hospital. It needs to start with the children

**DET32-** no thoughts on this

DET033**-** You need to reach out to us through the antenatal clinic or conventions and it needs to start with adults so that we can know if it is okay for the children

DET034 Going in different areas and telling people. Start with children.

DET035 Doctors should explain and reach us as soon as possible and it should start with Children because they are the leaders of tomorrow.

DET036

DET037 The one that has come to the hospital should be responsible to tell others and it needs to start with children.

DET038 using conventions

DET039- Using conventions

DET040

4. How should issues of privacy of both children and their guardians be maintained?

DET031 It should only be between the doctor and parents

DET032

DET033**-** Parents and doctors are responsible in keeping privacy of the child

DET034 it depends on the parent telling people or not about the child’s status

DET035 You as doctros are the ones to help us with this by keeping it private

DET036

DET037 Doctors and parents must keep it a secret

DET038- Doctors and parents must keep it a secret

DET039 Parents and doctors need to keep the secret

DET040

5a. What should be the role of parents/guardians in the implementations of these approaches?

DET031 They should be taking their children for testing without which they would know nothing.

DET032

DET033 Men need to take part by also going for the test

DET034 The parents that have heard about Ceiphed should also tell their friends

DET035 We need to reach out to our friends to explain to them about this method

DET036

DET037 take part by getting tested

DET038 We can only take part by going for the test

DET039 Taking part by getting tested

DET040

5b. What information should be provided to ensure that guardians understand the procedures involved?

DET031

DET032

DET033

DET034 They need to be explain the whole procedure and the advantages and disadvantages of this method

DET035 A wise parent should welcome it because it is benefiting us

DET036

DET037 they should be told about the whole procedures

DET038 Should be told prevention measures and how you can contract the virus

DET039 They should be told how to prevent and how one can contract the virus

DET040

6. What should be the role of male partners in the implementation of these approaches? (Probe: How should male partners be encouraged to take active role in these approaches?)

DET031 Women need to encourage their husbands to get tested

DET032

DET033 They would be happy that such an important thing is very close to them

DET034 Men should encourage their wives to get tested

DET035 We need to encourage men to come and get tested too.

DET036

DET037**-** Men should be coming to the hospital with families to get tested and we should motivate them as a woman

DET038 Men should come for testing and also be told the importance of getting their children tested

DET039 Men should come get tested and be told the importance of getting children tested for a better life

DET040

7. How would your community feel if these approaches were to be implemented in your nearest health facility? (What could be done to encourage community members to participate in these interventions)

DET031 They can like it because of the short distance. They need to go through the village chief

DET032**-** because of difficulties in transportation, they would love the idea

DET033- This method is really important but I am only worried that my child could be found positive

DET034-They can like that this test is very close to them

DET035-We need to encourage men to come and get tested too.

DET036

DET037 They can feel good about it and we need to reach out to everyone through convections

DET038-Some can accept it but others can not

DET039 People can welcome this development

DET040

**`8. What are some concerns that you and some members in the community might have related to receiving HIV test results of a child?**

DET031**-** I am worried of the results of my child

DET032

DET033

DET034**-** I would get worried because we are not sure of the health status of the child

DET035 I would be sad but I would need to accept it for the child to grow to healthy

DET036

DET037 **-** Fear of death arises to those who do not understand and accept it but those who understand and follow instructions find a way to care for the young one.

DET038- Concerns and fear comes because we do not know our child’s status

DET039- Concerns come for the reason that people might laugh at an HIV positive child.

DET040

**9. Do you have suggestions or ideas for addressing possible community concerns about these HIV testing strategies?**

DET031 We need to get children tested so that we should not be in doubts

DET032

DET033 I should know how my child is and how I can care for her

DET034 **-** We should get proper counselling before the results

DET035 - The ones who are supposed to help us are medical personnel because we cannot stay without worrying.

DET036

DET037 **-** Prayer is the only thing that can take away the stress and following the prescription when taking the medicine

DET038- Concerns and fear comes because we do not know our child’s status

DET039- We need to take initiative by keeping our child’s secret so that he/she shouldn’t be laughed at when playing.

DET040

B. Perceptions about time to receive test results

**10. From the time that your child is tested, how long would you be patient enough to know results from the blood tests? (Same day, after three, after three months?)**

**Tsiku Lomwelo □**

**Patatha masiku □**

**Miyezi iwiri kapena itatu □**

**Fotokozani zifukwa zomwe mwasankhira Yankho limeneli**

DET031 Same day, Because if they do not hear the results the same day will be very stressed

DET032

DET033 Same day, because I believe when a test is performed in the laboratory, it does not take too long

DET034

DET035 **-** no reason for choosing 2 months

DET036

DET037 Same day, That when I am going home I should know the status and if there is need I should get the help I require.

DET038 Same day, to know what you should do depending on the results

DET 039 Same day because I need to know immediately how to take care of my child

DET040

11. **If your child is tested for HIV, how long would you want to wait before you are told that results from the tests are HIV positive? (same day, after three, after three months?)Explain why you would prefer your chosen answer.**

**Tsiku Lomwelo □**

**Patatha masiku □**

**Miyezi iwiri kapena itatu** □

Fotokozani zifukwa zomwe mwasankhira Yankho limeneli

DET031 **-** no comment

DET032

DET033 Same day because I believe when a test is performed in the laboratory, it does not take too long

DET034 no reason for choosing 3months

DET035

DET036

DET037 Same day, because I would be happy with the condition their child is in

DET038 It’s a good amount of days to hear the real truth about how a child is

DET039 According to the research protocol

DET040

12. **If your child test for HIV, how long would you want to wait before you are told that results from the test are HIV negative? (Same day, after three, after three months?)Explain why you would prefer your chosen answer.**

**Tsiku Lomwelo □**

**Patatha masiku □**

**Miyezi iwiri kapena itatu □**

**Fotokozani zifukwa zomwe mwasankhira Yankho limeneli**

DET031 Same day, no comment

DET032

DET033 Same day, Because I want to know my child’s status

DET034 no answer

DET035 Same day

DET036

DET037 Same day, Because I would be happy with the condition their child is in.

DET038 So that you should reach acceptance level

DET039 Because I know that for results to come out there are so many processes that happen

DET040

C.Acceptability and decision making

13. **What information would you want to be given to make an informed decision to accept that your child should get an HIV test or not? Explain**

DET031 importance of HIV and AIDs testing

DET032

DET033 Reach out to us when we come here the hospital

DET034 **-** The hospital personnel should explain the importance of this test

DET035 Reaching us here at the hospital

DET036

DET037 Doctors should tell people at the hospitals

DET038 Should receive motivating counselling about the result

DET039 I would like to be counselled on prevention measures

DET040

14**. How would you want to be approached and given information about these two HIV testing strategies? Explain**

DET031 following the counselling

DET032

DET033 yes

DET034 In any way the hospital is comfortable with

DET035 reach us via the village chief

DET036

DET037 This must reach local health Centre’s so that everyone can understand it.

DET038 Motivating counselling towards the results when they come

DET039 In our local communities or mobile phone

DET040

D.Potential Social Harms/Concerns etc.

15. **Would you encourage other parents/guardians to allow their children to test for HIV using these two approaches?**

Yes □ No □

DET031 yes

DET032

DET033 yes

DET034 yes

DET035 yes

DET036

DET037 yes

DET038 yes

DET039 yes

DET040

15b. **What would be your main concerns and worries towards these approaches?**

DET031 I have no stress because I want to know how the child is

DET032

DET033 **-** no comment

DET034 no problem with the testing method

DET035 I have no objections because I want to know how my child is.

DET036

DET037 I have no concerns

DET038 - No problems with this

DET039 - I have no concerns because I just need to protect my child

DET040

**16. How would you personally feel is someone from your community learns about HIV test results for your child?**

DET031 wouldn’t feel good hearing someone preach the results

DET032

DET033 no concerns

DET034 I would be sad because these results are supposed to be confidential

DET035 I would be okay with it because HIV is common these days and anyone can have it.

DET036

DET037 I would not have worries because they would just be rumors and not the truth.

DET038 I would not worry because at least my child has been helped

DET039 I wouldn’t feel good because these things need to be kept private

DET040

17. **Do you have any other thoughts you wish to share on this topic?**

DET031 no problem but just joy from the new method

DET032

DET033

DET034 I have no concerns

DET035 **-** I would recommend that you should continue with this method because it sounds really good.

DET036

DET037 **-** I think these new methods should also reach local health Centre’s and clinics so that those who couldn’t access the information can also get tested.

DET038 No other thoughts or concerns about Cepheid

DET039 No concerns

DET040

*The Research Team*
