# Supplementary material for: ‘If I am on ART, my new-born baby should be put on treatment immediately’: Exploring the acceptability, and appropriateness of Cepheid Xpert HIV-1 Qual assay for early infant diagnosis of HIV in Malawi
Source: PLOS Glob Public Health. 2023 Mar 10;3(3):e0001135. doi: 10.1371/journal.pgph.0001135 (PMC10021387; doi:10.1371/journal.pgph.0001135)
Supplement: S2 File — (ZIP) [file pgph.0001135.s005.zip › transcripts responses chichewa& english/Answers DET 41-60.docx]

*A Questionnaire to validate new HIV tests called Cepheid Xpert HIV -1 Quay assay (Cepheid) in your hospital*

1. How would you as a parent/guardian feel if your child was to undergo HIV testing with Cepheid?

DET 0043 I would be happy knowing my child’s status

DET 0045 I would feel good because how to act after hearing the results

DET 0047 - I would feel good because after hearing the results I will care for the child based from that

DET 0048 I would feel good because I would want to know how my child is and how to take care of her.

DET 0050 I would like it so much because I want to know my child’s status and how I may help him/her if need arises

DET 0051 She would feel good because she would know the child’s status

DET 0052 I would feel good because I would know how my child health is.

DET 0053 I would feel good because I want to know the status of my child.

DET 0054 I think it is good because it will help me know my child’s status

DET 0055 I would feel good because when tested I will know if the child has bright future or not.

DET 0056- I think this is a good way and it will help reduce child death which comes about due to the virus.

DET 0057 - I feel good because if the child is positive, she should start receiving medical help

DET 0058- She would be very happy because I want my child to be helped

DET 0059- I would feel good because I want to know the child’s status

DET 0060- I would feel good because in the world these days everyone needs to know their status

1. What are your thoughts about these new strategies for testing HIV in children and giving results promptly?

DET 0043 - I am pleased because I will help my child according to the results

DET 0045- No thoughts on this

DET 0047 - No thoughts on this

DET 0048- It is a good idea because I will know early on how my child is and how to help.

DET 0050

I think we should just wait for the results and how see how we can help and I am also thankful for these methods

DET 0051 - Because it is a faster way

DET 0052 - I am happy because when you know your child’s status you will know how you care for him/her.

DET 0053

DET 0054 - I think it is good that we will know earlier

DET 0055 It’s a good strategy because I will know how their child is because without testing you may just keep an HIV positive child without knowing.

DET 0056 I think it is helpful

DET 0057- No comment

DET 0058- She thinks this can really help the children

DET 0059- I have a good feeling about this new method.

DET 0060- It’s a good thing because we know the child’s status and how the child can be helped

3. How should these approaches be implemented in a hospital? (Probe who should be targeted, why should they be targeted and why?)

DET 0043 We should establish this by telling each other to get tested and we should start with children because they cannot make a choice on their own.

DET 0045 They should be taught after coming here to the hospital

DET 0047 People should be told about testing after coming to the hospital is one way of establishing this and we should start we women because they breast feed children.

DET 0048 - It should start here in the hospital and I choose children because they are important

DET 0050 We should start with children because they don’t know anything and here in the hospital.

DET 0051 For those who are done with testing they should also tell their friends and we should start with children because methods of testing adults are already there.

DET 0052 When we come for testing you need to tell us in a closed room and it should start with children because they cannot do it by themselves.

DET 0053- Motivating them to get children tested and we should start with the youths

DET 0054 Telling us here in the hospital and children because they are important

DET 0055 - We should target rural areas because people in the village areas have difficulties with travelling and we should start with children to know their future

DET 0056- Find time and spread the news about the test to our communities and start with children because all this time, there was no method for testing children.

DET 0057

DET 0058

DET 0059

DET 0060- The hospital personnel are the ones who should tell us and testing should start with kids because they are our future leaders

4. How should issues of privacy of both children and their guardians be maintained?

DET 0043 The doctor is the one who is supposed to keep a secret

DET 0045 The secret should be with the child’s parents

DET 0047- After getting tested you shouldn’t just tell everyone about your results.

DET 0048- The secret should be between the doctor and parent

DET 0050- It depends on the one doing the test because it is in their hands

DET 0051- Parents need to keep the results of their child to themselves

DET 0052- Only you and the hospital must know

DET 0053- Everyone should go for the test alone and not as a group

DET 0054- You need to hear this alone and keep it private

DET 0055- When we come for testing keep the results should be kept only by us parents and the doctors.

DET 0056- The secret should be between the hospital and parents

DET 0057

DET 0058

DET 0059

DET 0060- It must be kept private by the hospital

5a.What should be the role of parents/guardians in the implementations of these approaches?

DET 0043 Parents should take their children for testing

DET 0045 They should take part by bringing their child to the hospital for testing.

DET 0047 Women should take part in this testing method of Cepheid.

DET 0048 I would explain to my friends at home about the new method of HIV testing called Cepheid and how it can help our children.

DET 0050 By explaining to their friends the importance of this method

DET 0051 They should come to get tested using Cepheid

DET 0052- I would help in explaining this test to my friends so that they can also come to get tested

DET 0053 Take part by telling others to get tested using this method of Cepheid

DET 0054 No idea

DET 0055 - No idea

DET 0056- I need to explain to my friend at the village the importance of this test so that can also get their children tested.

DET 0057

DET 0058

DET 0059

DET 0060- As a parent my role is to get my child tested

b.What information should be provided to ensure that guardians understand the procedures involved?

DET 0043 - Assurance that the hospital stuff knows how to perform the test

DET 0045 It depends on how you receive it because some receive counselling and don’t use it.

DET 0047-No thoughts

DET 0048

DET 0050- They should be taught the importance of this method.

DET 0051- They need to be counselled by the hospital stuff

DET 0052- Telling us the dangers and prevention of the virus

DET 0053- Giving the counselling slowly without pressure and should be taught the importance of using this test.

DET 0054- The importance of the test and prevention of the virus

DET 0055 - When they come for antenatal clinic, Cepheid should be explained

DET 0056- You need to explain to people that this method is here for us.

DET 0057

DET 0058

DET 0059

DET 0060- You need to explain to people that this method is here for us.

6. What should be the role of male partners in the implementation of these approaches? (Probe: How should male partners be encouraged to take active role in these approaches?)

DET 0043 - Should be told so that they can be tested too.

DET 0045- Women must tell their husbands the importance of these strategies.

DET 0047- Men should also becoming for testing and women should also encourage men who are not willing to get tested.

DET 0048-Men should be taking part by getting tested to know their HIV status

DET 0050- It is a man’s duty to take part in this

DET 0051- Men should also get tested and women must motivate their husbands.

DET 0052- Motivating them into getting their children tested

DET 0053- Men should encourage the youth and their wives to get tested

DET 0054- Men also need to come for the test

DET 0055-Men should be told the importance of testing so they can know their statuses

DET 0056- Men need to also come for the test. You also need to reach out to them at the village.

DET 0057

DET 0058

DET 0059

DET 0060- Men are hard to deal with

7. How would your community feel if these approaches were to be implemented in your nearest health facility? (What could be done to encourage community members to participate in these interventions)

DET 0043 - I would be happy because it is close by rather than the usual long distance and the village headman should also encourage his people to get tested

DET 0045- some might accept it, some cannot and the village chief must host a convection.

DET 0047 I would feel good because everyone wants to know their results and the hospital must encourage people about testing using Cepheid.

DET 0048-- They would feel good knowing that the help they need for their child is readily available

DET 0050- They would feel good because transport is difficult in rural areas. And explaining the importance of blood testing

DET 0051- They would welcome it

DET 0052- I would feel good because this method did not exist before

DET 0053-- Some would like this but other would not and they need to encouraged to use this method

DET 0054 I would receive it well but others might not understand and conventions might help

DET 0055- they would like it because they would know the status of their children and explain to them the importance of Cepheid

DET 0056- They can welcome it because this method is here to help us, and you need to elaborate to them the importance of this.

DET 0057

DET 0058

DET 0059

DET 0060- -They would be happy

8. What are some concerns that you and some members in the community might have related to receiving HIV test results of a child?

DET 0043 - Some are concerned about the fact that if found with HIV you will be taking ARVs for the rest of your life

DET 0045- Concern is there because you don’t know the child’s status

DET 0047- No concern because it is all for helping the child.

DET 0048- I wouldn’t have any concerns because I want to be helped

DET 0050- I would receive it well because I would know ways of taking care for my child.

DET 0051- I would not have any concerns because I was prepared for any result

DET 0052- The concerns come about that if found positive the child will indeed receive assistance but she is too young

DET 0053- Stress over the upcoming results

DET 0054- Its sad to watch a child take daily ART that is where the concern comes in.

DET 0055- I would not have concerns because it is for fixing my child’s future

DET 0056- The worry comes in because when a child is found positive, as a parent you become sad.

DET 0057

DET 0058

DET 0059

DET 0060- I would only be grateful because I would know how to take care of my child

9. Do you have suggestions or ideas for addressing possible community concerns about these HIV testing strategies?

DET 0043 - Advising them that they shouldn’t be stressed out if found positive cause they will receive medications.

DET 0045 Doctors should offer counselling to reduce the persons stress when waiting for the results.

DET 0047- Those with fear should be told that If found infected it is not the end of everything.

DET 0048- By encouraging our friends that its not the end of everything if they are found positive and not discriminating them.

DET 0050- Motivating them that it is a good way and counselling them into getting their child tested.

DET 0051- Encouraging them that not to be worried if found positive

DET 0052- No idea

DET 0053- Those with fear and concerns must be taught that having the virus is not the end

DET 0054 Encourage them to not be worried

DET 0055- Those with concerns should be told it cannot kill them on the same day but they should follow the doctor’s instruction

DET 0056- They need to be encouraged that being positive is not the end, they can take medicine and live a healthy life.

DET 0057

DET 0058

DET 0059

DET 0060- They need to be encouraged that being found with the virus is not the end of the world since the ART adds more days to life

B. Perceptions about time to receive test results

10. From the time that your child is tested, how long would you be patient enough to know results from the blood tests? (Same day, after three, after three months?)

Tsiku Lomwelo □

Patatha masiku □

Miyezi iwiri kapena itatu □

Fotokozani zifukwa zomwe mwasankhira Yankho limeneli

DET 0043 Same day, to know the child’s status

DET 0045- Same day, to know my child’s status and how I should care for him/her

DET 0047- Same day, So that if found with the virus he/she should receive medical help.

DET 0048- No idea

DET 0050 Same day, I chose this because I want to know how he is

DET 0051- Same day, it’s because I want to know fast and for my child to start getting assisted in good time

DET 0052- it’s because I need to go home to think about it properly before I receive the results

DET 0053 Same day, It is because waiting is very painful

DET 0054- To know and be free

DET 0055- Same day, she has other things to do so she cannot wait to get the results the same day

DET 0056- Same day, Because I need to know the health status of my child and help him promptly

DET 0057

DET 0058

DET 0059

DET 0060- Same day, Because I want to know how to take Care of my child

11. If your child is tested for HIV, how long would you want to wait before you are told that results from the tests are HIV positive? (same day, after three, after three months?)Explain why you would prefer your chosen answer.

Tsiku Lomwelo □

Patatha masiku □

Miyezi iwiri kapena itatu □

Fotokozani zifukwa zomwe mwasankhira Yankho limeneli

DET 0043 – Same day, because I stay very far from the hospital

DET 0045- with consideration on transport I would prefer 3days

DET 0047- Same day, No reason for choosing same day

DET 0048- Same day, Because I will know how my child is and encourage my friends on the importance of testing using Cepheid methods

DET 0050- I choose 3 days because I need to Know the results and receive correct guidance.

DET 0051- Same day, for my child to start getting medical assistance.

DET 0052- Because I need to discuss with my husband first.

DET 0053- Same day, because when you get tested and time passes by you might forget.

DET 0054

DET 0055- it is usually the hospital personnel that tells us to wait for a few days

DET 0056 – Same day, I just need to know how my child is

DET 0057

DET 0058

DET 0059

DET 0060- Same day, it is exciting to hear when the child is negative and to learn how to protect her

12. If your child test for HIV, how long would you want to wait before you are told that results from the test are HIV negative? (Same day, after three, after three months?)Explain why you would prefer your chosen answer.

Tsiku Lomwelo □

Patatha masiku □

Miyezi iwiri kapena itatu □

Fotokozani zifukwa zomwe mwasankhira Yankho limeneli

DET 0043 – 3 months, because sometimes the virus might hide so it is better 3 months

DET 0045- No thoughts on this

DET 0047- Any time the hospital chooses to release the results

DET 0048- No thoughts here

DET 0050— Same day, because I have to know how I will take care of my child.

DET 0051- Same day because that is what the medical personnel said

DET 0052- I have no idea

DET 0053- Same day, No reason as to why I have chosen same day

DET 0054

DET 0055- Same day, because it will nice to know the child is alright and protected from contracting HIV

DET 0056

DET 0057

DET 0058

DET 0059

DET 0060 Same day, I want to know how my child is and how I can help her

C.Acceptability and decision making

13. What information would you want to be given to make an informed decision to accept that your child should get an HIV test or not? Explain

DET 0043 – Should be encouraged that if the child is found negative he/she will be cared for and if found positive should start medication

DET 0045 - I need to receive good counselling without discrimination

DET 0047- It is up to me, without need for the hospitals counsel.

DET 0048- We should get counselling from the doctor

DET 0050- I would not expect any counselling because it is up to me to get my child tested

DET 0051- No thoughts on this

DET 0052- By explaining how we can protect ourselves and prevent this virus

DET 0053- - Counselling of what to do if found with the virus

DET 0054- Medical personnel should advise us about the testing and give us counselling

DET 0055- Should Receive the collect counselling on how her child can be protected from contracting HIV and AIDS

DET 0056 You need to counsel us and we need to get our child tested if he gets sick regularly

DET 0057

DET 0058

DET 0059

DET 0060 I want to know how my child is and how I can help her

14. How would you want to be approached and given information about these two HIV testing strategies? Explain

DET 0043- During her visit to the hospital

DET 0045- I choose finding me here in the hospital and telling me about Cepheid

DET 0047- I should be told about testing after coming to the hospital

DET 0048- You can reach me at my house

DET 0050- It will depend on the choice of the hospital

DET 0051- Get advice about Cepheid after coming to the hospital

DET 0052- You should reach us by using the hospitals and posters in hospitals.

DET 0053- No idea on this

DET 0054- It will depend on the doctor telling us the importance of testing.

DET 0055- Reaching communities and telling them about this

DET 0056- Reaching us in our villages ,radios even hospitals

DET 0057

DET 0058

DET 0059

DET 0060 I would prefer if you reach out to me when I come to the hospital

D.Potential Social Harms/Concerns etc.

15. Would you encourage other parents/guardians to allow their children to test for HIV using these two approaches? What would be your main concerns and worries towards these approaches?

Yes □ No □

DET 0043 yes

DET 0045 yes

DET 0047 yes

DET 0048 yes

DET 0050 yes

DET 0051yes

DET 0052 yes

DET 0053 yes

DET 0054 yes

DET 0055 yes

DET 0056 yes

DET 0057

DET 0058

DET 0059

DET 0060 yes

15.B

DET 0043 No concerns

DET 0045 - My concern is that if my child has the virus, I would be extremely sad.

DET 0047-- No worries about this

DET 0048-- I have no problem but my fear is the venous blood draw. I am afraid of it leaving a wound on the puncture site.

DET 0050- I do not have any concerns or worry because this method is there to how our community

DET 0051- No worries here.

DET 0052- My concern is on the fact if found positive, I would be stressed.

DET 0053- When they cannot find the vein, there is fear that they might hurt the child.

DET 0054 I have no concerns but joy because this needs to established.

DET 0055 No concern because it is for the child’s wellbeing

DET 0056- My problem is with the amount of blood taken and what they do with it

DET 0057

DET 0058

DET 0059

DET 0060- I would not be worried because you are drawing the blood with intentionsof helping my child

16. How would you personally feel is someone from your community learns about HIV test results for your child?

DET 0043 - I would be sad because a parent is the only one who is supposed to know.

DET 0045- It would be hard because we have different hearts and someone might preach my results to the community.

DET 0047- I Would not feel good because privacy is necessary

DET 0048

DET 0050- I would be feel hurt because there is a need for privacy.

DET 0051- I would not feel bad because right now issues of HIV/AIDS are everywhere.

DET 0052- I would not be happy because this is supposed to be kept secret

DET 0053- I would not be happy because the person might spread rumors

DET 0054 I wouldn’t be sad but rather tell them to get their child tested too.

DET 0055- I would not feel good because the person would be spreading news about my child and it supposed to a private

DET 0056- I would not feel good because everyone needs their privacy.

DET 0057

DET 0058

DET 0059

DET 0060 I would not feel bad because there would be no time for worries but thinking about the future

17. Do you have any other thoughts you wish to share on this topic?

DET 0043 - No problem with this but I hope that it should just continue

DET 0045- No problem with this

DET 0047- No further thoughts.

DET 0048- My worry is on the fact that the blood you have taken might make the child sick and I wouldn’t know what to do then

DET 0050- No problem or concerns

DET 0051- I am grateful because we never used to have this methods in the past

DET 0052 I have no further questions on this

DET 0053- I think that we should encourage these ways because it is helping people know their children’s health Status

DET 0054 I don’t have any more concerns

DET 0055- I have no concerns

DET 0056 I think this is a very good method because it was not there before, We will be able to know very fast the status of our children and they will be getting the help they need fast.

DET 0057

DET 0058

DET 0059

DET 0060- I have no concerns

*The Research Team*
