# Supplementary material for: ‘If I am on ART, my new-born baby should be put on treatment immediately’: Exploring the acceptability, and appropriateness of Cepheid Xpert HIV-1 Qual assay for early infant diagnosis of HIV in Malawi
Source: PLOS Glob Public Health. 2023 Mar 10;3(3):e0001135. doi: 10.1371/journal.pgph.0001135 (PMC10021387; doi:10.1371/journal.pgph.0001135)
Supplement: S2 File — (ZIP) [file pgph.0001135.s005.zip › transcripts responses chichewa& english/ANSWERS FOR DET 1-10.docx]

*A Questionnaire to validate new HIV tests called Cepheid Xpert HIV -1 Quay assay (Cepheid) in your hospital*

1. How would you as a parent/guardian feel if your child was to undergo HIV testing with Cepheid?

DET 001 They would feel okay because they would know the results.

DET 002 I would feel like it because I would know the status of my child.

DET 003 **-** I support this because I would hear the results on the same day

DET 004 - I would receive it in a good way because I would want to know how the child is, on the same day.

DET 005 - no problem because I only want to know how my child is.

DET 006 **-** This method looks useful

DET 007 I would feel good because I would know the results on the same day.

DET 008 No problem because the point is knowing how my child is.

DET 009 it is good because when drawing blood like this and it is screened you may be helped

DET 010 I would feel good because I would want to know my child’s status

1. What are your thoughts about these new strategies for testing HIV in children and giving results promptly?

DET 001 It Is a good idea because using this method we can know the status of the child and how we can take care of him/her if found infected and if found negative how we can prevent

DET 002 I think I need to know the results the same day

DET 003 its good because you hear the results on the same day.

DET 004 I think it’s a good way because I will know the status of my child’s body and how I will help him/her.

DET 005 The problem is that there are not many doctors who draw blood from the veins

DET 006 I greatly welcome this because this method help us know the status of our child’s health quickly

DET 007 I like this method because I will know is the child is negative or positive

DET 008 No problem, but my concern is that there are a few doctors capable of doing this.

DET 009 I think this will help us

DET 010 It is a good idea because it will help us get the results.

3. How should these approaches be implemented in a hospital? (Probe who should be targeted, why should they be targeted and why?)

DET 001 Everyone with a child should take part. Starting with children because they cannot manage to go get tested on their own

DET 002 Telling the community people and everyone else We need to start with children because it is helpful to protect our child as early as possible.

DET 003 It should start with children because it takes time to know their status while the older people get tested regularly

DET 004 we should start with the children because we have a lot of children and we have waited for a long time for a method like this.

DET 005 These ways should be implemented in hospitals, because that is where you find a lot of women and children. When spreading the message use posters. we should start with the adults because when you cannot find blood vessels of a child, parents might complain that you are hurting their child.

DET 006 we need to reach village heads and explain to them about how this testing takes place and they should start with children because we didn’t have it earlier on.

DET 007 They should start with the children because if I am on ART, I should know that my child should receive help as soon as possible as well.

DET 008 Starting with the adults because when they know their status they might get their children tested.

DET 009 Since you are the ones who work in the hospital, you should see for yourself which group to start with

DET 010 Women should be doing things according to the doctor

4. How should issues of privacy of both children and their guardians be maintained?

DET 001

DET 002 only one who can keep your secret is you thereby we need to keep it to ourselves.

DET 003 a parent should keep the child’s privacy because the child has not started talking

DET 004 we as parents must keep this private.

DET 005 testing one person at a time for privacy

DET 006 Giving good counselling to the guardians and how it is their right to keep it a secret

DET 007 The guardian should be told to keep it private.

DET 008 people must come to the hospital alone because if someone goes with them the village people would know they went for testing.

DET 009 The doctors are the ones who know the process so however they do it is okay.

DET 010 it should start with the children because adults already have their methods.

5a. What should be the role of parents/guardians in the implementations of these approaches?

DET001 Only the parent of the child and doctor must know to keep it secretive

DET002 Explaining the process to our friends

DET003 parents should allow children be tested using this method because if they don’t they are destroying their child’s future

DET004 parents tell others because this method brings results the same day.

DET005 bringing children to the hospital without hesitation.

DET006 Parents need to receive and accept this type of testing

DET007 As parents we can tell other parents to come and take part.

DET008 Telling other parents to come to the hospital with their children

DET009 No comment

DET010 Our full participation

5b. What information should be provided to ensure that guardians understand the procedures involved?

DET 001

DET 002 Any way you can advise because you are the ones who gives proper counseling

DET 003 it is good because if they know the status of the child, they will know how they can take care of him/her is infected or not

DET 004 Parents need to be given good counselling and a good explanation on this method of testing using Cepheid and cause life of their kid is in their hands.

DET 005 they should know the reason for Cepheid and why the method will help the child as soon as possible.

DET 006 we need to explain with the importance of the test

DET 007 I can understand in any way.

DET 008 Telling other parents to come to the hospital with their children

DET 009 to clearly understand they need to go to the hospital and get the correct counsel

DET 010 Listening to the advice the doctor gives about the method.

6. What should be the role of male partners in the implementation of these approaches? (Probe: How should male partners be encouraged to take active role in these approaches?)

DET 001 They need to understand it and take part in spreading the message in villages

DET 002 Any way you can advise because you are the ones who gives proper counseling

DET 003 They can take part by motivating their wives to get tested using these ways which are fast and you don’t have to wait for a long time.

DET 004 we need to assure them that these ways are right because the results come out on the same day, and they also need to take part so our child should be healthy and strong.

DET 005 they should know the reason for Cepheid and why the method will help the child as soon as possible.

DET 006 we need to explain with the importance of the test

DET 007 We can tell the husbands to come and get tested or bring the children for testing.

DET 008 Understanding husbands would encourage their wives to go get their child tested

DET 009 Men should take part in what they feel is right.

DET 010 They should take part because they are also parents and their wives should tell them the importance of Cepheid

7. How would your community feel if these approaches were to be implemented in your nearest health facility? (What could be done to encourage community members to participate in these interventions)

DET1 During village conventions and radios.

DET2 They would like it because everyone wants to know their results.

DET3 explaining to the village head the importance of these new methods so he can host a convention.

DE4 this can be good because we will not walk a long distance to know the results of our child.

DET5 they would be happy, explaining the importance of these methods so they can understand that it is fast and holding convections.

DET6 it would be wonderful because transport would be easy and we would be motivated to get tested.

DET7 They would be happy because it is easier and good.

DET8**-** People would receive it positively because everyone wants to know their status.

DET9 This is what is needed in this world because everyone wants life

DET10**-**They can welcome it because there would no longer be transport problems

8. What are some concerns that you and some members in the community might have related to receiving HIV test results of a child?

DET1 it would be good because many village people are less likely to get tested because hospitals are usually far but if it were to happen like that it would be helpful.

DET2

DET3**-** the fear comes because other people might discriminate the child in the community

DET4 I wouldn’t have concerns because I will know the results of my child and know how to prevent and take care of him/her

DET5**-** my fear would arise towards the fact the child might be getting sick and need good food because

DET6 Everyone who is tested has fear because they are afraid if found positive they may die

DET7 As a parent, I would not have any concerns because if something was to happen to my child, it would be my fault.

DET8 I am worried that if positive my child would need good food and will be sick regularly.

DET9 You get worried because you want to know if the child is fine

DET10 Fear arises for the fact that they might be found with HIV.

9. Do you have suggestions or ideas for addressing possible community concerns about these HIV testing strategies?

DET1 The fear comes from how the child will be raised healthy if found with the virus

DET2**-** No comment on this

DET3**-** People who have the virus should openly come to air and tell everyone the virus isn’t the **-**

DET4 way of reducing the stress can be praying and working hard on my child so that the child should have a great future

DET5 People need to be in groups and talk thereby taking away the fear.

DET6 Prayer is the beginning of a fearless life and helping each other

DET7 counselling them on why they should have no fear because some have suicidal thoughts and they need to know it is not the end of everything.

DET8 Prayer is the only answer.

DET9**-** To reduce stress you need to go to the hospital for testing

DET10 Being brave is all that is needed

B. Perceptions about time to receive test results

10. From the time that your child is tested, how long would you be patient enough to know results from the blood tests? (Same day, after three, after three months?)

Tsiku Lomwelo □

Patatha masiku □

Miyezi iwiri kapena itatu □

Fotokozani zifukwa zomwe mwasankhira Yankho limeneli

DET001

DET002 same day, So that I know the results and when going back homw I should have confidence

DET003 same day so that I will know how my child is the same day and give him help as soon as possible.

DET004 Same day, waiting is painful, I would prefer knowing the results on the same day, because I will know how to prevent or help my child.

DET005 Same day, because if it takes too long you might forget your child’s blood was taken for testing.

DET006 Same day, it will help us know how we will take care of our child.

DET007 same day because if there is need, the child should receive help on the same day

DET008 Same day, Because when you leave home you expect to hear the results on the same day

DET009 Same day, Because I stay very far and I need to know the results

DET010 Same day, Because I would be walking with pride knowing the results of my child

11. If your child is tested for HIV, how long would you want to wait before you are told that results from the tests are HIV positive? (same day, after three, after three months?)Explain why you would prefer your chosen answer.

Tsiku Lomwelo □

Patatha masiku □

Miyezi iwiri kapena itatu □

Fotokozani zifukwa zomwe mwasankhira Yankho limeneli

DET001 same day, when they come for testing they need to hear the results on the same day because they have worries about the possible results

DET002 Same Day, I choose to hear the same day because it is what I was expecting.

DET003 Same day, to get the correct guidance in due time

DET004 Same day, because you are waiting to hear how your child will be so its important to know fast to see how you will help the child

DET005 Same day, so I will know how I will take care of my child is found positive or not.

DET006 According to how long the doctor tells us to wait

DET007 No comment

DET008 After three days, because when you leave home you expect to get results as soon as possible.

DET009

DET010 Same day because you will be walking with pride after knowing the results.

12. If your child test for HIV, how long would you want to wait before you are told that results from the test are HIV negative? (Same day, after three, after three months?)Explain why you would prefer your chosen answer.

Tsiku Lomwelo □

Patatha masiku □

Miyezi iwiri kapena itatu □

Fotokozani zifukwa zomwe mwasankhira Yankho limeneli

DET001 Same day, Because of transport problems for us village people it would take a lot of time to come back and hear the results.

DET002 it is the right of everyone to know the results as soon as possible to know how they will protect themselves.

DET003 Same day, if we know on the same day we will know the future of the child

DET004 Same day, I just think so

DET005 Same day, Because it is the will of every parent to know the results of their children so that they will know how they will take care of him/her

DET006 Same day, because I will know if my child needs to take medications or not.

DET007 Same day, it is the will of every parent to know on the same day

DET008 Same day, Because when leaving home, you expect to immediately get the results.

DET009

DET010 Same day, Because you will be walking with pride after knowing the results.

C.Acceptability and decision making

13. What information would you want to be given to make an informed decision to accept that your child should get an HIV test or not? Explain

DET001

DET002 no thoughts on this

DET003 The child will be taken care in accordance with the results

DET004 if you would explain the preventative and ways to care for the child, I would be extremely happy.

DET005 To be encouraged that when they get tested, they will receive the needed help

DET006 us as parents need to be counselled and the counsellor needs to explain clear step by step.

DET007 no comment

DET008 I would just need to accept anything

DET009 I would want to go with the child to the hospital so the doctor can provide me with good counselling

DET010 We should be taught the importance of knowing a child’s HIV status.

14. How would you want to be approached and given information about these two HIV testing strategies? Explain

DET001 Helping them understand the importance of blood testing

DET002 no thoughts on this

DET003 I would be happy if you could reach us indiscriminately to help us receive the correct guidance.

DET004 I think it Is better at antenatal clinic so that we can all listen together

DET005 Reach then using the radio and plays even posters should be posted at the hospitals

DET006 using radios and different posters in hospitals

DET007 finding us in our home villages and teaching us the cepheid method.

DET008 Through radios or televisions and posters

DET009 no comment

DET010 no idea

D.Potential Social Harms/Concerns etc.

15. Would you encourage other parents/guardians to allow their children to test for HIV using these two approaches? What would be your main concerns and worries towards these approaches?

Yes □ No □

DET001 Yes

DET002 Yes

DET003 Yes

DET004 Yes

DET005 Yes

DET006 Yes

DET007 Yes

DET008 Yes

DET009 Yes

DET010 Yes

16. How would you personally feel is someone from your community learns about HIV test results for your child?

DET001 A child might feel pain during venous blood draw

DET002 I would be okay with it because their desire has been fulfilled

DET003 I wouldn’t feel good because issues about HIV should be kept private unless its your choice to tell people if your child is positive.

DET004 I have no idea

DET005 These days I wouldn’t be too worried because if I know I will know how I will take care of my child.

DET006 we need to know it is within our right to protect the life of our child and its not right to allow someone else results because me as a parent need to take part in protecting my child.

DET007 I would not feel good about it

DET008 no comment

DET009 I would feel good because I would want to know how my child is

DET010 I would feel good because I would want to know how my child is

17. Do you have any other thoughts you wish to share on this topic?

DET001 Questions arise with what is being done with the large amount of blood taken.

DET002 I have no problem with this

DET003 I have no comment or question because the results will be stated immediately.

DET004 no concern.

DET005 My concern is that it is a new thing so I will not know if the results will really be out on the same day.

DET006 I have great concern with where the blood goes after being taken

DET007 I think you should encourage people to know their results earlier.

DET008 No comment

DET009 no comment

DET010 no concerns

*The Research Team*
