# Supplementary material for: ‘If I am on ART, my new-born baby should be put on treatment immediately’: Exploring the acceptability, and appropriateness of Cepheid Xpert HIV-1 Qual assay for early infant diagnosis of HIV in Malawi
Source: PLOS Glob Public Health. 2023 Mar 10;3(3):e0001135. doi: 10.1371/journal.pgph.0001135 (PMC10021387; doi:10.1371/journal.pgph.0001135)
Supplement: S2 File — (ZIP) [file pgph.0001135.s005.zip › transcripts responses chichewa& english/ANSWERS FOR DET 11-30.docx]

*A Questionnaire to validate new HIV tests called Cepheid Xpert HIV -1 Quay assay (Cepheid) in your hospital*

1. How would you as a parent/guardian feel if your child was to undergo HIV testing with Cepheid?

DET 011 I would feel good because I want to know if my child is okay or not so that he can be helped as soon as possible

DET 012 I can feel good because I would know the status of my child

DET 013 I would feel good because that would be what I was expecting so that the child can be helped

DET 014 I would feel good with the change

DET 015 I would feel good because I would know the status of my child.

DET 016 I have nothing to say because it is the government’s will

DET 017 I would feel good because I want to know the status of my child

DET 018

DET 019 it is a good way because when they draw venous blood, the results are faster

DET 020 it is a good way because we want to know the status of the child

DET 021 I would feel good knowing my child’s status

DET 022 I would feel good because I would want to know the results

DET023 I would feel good because it is the child’s future

DET024 Blood sample of a child need to been drawn from the vein because you can not find enough blood on the finger

DET025 I would be okay with it because it is my child’s future

DET026 **-** I would feel good because it is the child’s future

DET027 I would feel good knowing the status of my child

DET028 I would feel good because I would know how my child is quickly.

DET029 I would like it because it is for the future of my child

DET030 I would not be comfortable with venous blood draw but I would accept it because I want my child to be helped.

1. What are your thoughts about these new strategies for testing HIV in children and giving results promptly?

DET 011 it can help because I know that immediately we will get the needed help

DET 012 no comment

DET 013 Very happy because this method was not available and now that it is here, I’m happy.

DET 014**-** I consider it a good method because results don’t take long to come

DET 015 no comment on this

DET 016**-** I think that this will help us know quickly if our child is infected or not.

DET 017**-** no idea

DET 018

DET 019 when you know your child’s status, it is very good because you might receive help as soon as possible and you will know how to care for the child.

DET 020

DET021 No idea

DET022 it is a good method because you hear the results in short time

DET023 I would be okay with it and I would and I would follow Doctors advise

DET024 the child should be immediately tested and results should be out because waiting for the child to grow would be a bit hard because he/she might get sick

DET025 **-** it is a good idea because It will help me know if my child has the virus or not.

DET026 It is good because things are moving faster rather than in the old days when we didn’t have this way.

DET027 it is a good idea knowing the child’s status

DET028 I think it very good because I’ll now know the status of my child

DET029 I like this because I want to know the status of my child

DET030 **-** I like this method because it is faster than the past methods

3. How should these approaches be implemented in a hospital? (Probe who should be targeted, why should they be targeted and why?)

DET 011**-** They should start with children because as a child you do not know what is wrong with you

DET 012**-** Reach out to people when they come to the hospital and start with the adults.

DET 013 both children and adults

DET 014 People should get more children tested because they are more important than adults

DET 015**-**When a person comes to the hospital they can be told about this and we should start with the adults.

DET 016 We should start with children because this was not available before

DET017**-** Children because they cannot get tested on their own

DET018

DET019 I think we should start with children because they are the leaders of tomorrow and them being leaders need to be protected.

DET020 Start with children because it is easy for adults to know their status

DET021 it should start with kids because they cannot go to for the test alone

DET022 Start with the children because it is hard to get them tested

DET023 It should be according to the doctors and it should start with the adults because they are the ones that transmit the virus

DET024 this message must reach parents and we should start with parents because they are the ones who can take their child to the hospital

DET025 Using pediatrics wards, explain the importance of the methods. Start with children

DET026 We should establish that people should be receiving things quickly and start with children because we would just see signs of malnutrition not knowing what may be wrong with them.

DET027 Nurses need to explain the importance’s of this testing method and they also need to start because we do not have testing methods like this

DET028 no comment but we should start with children because they are the leaders of tomorrow

DET029 Reach us through the peadatrics wards and start with children

DET030 It needs to be implemented firstly to children because sometimes they get malnourished and we do not know what they are suffering from

4. How should issues of privacy of both children and their guardians be maintained?

DET011**-** We are supposed to go to the hospital alone not as group

DET012 It should be between the doctor and parents

DET013 It depends with the person keeping the secret

DET014**-** The child should be told by the parents or guardian.

DET015**-** discuss the results only with the parents without telling other people

DET016 I think there is no privacy.

DET017**-** no answer

DET18

DET019**-** it is not good that someone else knows because some people have corrupt mindsets which might destroy a child’s future

DET020**-** it is required that the doctor must know the results of the person tested and tell no one else

DET021 - I have no thought

DET022 Telling them the results in a closed room so that it should be up to the owner to tell people.

DET023 Only the parents and the child should go to the hospital not accompanied by anyone else

DET024 Telling the child’s parent and no one else

DET025 The secret is between the mother and child.

DET026 Testing should take place in a confined room

DET027 **-** It is supposed to be between the doctor and the one getting tested.

DET028 Only give the results to the parents

DET029 - The child should keep this confidential

DET030 **-** The process needs to be done in a private room

5a. What should be the role of parents/guardians in the implementations of these approaches?

DET011**-** I can take part by explaining to my friends in my community about the new fast way of testing

DET012**-** They should come to the hospital to learn more about the strategy.

DET013**-** It will depend on the person accepting it.

**DET0**14 Letting their friends know about the method so that they can go too.

DET015 They should go to the hospital to know how this method would be established

DET016**-**I already took part in this by coming with my child.

DET017 They should just support this methods

DET018

DET019 They should go to the hospital for testing using Cepheid methods.

DET020 They should also go and get tested and tell people close to them about the new testing

DET021

DET022 For every parent that loves their children it is easy to understand this method and for them to tell other people

DET023 I do not know

DET024 understanding and telling our friends the message

DET025 Taking part by getting their children tested using Cepheid

DET026 Encouraging the helping organizations to come and assist

DET027 They should be taking their children to get tested

DET028 understanding the message and telling our friends

DET029 **-** I will take part by having my children tested

DET030 Encouraging the sponsors that they should help out in setting up this methods

5b. What information should be provided to ensure that guardians understand the procedures involved?

DET011 No comment here

DET012 They should be told with love and care

DET013 no comment

DET014 Good guidance and counselling

DET015 They should be told with love and care

DET016 hosting a convection

DET017 I have no idea

DET018

DET019 we should receive the guidance and counselling from medical personnel

DET20 I am just grateful to the new testing method

DET021 I have not answer

DET022 **-** Advise them how they can take care of their child after they get the results

DET023 **-** Helping them understand and know the importance of this method .

DET024 should tell us the ways which we can use and also on how we can care for our child.

DET025 Counselling concerning the importance of this method

DET026 we need to receive counselling.

DET027 Following the doctors advise

DET028 We should be told how to take care of our child

DET029 Be counselled on the importance of this method

DET030 They need to be advised on the whole process on the test

6. What should be the role of male partners in the implementation of these approaches? (Probe: How should male partners be encouraged to take active role in these approaches?)

DET011 Men can take part by coming for the test. We need to encourage them the results come out same day.

DET012 Men can take part by coming for the test. We need to encourage them the results come out same day.

DET013 I would tell them about the new ways of testing blood of a child and the results come on the same day and verify that it is a good way.

DET014 They should receive counselling and women should encourage them when they get home.

DET015 Men should be coming with their wives to get tested

DET016 I do not know how men can take part.

DET017 Men must encourage doctors that they should not stop testing

DET018

DET019 Men must take a huge part because they are the ones that might transmit the virus to the wife and women must encourage their husbands to take part.

DET020 Men should also come for testing at the hospital

DET021 Men should encourage the doctors towards these ways and encourage them to get their child tested

DET022 The same way this issue is being handled with women because the child is for them both

DET023 Even men can come for testing and also get counselling from the hospital.

DET024 we need to explain to them and tell them to get tested and when getting tested we need to go with our husbands and tell them the dangers of HIV.

DET025 When they need to be tested, they should go to the hospital

DET026 when they understand they should also come for testing to know their bodies status

DET027 Women need to encourage their husbands to get tested

DET028 When we come with for the test we should bring husbands along and tell them the importance of the test

DET029 They should come to the hospital for testing

DET030 They should be encouraged to be coming for testing in early stages

7. How would your community feel if these approaches were to be implemented in your nearest health facility? (What could be done to encourage community members to participate in these interventions)

DET011 They would be happy because they would see this method has been implemented close to them.

DET012 They can welcome it. Encourage them to come and meet the doctor for the testing

DET013 I would be happy because we did not have methods for testing children.

DET014 They would be happy because they will know the results faster unlike the old days when it took a lot of time

DET015 They would welcome it

DET016 people might receive it well and how they might be reached

DET017 This can make them happy because most people usually complain about long distance

DET018

DET019 I would be happy with it because transportation is hard and people are lazy because of that.

DET020 They can be happy with it because they do not have to travel a long distance just to get tested

DET021 it can make them happy because people in the village find the distance too much so if it is available in their local health Centre it would be easier

DET022 They would welcome it because everyone wants to know the status of their child

DET023 other people might receive it well because they would want to be protected from the virus.

DET024 No thoughts on this

DET025 They would like it and they would be motivated to go for the test

DET026 They would like it and they would be encouraged to come for the test

DET027 They would like it because they would not have to walk for a long distance now. You need to go through the village chief to reach out to a lot more people

DET028 no comment on this

DET029 They would like it and be encouraged to get tested. Reach us through conventions

DET030 **-** They would like it because the hospital is really close and they would not need to walk for a long distance to get tested

**`8. What are some concerns that you and some members in the community might have related to receiving HIV test results of a child?**

DET011 You get stressed and scared but when you get counselled you get motivated

DET012 I have no concerns because if found positive, the doctors would help

DET013 no comment

DET014 I would not have stress or fear but hope that my child will be helped

DET015 I would not be worried because if my child is found positive, we will get the needed help.

DET016 no comment on this

DET017 Fear comes because if found positive, the child’s life is on the line but if negative she will know how to care for the child

DET018

DET019 I have no concerns because we need to be helped and if I am found positive I would also not hesitate but get my children tested

DET020 I wouldn’t be worried because I will know my status

DET021 Concerns comes that if found positive, the life of my child may be in danger

DET022 **-** I do not have any concerns because I know we will be assisted

DET023 I would not be worried because I know I would be helped regardless of the result

DET024 I have concerns because I have never done this test for my child

DET025 **-** I would be worried because I am un sure of the results

DET026 I have no concerns because it is helping us the people.

DET027 Stress comes about because of fear of knowing the results

DET028 I get worried when I have not gone for any testing

DET029 I am worried of what the results will be

DET030 I do not have any worry because I know my child will get helped regardless of the results

**9. Do you have suggestions or ideas for addressing possible community concerns about these HIV testing strategies?**

DET011 We need to receive good counselling

DET012 Getting tested so that you should not have fear

DET013 I would accept it and if I was found to be negative, I would praise God.

DET014 My worries would end after hearing the results.

DET015 get tested so that you should not have doubts

DET016 if you have fear you would not gain anything

DET017 Avoiding discrimination of HIV positive people and give counselling on how to avoid the spread of the virus

DET018

DET019 Encouraging people not be stressed but just accept the results

DET020 I think when you are worried of the results you delay yourself in knowing the status of your child

DET021 we should not discriminate HIV infected children by playing with them and giving them advice so that they should not spread it.

DET022They need to be counselled that even if the child is positive, it is not the end of the world

DET023 I would not be worried because when you are found with the virus you get the requried help

DET024 Everyone has fear and the only way to get lid of it is knowing the results

DET025 We should just be strong because if found positive we would get assisted

DET026 Forming groups where we can discuss issues of HIV/AIDS

DET027 To reduce fear, people just need to get their children tested so they can know their status and accept it

DET028 Everyone has stress but knowing the results is the only help.

DET029 Be strong and if the child is positive learn how to take care of her

DET030 joining different groupings and sharing HIV related issues and performing plays/dramas so as to entertain and motivate the infected

B. Perceptions about time to receive test results

**10. From the time that your child is tested, how long would you be patient enough to know results from the blood tests? (Same day, after three, after three months?)**

**Tsiku Lomwelo □**

**Patatha masiku □**

**Miyezi iwiri kapena itatu □**

**Fotokozani zifukwa zomwe mwasankhira Yankho limeneli**

DET011 Same day, I think when you are worried of the results you delay yourself in knowing the status of your child

DET012 Three days, No reason

DET013 Three days, Because I would want to know if my child has the virus or not

DET014 Same day, **-** To know about the health of my child fast

DET015 After afew days, it just feels right

DET016 They would be the ones to tell me because I do not know the procedure

DET017 Same day, It is nice to know on the same day.

DET018

DET019 Same day, that if the child is found positive, we can get assisted immediately

DET020 Same day, because it is not easy to travel to the hospitaL

DET021 Same day, when you get the results on the same day it makes one happier.

DET022 Same day, Because I need to know how my child is and how to she can get assisted

DET023 Same day because I want my child to be assisted immediately

DET024 Same day if found positive, you need to take it in quickly and protect the child

DET025 Same day, Because I want to know and accept the results

DET026 Same day, Because I would not have worries on the same day results.

DET027 Same day, I need to hear them on the same day to prevent stress

DET028 Same day, if found positive, quickly accept it so you can protect the child.

DET029 Same day, Because they will only be comfortable after hearing the results

DET030 Same day, Because when you get your results immediately you do not become worried

11. **If your child is tested for HIV, how long would you want to wait before you are told that results from the tests are HIV positive? (same day, after three, after three months?)Explain why you would prefer your chosen answer.**

**Tsiku Lomwelo □**

**Patatha masiku □**

**Miyezi iwiri kapena itatu** □

Fotokozani zifukwa zomwe mwasankhira Yankho limeneli

DET011 Same day, you get counselling and help quickly.

DET012 Three day, you get counselling and help quickly.

DET013 Same day, I choose the same day because a child is a child and they might cause troubles.

DET014 Same day, So that my child can get assisted immediately

DET015 Three days, so that the hospital should have time to sort out the results

DET016 Same day, if found positive the child needs to be helped immediately

DET017 Same day, no reason for choosing same day.

DET018

DET019 2-3 months because it gives time for the doctor to verify the results

DET020 Same day, Because of transportation issues

DET021 Same day, no reason for picking the same day

DET022 Same day, Because I want to get the results before someone manipulates my mind

DET023 Same day, because I stay very far from the hospital

DET024 Same day, it is the day I am expecting the results of my child

DET025 Same day, I stay very far, so I want to know today

DET026 Same day, you need to know on the same day so u can receive help

DET027 Same day

DET028 Same day, I want to get results same day

DET029 Same day Because the test is taking place in the laboratory I think it cannot take a lot of time

DET030 Same day because my child need to get help at the same time too

12. **If your child test for HIV, how long would you want to wait before you are told that results from the test are HIV negative? (Same day, after three, after three months?)Explain why you would prefer your chosen answer.**

**Tsiku Lomwelo □**

**Patatha masiku □**

**Miyezi iwiri kapena itatu □**

**Fotokozani zifukwa zomwe mwasankhira Yankho limeneli**

DET011 Same day, no comment

DET012 Same day, To know the health status of the child

DET013 Same day, I have no idea

DET014 Same day, if it takes too long you would get tired of waiting

DET015 Same day, to know the HIV status of my child

DET016 Same day, Because the child needs to receive assistance fast, if required

DET017 Same day, Same day so you will know how to care for the child after results

DET018

DET019 Same day, I would prefer going home without any fear or hope

DET020 Same day, I would prefer going home without any fear or hope

DET021 Same day so that I should know how to care for my child

DET022

DET023 Same day,

DET024 Same day, **CG-** I need know if my child is positive or not

DET025 Same day. Considering transport issues same day results would be better

DET026 Same day because I would not be at ease if I was to wait longer

DET027 No thoughts on this

DET028 Same day Because I need to start thinking of what to do next

DET029 Same day Because I stay far and transportation is difficult

DET030 Same day, because when you have not heard the results your heart is not at ease

C.Acceptability and decision making

13. **What information would you want to be given to make an informed decision to accept that your child should get an HIV test or not? Explain**

DET011 you would get counselling at the hospital

DET012 no idea

DET013 Counselling should come from the hospital

DET014 we should be told when they are drawing the blood from the child and it will depend on you to help the child.

DET015 no idea

DET016 I would want to know the whole procedure

DET017 to know the actual truth about the child’s status

DET018

DET019 if you love your child you do not have to wait for the doctors to tell you what to do

DET020 Following the doctors counselling about the importance of testing

DET021 to know the actual truth about the child’s health

DET022 How to take care of a child if found positive or negative

DET023 **-** They should receive counselling of what to do if the child is found Positive

DET024 Being told the dangers of this disease

DET025 How to take care of child if positive or negative

DET026 Meeting the doctor to tell us the correct order to follow things.

DET027 **-**They should be taught the importance of HIV testing

DET028 being told the dangers of the virus

DET029 Teach them how they can take care of the child if found positive or negative

DET030 Meeting the doctor that he/she should give proper advise

14**. How would you want to be approached and given information about these two HIV testing strategies? Explain**

DET011 Using radios and giving us good counselling

DET012 following the doctor’s instructions

DET013 in every way possible even homes

DET014 When reached it would then be a personal decision whether to go for the test or not

DET015 Just following the hospital instructions

DET016 Setting up conventions

DET017 finding me in the village and telling me my HIV status

DET018

DET019 I choose the doctor reaching us in our respective villages and teaching us the strategies

DET020 Reaching them well so they understand the importance of Cepheid

DET021 finding them in their villages

DET022 When we come for antenatal clinic because you can not manage to reach us in our homes

DET023 no comment on this

DET024 At antenatal clinic or visiting us

DET025 Any means the medical team might use

DET026 by hosting conventions and using radios

DET027 listening to the hospital’s advice

DET028 Antenatal Clinic or at our home

DET029 How ever the hospital is comfortable with

DET030 Organising meetings and through the radio

D.Potential Social Harms/Concerns etc.

15. **Would you encourage other parents/guardians to allow their children to test for HIV using these two approaches?**

Yes □ No □

DET011 Yes

DET012 Yes

DET013 Yes

DET014 Yes

DET015 Yes

DET016 Yes

DET017 Yes

DET018

DET019 Yes

DET020 Yes

DET021 Yes

DET022 Yes

DET023 Yes

DET024 Yes

DET025 Yes

DET026 Yes

DET027 Yes

DET028 Yes

DET029 Yes

DET030 Yes

15b. **What would be your main concerns and worries towards these approaches?**

DET011 I feel that the blood taken from the vein is a lot and I am worried that my child might not have any more blood remaining in her

DET012 no problem here

DET013 no problem

DET014 no problem

DET015 I have no concerns

DET016 I think they are drawing too much blood so a child might get weak

DET017 I have no concerns

DET018

DET019 I have no problems with this because it is helpful to me and my family.

DET020 no problem with this because I would want to know the results of the child

DET021 no concerns

DET022 **-** I have not concern

DET023 has no problem with this

DET024 if mistakes are made the child might lose a lot of blood which strikes fear in our hearts

DET025 My concern is that when drawing blood, it might hurt my child

DET026 no problem with this issue.

DET027 no worries because its her child getting help

DET028 Losing blood in the process of blood draw

DET029 **-** I feel like the venous puncture would hurt my child

DET030 have no problem with it

**16. How would you personally feel is someone from your community learns about HIV test results for your child?**

DET011 I would personally feel good because I would know my child’s status

DET012 it can be difficult because someone who you are not related to can not keep a secret at least if it was a relative

DET013 I would feel good

DET014 I would feel good because they are protecting their child’s future

DET015 it is hard especially when they are not relatives

DET016 I can’t complain because I know how to take care of the child

DET017 I would not be happy because these results are confidential

DET018

DET019 I wouldn’t be very sad because this can happen to anyone at any time

DET020 **-** it would break my heart because my friends would laugh at my child.

DET021 I would not feel good because results are confidential

DET022 no problem because there is no reason to hide these days

DET023 I would not feel good because it is only supposed between the doctor and me.

DET024 it would be painful because everyone wants to keep this a secret

DET025 Wouldn’t like it seeing another person talking about my child’s HIV status

DET026 I would be sad because I would become a laughing stock

DET027 **I** would not be happy seeing someone preach about the results

DET028 it would be painful because everyone wants to keep their secrets

DET029 I would not like it because I do not want my childs status to be talked of by everyone

DET030 **-** I can not like that because of fear of discrimination

17. **Do you have any other thoughts you wish to share on this topic?**

DET011 My concern is on the vein which blood is taken from which is painful to a child

DET012 no problem with this

DET013 no problems

DET014 no comment

DET015 No more concerns

DET016 my concern is on the blood and how it is taken

DET017 No more concerns

DET018

DET019 see no problem because it is all for my child’s future

DET020 no thoughts on this, I have understood the importance of the test.

DET021 no concerns

DET022 No question, it should just continue so that it can reach others

DET023 I have not yet received my results from yesterdays test

DET024 no problem

DET25 I only worried because I do not know the status of my child.

DET026 I have no problem with this but rather joy so they should continue.

DET027 only joy brought about because of this new development

DET028 no objections on this

DET029 **-** I am only worried because I do not know the status of my child

DET030 I have no problem with it, I am just glad and wish that this method continues

*The Research Team*
