# Supplementary material for: ‘If I am on ART, my new-born baby should be put on treatment immediately’: Exploring the acceptability, and appropriateness of Cepheid Xpert HIV-1 Qual assay for early infant diagnosis of HIV in Malawi
Source: PLOS Glob Public Health. 2023 Mar 10;3(3):e0001135. doi: 10.1371/journal.pgph.0001135 (PMC10021387; doi:10.1371/journal.pgph.0001135)
Supplement: S2 File — (ZIP) [file pgph.0001135.s005.zip › transcripts responses chichewa& english/ANSWERS_ FOR _DET61- DET70.docx]

**HEALTH CARE WORKES**

1. **Why do caregivers have a lot of trust in hospital staff?**

DET061 because they help them in so many things hence they have faith that they can help out with anything.

DET062 because they know everything so they need to be trust them

DET063 because the hospital staff are the ones that help sick people so they trust them

DET064 because they are the ones that can help us with our problems

DET065 **-** I trust them depending on how they are performing their job

DET066 because when we come to the hospital, they welcome us and help us as required

DET067 because when they give us medication when we are sick, we get better

DET068 **-** Because they help us whenever we are sick

DET069 because they help them in so many things hence, they have faith that they can help out with anything.

DET070 because they are the ones that can help us with our problems

1. **Why is that most caregivers do not have anything to say when asked question?**

DET061 Because they do not want to prolong the discussion

DET062 No reason

DET063 some think that their comment will not be important or helpful

DET064 I usually have something to say but I don’t because of fear

DET065 some are just ashamed because of their status so they do know want to say anything

DET066 because they either have no idea or they didn’t understand the question

DET067 They either don’t know the answer or they are afraid of getting it wrong

DET068 Because of fear

DET069 because they do not want to prolong the discussion

DET070 **-** I usually have something to say but I don’t because of fear

1. **Why do mothers think their children should be tested if they themselves are HIV negative**?

DET061 They want to know the status of the child

DET062 they want to know the child’s status because children sometime play with sharp objects

DET063 that is how it is supposed to be because children might have contracted it somewhere without parents knowing

DET064 **-** A child might have contracted the virus from friends while playing

DET065 Maybe the mother could be in window period and transmit to the child and the child might also have contracted while playing

DET066 we just want to know they status of the child

DET067 because children sometimes would play with sharp objects that were unknowingly used by an infected person

DET068 that is how it is supposed to be because children might have contracted it somewhere without parents knowing

DET069 they want to know the status of the child

DET070 a child might have contracted the virus from friends while playing

1. **Do women understand the role of ART as the preventative measure if partners are HIV positive?**

DET061 She doesn’t have any idea.

DET062 they understand and they just need to protect themselves during sexual intercourse

DET063 this is new to me

DET064 I don’t have any idea

DET065 I don’t have any idea.

DET066 Yes we understand and follow it

DET067 Yes I understand

DET068 She doesn’t have any idea.

DET069 She doesn’t have any idea

DET070 She doesn’t have any idea.
