# Supplementary material for: ‘If I am on ART, my new-born baby should be put on treatment immediately’: Exploring the acceptability, and appropriateness of Cepheid Xpert HIV-1 Qual assay for early infant diagnosis of HIV in Malawi
Source: PLOS Glob Public Health. 2023 Mar 10;3(3):e0001135. doi: 10.1371/journal.pgph.0001135 (PMC10021387; doi:10.1371/journal.pgph.0001135)
Supplement: S2 File — (ZIP) [file pgph.0001135.s005.zip › transcripts responses chichewa& english/DET 0038.docx]

*A Questionnaire to validate new HIV tests called Cepheid Xpert HIV -1 Quay assay (Cepheid) in your hospital*

DET 0039

1. How would you as a parent/guardian feel if your child was to undergo HIV testing with Cepheid ?

Ine ndingamve bwino chifukwa ndikamva mwana mene alili ndizaziwa mene alili

CG- I would feel good when I know the results, I will know my child’s status

2. What are your thoughts about these new strategies for testing HIV in children and giving results promptly?

Ineyo ndikuona kuti njira imeneyi ndiyabwino chifukwa itithandizira kuziwa za nthupi la mwana

CG- I think this method is good because it will help us know the status of the child.

3. How should these approaches be implemented in a hospital? (Probe who should be targeted, why should they be targeted and why?)

-Pozera msokhano

CG - using conventions

-Tiyambire ana chifukwa njira zimenezi akulu alinazo kale

CG- Start with the children because the adults already know have their own methods

4. How should issues of privacy of both children and their guardians be maintained?

Makolo ndi madotoo akuyenera kusunga chinsisi

CG- Doctors and parents must keep it a secret

5a.What should be the role of parents/guardians in the implementations of these approaches?

Gawo limene tikatenge ndikutengapo gawo poyezesa magazi

CG- We can only take part by going for the test

b.What information should be provided to ensure that guardians understand the procedures involved?

Auzidwe masamalidwe ndi matengedwe amatenda amenewa

CG- Should be told prevention measures and how you can contract the virus

6. What should be the role of male partners in the implementation of these approaches? (Probe: How should male partners be encouraged to take active role in these approaches?)

-Azibambo akungoyenera kubwera kuzayezesa ndipo kuwalangiza ubwino wake wowayezetsa ana kuti akhale ndi umoyo wabwino

CG- Men should come for testing and also be told the importance of getting their children tested

7. How would your community feel if these approaches were to be implemented in your nearest health facility? (What could be done to encourage community members to participate in these interventions?)

-Ena atha kuchimva koma ena sangachimve

CG -Some can accept it but others can not

-pakuyenera kuwauza afumu kuti apange msonkhano wokhuzana ndi njirazi

CG- Telling the village chief to hold a convention concerning this.

8. What are some concerns that you and some members in the community might have related to receiving HIV test results of a child?

Nkhawa imakhalapo kuti chifukwa suziwa mene mwana alili nthupi

CG- Concerns and fear comes because we do not know our child’s status

9. Do you have suggestions or ideas for addressing possible community concerns about these HIV testing strategies?

Madakotala apelike malangiso athesa nkhawa akamadikira Zotsatira

CG- Doctors should counsel people while waiting for the results

B. Perceptions about time to receive test results

10. From the time that your child is tested, how long would you be patient enough to know results from the blood tests? (Same day, after three, after three months?)

Tsiku Lomwelo □

Patatha masiku □

Miyezi iwiri kapena itatu □

Fotokozani zifukwa zomwe mwasankhira Yankho limeneli

Kuti uziwe mene thupi mulili ndikuziwa chomwe ungachite malingana ndizotsatilazo

CG- To know what you should do depending on the results.

11. If your child is tested for HIV, how long would you want to wait before you are told that results from the tests are HIV positive? (same day, after three, after three months?)Explain why you would prefer your chosen answer.

Tsiku Lomwelo □

Patatha masiku □

Miyezi iwiri kapena itatu □

Fotokozani zifukwa zomwe mwasankhira Yankho limeneli

Ndi masiku abwino kuti ukamve zenizeni za mene mwana alili

CG- It’s a good amount of days to hear the real truth about how a child is

12. If your child test for HIV, how long would you want to wait before you are told that results from the test are HIV negative? (Same day, after three, after three months?)Explain why you would prefer your chosen answer.

Tsiku Lomwelo □

Patatha masiku □

Miyezi iwiri kapena itatu □

Fotokozani zifukwa zomwe mwasankhira Yankho limeneli

Kuti ulimbe mtima ndi zosatilazo

CG- So that you should reach acceptance level

C.Acceptability and decision making

13. What information would you want to be given to make an informed decision to accept that your child should get an HIV test or not? Explain

Alandire uphungu wachilimbikiso Kamba ka zotsatila

CG- Should receive motivating counselling about the result

14. How would you want to be approached and given information about these two HIV testing strategies? Explain

Akabwera uphungu wachilimbikiso Kamba ka zotsatila

CG- Motivating counselling towards the results when they come

D.Potential Social Harms/Concerns etc.

15. Would you encourage other parents/guardians to allow their children to test for HIV using these two approaches? What would be your main concerns and worries towards these approaches?

Yes □ No □

Nkhawa palibepo ndimayezedwawa

CG- No problems with this

16. How would you personally feel is someone from your community learns about HIV test results for your child?

Sangadandaule chifukwa mwana wawo akhala wathandizidwa

CG- I would not worry because at least my child has been helped

17. Do you have any other thoughts you wish to share on this topic?

Alibe maganizo kapena nkhawa inailiyonse yokhuza mayezedwe a Cephid

CG- No other thoughts or concerns about Cepheid

*The Research Team*

Participant 0038 akuti akumva bwino chifukwa cha njilazi zomwe zithandizire kuziwa mene mwana wawo alili komanso anali omasuka mukuthandiza kuyankha mafunso onse omwe anali muchipatala chakafukufuku ameneyu

She says she is happy because if this method that will help in knowing the status of her child. She was very comfortable in taking part in this interview
