# Supplementary material for: ‘If I am on ART, my new-born baby should be put on treatment immediately’: Exploring the acceptability, and appropriateness of Cepheid Xpert HIV-1 Qual assay for early infant diagnosis of HIV in Malawi
Source: PLOS Glob Public Health. 2023 Mar 10;3(3):e0001135. doi: 10.1371/journal.pgph.0001135 (PMC10021387; doi:10.1371/journal.pgph.0001135)
Supplement: S2 File — (ZIP) [file pgph.0001135.s005.zip › transcripts responses chichewa& english/DET 0039.docx]

*A Questionnaire to validate new HIV tests called Cepheid Xpert HIV -1 Quay assay (Cepheid) in your hospital*

DET 0039

1. How would you as a parent/guardian feel if your child was to undergo HIV testing with Cepheid ?

Ine ndingamve bwino chifukwa ndikamva mwana mene alili ndizaziwa mene alili

CG- I would feel good because I will know the health status of my child

2. What are your thoughts about these new strategies for testing HIV in children and giving results promptly?

Ineyo ndikuona kuti njira imeneyi ndiyabwino chifukwa itithandizira kuziwa za nthupi la mwana

CG- It is a good method because it will help us know status of our children

3. How should these approaches be implemented in a hospital? (Probe who should be targeted, why should they be targeted and why?)

-Pozera msokhano

CG- Using conventions

-Tiyambire ana chifukwa njira zimenezi akulu alinazo kale

CG- It should start with the children

4. How should issues of privacy of both children and their guardians be maintained?

Makolo ndi madotoo akuyenera kusunga chinsisi

CG- Parents and doctors need to keep the secret

5a.What should be the role of parents/guardians in the implementations of these approaches?

Gawo limene tikatenge ndikutengapo gawo poyezesa magazi

CG- Taking part by getting testet

b.What information should be provided to ensure that guardians understand the procedures involved?

Auzidwe masamalidwe ndi matengedwe amatenda amenewa

CG- They should be told how to prevent and how one can contract the virus

6. What should be the role of male partners in the implementation of these approaches? (Probe: How should male partners be encouraged to take active role in these approaches?)

-Azibambo akungoyenera kubwera kuzayezesa ndipo kuwalangiza ubwino wake wowayezetsa ana kuti akhale ndi umoyo wabwino

CG- Men should come get tested and be told the importance of getting children tested for a better life.

7. How would your community feel if these approaches were to be implemented in your nearest health facility? (What could be done to encourage community members to participate in these interventions?)

-Anthu akhoza kuchilandira bwino

CG- People can welcome this development

-Tikungoyenera kuwalangiza zaubwino woyezetsa magazi

CG- They need to be counselled on the importance of the test

8. What are some concerns that you and some members in the community might have related to receiving HIV test results of a child?

Nkhawa zimakhalapo pakuti mwina mwana wapezeka nako anthu amakuseka

CG- Concerns come for the reason that people might laugh at an HIV positive child.

9. Do you have suggestions or ideas for addressing possible community concerns about these HIV testing strategies?

Tikuyenera kutenga gawo pomusungira chinsisi kuti akamasewera ndi amzake asamasekedwe

CG- We need to take initiative by keeping our child’s secret so that he/she shouldn’t be laughed at when playing.

B. Perceptions about time to receive test results

10. From the time that your child is tested, how long would you be patient enough to know results from the blood tests? (Same day, after three, after three months?)

Tsiku Lomwelo □

Patatha masiku □

Miyezi iwiri kapena itatu □

Fotokozani zifukwa zomwe mwasankhira Yankho limeneli

Ndasankha siku lomwelo chifukwa ndiziwa momusamalira

CG- Same daybecause I need to know immediately how to take care of my child

11. If your child is tested for HIV, how long would you want to wait before you are told that results from the tests are HIV positive? (same day, after three, after three months?)Explain why you would prefer your chosen answer.

Tsiku Lomwelo □

Patatha masiku □

Miyezi iwiri kapena itatu □

Fotokozani zifukwa zomwe mwasankhira Yankho limeneli

Malingana ndikafukufukuyo kuti aziwe bwino ngati ali nako kapena ayi

CG- According to the research protocol

12. If your child test for HIV, how long would you want to wait before you are told that results from the test are HIV negative? (Same day, after three, after three months?)Explain why you would prefer your chosen answer.

Tsiku Lomwelo □

Patatha masiku □

Miyezi iwiri kapena itatu □

Fotokozani zifukwa zomwe mwasankhira Yankho limeneli

Chifukwa choti pamakhala ma process ambiri kuti ma results atuluke

CG- Because I know that for results to come out there are so many processes that happen

C.Acceptability and decision making

13. What information would you want to be given to make an informed decision to accept that your child should get an HIV test or not? Explain

Ndingafune mundilangize masamalidwe ndi kapewedwe kamatendawa

CG- I would like to be counselled on prevention measures

14. How would you want to be approached and given information about these two HIV testing strategies? Explain

Mukhoza kutipeza m’mudzi kapena pa phone

CG- In our local communities or mobile phone

D.Potential Social Harms/Concerns etc.

15. Would you encourage other parents/guardians to allow their children to test for HIV using these two approaches? What would be your main concerns and worries towards these approaches?

Yes □ No □

Kwa ine nkhawa sindingakhale nayo chifukwa choti ndikufuna kuteteza mwana

CG- I have no concerns because I just need to protect my child

16. How would you personally feel is someone from your community learns about HIV test results for your child?

Sindingamve bwino chifukwa choti zinthuzi zimayenela kukhala mwa chinsinsi

I wouldn’t feel good because these things need to be kept private

17. Do you have any other thoughts you wish to share on this topic?

Ine nkhawa ndilibe kwanga ndikusangalala kuti njirazi zikufuna zikhazikidwe

CG- No concerns

*The Research Team*
