# Supplementary material for: ‘If I am on ART, my new-born baby should be put on treatment immediately’: Exploring the acceptability, and appropriateness of Cepheid Xpert HIV-1 Qual assay for early infant diagnosis of HIV in Malawi
Source: PLOS Glob Public Health. 2023 Mar 10;3(3):e0001135. doi: 10.1371/journal.pgph.0001135 (PMC10021387; doi:10.1371/journal.pgph.0001135)
Supplement: S2 File — (ZIP) [file pgph.0001135.s005.zip › transcripts responses chichewa& english/DET 0043.docx]

*A Questionnaire to validate new HIV tests called Cepheid Xpert HIV -1 Quay assay (Cepheid) in your hospital*

DET 0043

1. How would you as a parent/guardian feel if your child was to undergo HIV testing with Cepheid

Atha kukhala osangala chifukwa afuna kuziwa nthupi mwa mwana kuti muli bwanji

CG- I would be happy knowing my child’s status

2. What are your thoughts about these new strategies for testing HIV in children and giving results promptly?

Maganizo awo ndikukondwa ndi njilazi kuti alandila chithandizo malingana ndi ma results

CG- I am pleased because I will help my child according to the results

3. How should these approaches be implemented in a hospital? (Probe who should be targeted, why should they be targeted and why?)

Tizikhazikitse powuzana kuti tikayezetese komanso tiyambile kuyeza ana chifukwa mwana sangathe kupanga chisankho paiye yekha kuti akayezetse

CG- We should establish this by telling each other to get tested and we should start with children because they cannot make a choice on their own.

4. How should issues of privacy of both children and their guardians be maintained?

A dotolo ndi amene akuyenzetsayo ndi amene akuyenela kusunga chinsinsi

CG- The doctor is the one who is supposed to keep a secret

5a.What should be the role of parents/guardians in the implementations of these approaches?

Makolo atenge ana awo ndikukayezetsa

CG- Parents should take their children for testing

b.What information should be provided to ensure that guardians understand the procedures involved?

A chipata adziwe kunika kwa Cepheid

CG- Assurance that the hospital stuff knows how to perform the test

6. What should be the role of male partners in the implementation of these approaches? (Probe: How should male partners be encouraged to take active role in these approaches?)

Awuzidwe kuti nawonso akayezetse pakubwela nawo ku chipatala kuno

CG- Should be told so that they can be tested too.

7. How would your community feel if these approaches were to be implemented in your nearest health facility? (What could be done to encourage community members to participate in these interventions?)

Atha kukhala osangalala chifukwa zawafupikila kusiyana mtunda wa utali kwambiri komanso a chipatala ndi a mfumu akuyenela kulimbitsa anthu mtima kuti akayezetse

CG- I would be happy because it is close by rather than the usual long distance and the village headman should also encourage his people to get tested

8. What are some concerns that you and some members in the community might have related to receiving HIV test results of a child?

Ena amadandawula kuti ukapezeka ndi H.I.V uzimwa ma ARV moyo wako wonse.

CG- Some are concerned about the fact that if found with HIV you will be taking ARVs for the rest of your life

9. Do you have suggestions or ideas for addressing possible community concerns about these HIV testing strategies?

Kuwalangiza kuti asamadandawule chifukwa ayamba kulandila chithandizo akapezeka ndi H.I.V

CG- Advising them that they shouldn’t be stressed out if found positive cause they will receive medications.

B. Perceptions about time to receive test results

10. From the time that your child is tested, how long would you be patient enough to know results from the blood tests? (Same day, after three, after three months?)

Tsiku Lomwelo □

Patatha masiku □

Miyezi iwiri kapena itatu □

Fotokozani zifukwa zomwe mwasankhira Yankho limeneli

Kuti aziwe m’mene thupi mwa mwana mulili

CG- To know the child’s status

11. If your child is tested for HIV, how long would you want to wait before you are told that results from the tests are HIV positive? (same day, after three, after three months?)Explain why you would prefer your chosen answer.

Tsiku Lomwelo □

Patatha masiku □

Miyezi iwiri kapena itatu □

Fotokozani zifukwa zomwe mwasankhira Yankho limeneli

Chifukwa kwawo ndikutali kuti abwele kuzangomva zosatila

CG- Because I stay very far from the hospital.

12. If your child test for HIV, how long would you want to wait before you are told that results from the test are HIV negative? (Same day, after three, after three months?)Explain why you would prefer your chosen answer.

Tsiku Lomwelo □

Patatha masiku □

Miyezi iwiri kapena itatu □

Fotokozani zifukwa zomwe mwasankhira Yankho limeneli

Chifukwa kamatha kubisala ka chilombo nde Patatha Miyezi itatu

CG- Because sometimes the virus might hide so it is better 3 months

C.Acceptability and decision making

13. What information would you want to be given to make an informed decision to accept that your child should get an HIV test or not? Explain

Awuzidwe zowalimbitsa mtima kuti ngati mwana alibe H.I.V amusamale komanso ngati ali ndi H.I.V ayamba kulandila chithandizo

CG- Should be encouraged that if the child is found negative he/she will be cared for and if found positive should start medication

14. How would you want to be approached and given information about these two HIV testing strategies? Explain

Akabwela ku chipatala afotokozeledwe za njilazi

CG- During her visit to the hospital

D.Potential Social Harms/Concerns etc.

15. Would you encourage other parents/guardians to allow their children to test for HIV using these two approaches? What would be your main concerns and worries towards these approaches?

Yes □ No □

Alibepo Nkhawa ina iliyonse

No concerns

16. How would you personally feel is someone from your community learns about HIV test results for your child?

Atha kukhala okhumudwa chifukwa iwowo monga mokhala kholo akuyenela kuziwa zotsatilazo

CG- I would be sad because a parent is the only one who is supposed to know.

17. Do you have any other thoughts you wish to share on this topic?

Alibepo Nkhawa ina iliyonse koma maganizo awo ndiwoti njilazi zipitilile

CG- No problem with this but I hope that it should just continue

*The Research Team*
