# Supplementary material for: ‘If I am on ART, my new-born baby should be put on treatment immediately’: Exploring the acceptability, and appropriateness of Cepheid Xpert HIV-1 Qual assay for early infant diagnosis of HIV in Malawi
Source: PLOS Glob Public Health. 2023 Mar 10;3(3):e0001135. doi: 10.1371/journal.pgph.0001135 (PMC10021387; doi:10.1371/journal.pgph.0001135)
Supplement: S2 File — (ZIP) [file pgph.0001135.s005.zip › transcripts responses chichewa& english/DET001.docx]

**DET001_CG_F_24.7.18**

1. **Malingana ndi mmene tafotokozera za kayezedwe ka Cepheid, mwana ayenera kutengedwa magazi pachara kapena pa nsempha, inu monga kholo mungamve bwanji kuti mwana wanu ayezedwe magazi kuzera njira zimezi?**

**According to the explanation on how we test for Cepheid ,the child**

- **CG-** Atha kumva bwino popeza akufuna kumva zotsatira
- **CG-**  They would feel okay because they would know the results.

1. **Kwainu monga kholo la mwana wa chichepere, maganizo anu ndi otani pokhuzana ndi mayezedwe a magazi kuti tidziwe kuti mwana ali ndi HIV kapena ayi malingana ndi mmene tafotokozera za kayezedwe ka Cepheid kuti zosatira zimatuluka kwa minitsi 92?**

- **CG-**  Ndimaganizo abwino chifukwa kuzera munjirazi utha kuziwa kuti mwana ali bwanji mthupi ndikuziwa momusamalira ngati atapezeka nako komanso ngati atapezeka alibe tithe kuziwa momusamalira kuti asatengere
- **CG-** It’s a good idea because using this method we can know the status of the child and how we can take care of him/her if found infected and if found negative how we can prevent

1. **Kodi njira zimenezi tingazikhazikise bwanji mu zipatala? (tatiwuzani, tiyambe ndi gulu liti la anthu ndipo nchifukwa chani mukuganiza kuti tiyambe ndi gulu limeneli chifukwa chain?**

- **CG-** Aliyense ali ndi mwana akuyenera kutengapo gawo . Makamaka ndipofunika kuyambira ana chifukwa sangwanise kupita yekha kukayezesa magazi.
- **CG-** Everyone with a child should take part. Starting with children because they cannot manage to go get tested on their own

1. **Kodi tingapange bwanji kuti kuyezesa magazi kwa ana ndi makolo awo kapena anthu owayang’ira zikhale za chinsinsi?**

- **ANS-** Akuyenera kuziwa ndi makolo a mwana ndi a dokotala kuti zikhale za chinsinsi
- ANS- Only the parent of the child and doctor must know to keep it secretive

1. **Kodi makolo angatengepo gawo lanji kuti njira zoyezesera magazi za Cepheid zikhazikisidwe mu chipatala chathu chino cha Mulanje?**

- **CG-**  Akuyenera ku chimvesesa ndi kuchirandila msanga potenga gawo pofalisa uthengawu mmidzi
- **CG-** They need to understand it and take part in spreading the message in villages

b). **Kodi makolo awuzidwe zotani ndi uphungu wotani kuti amvesese za njira zoyezesera magazi za Cepheid ndi ?**

- **CG-** Kuzera mmisonkhano mmudzi ndi ma wailesi
- **CG-** During village conventions and radios.

1. **Kodi azibambo angatengepo gawo lanji kuti njira zoyezesera magazi za Cepheid ndi zikhazikisidwe mu chipatala chathu chino cha Mulanje? Tingawalimbikise bwanji azibambo kuti azitenga nawo gawo mukuyezedwa magazi mu njira za Cepheid?**

- **CG-**  Akuyenera kutengapo gawo chifukwa nawonso ndi kholo, osangoti poti mayi ndamene amakonda kulera mwani ayi.
- **CG-** They also need to take part because the child is also theirs and not just leaving he responsibility of taking care of the child to the mother

1. **Kodi anthu a mmudzi mwanu angamve bwanji njira zoyezesera magazi za Cepheid zitakhazikisidwa pa chipatala chanu chaching’ono mmudzi mwanu. Tingatani kuti anthu a mmudzi muno alimbikisidwe kutenga nawo mbali mu njira zoyezetsera magazi za Cepheid?**

- **CG-** Zitha kukhala zabwino chifukwa anthu aku ma midzi sakonda kuyezetsa magazi chifukwa chipatala chimakhala kutali koma zitatero zitha kuthandiza
- **CG-** it would be good because many village people are less likely to get tested because hospitals are usually far but if it were to happen like that it would be helpful.

1. **Kodi inu ndi anthu ena mma midzi mu mumakhala ndi nkhwa zanji zokhuzana ndi kulandila zosatira za magazi mwana akayezedwa kuti tiziwe kuti mwana ali ndi HIV kapena ayi?**

- **CG-** Nkhawa imakhala poti mwana akule ndi thanzi apange bwanji akapezeka ndi ka chilombo
- **CG-** The fear comes from how the child will be raised healthy if found with the virus.

1. **Kodi mungakhale ndi njira kapena maganizo a momwe tingathandizire kuchepesa nkhawa zokhuzana ndikulandila zotsatira za magazi mwana wayezedwa kuti tidziwe kuti mwana ali ndi HIV kapena ayi?**

- **CG-** Makolo amafunika kulimba mtima kuti zachitika kuti athe kumulangizaso mwana zabwino
- **CG-** Parents need to be strong so they can counsel the child

1. **Kuchokera pa nthawi yomwe mwana wanu wayezedwa magazi kuti tidziwe kuti mwana ali ndi HIV kapena ayi, mungapilile nthawi yayitali bwanji kuti mudziwe zosatira**

**Tsiku lomwelo**

**Patatha masiku**

**Miyezi iwiri kapena itatu**

**Fotokozani zifukwa zomwe mungasankhile yankho limeneli**

- **CG-** Akabwera amayenera kumva tsiku lomwelo chifukwa akabwera amakhala ndi nkhawa kuti zosatira zikhala zotani.
- **CG-** when they come for testing they need to hear the results on the same day because they have worries about the possible results.

1. **Mwana wanu atayezedwa magazi, mungafune kudikila nthawi yayitali bwanji kuti mudziwe kuti mwana ali ndi HIV yomwe yimayambitsa matenda a AIDS?**

**TSiku lomwelo**

**Patatha masiku**

**Miyezi iwiri kapena itatu**

**Fotokozani zifukwa zimene mwasankhila yankho limenelo**

- **CG-** Chifukwa mayendwe ndi ovuta kwa ife anthu a mmidzi ndekuti tizachoite kubweranso kuzamva zosatira pakhoza kutenga nthawi
- **CG-** Because of transport problems for us village people it would take a lot of time to come back and hear the results.

1. **Mwana wanu atayezedwa magazi mungafune kudikila nthaawi yayitali bwanji kuti muziwe kuti mwana alibe HIV yomwe imayambitsa matenda a AIDS**

- **Same day**

**Patatha masiku**

**Miyezi iwiri kapena itatu**

**Fotokozani zifukwa zomwe mungasankhile yankho limenelo**

1. **kodi mungafune muwuzidwe zotani ndi uphungu otani kuti inu mupange chisankho choti mwana wanu ayezedwe magazi kuti mudziwe kuti mwana ali ndi HIV yomwe imayambitsa matenda a AIDS kapena ayi? Fotokozani bwino lomwe.**

- **CG-** Kuwalimbikisa kufunika kwa ubwino oyezetsa magazi
- **CG-** Helping them understand the importance of blood testing

1. **Mungafune kuti tikufikileni mu njira yotani kuti tikuwuzeni zimezi ndikukupasani uphungu umenewu wa njira zoyezesera magazi za Cepheid?**

- **CG-** Polemba ma posita ndikumata muzipatala kuti uthengawu upite patali.
- **CG-** Using posters to spread the message.

1. **Kodi mungathe kuwalimbikisa makolo anzanu kapena owasamalira ana kuti alore ana Awo ayezedwwe magazi kuti aziwe ngati ali ndi HIV yoyambitsa matenda a AIDS kugwilitsa ntchito Cepheid?**

- **CG-**  Eya
- **CG-** Yes

**15b) Nkhawa zanu zingakhale zotani ndi mayezedwe amenewa a Cepheid?**

- **CG-**  Pomutenga mwana magazi pa nsempha atha kumamva kupweteka kwambiri kamba ka njirazi.
- **CG-** A child might feel pain during venous blood draw

1. **Kodi mungamve bwanji ngati munthu wina wa mmudzi mwanu ataziwa zotsatira za magazi a mwana wanu atayezedwa kufufuza ngati ali ndi HIV kapena ayi?**

- **CG-** Nditha kumva bwino chifukwa masiku ano nkhani za HIV sizobisa
- **CG-** I would be okay with it because the issues about HIV now are too private

1. **Kodi muli ndi maganizo kapena nkhawa zina zomwe mungafune kutidziwisa pa nkhani imeneyi**

- **CG-** Nkhwawa ikumakhalapo kuti magazi ochuluka akumakatani nawo akatengedwa.
- **CG-** Questions arise with what is being done with the large amount of blood taken.
