# Supplementary material for: ‘If I am on ART, my new-born baby should be put on treatment immediately’: Exploring the acceptability, and appropriateness of Cepheid Xpert HIV-1 Qual assay for early infant diagnosis of HIV in Malawi
Source: PLOS Glob Public Health. 2023 Mar 10;3(3):e0001135. doi: 10.1371/journal.pgph.0001135 (PMC10021387; doi:10.1371/journal.pgph.0001135)
Supplement: S2 File — (ZIP) [file pgph.0001135.s005.zip › transcripts responses chichewa& english/DET002.docx]

**DET002_CG_F_24.7.1**

1. **Malingana ndi mmene tafotokozera za kayezedwe ka Cepheid, mwana ayenera kutengedwa magazi pachara kapena pa nsempha, inu monga kholo mungamve bwanji kuti mwana wanu ayezedwe magazi kuzera njira zimezi?**

- **CG-**  Ndingamve bwino chifukwa ndidziwa mmene mnthupi mwa mwana muliri.
- **CG-** I would feel like it because I would know the status of my child.

1. **Kwainu monga kholo la mwana wa chichepere, maganizo anu ndi otani pokhuzana ndi mayezedwe a magazi kuti tidziwe kuti mwana ali ndi HIV kapena ayi malingana ndi mmene tafotokozera za kayezedwe ka Cepheid kuti zosatira zimatuluka 92 ya Cepheid?**

- **CG-**  Maganizo anga omwe ndinali nawo ndiokuti ndiziwe zotsatila za mwana wanga lero lomwe
- **CG-**  My thoughts are that I need to know the results the same day

1. **Kodi njira zimenezi tingazikhazikise bwanji mu zipatala? (tatiwuzani, tiyambe ndi gulu liti la anthu ndipo nchifukwa chani mukuganiza kuti tiyambe ndi gulu limeneli chifukwa chain?**

- **CG-** Kuwuza anthu mmudzi komanso penapaliponse. Tikuyenera kuyambila gulu la ana chifukwa ndizothandiza kuteteza mwana wathu munthawi yake.
- **CG-** Telling the community people and everyone. We need to start with children because it is helpful to protect our child as early as possible.

1. **Kodi tingapange bwanji kuti kuyezesa magazi kwa ana ndi makolo awo kapena anthu owayang’ira zikhale za chinsinsi?**

- **CG-**  Chinsinsi ndiwekha kotero tikuyenera kusunga chinsinsi patokha.
- **CG-** only one who can keep your secret is you thereby we need to keep it to ourselves.

1. **Kodi makolo angatengepo gawo lanji kuti njira zoyezesera magazi za Cepheid zikhazikisidwe mu chipatala chathu chino cha Mulanje?**

- **CG-** Kuwafotokozera anzathu za upangiri umenewu kapena kuti njira zimenezi.
- **CG-** Explaining the process to our friends

b). **Kodi makolo awuzidwe zotani ndi uphungu wotani kuti amvesese za njira zoyezesera magazi za Cepheid?**

- **CG-** Mwina mulimonse mungandiwuzire ndimvera inuyo chifukwa inu ndi amene mumapeleka malangizo
- **CG-** Any way you can advise because you are the ones who gives proper counseling

1. **Kodi azibambo angatengepo gawo lanji kuti njira zoyezesera magazi za Cepheid ndi zikhazikisidwe mu chipatala chathu chino cha Mulanje? Tingawalimbikise bwanji azibambo kuti azitenga nawo gawo mukuyezedwa magazi mu njira za Cepheid?**

- **CG-**  Tikuyenera kukhala nawo pansi ndikuwafotokozera , kuwalimbikitsa kwake ndikuwawuza kuti atithandize kupewa matendawa.
- **CG-** We need to explain it to them, helping them by telling them how they can prevent the disease

1. **Kodi anthu a mmudzi mwanu angamve bwanji njira zoyezesera magazi za Cepheid zitakhazikisidwa pa chipatala chanu chaching’ono mmudzi mwanu. Tingatani kuti anthu a mmudzi muno alimbikisidwe kutenga nawo mbali mu njira zoyezetsera magazi za Cepheid?**

- **CG-** Akhonza kumva bwino chifukwa aliyense amafuna kumva zotsatira zake.
- **CG-** They would like it because everyone wants to know their results.

1. **Kodi inu ndi anthu ena mma midzi mu mumakhala ndi nkhwa zanji zokhuzana ndi kulandila zosatira za magazi mwana akayezedwa kuti tiziwe kuti mwana ali ndi HIV kapena ayi?**

- **CG-** Nkhawa sitikhala nayo chifukwa mwana amakhala wathandizika poti ife taziwa m’mene aliri ndipo tikhoza kuziwa momutetezera.
- **CG-** we have no fears because the child is helped in a sense that we know his status and how we can protect him/her

1. **Kodi mungakhale ndi njira kapena maganizo a momwe tingathandizire kuchepesa nkhawa zokhuzana ndikulandila zotsatira za magazi mwana wayezedwa kuti tidziwe kuti mwana ali ndi HIV kapena ayi?**

- **CG-** Maganizo ndilibe
- **CG-** No comment on this

1. **Kuchokera pa nthawi yomwe mwana wanu wayezedwa magazi kuti tidziwe kuti mwana ali ndi HIV kapena ayi, mungapilile nthawi yayitali bwanji kuti mudziwe zosatira**

**Tsiku lomwelo**

**Patatha masiku**

**Miyezi iwiri kapena itatu**

**Fotokozani zifukwa zomwe mungasankhile yankho limeneli**

- **CG-** Chifukwa ndifune ndimve zotsatira kuti ndikamapita ndikhale ndi chilimbikitso.
- **CG-** So that I know the results and when going I should have confidence

1. **Mwana wanu atayezedwa magazi, mungafune kudikila nthawi yayitali bwanji kuti mudziwe kuti mwana ali ndi HIV yomwe yimayambitsa matenda a AIDS?**

**Same day**

**Patatha masiku**

**Miyezi iwiri kapena itatu**

**Fotokozani zifukwa zimene mwasankhila yankho limenelo**

- **CG-**  Ndasankha kumva lero lomwe chifukwa ndizimene ndimayembekezera.
- **CG-** I choose to hear the same day because it is what I was expecting.

1. **Mwana wanu atayezedwa magazi mungafune kudikila nthaawi yayitali bwanji kuti muziwe kuti mwana alibe HIV yomwe imayambitsa matenda a AIDS**

**Tsiku lomwelo**

**Patatha masiku**

**Miyezi iwiri kapena itatu**

**Fotokozani zifukwa zomwe mungasankhile yankho limenelo**

- **CG-**  Ndiwudindo wa aliyense kumva zotsatira zake mwachanga ndikuyamba kuziteteza mosachedwa.
- **CG-** it is the duty of everyone to know the results as soon as possible to know how they will protect themselves.

1. **kodi mungafune muwuzidwe zotani ndi uphungu otani kuti inu mupange chisankho choti mwana wanu ayezedwe magazi kuti mudziwe kuti mwana ali ndi HIV yomwe imayambitsa matenda a AIDS kapena ayi? Fotokozani bwino lomwe.**

- **CG-** Ndilibe ganizo lililonse.
- **CG-** no thoughts on this

1. **Mungafune kuti tikufikileni mu njira yotani kuti tikuwuzeni zimezi ndikukupasani uphungu umenewu wa njira zoyezesera magazi za Cepheid?**

- **CG-**  Ndilibe ganizo lililonse.
- **CG-** no thoughts on this

1. **Kodi mungathe kuwalimbikisa makolo anzanu kapena owasamalira ana kuti alore ana Awo ayezedwwe magazi kuti aziwe ngati ali ndi HIV yoyambitsa matenda a AIDS kugwilitsa ntchito Cepheid?**

- **CG-**  Eya
- **CG-** yes

**15b) Nkhawa zanu zingakhale zotani ndi mayezedwe amenewa a ndi Cepheid?**

- **CG-** Sindingakhale ndi nkhawa ili yonse chifukwa ndikhara kuti ndamva zotsatira za mwana wanga.
- **CG-** I cannot have any problems with it because I’d have known the results of my child.

1. **Kodi mungamve bwanji ngati munthu wina wa mmudzi mwanu ataziwa zotsatira za magazi a mwana wanu atayezedwa kufufuza ngati ali ndi HIV kapena ayi?**

- **CG-** Ndimva bwino chifukwa choti zokhumba zamoyo wake zakwanilitsidwa.
- **CG-** I would be okay with it because their desire has been fulfilled

1. **Kodi muli ndi maganizo kapena nkhawa zina zomwe mungafune kutidziwisa pa nkhani imeneyi**

- **CG-**  Ndilibe nkhawa ili yonse.
- **CG-** I have no problem with this
