# Supplementary material for: ‘If I am on ART, my new-born baby should be put on treatment immediately’: Exploring the acceptability, and appropriateness of Cepheid Xpert HIV-1 Qual assay for early infant diagnosis of HIV in Malawi
Source: PLOS Glob Public Health. 2023 Mar 10;3(3):e0001135. doi: 10.1371/journal.pgph.0001135 (PMC10021387; doi:10.1371/journal.pgph.0001135)
Supplement: S2 File — (ZIP) [file pgph.0001135.s005.zip › transcripts responses chichewa& english/DET003.docx]

**DET003_CG_F_24.7.18**

1. **Malingana ndi mmene tafotokozera za kayezedwe ka Cepheid, mwana ayenera kutengedwa magazi pachara kapena pa nsempha, inu monga kholo mungamve bwanji kuti mwana wanu ayezedwe magazi kuzera njira zimezi?**

- **CG-** Ati akugwirizana nazo chifukwa zotsatira amva tsiku lomwelo osadikila nthawi yayitali.
- **CG-** I support this because I would hear the results on the same day

1. **Kwainu monga kholo la mwana wa chichepere, maganizo anu ndi otani pokhuzana ndi mayezedwe a magazi kuti tidziwe kuti mwana ali ndi HIV kapena ayi malingana ndi mmene tafotokozera za kayezedwe ka Cepheid ndi kuti zosatira zimatuluka kwa minitsi 92?**

- **CG-**  Ndi abwino chikfukwa ukamva zosatira tsiku lomwero osadikila nthawi.
- **CG-** its good because you hear the results on the same day.

1. **Kodi njira zimenezi tingazikhazikise bwanji mu zipatala? (tatiwuzani, tiyambe ndi gulu liti la anthu ndipo nchifukwa chani mukuganiza kuti tiyambe ndi gulu limeneli chifukwa chain?**

- **CG-** Ziyambike ndi ana chifukwa ana pametenga nthawi yaitali kuti aziwe mene alili pomwe akuluakulu amayezedwayezedwa.
- **CG-**It should start with children because it takes time to know their status while the older people get tested regularly

1. **Kodi tingapange bwanji kuti kuyezesa magazi kwa ana ndi makolo awo kapena anthu owayang’ira zikhale za chinsinsi?**

- **CG-** Kholo likuyenera kusunga chinsinsi cha mwana chifukwa mwana amakhala sanayambe kuyankhula.
- **CG-** a parent should keep the child’s privacy because the child has not started talking

1. **Kodi makolo angatengepo gawo lanji kuti njira zoyezesera magazi za Cepheid zikhazikisidwe mu chipatala chathu chino cha Mulanje?**

- **CG-** Makolo azivomereza kuti ana aziyezedwa ndi njiraazi, chifukwa makolo ana amatha kuthawa ndi ana posazindikila kuti akuwaphela tsogolo.
- **CG-** parents should allow children be tested using this method because if they don’t they are destroying their child’s future

b). **Kodi makolo awuzidwe zotani ndi uphungu wotani kuti amvesese za njira zoyezesera magazi za Cepheid ?**

- **CG-** Ndi zabwino njirazi chifukwa akaziwa status yamwana aziwa mene angamusamalarile ngati ali ndi matenda kapena ayi.
- **CG-**it is good because if they know the status of the child, they will know how they can take care of him/her is infected or not

1. **Kodi azibambo angatengepo gawo lanji kuti njira zoyezesera magazi za Cepheid zikhazikisidwe mu chipatala chathu chino cha Mulanje? Tingawalimbikise bwanji azibambo kuti azitenga nawo gawo mukuyezedwa magazi mu njira za Cepheid?**

- **CG-**  Atha kutengapo gawo powalimbikitsa akazi awo kuti ayezedwe kudzera munjilazi zomwe zili zachangu zosadikila kuti papite nthawi yaitali kuti amve sozatila.
- **CG-** They can take part by motivating their wives to get tested using these ways which are fast and you don’t have to wait for a long time.

1. **Kodi anthu a mmudzi mwanu angamve bwanji njira zoyezesera magazi za Cepheid zitakhazikisidwa pa chipatala chanu chaching’ono mmudzi mwanu. Tingatani kuti anthu a mmudzi muno alimbikisidwe kutenga nawo mbali mu njira zoyezetsera magazi za Cepheid?**

- **CG-** Kupita kwa mfumu kukafotokoza za ubwino wanjira za tsopanozi kuti amfumu ayitane msonkhano
- **CG-** explaining to the village head the importance of these new methods so he can host a convention.

1. **Kodi inu ndi anthu ena mma midzi mu mumakhala ndi nkhwa zanji zokhuzana ndi kulandila zosatira za magazi mwana akayezedwa kuti tiziwe kuti mwana ali ndi HIV kapena ayi?**

- **CG-** Nkhwawa imakhalapo kuti anthu ena angathe kumamusala mu community imene akukhalamo
- **CG-** the fear comes because other people might discriminate the child in the community

1. **Kodi mungakhale ndi njira kapena maganizo a momwe tingathandizire kuchepesa nkhawa zokhuzana ndikulandila zotsatira za magazi mwana wayezedwa kuti tidziwe kuti mwana ali ndi HIV kapena ayi?**

- **CG-** Anthu amene alindi kachilombo azibwera poyera ndikunena kuti kukhala ndi ka chilombo simathero a zonse koma kungotsatila ndondomeko yoyenera yomwe akuwuzani kuchipatala
- **CG-** People who have the virus should openly come to air and tell everyone the virus isn’t the end of everything but just follow the correct prescriptions described by the hospital.

1. **Kuchokera pa nthawi yomwe mwana wanu wayezedwa magazi kuti tidziwe kuti mwana ali ndi HIV kapena ayi, mungapilile nthawi yayitali bwanji kuti mudziwe zosatira**

**Tsiku lomwelo**

**Patatha masiku**

**Miyezi iwiri kapena itatu**

**Fotokozani zifukwa zomwe mungasankhile yankho limeneli**

- **CG-**  Ndasankha tsiku lomwelo chifukwa choti ndziziwa mwana wanga m’mene alili tsiku lomwero ndikumupasa thandizo mwansanga.
- **CG-** I choose the same day so that I will know how my child is the same day and give him help as soon as possible.

1. **Mwana wanu atayezedwa magazi, mungafune kudikila nthawi yayitali bwanji kuti mudziwe kuti mwana ali ndi HIV yomwe yimayambitsa matenda a AIDS?**

**Same day**

**Patatha masiku**

**Miyezi iwiri kapena itatu**

**Fotokozani zifukwa zimene mwasankhila yankho limenelo**

- **CG-** Chifukwa choti ndimpase uphungu woyenerala munthawi yake.
- **CG-** to give the correct guidance in due time

1. **Mwana wanu atayezedwa magazi mungafune kudikila nthaawi yayitali bwanji kuti muziwe kuti mwana alibe HIV yomwe imayambitsa matenda a AIDS**

**Same Day**

**Patatha masiku**

**Miyezi iwiri kapena itatu**

**Fotokozani zifukwa zomwe mungasankhile yankho limenelo**

- **CG-** Akadziwa tsiku lomwero ayamba kuona tsogolo lake kuti amusamare motani.
- **CG-** if they know on the same day you will know the future in how the child will be taken care of.

1. **kodi mungafune muwuzidwe zotani ndi uphungu otani kuti inu mupange chisankho choti mwana wanu ayezedwe magazi kuti mudziwe kuti mwana ali ndi HIV yomwe imayambitsa matenda a AIDS kapena ayi? Fotokozani bwino lomwe.**

- **CG-** Atha kumusamala malingana ndi mmene zotsatila zatulukila.
- **CG-**The child will be taken care in accordance with the results

1. **Mungafune kuti tikufikileni mu njira yotani kuti tikuwuzeni zimezi ndikukupasani uphungu umenewu wa njira zoyezesera magazi za Cepheid ndi ?**

- **CG-** Ndingakonde mumatifikila mosayang’ana nkhope, kapena ndi mmene alili munthu zimenezi zizathandiza kuti tiwulandile uphungu moyenera.
- **CG-** I would be happy if you could reach us indiscriminately to help us receive the correct guidance.

1. **Kodi mungathe kuwalimbikisa makolo anzanu kapena owasamalira ana kuti alore ana Awo ayezedwwe magazi kuti aziwe ngati ali ndi HIV yoyambitsa matenda a AIDS kugwilitsa ntchito Cepheid?**

- **CG-** Eya
- **CG-** yes

**15b) Nkhawa zanu zingakhale zotani ndi mayezedwe amenewa a ndi Cepheid?**

- **CG-** NKhwawa itha kukhalapo yokuti mwana akamatengedwa magazi amatha kusuntha suntha zomwe zimapangisa kuthyokera kwa singano
- I would be worried that the drawing needle might break while inside the child due to the moving about of the child while blood draw.

1. **Kodi mungamve bwanji ngati munthu wina wa mmudzi mwanu ataziwa zotsatira za magazi a mwana wanu atayezedwa kufufuza ngati ali ndi HIV kapena ayi?**

- **CG-** Sangamve bwino chifukwa nkhani za HIV zimafunika chinsinsi kukhale kufuna kako kuti unene mmene mwana alili kuti ali positivi kapena ayi.
- **CG-** I wouldn’t feel good because issues about HIV should be kept private unless its your choice to tell people if your child is positive.

1. **Kodi muli ndi maganizo kapena nkhawa zina zomwe mungafune kutidziwisa pa nkhani imeneyi**

- **CG-**  Alibe nkhawa chifukwa zotsatira ava nthawi yomweyo.
- **CG-** I have no comment or question because the results will be stated immediately.
