# Supplementary material for: ‘If I am on ART, my new-born baby should be put on treatment immediately’: Exploring the acceptability, and appropriateness of Cepheid Xpert HIV-1 Qual assay for early infant diagnosis of HIV in Malawi
Source: PLOS Glob Public Health. 2023 Mar 10;3(3):e0001135. doi: 10.1371/journal.pgph.0001135 (PMC10021387; doi:10.1371/journal.pgph.0001135)
Supplement: S2 File — (ZIP) [file pgph.0001135.s005.zip › transcripts responses chichewa& english/DET004.docx]

**DET004­_CG_F_24.7.18**

1. **Malingana ndi mmene tafotokozera za kayezedwe ka Cepheid, mwana ayenera kutengedwa magazi pachara kapena pa nsempha, inu monga kholo mungamve bwanji kuti mwana wanu ayezedwe magazi kuzera njira zimezi?**

- **CG-** Ndingachilandile bwino chifukwa ndifuna ndimve tsiku lomwero kuti mwana wanga ali bwanji.
- **CG-** I would receive it in a good way because I would want to know how the child is, on the same day.

1. **Kwainu monga kholo la mwana wa chichepere, maganizo anu ndi otani pokhuzana ndi mayezedwe a magazi kuti tidziwe kuti mwana ali ndi HIV kapena ayi malingana ndi mmene tafotokozera za kayezedwe ka Cepheid kuti zosatira zimatuluka kwa minitsi 92?**

- **CG-**  Ine ndikuganiza kuti njira imeneyi ndiyabwino chifukwa indithandiza kuziwa kuti mwana wanga mthupi mwake muli bwanji, ndipo kumuthandiza moyenerela pa nthawi yake.
- **CG-** I think it’s a good way because I will know the status of my child’s body and how I will help him/her.

1. **Kodi njira zimenezi tingazikhazikise bwanji mu zipatala? (tatiwuzani, tiyambe ndi gulu liti la anthu ndipo nchifukwa chani mukuganiza kuti tiyambe ndi gulu limeneli chifukwa chain?**

- **CG-**  Tiyambe ndi gulu la ana chifukwa ndikumene kumapezeka ana ochuluka malingana ndikuti takhala nthawi yayitali tikudikila njira ngati imeneyi.
- **CG-** we should start with the children because we have a lot of children and we have waited for a long time for a method like this.

1. **Kodi tingapange bwanji kuti kuyezesa magazi kwa ana ndi makolo awo kapena anthu owayang’ira zikhale za chinsinsi?**

- **CG-**  Tikuyenera ife monga makolo kusunga chinsinsi.
- **CG-** we as parents must keep this private.

1. **Kodi makolo angatengepo gawo lanji kuti njira zoyezesera magazi za Cepheid ndi zikhazikisidwe mu chipatala chathu chino cha Mulanje?**

- **CG-** Makolo akuyenera kulimbikitsana chifukwa njira zimenezo zimatuluka tsiku lomwero
- **CG-**parents tell others because this method brings results the same day.

b). **Kodi makolo awuzidwe zotani ndi uphungu wotani kuti amvesese za njira zoyezesera magazi za Cepheid?**

- **CG-** Makolo akuyenera kupatsidwa uphungu wabwino ndikuwa fotokozera bwinobwino pa zamayezedwe amenewa a Cepheid ndi chifukwa choti moyo mwana umakhala m’manja mwawo.
- **CG-** Parents need to be given good counselling and a good explanation on this method of testing using Cepheid and cause life of their kid is in their hands.

1. **Kodi azibambo angatengepo gawo lanji kuti njira zoyezesera magazi za Cepheid ndi zikhazikisidwe mu chipatala chathu chino cha Mulanje? Tingawalimbikise bwanji azibambo kuti azitenga nawo gawo mukuyezedwa magazi mu njira za Cepheid?**

- **CG-**  Tikuyenera kuwatsimikizira kuti njira zimenezi ndizoyenera chifukwa zotsatira zamagazi zimatuluka tsiku lomwero, ndipo akuyenera nawonso kutengapo gawo kuti mwana wathu akhala wa thanzi ndi moyo wa mphamvu.
- **CG-**we need to assure them that these ways are right because the results come out on the same day, and they also need to take part so our child should be healthy and strong.

1. **Kodi anthu a mmudzi mwanu angamve bwanji njira zoyezesera magazi za Cepheid ndi zitakhazikisidwa pa chipatala chanu chaching’ono mmudzi mwanu. Tingatani kuti anthu a mmudzi muno alimbikisidwe kutenga nawo mbali mu njira zoyezetsera magazi za Cepheid?**

- **CG-** Zimenezi zingakhale zabwino ndithu chifukwa choti sitimayenda ntunda wautali pofuna kumva zosatila zamwana wathu.
- **CG-** this can be good because we will not walk a long distance to know the results of our child.

1. **Kodi inu ndi anthu ena mma midzi mu mumakhala ndi nkhwa zanji zokhuzana ndi kulandila zosatira za magazi mwana akayezedwa kuti tiziwe kuti mwana ali ndi HIV kapena ayi?**

- **CG-**  Ine sindingakhale ndi nkhawa chifukwa choti ndikhala ndikuziwa zosatira za mwana wanga ndikukhala wopewa konso kuziwa kuti mwana wanga ndimusamala bwanji.
- **CG-** I wouldn’t have concerns because I will know the results of my child and know how to prevent and take care of him/her.

1. **Kodi mungakhale ndi njira kapena maganizo a momwe tingathandizire kuchepesa nkhawa zokhuzana ndikulandila zotsatira za magazi mwana wayezedwa kuti tidziwe kuti mwana ali ndi HIV kapena ayi?**

- **CG-** Njira yothandizira kuchepesa nkhawa ingakhale kupemphera, komanso kukhala wolimbikila pa moyo wa mwana wanga kuti azakhalae ndi tsogolo labwino.
- **CG-** way of reducing the stress can be praying and working hard on my child so that the child should have a great future

1. **Kuchokera pa nthawi yomwe mwana wanu wayezedwa magazi kuti tidziwe kuti mwana ali ndi HIV kapena ayi, mungapilile nthawi yayitali bwanji kuti mudziwe zosatira**

- **Same day**

**Patatha masiku**

**Miyezi iwiri kapena itatu**

**Fotokozani zifukwa zomwe mungasankhile yankho limeneli**

- **CG-** Kudikila ndikowawa, ndingakonde kuti ndidziwe tsiku lomwero chifukwa ndidziwa mapewedwe kapena momuthandizira mwana.
- **CG-** waiting is painful, I would prefer knowing the results on the same day, because I will know how I will prevent and help my child.

1. **Mwana wanu atayezedwa magazi, mungafune kudikila nthawi yayitali bwanji kuti mudziwe kuti mwana ali ndi HIV yomwe yimayambitsa matenda a AIDS?**

- **Same day**

**Patatha masiku**

**Miyezi iwiri kapena itatu**

**Fotokozani zifukwa zimene mwasankhila yankho limenelo**

- **CG-** Chifukwa choti ukudikira kuti umve mmene mwana wako alili ndiye ndiye ndikoyenera kuti umve tsiku lomwero ndikuziwa m’mene ungamuthandizire msanga.
- **CG-** because you are waiting to hear how your child will be so its important to know fast to see how you will help the child.

1. **Mwana wanu atayezedwa magazi mungafune kudikila nthaawi yayitali bwanji kuti muziwe kuti mwana alibe HIV yomwe imayambitsa matenda a AIDS**

- **Same day**

**Patatha masiku**

**Miyezi iwiri kapena itatu**

**Fotokozani zifukwa zomwe mungasankhile yankho limenelo**

- **CG-** Ndikuwona ngati.
- **CG-** I just think so

1. **kodi mungafune muwuzidwe zotani ndi uphungu otani kuti inu mupange chisankho choti mwana wanu ayezedwe magazi kuti mudziwe kuti mwana ali ndi HIV yomwe imayambitsa matenda a AIDS kapena** ayi**? Fotokozani bwino lomwe.**

- **CG-** Mutandifotokozera kapewedwe ndi masamalidwe a mwana wanga nditha kukhala osangalala zedi.
- **CG-** if you would explain the preventative and ways to care for the child, I would be extremely happy.

1. **Mungafune kuti tikufikileni mu njira yotani kuti tikuwuzeni zimezi ndikukupasani uphungu umenewu wa njira zoyezesera magazi za Cepheid?**

- **CG-**  Monga ine kholo ndikuganiza kutikumatifikila kusikero ya ana kuti tonse tizimvera limodzi.
- **CG-** I think its better at antenatal clinic so that we can all listen together

1. **Kodi mungathe kuwalimbikisa makolo anzanu kapena owasamalira ana kuti alore ana Awo ayezedwwe magazi kuti aziwe ngati ali ndi HIV yoyambitsa matenda a AIDS kugwilitsa ntchito Cepheid?**

- **CG-**  Eya
- **CG-** yes

**15b) Nkhawa zanu zingakhale zotani ndi mayezedwe amenewa a Cepheid?**

- **CG-**  Ndikwabwino kuwalimbikitsa makolo anzathu za kugwilitsa njira ya Cepheid chifukwa umaziwa tsiku lomwero ndipo ine monga kholo sindingakhale ndinkhawa konse.
- **CG-** it is good motivating our fellow parents on the use of Cepheid because you know the same day and me as a parent wouldn’t have a problem with it.

1. **Kodi mungamve bwanji ngati munthu wina wa mmudzi mwanu ataziwa zotsatira za magazi a mwana wanu atayezedwa kufufuza ngati ali ndi HIV kapena ayi?**

- **CG-** Ndilibe ganizo lililonse.
- **CG-** I have no idea

1. **Kodi muli ndi maganizo kapena nkhawa zina zomwe mungafune kutidziwisa pa nkhani imeneyi**

- **CG-**  Ndilibe nkhawa kapena vuto lina lililonse
- **CG-** no concern.
