# Supplementary material for: ‘If I am on ART, my new-born baby should be put on treatment immediately’: Exploring the acceptability, and appropriateness of Cepheid Xpert HIV-1 Qual assay for early infant diagnosis of HIV in Malawi
Source: PLOS Glob Public Health. 2023 Mar 10;3(3):e0001135. doi: 10.1371/journal.pgph.0001135 (PMC10021387; doi:10.1371/journal.pgph.0001135)
Supplement: S2 File — (ZIP) [file pgph.0001135.s005.zip › transcripts responses chichewa& english/DET005.docx]

**DET005_CG_F_24.7.18**

1. **Malingana ndi mmene tafotokozera za kayezedwe ka Cepheid, mwana ayenera kutengedwa magazi pachara kapena pa nsempha, inu monga kholo mungamve bwanji kuti mwana wanu ayezedwe magazi kuzera njira zimezi?**

- **CG-** Palibe vuto cholinga change ndikufuna kudziwa mmene mwana wanga alili.
- **CG-** no problem because I only want to know how my child is.

1. **Kwainu monga kholo la mwana wa chichepere, maganizo anu ndi otani pokhuzana ndi mayezedwe a magazi kuti tidziwe kuti mwana ali ndi HIV kapena ayi malingana ndi mmene tafotokozera za kayezedwe ka Cepheid kuti zosatira zimatuluka kwa minitsi 92?**

- **CG-**  PAli vuto chifukwa a dokotala otenga ana magazi pa nsempha ndi ochepa.
- **CG-** The problem is that there are not many doctors who draw blood from the veins

1. **Kodi njira zimenezi tingazikhazikise bwanji mu zipatala? (tatiwuzani, tiyambe ndi gulu liti la anthu ndipo nchifukwa chani mukuganiza kuti tiyambe ndi gulu limeneli chifukwa chain?**

- **CG-** Njira zimenezi zitsegulisidwe mu zipatala chifukwa ku chipatala ndikumene kumapezeka azimayi ambiri ndi ana pofalisa uthenga kuzera ma positala, tiyambira akuluakulu chifukwa kwa mwana nsempha supezeka msanga nde kholo litha kumadandaula kuti akuwapwetekera mwana
- **CG-** These ways should be implemented in hospitals, because that is were you find a lot of women and children. When spreading the message using posters we should start with the adults because when you cannot find blood vessels of a child, parents might complain that you are hurting their child.

1. **Kodi tingapange bwanji kuti kuyezesa magazi kwa ana ndi makolo awo kapena anthu owayang’ira zikhale za chinsinsi?**

- **CG-**  Kumapanga m’modzi m’modzi zitha kukhala za chinsinsi.
- **CG-** testing one person at a time for privacy

1. **Kodi makolo angatengepo gawo lanji kuti njira zoyezesera magazi za Cepheid zikhazikisidwe mu chipatala chathu chino cha Mulanje?**

- **CG-** Kuwabweresa ana ku chipatala osachedwa nawo ayi.
- **CG-** bringing children to the hospital without hesitation.

b). **Kodi makolo awuzidwe zotani ndi uphungu wotani kuti amvesese za njira zoyezesera magazi za Cepheid?**

- **CG-** Aziwe cholinga cha Cepheid ndi konsa azindikire kuti njirazi zithandiza kuti aziwe mmene mwana alili mwachangu.
- **CG-** they should know the reason for Cepheid and why the method will help the child as soon as possible.

1. **Kodi azibambo angatengepo gawo lanji kuti njira zoyezesera magazi za Cepheid zikhazikisidwe mu chipatala chathu chino cha Mulanje? Tingawalimbikise bwanji azibambo kuti azitenga nawo gawo mukuyezedwa magazi mu njira za Cepheid?**

- **CG-**  Kuwalimbikitsa kuti azibwera ngati banja osato kungosiya amayi, kuwalimbikitsa kuti akabwera kuti aziyambilira ndi iwowo kuti asachedwe akagwire ntchito zina monga bambo.
- **CG-** telling them why it is important for them to come as a family and that when they come they will be assisted before others so they can continue their work as men

1. **Kodi anthu a mmudzi mwanu angamve bwanji njira zoyezesera magazi za Cepheid zitakhazikisidwa pa chipatala chanu chaching’ono mmudzi mwanu. Tingatani kuti anthu a mmudzi muno alimbikisidwe kutenga nawo mbali mu njira zoyezetsera magazi za Cepheid?**

- **CG-** Atha kukhala osangalala, Kuwafotokozera ubwino wanjirazi kuti amvetsetse ndizachangu komanso kupangitsa nsonkhano ma midzi.
- **CG-** they would be happy, explaining the importance of these methods so they can understand that it is fast and holding convections.

1. **Kodi inu ndi anthu ena mma midzi mu mumakhala ndi nkhwa zanji zokhuzana ndi kulandila zosatira za magazi mwana akayezedwa kuti tiziwe kuti mwana ali ndi HIV kapena ayi?**

- **CG-**  Nkhawa imakhala yoti mwana atha kumadwala komanso amafunika zakudya zabwino akakhala ndi HIV.
- **CG-** my fear would arise towards the fact the child might be getting sick and need good food because of HIV

1. **Kodi mungakhale ndi njira kapena maganizo a momwe tingathandizire kuchepesa nkhawa zokhuzana ndikulandila zotsatira za magazi mwana wayezedwa kuti tidziwe kuti mwana ali ndi HIV kapena ayi?**

- **CG-**  Njira yake anthu akhale mmagulu nkumakambirana , kuchotsana nkhawa.
- **CG-** People need to be in groups and talk thereby taking away the fear.

1. **Kuchokera pa nthawi yomwe mwana wanu wayezedwa magazi kuti tidziwe kuti mwana ali ndi HIV kapena ayi, mungapilile nthawi yayitali bwanji kuti mudziwe zosatira**

- **Tsiku lomwelo**

**Patatha masiku**

**Miyezi iwiri kapena itatu**

**Fotokozani zifukwa zomwe mungasankhile yankho limeneli**

- **CG-**  Chifukwa choti zikachedwa umayiwala kuti mwana anatengedwa magazi.
- **CG-**because if it takes too long you might forget your child’s blood was taken for testing.

1. **Mwana wanu atayezedwa magazi, mungafune kudikila nthawi yayitali bwanji kuti mudziwe kuti mwana ali ndi HIV yomwe yimayambitsa matenda a AIDS?**

- **TSiku lomwelo**

**Patatha masiku**

**Miyezi iwiri kapena itatu**

**Fotokozani zifukwa zimene mwasankhila yankho limenelo**

- **CG-** Ndimafuna kuziwa tsiku lomwero chifukwa choti ndikufuna ndidziwe mmene ndingasamalilile mwana akapezeka nako ka chilombo kapena ayi.
- **CG-** I need to know on the same day so I will know how I will take care of my child is found positive or not.

1. **Mwana wanu atayezedwa magazi mungafune kudikila nthaawi yayitali bwanji kuti muziwe kuti mwana alibe HIV yomwe imayambitsa matenda a AIDS**

- **Tsiku lomwelo**

**Patatha masiku**

**Miyezi iwiri kapena itatu**

**Fotokozani zifukwa zomwe mungasankhile yankho limenelo**

- **CG-**  Chifukwa chakuti ndikhumbo la kholo aliyense kuziwa zotsatira za mwana wake kuti aziwe mmene angamusamalalire mwanayo.
- **CG-** Because it is the will of every parent to know the results of their children so that they will know how they will take care of him/her

1. **kodi mungafune muwuzidwe zotani ndi uphungu otani kuti inu mupange chisankho choti mwana wanu ayezedwe magazi kuti mudziwe kuti mwana ali ndi HIV yomwe imayambitsa matenda a AIDS kapena ayi? Fotokozani bwino lomwe.**

- **CG-**  Kungolimbikitsidwa kuti akayezedwa chithandizo ulandila
- .CG- To be encouraged that when they get tested they will receive the needed help

1. **Mungafune kuti tikufikileni mu njira yotani kuti tikuwuzeni zimezi ndikukupasani uphungu umenewu wa njira zoyezesera magazi za Cepheid ndi ?**

- **CG-** Kufikilidwa kuzera pa wailesi komanso masewero komanso kuyika ma positala mzipatala ofotokoza za ubwino wa njirazi.
- **CG-** Reach then using the radio and plays even posters should be posted at the hospitals

1. **Kodi mungathe kuwalimbikisa makolo anzanu kapena owasamalira ana kuti alore ana Awo ayezedwwe magazi kuti aziwe ngati ali ndi HIV yoyambitsa matenda a AIDS kugwilitsa ntchito Cepheid?**

- **CG-**  Eya
- **CG-** yes

**15b) Nkhawa zanu zingakhale zotani ndi mayezedwe amenewa a Cepo heid?**

- **CG-** Nkhawa palibe koma mwana wang’ono amavuta kuti umutenge magazi.
- **CG-** No concerns but young children become difficult during blood draw.

1. **Kodi mungamve bwanji ngati munthu wina wa mmudzi mwanu ataziwa zotsatira za magazi a mwana wanu atayezedwa kufufuza ngati ali ndi HIV kapena ayi?**

- **CG-** Masiku ano ndizosadetsa nkhawa chifukwa ndikadziwa ndidziwa mmene ndingamusamalilile mwana wanga
- **CG-** These days I wouldn’t be too worried because if I know I will know how I will take care of my child.

1. **Kodi muli ndi maganizo kapena nkhawa zina zomwe mungafune kutidziwisa pa nkhani imeneyi**

- **CG-**  Nkhawa ndiyoti poti zayamba kumene nde sitikudziwa ngati zotsatilazo zibweredi lero lomwe kapena ayi.
- **CG-** My concern is that it is a new thing so I will not know if the results will really be out on the same day.
