# Supplementary material for: ‘If I am on ART, my new-born baby should be put on treatment immediately’: Exploring the acceptability, and appropriateness of Cepheid Xpert HIV-1 Qual assay for early infant diagnosis of HIV in Malawi
Source: PLOS Glob Public Health. 2023 Mar 10;3(3):e0001135. doi: 10.1371/journal.pgph.0001135 (PMC10021387; doi:10.1371/journal.pgph.0001135)
Supplement: S2 File — (ZIP) [file pgph.0001135.s005.zip › transcripts responses chichewa& english/DET006.docx]

**DET006_CG_F_24.7.18**

1. **Malingana ndi mmene tafotokozera za kayezedwe ka Cepheid, mwana ayenera kutengedwa magazi pachara kapena pa nsempha, inu monga kholo mungamve bwanji kuti mwana wanu ayezedwe magazi kuzera njira zimezi?**

- **CG-** NJira zimenezi zikuwoneka kuti ndizothandiza.
- **CG-** This method looks useful

1. **Kwainu monga kholo la mwana wa chichepere, maganizo anu ndi otani pokhuzana ndi mayezedwe a magazi kuti tidziwe kuti mwana ali ndi HIV kapena ayi malingana ndi mmene tafotokozera za kayezedwe ka Cepheid ndi kuti zosatira zimatuluka kwa minitsi 92?**

- **CG-**  Ineyo ndiwochilandira kwambiri chifukwa njira zimenezi zitithandiza kuziwa msanga mmene mmatupi a mwana wathu aliri.
- **CG-** I greatly welcome this because this method help us know the status of our child’s health quickly

1. **Kodi njira zimenezi tingazikhazikise bwanji mu zipatala? (tatiwuzani, tiyambe ndi gulu liti la anthu ndipo nchifukwa chani mukuganiza kuti tiyambe ndi gulu limeneli chifukwa chain?**

- **CG-**  Tikuyenera kufikila mafumu ndikuwafotokozera kuti awuze anthu a m’mudzi za mayezedwe amenewa, muyambire ana chifukwa njira zimenezi kunalibe ndi kale lonse.
- **CG-** we need to reach village heads and explain to them about how this testing takes place and they should start with children cause we didn’t have it earlier on.

1. **Kodi tingapange bwanji kuti kuyezesa magazi kwa ana ndi makolo awo kapena anthu owayang’ira zikhale za chinsinsi?**

- **CG-** Popeleka uphungu wabwino kwa owayang’anira komanso kuwaziwitsa kuti ndi ufulu wawo kusunga chinsinsi.
- **CG-** Giving good counselling to the guardians and how it is their right to keep it a secret.

1. **Kodi makolo angatengepo gawo lanji kuti njira zoyezesera magazi za Cepheid zikhazikisidwe mu chipatala chathu chino cha Mulanje?**

- **CG-** Makolo akuyenera kuchilandira ndikumvesesa pamayezedwe amenewa.
- **CG-** Parents need to receive and accept this type of testing

b). **Kodi makolo awuzidwe zotani ndi uphungu wotani kuti amvesese za njira zoyezesera magazi za Cepheid?**

- **CG-** Tikunera kuwafotokozera kuti uphungu umenewu ndiwonera kwa ana athu.
- **CG-** we need to explain with the importance of the test

1. **Kodi azibambo angatengepo gawo lanji kuti njira zoyezesera magazi za Cepheid ndi zikhazikisidwe mu chipatala chathu chino cha Mulanje? Tingawalimbikise bwanji azibambo kuti azitenga nawo gawo mukuyezedwa magazi mu njira za Cepheid ?**

- **CG-**  Ndilibe ganizo lililonse.
- **CG-** no comment here

1. **Kodi anthu a mmudzi mwanu angamve bwanji njira zoyezesera magazi za Cepheid zitakhazikisidwa pa chipatala chanu chaching’ono mmudzi mwanu. Tingatani kuti anthu a mmudzi muno alimbikisidwe kutenga nawo mbali mu njira zoyezetsera magazi za Cepheid?**

- **CG-** Zingakhale zonyaditsa ndi zokoma chifukwa mayendedwe angakhale osavuta komanso tingakhale olimbikitsana m’mudzi pakayezedwe kasopanoka.
- **CG-**it would be wonderful because transport would be easy and we would be motivated to get tested.

1. **Kodi inu ndi anthu ena mma midzi mu mumakhala ndi nkhwa zanji zokhuzana ndi kulandila zosatira za magazi mwana akayezedwa kuti tiziwe kuti mwana ali ndi HIV kapena ayi?**

- **CG-**  Aliyense pamene akuyezedwa amakhala ndi nkhawa makamaka pamene ukamamvesera zotsatira za magazi ako chifukwa umatha kuganiza kuti sindifa ndikapezeka nako.
- **CG-** Everyone who is tested has fear because they are afraid if found positive they may die

1. **Kodi mungakhale ndi njira kapena maganizo a momwe tingathandizire kuchepesa nkhawa zokhuzana ndikulandila zotsatira za magazi mwana wayezedwa kuti tidziwe kuti mwana ali ndi HIV kapena ayi?**

- **CG-**  Kupemphera ndichiyambi chochotsa nkhawa komanso kulimbikitsana.
- **CG-** Prayer is the beginning of a fearless life and helping each other

1. **Kuchokera pa nthawi yomwe mwana wanu wayezedwa magazi kuti tidziwe kuti mwana ali ndi HIV kapena ayi, mungapilile nthawi yayitali bwanji kuti mudziwe zosatira**

- **Tsiku lomwelo**

**Patatha masiku**

**Miyezi iwiri kapena itatu**

**Fotokozani zifukwa zomwe mungasankhile yankho limeneli**

- **CG-**  Chifukwa zizatithandiza kuziwa m’mene tingamusamalilire mwana wanga ndikumuteteza moyenera
- **CG-** it will help us know how we will take care of our child.

1. **Mwana wanu atayezedwa magazi, mungafune kudikila nthawi yayitali bwanji kuti mudziwe kuti mwana ali ndi HIV yomwe yimayambitsa matenda a AIDS?**

- **TSiku lomwelo**

**Patatha masiku**

**Miyezi iwiri kapena itatu**

**Fotokozani zifukwa zimene mwasankhila yankho limenelo**

- **CG-** Nthawi ina iliyonse imene a dokotala anenere kuti tidikire.
- **CG-** according to how long the doctor tells us to wait.

1. **Mwana wanu atayezedwa magazi mungafune kudikila nthaawi yayitali bwanji kuti muziwe kuti mwana alibe HIV yomwe imayambitsa matenda a AIDS**

- **Tsiku lomwelo**

**Patatha masiku**

**Miyezi iwiri kapena itatu**

**Fotokozani zifukwa zomwe mungasankhile yankho limenelo**

- **CG-**  Chifukwa choti ndidziwa nthawi yoyenera komanso ngati mwana wanga akuyenera kumwa mankhwala kapena ayi.
- **CG-** it is because I will know if my child needs to take medications or not.

1. **kodi mungafune muwuzidwe zotani ndi uphungu otani kuti inu mupange chisankho choti mwana wanu ayezedwe magazi kuti mudziwe kuti mwana ali ndi HIV yomwe imayambitsa matenda a AIDS kapena ayi? Fotokozani bwino lomwe.**

- **CG-**  ife tikuyenera kulandila uphungu monga makolo, ndipo otofikila potiwuza uphunguwu akuyenera kutiwuza mwa ndondomeko.
- **CG-** us as parents need to be counselled and the counsellor needs to explain clear step by step.

1. **Mungafune kuti tikufikileni mu njira yotani kuti tikuwuzeni zimezi ndikukupasani uphungu umenewu wa njira zoyezesera magazi za Cepheid?**

- **CG-**  Potiphunzitsa kuzera ma wailesi komanso kumata zithunzi mzipatala zophunzitsa njirazi.
- **CG-** using radios and different posters in hospitals

1. **Kodi mungathe kuwalimbikisa makolo anzanu kapena owasamalira ana kuti alore ana Awo ayezedwwe magazi kuti aziwe ngati ali ndi HIV yoyambitsa matenda a AIDS kugwilitsa ntchito Cepheid?**

- **CG-**  Eya
- **CG-** yes

**15b) Nkhawa zanu zingakhale zotani ndi mayezedwe amenewa a Cepheid?**

- **CG-** Ine sindikuwonapi nkhawa iliyonse chifukwa njirayi itichitira ubwino kwabasi.
- **CG-**I have no problem with this because it is doing us much good.

1. **Kodi mungamve bwanji ngati munthu wina wa mmudzi mwanu ataziwa zotsatira za magazi a mwana wanu atayezedwa kufufuza ngati ali ndi HIV kapena ayi?**

- **CG-** Tikungoyenera kudziwa ndi ufulu wathu kuteteza moyo wa mwana wathu chifukwa sikoyenera kulola munthu wina kudziwa zosatila za mwana wathu, monga ine kholo ndikuyenera kutenga gawo ndikuteteza mwana wanga.
- **CG-**we need to know it is within our right to protect the life of our child and its not right to allow someone else results because me as a parent need to take part in protecting my child.

1. **Kodi muli ndi maganizo kapena nkhawa zina zomwe mungafune kutidziwisa pa nkhani imeneyi**

- **CG-**  Ine nkhawa yanga yaikulu ndiyapotenga magazi basi chifukwa ndimadabwa kuti magazi a mwana wanga akupita kuti.
- **CG-** I have great concern with where the blood goes after being taken
