# Supplementary material for: ‘If I am on ART, my new-born baby should be put on treatment immediately’: Exploring the acceptability, and appropriateness of Cepheid Xpert HIV-1 Qual assay for early infant diagnosis of HIV in Malawi
Source: PLOS Glob Public Health. 2023 Mar 10;3(3):e0001135. doi: 10.1371/journal.pgph.0001135 (PMC10021387; doi:10.1371/journal.pgph.0001135)
Supplement: S2 File — (ZIP) [file pgph.0001135.s005.zip › transcripts responses chichewa& english/DET007.docx]

**DET007_CG_F_24.7.18**

1. **Malingana ndi mmene tafotokozera za kayezedwe ka Cepheid, mwana ayenera kutengedwa magazi pachara kapena pa nsempha, inu monga kholo mungamve bwanji kuti mwana wanu ayezedwe magazi kuzera njira zimezi?**

- **CG-** Ndingamve bwino pofuna kumva zotsatira mwachangu.
- **CG-** I would feel good because I would know the results on the same day.

1. **Kwainu monga kholo la mwana wa chichepere, maganizo anu ndi otani pokhuzana ndi mayezedwe a magazi kuti tidziwe kuti mwana ali ndi HIV kapena ayi malingana ndi mmene tafotokozera za kayezedwe ka Cepheid ndi kuti zosatira zimatuluka kwa minitsi 92?**

- **CG-**  Maganizo anga ndi abino chifukwa choti ndidziwa ngati alinako kapena ayi.
- **CG-** I like this method because I will know is the child is negative or positive

1. **Kodi njira zimenezi tingazikhazikise bwanji mu zipatala? (tatiwuzani, tiyambe ndi gulu liti la anthu ndipo nchifukwa chani mukuganiza kuti tiyambe ndi gulu limeneli chifukwa chain?**

- **CG-**  Ayambilire ana Chifukwa choti ngati ine ndi mamwa mankhwala ndikuyenera kuti ndidziwe kuti mwana wanga alandile chithandizo mwansanga.
- **CG-** They should start with the children because if I am on ART I should know that my child should receive help as soon as possible as well.

1. **Kodi tingapange bwanji kuti kuyezesa magazi kwa ana ndi makolo awo kapena anthu owayang’ira zikhale za chinsinsi?**

- **CG-**  Tikhonza kumuwuza munthu mmodzi wapadera yemwe akutiyang’anira kuti atisungile chinsinsi .
- **CG-** The guardian should be told to keep it private.

1. **Kodi makolo angatengepo gawo lanji kuti njira zoyezesera magazi za Cepheid zikhazikisidwe mu chipatala chathu chino cha Mulanje?**

- **CG-**  Kwa ife makolo tikhonza kukawuza makolo ena kuti abwere atengepo nawo gawo pa njira zimenezi.
- **CG-** As parents we can tell other parents to come and take part.

b). **Kodi makolo awuzidwe zotani ndi uphungu wotani kuti amvesese za njira zoyezesera magazi za Cepheid?**

- **CG-** Ndikhonza kumvetsetsa munjira ina iliyonse.
- **CG-** I can understand in any way.

1. **Kodi azibambo angatengepo gawo lanji kuti njira zoyezesera magazi za Cepheid zikhazikisidwe mu chipatala chathu chino cha Mulanje? Tingawalimbikise bwanji azibambo kuti azitenga nawo gawo mukuyezedwa magazi mu njira za Cepheid?**

- **CG-**  Tikhonza kuwawuza kuti abwere azayezedwe kapena kuwalimbikitsa azibambo kuti atenge ana azayezedwe.
- **CG-** We can tell the husbands to come and get tested or bring the children for testing.

1. **Kodi anthu a mmudzi mwanu angamve bwanji njira zoyezesera magazi za Cepheid zitakhazikisidwa pa chipatala chanu chaching’ono mmudzi mwanu. Tingatani kuti anthu a mmudzi muno alimbikisidwe kutenga nawo mbali mu njira zoyezetsera magazi za Cepheid?**

- **CG-** Angasangalale chifukwa ndi njira yapafupi komanso yabwino.
- **CG-** They would be happy because it is easier and good.

1. **Kodi inu ndi anthu ena mma midzi mu mumakhala ndi nkhwa zanji zokhuzana ndi kulandila zosatira za magazi mwana akayezedwa kuti tiziwe kuti mwana ali ndi HIV kapena ayi?**

- **CG-** Kwa ine monga kholo sindingakhale ndi nkhawa chifukwa kuti vuto kuti mwana limugwere ndi chifukwa cha ine kholo.
- **CG-** As a parent, I would not have any concerns because if something was to happen to my child, it would be my fault.

1. **Kodi mungakhale ndi njira kapena maganizo a momwe tingathandizire kuchepesa nkhawa zokhuzana ndikulandila zotsatira za magazi mwana wayezedwa kuti tidziwe kuti mwana ali ndi HIV kapena ayi?**

- **CG-**  Kulimbikitsa kuti asakhale ndi nkhawa ena akayezedwa amafuna kuzipha koma umafuna kuwalimbikitsa kuti simathero azonse.
- **CG-** counselling them on why they should have no fear because some have suicidal thoughts and they need to know it is not the end of everything.

1. **Kuchokera pa nthawi yomwe mwana wanu wayezedwa magazi kuti tidziwe kuti mwana ali ndi HIV kapena ayi, mungapilile nthawi yayitali bwanji kuti mudziwe zosatira**

- **Same day**

**Patatha masiku**

**Miyezi iwiri kapena itatu**

**Fotokozani zifukwa zomwe mungasankhile yankho limeneli**

- **CG-** Tasankha tsiku lomwero chifukwa choti kudikila miyezi yambiri ukhoza kupwetekesa mwana mosasatira ndondomeko chifukwa chosadziwa.
- **CG-** I choose the same day because if there is need, the child should receive help on the same day

1. **Mwana wanu atayezedwa magazi, mungafune kudikila nthawi yayitali bwanji kuti mudziwe kuti mwana ali ndi HIV yomwe yimayambitsa matenda a AIDS?**

- **Same day**

**Patatha masiku**

**Miyezi iwiri kapena itatu**

**Fotokozani zifukwa zimene mwasankhila yankho limenelo**

- **CG-** Ndilibe ganizo lililonse.
- **CG-** No comment

1. **Mwana wanu atayezedwa magazi mungafune kudikila nthaawi yayitali bwanji kuti muziwe kuti mwana alibe HIV yomwe imayambitsa matenda a AIDS**

- **Same day**

**Patatha masiku**

**Miyezi iwiri kapena itatu**

**Fotokozani zifukwa zomwe mungasankhile yankho limenelo**

- **CG-** Ndichifuniro cha kholo lina lililonse kusiwa zotsatira tsiku lomwero.
- **CG-** it is the will of every parent to know on the same day

1. **kodi mungafune muwuzidwe zotani ndi uphungu otani kuti inu mupange chisankho choti mwana wanu ayezedwe magazi kuti mudziwe kuti mwana ali ndi HIV yomwe imayambitsa matenda a AIDS kapena ayi? Fotokozani bwino lomwe.**

- **CG-**  Ndilibe ganizo lililonse.
- **CG-** no comment

1. **Mungafune kuti tikufikileni mu njira yotani kuti tikuwuzeni zimezi ndikukupasani uphungu umenewu wa njira zoyezesera magazi za Cepheid?**

- **CG-**  Kutipeza kumudzi kwathu ndikuzatiphunzitsa za njira zimenezi za Cepheid.
- **CG-** finding us in our home villages and teaching us the cepheid method.

1. **Kodi mungathe kuwalimbikisa makolo anzanu kapena owasamalira ana kuti alore ana Awo ayezedwwe magazi kuti aziwe ngati ali ndi HIV yoyambitsa matenda a AIDS kugwilitsa ntchito Cepheid?**

- **CG-**  Eya
- **CG-** yes

**15b) Nkhawa zanu zingakhale zotani ndi mayezedwe amenewa a Cepheid?**

- **CG-**  Ndilibe nkhawa iliyonse.
- **CG-** no concern

1. **Kodi mungamve bwanji ngati munthu wina wa mmudzi mwanu ataziwa zotsatira za magazi a mwana wanu atayezedwa kufufuza ngati ali ndi HIV kapena ayi?**

- **CG-** Sindingamve bwino chifukwa ndi njira imodzi ya chinsinsi.
- **CG-** I would not feel good about it

1. **Kodi muli ndi maganizo kapena nkhawa zina zomwe mungafune kutidziwisa pa nkhani imeneyi**

- **CG-** Ganizo langa ndilakuti mungolimbikitsa kuthandiza anthu kuti adziwe zotsatira mwachangu.
- **CG-** I think you should encourage people to know their results earlier.
