# Supplementary material for: ‘If I am on ART, my new-born baby should be put on treatment immediately’: Exploring the acceptability, and appropriateness of Cepheid Xpert HIV-1 Qual assay for early infant diagnosis of HIV in Malawi
Source: PLOS Glob Public Health. 2023 Mar 10;3(3):e0001135. doi: 10.1371/journal.pgph.0001135 (PMC10021387; doi:10.1371/journal.pgph.0001135)
Supplement: S2 File — (ZIP) [file pgph.0001135.s005.zip › transcripts responses chichewa& english/DET008.docx]

**DET009_CG_F_24.7.18**

1. **Malingana ndi mmene tafotokozera za kayezedwe ka Cepheid ndi , mwana ayenera kutengedwa magazi pachara kapena pa nsempha, inu monga kholo mungamve bwanji kuti mwana wanu ayezedwe magazi kuzera njira zimezi?**

- **CG-** Palibe vuto chifukwa cholinga change ndikufuna kudziwa mmene mwana wanga alili.
- **CG-** No problem because the point is knowing how my child is.

1. **Kwainu monga kholo la mwana wa chichepere, maganizo anu ndi otani pokhuzana ndi mayezedwe a magazi kuti tidziwe kuti mwana ali ndi HIV kapena ayi malingana ndi mmene tafotokozera za kayezedwe ka Cepheid ndi kuti zosatira zimatuluka kwa minitsi 92?**

- **CG-**  Pamenepa palibe vuto koma nkhawa yanga ndiyokuti ma dotolo otenga magazi pa nsempha ndiochepa
- **CG-** No problem, but my concern is that there are a few doctors capable of doing this.

1. **Kodi njira zimenezi tingazikhazikise bwanji mu zipatala? (tatiwuzani, tiyambe ndi gulu liti la anthu ndipo nchifukwa chani mukuganiza kuti tiyambe ndi gulu limeneli chifukwa chain?**

- **CG-**  Kuyambira anthu akulu chifukwa akaziwa mmene alili atha kupanga chisankho chokuti akayezetse ana awo.
- **CG-** Stating with the adults because when they know their status they might get their children tested.

1. **Kodi tingapange bwanji kuti kuyezesa magazi kwa ana ndi makolo awo kapena anthu owayang’ira zikhale za chinsinsi?**

- **CG-**  Munthu azibwera kuchipatala yekha chifukwa amakhala awiriwiri ndi dokotala, osati kuwalondola chifukwa anthu a mmudzi akawona adziwa kuti munthu amayezedwa.
- **CG-** people must come to the hospital alone because if someone goes with them the village people would know they went for testing.

1. **Kodi makolo angatengepo gawo lanji kuti njira zoyezesera magazi za Cepheid zikhazikisidwe mu chipatala chathu chino cha Mulanje?**

- **CG-** Powuza makolo ena kuti azibwera nsanga kuchipata ndi ana
- **CG-** Telling other parents to come to the hospital with their children

b). **Kodi makolo awuzidwe zotani ndi uphungu wotani kuti amvesese za njira zoyezesera magazi za Cepheid?**

- **CG-** Aziwe cholinga cha Cepheid komanso azindikire kuti njirazi zithandidza kuti aziwe m’mene mwana alili mwachangu.
- **CG-** They should know the importance of Ceipheid and how helpful and fast this method is

1. **Kodi azibambo angatengepo gawo lanji kuti njira zoyezesera magazi za Cepheid zikhazikisidwe mu chipatala chathu chino cha Mulanje? Tingawalimbikise bwanji azibambo kuti azitenga nawo gawo mukuyezedwa magazi mu njira za Cepheid?**

- **CG-**  Abambo omvetsetsa atha kulimbikitsa kuti mayi atenge mwana kupita naye ku chipatala.
- **CG-** Understanding husbands would encourage their wives to go get their child tested.

1. **Kodi anthu a mmudzi mwanu angamve bwanji njira zoyezesera magazi za Cepheid zitakhazikisidwa pa chipatala chanu chaching’ono mmudzi mwanu. Tingatani kuti anthu a mmudzi muno alimbikisidwe kutenga nawo mbali mu njira zoyezetsera magazi za Cepheid?**

- **CG-** Anthu akhoza kuchilandila bwinochifukwa laiyense akufuna kudziwa mmena alili.
- **CG-** People would receive it positively because everyone wants to know their status.

1. **Kodi inu ndi anthu ena mma midzi mu mumakhala ndi nkhwa zanji zokhuzana ndi kulandila zosatira za magazi mwana akayezedwa kuti tiziwe kuti mwana ali ndi HIV kapena ayi?**

- **CG-** Nkhawa imakhala yoti mwana atha kumadwaladwala komanso amafunika zokudya zabwino akakhala ndi HIV.
- **CG-** I am worried that if positive my child would need good food and will be sick regularly.

1. **Kodi mungakhale ndi njira kapena maganizo a momwe tingathandizire kuchepesa nkhawa zokhuzana ndikulandila zotsatira za magazi mwana wayezedwa kuti tidziwe kuti mwana ali ndi HIV kapena ayi?**

- **CG-** Kumafunika ku mapemphera kuti usakhale ndi nkhawa chifukwa pemphero ndiye yankho.
- **CG-** Prayer is the only answer.

1. **Kuchokera pa nthawi yomwe mwana wanu wayezedwa magazi kuti tidziwe kuti mwana ali ndi HIV kapena ayi, mungapilile nthawi yayitali bwanji kuti mudziwe zosatira**

- **Same day**

**Patatha masiku**

**Miyezi iwiri kapena itatu**

**Fotokozani zifukwa zomwe mungasankhile yankho limeneli**

- **CG-** Chifukwa ponyamuka kunyumba umakhala ukufuna ukamve zotsatila osadikiranso nthawi yayitali.
- **CG-** Because when you leave home you expect to hear the results on the same day

1. **Mwana wanu atayezedwa magazi, mungafune kudikila nthawi yayitali bwanji kuti mudziwe kuti mwana ali ndi HIV yomwe yimayambitsa matenda a AIDS?**

**TSiku lomwelo**

- **Three days**

**Miyezi iwiri kapena itatu**

**Fotokozani zifukwa zimene mwasankhila yankho limenelo**

- **CG-** Chifukwa ponyamuka kunyumba umakhala kuti ukufuna ukamve zosatila kusadikila nthawi yayitali.
- **CG-** because when you leave home you expect to get results as soon as possible.

1. **Mwana wanu atayezedwa magazi mungafune kudikila nthaawi yayitali bwanji kuti muziwe kuti mwana alibe HIV yomwe imayambitsa matenda a AIDS**

- **Same day**

**Patatha masiku**

**Miyezi iwiri kapena itatu**

**Fotokozani zifukwa zomwe mungasankhile yankho limenelo**

- **CG- -** Chifukwa ponyamuka kunyumba umakhala kuti ukufuna ukamve zosatila kusadikila nthawi yayitali.
- **CG-** Because when leaving home, you expect to immediately get the results.

1. **kodi mungafune muwuzidwe zotani ndi uphungu otani kuti inu mupange chisankho choti mwana wanu ayezedwe magazi kuti mudziwe kuti mwana ali ndi HIV yomwe imayambitsa matenda a AIDS kapena ayi? Fotokozani bwino lomwe.**

- **CG-**  Kumafunika kuchivomereza.
- **CG-** I would just need to accept anything

1. **Mungafune kuti tikufikileni mu njira yotani kuti tikuwuzeni zimezi ndikukupasani uphungu umenewu wa njira zoyezesera magazi za Cepheid ndi ?**

- **CG-**  Akayunera kuzera pa wailesi kapena yakanema ndikumata ma positala.
- **CG-** Through radios or televisions and posters

1. **Kodi mungathe kuwalimbikisa makolo anzanu kapena owasamalira ana kuti alore ana Awo ayezedwwe magazi kuti aziwe ngati ali ndi HIV yoyambitsa matenda a AIDS kugwilitsa ntchito Cepheid?**

- **CG-**  Eya
- **CG-** yes

**15b) Nkhawa zanu zingakhale zotani ndi mayezedwe amenewa a Cepheid?**

- **CG-** Zomutenga mwana magazi zinandipatsa chiwopsezo kuti magaziwo akupita nawo kuti chifukwa magaziwo anatenga ambiri.
- **CG-** They took a lot of blood, my concern is what they are going to do with all that blood.

1. **Kodi mungamve bwanji ngati munthu wina wa mmudzi mwanu ataziwa zotsatira za magazi a mwana wanu atayezedwa kufufuza ngati ali ndi HIV kapena ayi?**

- **CG-** Ndilibe ganizo lililonse.
- **CG-** no comment

1. **Kodi muli ndi maganizo kapena nkhawa zina zomwe mungafune kutidziwisa pa nkhani imeneyi**

- **CG-** Ndilibe maganizo kapena nkhawa ina iliyonse.
- **CG-** No comment
