# Supplementary material for: ‘If I am on ART, my new-born baby should be put on treatment immediately’: Exploring the acceptability, and appropriateness of Cepheid Xpert HIV-1 Qual assay for early infant diagnosis of HIV in Malawi
Source: PLOS Glob Public Health. 2023 Mar 10;3(3):e0001135. doi: 10.1371/journal.pgph.0001135 (PMC10021387; doi:10.1371/journal.pgph.0001135)
Supplement: S2 File — (ZIP) [file pgph.0001135.s005.zip › transcripts responses chichewa& english/DET009.docx]

**DET0010_CG_F_24.7.18**

1. **Malingana ndi mmene tafotokozera za kayezedwe ka Cepheid, mwana ayenera kutengedwa magazi pachara kapena pa nsempha, inu monga kholo mungamve bwanji kuti mwana wanu ayezedwe magazi kuzera njira zimezi?**

- **CG-**  Ndizabwino zimenezi pokutenga magazi chonchi, chifukwa akawona akhoza kukuthandiza.
- **CG-** it is good because when drawing blood like this and it is screened you may be helped

1. **Kwainu monga kholo la mwana wa chichepere, maganizo anu ndi otani pokhuzana ndi mayezedwe a magazi kuti tidziwe kuti mwana ali ndi HIV kapena ayi malingana ndi mmene tafotokozera za kayezedwe ka Cepheid kuti zosatira zimatuluka kwa minitsi 92?**

- **CG-**  Maganizo anga ndiokuti zinthuzi zitichitila ubwino chifukwa ndizothandiza kumene.
- **CG-** I think this will help us

1. **Kodi njira zimenezi tingazikhazikise bwanji mu zipatala? (tatiwuzani, tiyambe ndi gulu liti la anthu ndipo nchifukwa chani mukuganiza kuti tiyambe ndi gulu limeneli chifukwa chain?**

- **CG-** Nanga siinu mukugwira muchipatala mukungoyenera kuwona nokha, gulu latsopano mumagwira inu mupitilize.
- CG- Since you are the ones who work in the hospital, you should see for yourself which group to start with.

1. **Kodi tingapange bwanji kuti kuyezesa magazi kwa ana ndi makolo awo kapena anthu owayang’ira zikhale za chinsinsi?**

- **CG-**  Zonse amaziwa ndima dokotala mmene angapangile iwowo ndi momwemo.
- **CG-** The doctors are the ones who know the process so however they do it is okay.

1. **Kodi makolo angatengepo gawo lanji kuti njira zoyezesera magazi za Cepheid zikhazikisidwe mu chipatala chathu chino cha Mulanje?**

- **CG-**  Pamenepo ndilibe ganizo.
- **CG-** No comment

b). **Kodi makolo awuzidwe zotani ndi uphungu wotani kuti amvesese za njira zoyezesera magazi za Cepheid?**

- **CG-** Kuti amvesese ayenera kupita ku chipatala ndikukapasidwa uphungu wabwino sinanga inu achipatala mumadziwa bwino zonse.
- **CG-** to clearly understand they need to go to the hospital and get the correct counsel

1. **Kodi azibambo angatengepo gawo lanji kuti njira zoyezesera magazi za Cepheid zikhazikisidwe mu chipatala chathu chino cha Mulanje? Tingawalimbikise bwanji azibambo kuti azitenga nawo gawo mukuyezedwa magazi mu njira za Cepheid?**

- **CG-**  Azibambo azatenge gawo lomwe akuliwona iwo, apa sindikudziwa kanthu.
- **CG-** Men should take part in what they feel is right.

1. **Kodi anthu a mmudzi mwanu angamve bwanji njira zoyezesera magazi za Cepheid zitakhazikisidwa pa chipatala chanu chaching’ono mmudzi mwanu. Tingatani kuti anthu a mmudzi muno alimbikisidwe kutenga nawo mbali mu njira zoyezetsera magazi za Cepheid?**

- **CG-** Zimenezi ndizimene zimafunika padzikozo chifukwa aliyense amafuna moyo.
- **CG-** This is what is needed in this world because everyone wants life

1. **Kodi inu ndi anthu ena mma midzi mu mumakhala ndi nkhwa zanji zokhuzana ndi kulandila zosatira za magazi mwana akayezedwa kuti tiziwe kuti mwana ali ndi HIV kapena ayi?**

- **CG-** Umadandaula chifukwa umati koma mwanayo alibwinobwino kotero umafuna udziwe ngati uli bwinobwino.
- **CG-** You get worried because you want to know if the child is fine

1. **Kodi mungakhale ndi njira kapena maganizo a momwe tingathandizire kuchepesa nkhawa zokhuzana ndikulandila zotsatira za magazi mwana wayezedwa kuti tidziwe kuti mwana ali ndi HIV kapena ayi?**

- **CG-**  Kuti uchepese nkhawa ukuyenera kupita kuchipatala kuti akuthandize.
- **CG-** To reduce stress you need to go to the hospital for testing

1. **Kuchokera pa nthawi yomwe mwana wanu wayezedwa magazi kuti tidziwe kuti mwana ali ndi HIV kapena ayi, mungapilile nthawi yayitali bwanji kuti mudziwe zosatira**

- **Same day**

**Patatha masiku**

**Miyezi iwiri kapena itatu**

**Fotokozani zifukwa zomwe mungasankhile yankho limeneli**

- **CG-**  Chifukwa komwe ndachokera ndikutali, sopano ndikufuna kumva tsiku lomwero.
- **CG-** Because I stay very far and I need to know the results on the same day.

1. **Mwana wanu atayezedwa magazi, mungafune kudikila nthawi yayitali bwanji kuti mudziwe kuti mwana ali ndi HIV yomwe yimayambitsa matenda a AIDS?**

**TSiku lomwelo**

**Patatha masiku**

**Miyezi iwiri kapena itatu**

**Fotokozani zifukwa zimene mwasankhila yankho limenelo**

- **CG-**

1. **Mwana wanu atayezedwa magazi mungafune kudikila nthaawi yayitali bwanji kuti muziwe kuti mwana alibe HIV yomwe imayambitsa matenda a AIDS**

**Tsiku lomwelo**

**Patatha masiku**

**Miyezi iwiri kapena itatu**

**Fotokozani zifukwa zomwe mungasankhile yankho limenelo**

1. **kodi mungafune muwuzidwe zotani ndi uphungu otani kuti inu mupange chisankho choti mwana wanu ayezedwe magazi kuti mudziwe kuti mwana ali ndi HIV yomwe imayambitsa matenda a AIDS kapena ayi? Fotokozani bwino lomwe.**

- **CG-**  Ndingafune kupita naye kuchipatala kuti achipatala akamuwone ndikundipatsa uphungu wabwino.
- **CG-** I would want to go with the child to the hospital so the doctor can me good counselling

1. **Mungafune kuti tikufikileni mu njira yotani kuti tikuwuzeni zimezi ndikukupasani uphungu umenewu wa njira zoyezesera magazi za Cepheid ndi ?**

- **CG-** Apo ndilibe yankho
- **CG-** no comment

1. **Kodi mungathe kuwalimbikisa makolo anzanu kapena owasamalira ana kuti alore ana Awo ayezedwwe magazi kuti aziwe ngati ali ndi HIV yoyambitsa matenda a AIDS kugwilitsa ntchito Cepheid ndi ?**

- **CG-**  Eya
- **CG-** yes

**15b) Nkhawa zanu zingakhale zotani ndi mayezedwe amenewa a ndi Cepheid?**

- **CG-**  Ndilibe nkhawa iliyonse.
- **CG-** no problem with this

1. **Kodi mungamve bwanji ngati munthu wina wa mmudzi mwanu ataziwa zotsatira za magazi a mwana wanu atayezedwa kufufuza ngati ali ndi HIV kapena ayi?**

- **CG-** Ndingamve bwino chifukwa ndimangoyenera kudziwa mmene mwana wanga alili.
- **CG-** I would feel good cause I would want to know how my child is

1. **Kodi muli ndi maganizo kapena nkhawa zina zomwe mungafune kutidziwisa pa nkhani imeneyi**

- **CG-** Ndilibe ganizo kapena nkhawa ina iliyonse.
- **CG-** no comment
