# Supplementary material for: ‘If I am on ART, my new-born baby should be put on treatment immediately’: Exploring the acceptability, and appropriateness of Cepheid Xpert HIV-1 Qual assay for early infant diagnosis of HIV in Malawi
Source: PLOS Glob Public Health. 2023 Mar 10;3(3):e0001135. doi: 10.1371/journal.pgph.0001135 (PMC10021387; doi:10.1371/journal.pgph.0001135)
Supplement: S2 File — (ZIP) [file pgph.0001135.s005.zip › transcripts responses chichewa& english/DET010.docx]

**DET010_CG_F_26.7.18**

1. **Malingana ndi mmene tafotokozera za kayezedwe ka Cepheid mwana ayenera kutengedwa magazi pachara kapena pa nsempha, inu monga kholo mungamve bwanji kuti mwana wanu ayezedwe magazi kuzera njira zimezi?**

- **CG-**  Atha kumva bwino chifukwa akufuna kudziwa mmene alili mwana wawo.
- **CG-** I would feel good because I would want to know my child’s status

1. **Kwainu monga kholo la mwana wa chichepere, maganizo anu ndi otani pokhuzana ndi mayezedwe a magazi kuti tidziwe kuti mwana ali ndi HIV kapena ayi malingana ndi mmene tafotokozera za kayezedwe ka Cepheid kuti zosatira zimatuluka kwa minitsi 92?**

- **CG-**  Maganizo abwino chifukwa zithandiza kuti amve zotsatira.
- **CG-** It is a good idea because it will help us get the results.

1. **Kodi njira zimenezi tingazikhazikise bwanji mu zipatala? (tatiwuzani, tiyambe ndi gulu liti la anthu ndipo nchifukwa chani mukuganiza kuti tiyambe ndi gulu limeneli chifukwa chain?**

- **CG-**  Azimayi azipeleka mwachangu komanso azipanga azigwilizana ndi ma dotolo.
- **CG-** Women should be doing things according to the doctor

1. **Kodi tingapange bwanji kuti kuyezesa magazi kwa ana ndi makolo awo kapena anthu owayang’ira zikhale za chinsinsi?**

- **CG-** Liyambike gulu la ana chifukwa akuluakulu ndondomeko yawo ilipo kale.
- **CG-** it should start with the children because adults already have their methods.

1. **Kodi makolo angatengepo gawo lanji kuti njira zoyezesera magazi za Cepheid zikhazikisidwe mu chipatala chathu chino cha Mulanje?**

- **CG-**  Kuzipeleka kwa thunthu panjirazi.
- **CG-** Our full participation

b). **Kodi makolo awuzidwe zotani ndi uphungu wotani kuti amvesese za njira zoyezesera magazi za Cepheid?**

- **CG-** Kumvera malangizo omwe a dokotala apeleka zokhuza njirazi.
- **CG-** Listening to the advice the doctor gives about the method.

1. **Kodi azibambo angatengepo gawo lanji kuti njira zoyezesera magazi za Cepheid zikhazikisidwe mu chipatala chathu chino cha Mulanje? Tingawalimbikise bwanji azibambo kuti azitenga nawo gawo mukuyezedwa magazi mu njira za Cepheid?**

- **CG-**  Atengepo gawo chifukwa nawonso ndi anakubala, azimayi azithaso kuwafotokozera za ubwino wa Cepheid.
- **CG-** They should take part because they are also parents and their wives should tell them the importance of Cepheid

1. **Kodi anthu a mmudzi mwanu angamve bwanji njira zoyezesera magazi za Cepheid zitakhazikisidwa pa chipatala chanu chaching’ono mmudzi mwanu. Tingatani kuti anthu a mmudzi muno alimbikisidwe kutenga nawo mbali mu njira zoyezetsera magazi za Cepheid?**

- **CG-** Atha kuchilandila chifukwa sipangakhale vuto lamayendedwe.
- **CG-**They can welcome it because there would no longer be transport problems

1. **Kodi inu ndi anthu ena mma midzi mu mumakhala ndi nkhwa zanji zokhuzana ndi kulandila zosatira za magazi mwana akayezedwa kuti tiziwe kuti mwana ali ndi HIV kapena ayi?**

- **CG-**  Nkhawa imakhala poti sandipeza nako kachilombo koyambitsa Edzi.
- **CG-**Fear arises for the fact that they might be found with HIV.

1. **Kodi mungakhale ndi njira kapena maganizo a momwe tingathandizire kuchepesa nkhawa zokhuzana ndikulandila zotsatira za magazi mwana wayezedwa kuti tidziwe kuti mwana ali ndi HIV kapena ayi?**

- **CG-** Kulimba mtima ndikomwe kumafunika basi.
- **CG-** Being brave is all that is needed

1. **Kuchokera pa nthawi yomwe mwana wanu wayezedwa magazi kuti tidziwe kuti mwana ali ndi HIV kapena ayi, mungapilile nthawi yayitali bwanji kuti mudziwe zosatira**

- **Same day**

**Patatha masiku**

**Miyezi iwiri kapena itatu**

**Fotokozani zifukwa zomwe mungasankhile yankho limeneli**

- **CG-**  Chifukwa uziyenda ndi m’didi ndikuziwa zotsatira za mwanayo.
- **CG-** Because I would be walking with pride knowing the results of my child

1. **Mwana wanu atayezedwa magazi, mungafune kudikila nthawi yayitali bwanji kuti mudziwe kuti mwana ali ndi HIV yomwe yimayambitsa matenda a AIDS?**

- **Same day**

**Patatha masiku**

**Miyezi iwiri kapena itatu**

**Fotokozani zifukwa zimene mwasankhila yankho limenelo**

- **CG-**  Chifukwa uziyenda ndi m’didi ndikuziwa zotsatira za mwanayo.
- **CG-** Because you will be walking with pride after knowing the results.

1. **Mwana wanu atayezedwa magazi mungafune kudikila nthaawi yayitali bwanji kuti muziwe kuti mwana alibe HIV yomwe imayambitsa matenda a AIDS**

- **Same day**

**Patatha masiku**

**Miyezi iwiri kapena itatu**

**Fotokozani zifukwa zomwe mungasankhile yankho limenelo**

- **CG-** Chifukwa uziyenda ndi mphavu, poti waziwa zotsatira zamwana wako.
- **CG-** Because you will be walking with pride after knowing the results.

1. **kodi mungafune muwuzidwe zotani ndi uphungu otani kuti inu mupange chisankho choti mwana wanu ayezedwe magazi kuti mudziwe kuti mwana ali ndi HIV yomwe imayambitsa matenda a AIDS kapena ayi? Fotokozani bwino lomwe.**

- **CG-** Awuzidwe zaubwino oziwa mmene mwana alili nthupi kuti ali ndi HIV kapena kuti alibe.
- **CG-** We should be taught the importance of knowing a child’s HIV status.

1. **Mungafune kuti tikufikileni mu njira yotani kuti tikuwuzeni zimezi ndikukupasani uphungu umenewu wa njira zoyezesera magazi za Cepheid?**

- **CG-**  Alibe ganizo lililonse pokhudzana ndi mmene angafikilidwe.
- **CG-** no idea

1. **Kodi mungathe kuwalimbikisa makolo anzanu kapena owasamalira ana kuti alore ana Awo ayezedwwe magazi kuti aziwe ngati ali ndi HIV yoyambitsa matenda a AIDS kugwilitsa ntchito Cepheid?**

- **CG-**  Eya
- **CG-**yes

**15b) Nkhawa zanu zingakhale zotani ndi mayezedwe amenewa a Cepheid?**

- **CG-** Alibe nkhawa inailiyonse panjirazi.
- **CG-**no concerns

1. **Kodi mungamve bwanji ngati munthu wina wa mmudzi mwanu ataziwa zotsatira za magazi a mwana wanu atayezedwa kufufuza ngati ali ndi HIV kapena ayi?**

- **CG-** Kuchilandira bwinobwino chifukwa choti akufuna mwana alandire chithandizo.
- **CG-** I would be okay with it because I would want my child to receive help.

1. **Kodi muli ndi maganizo kapena nkhawa zina zomwe mungafune kutidziwisa pa nkhani imeneyi**

- **CG-**  Alibe nkhawa kapena ganizo linalililonse yokhudza njirazi.
- **CG-** no concerns
