# Supplementary material for: ‘If I am on ART, my new-born baby should be put on treatment immediately’: Exploring the acceptability, and appropriateness of Cepheid Xpert HIV-1 Qual assay for early infant diagnosis of HIV in Malawi
Source: PLOS Glob Public Health. 2023 Mar 10;3(3):e0001135. doi: 10.1371/journal.pgph.0001135 (PMC10021387; doi:10.1371/journal.pgph.0001135)
Supplement: S2 File — (ZIP) [file pgph.0001135.s005.zip › transcripts responses chichewa& english/DET011.docx]

**DET011_CG_F_26.7.18**

1. **Malingana ndi mmene tafotokozera za kayezedwe ka Cepheid, mwana ayenera kutengedwa magazi pachara kapena pa nsempha, inu monga kholo mungamve bwanji kuti mwana wanu ayezedwe magazi kuzera njira zimezi?**

- **CG-** Ndikhonza kumva bwino chifukwa ndikufuna kuziwa ngati alibwino kapena ayi kuti akuthandize mwachangu.
- **CG-** I would feel good because I want to know if my child is okay or not so he can be helped as soon as possible

1. **Kwainu monga kholo la mwana wa chichepere, maganizo anu ndi otani pokhuzana ndi mayezedwe a magazi kuti tidziwe kuti mwana ali ndi HIV kapena ayi malingana ndi mmene tafotokozera za kayezedwe ka Cepheid kuti zosatira zimatuluka kwa minitsi 92?**

- **CG-**  Zingandithandize chifukwa ndikuziwa kuti umathandizidwa pompo malingana ndi m’mene mwafotokozera kuti zotsatira zimatuluka tsiku lomwero.
- **CG-** it can help because I know that immediately we will get the needed help

1. **Kodi njira zimenezi tingazikhazikise bwanji mu zipatala? (tatiwuzani, tiyambe ndi gulu liti la anthu ndipo nchifukwa chani mukuganiza kuti tiyambe ndi gulu limeneli chifukwa chain?**

- **CG-** Ayambe ana, chifukwa choti mwana ndi mwana chifukwa suziwa chomwe chikumuvuta mwana.
- **CG-** They should start with children because as a child you do not know what is wrong with you

1. **Kodi tingapange bwanji kuti kuyezesa magazi kwa ana ndi makolo awo kapena anthu owayang’ira zikhale za chinsinsi?**

- **CG-**  Tikuyenera kupita tokha ku chipatala osati ndi gulu ayi kuti zikhale za chinsinsi.
- **CG-** We are supposed to go to the hospital alone not as group

1. **Kodi makolo angatengepo gawo lanji kuti njira zoyezesera magazi za Cepheid ndi zikhazikisidwe mu chipatala chathu chino cha Mulanje?**

- **CG-** Gawo lomwe ndingatengepo ndikufotokozera anzanga m’mudzi mwathu kuti kwabwera njira zatsopano kuti utha kudziwa zotsatira tsiku lomwero.
- **CG-** I can take part by explaining to my friends in my community about the new fast way of testing

b). **Kodi makolo awuzidwe zotani ndi uphungu wotani kuti amvesese za njira zoyezesera magazi za Cepheid ndi ?**

- **CG-** Ndilibe ganizo lililonse.
- **CG-** No comment here

1. **Kodi azibambo angatengepo gawo lanji kuti njira zoyezesera magazi za Cepheid zikhazikisidwe mu chipatala chathu chino cha Mulanje? Tingawalimbikise bwanji azibambo kuti azitenga nawo gawo mukuyezedwa magazi mu njira za Cepheid?**

- **CG-**  Azibambo angatengepo gawo pobwera ku chipatala kuti azayezetse, Azibambo tingawalimbikitse powawudza kuti zotsatira sizichedwa.
- **CG-** Men can take part by coming for the test. We need to encourage them the results come out same day.

1. **Kodi anthu a mmudzi mwanu angamve bwanji njira zoyezesera magazi za Cepheid ndi zitakhazikisidwa pa chipatala chanu chaching’ono mmudzi mwanu. Tingatani kuti anthu a mmudzi muno alimbikisidwe kutenga nawo mbali mu njira zoyezetsera magazi za Cepheid?**

- **CG-** Angakhale okondwa chifukwa azasangalala kuti njira zimenezi zakhazikitsidwa pafupi.
- **CG-**They would be happy because they would see this method has been implemented close to them.

1. **Kodi inu ndi anthu ena mma midzi mu mumakhala ndi nkhwa zanji zokhuzana ndi kulandila zosatira za magazi mwana akayezedwa kuti tiziwe kuti mwana ali ndi HIV kapena ayi?**

- **CG-**  Umakhumudwa ndikukhala ndi mantha koma ukalandira uphungu umalimbikitsidwa.
- **CG-** You get stressed and scared but when you get counsel you get motivated

1. **Kodi mungakhale ndi njira kapena maganizo a momwe tingathandizire kuchepesa nkhawa zokhuzana ndikulandila zotsatira za magazi mwana wayezedwa kuti tidziwe kuti mwana ali ndi HIV kapena ayi?**

- **CG-**  Tikuyenera kulandira uphungu wabwino.
- **CG-** We need to receive good counselling

1. **Kuchokera pa nthawi yomwe mwana wanu wayezedwa magazi kuti tidziwe kuti mwana ali ndi HIV kapena ayi, mungapilile nthawi yayitali bwanji kuti mudziwe zosatira**

- **Same day**

**Patatha masiku**

**Miyezi iwiri kapena itatu**

**Fotokozani zifukwa zomwe mungasankhile yankho limeneli**

- **CG-**  Chifukwa iweyo m’mene umapita ku chipatala umapita kuti ukadziwe zotsatira ndipo ukaziwa kuti ulinako kapena ayi, ukuyenera uthandizidwe mwachangu.
- **CG-** Because when going to the hospital you go with the mind that you will get results on the same day and get helped.

1. **Mwana wanu atayezedwa magazi, mungafune kudikila nthawi yayitali bwanji kuti mudziwe kuti mwana ali ndi HIV yomwe yimayambitsa matenda a AIDS?**

- **Same day**

**Patatha masiku**

**Miyezi iwiri kapena itatu**

**Fotokozani zifukwa zimene mwasankhila yankho limenelo**

- **CG-** Umapatsidwa uphungu ndikuthandizidwa mwachangu
- **CG-** You get counselling and help quickly.

1. **Mwana wanu atayezedwa magazi mungafune kudikila nthaawi yayitali bwanji kuti muziwe kuti mwana alibe HIV yomwe imayambitsa matenda a AIDS**

- **Same day**

**Patatha masiku**

**Miyezi iwiri kapena itatu**

**Fotokozani zifukwa zomwe mungasankhile yankho limenelo**

- **CG-** Ndilibe ganizo lililonse.
- **CG-** no comment

1. **kodi mungafune muwuzidwe zotani ndi uphungu otani kuti inu mupange chisankho choti mwana wanu ayezedwe magazi kuti mudziwe kuti mwana ali ndi HIV yomwe imayambitsa matenda a AIDS kapena ayi? Fotokozani bwino lomwe.**

- **CG-** Kuchipatala ndikomwe atha kukulangiza.
- **CG-** you would get counselling at a hospital

1. **Mungafune kuti tikufikileni mu njira yotani kuti tikuwuzeni zimezi ndikukupasani uphungu umenewu wa njira zoyezesera magazi za Cepheid?**

- **CG-** Pozera pa wailesi ndikutipatsa uphungu wabwino.
- **CG-** Using radios and giving us good counselling

1. **Kodi mungathe kuwalimbikisa makolo anzanu kapena owasamalira ana kuti alore ana Awo ayezedwwe magazi kuti aziwe ngati ali ndi HIV yoyambitsa matenda a AIDS kugwilitsa ntchito Cepheid?**

- **CG-**  Eya
- **CG-** yes

**15b) Nkhawa zanu zingakhale zotani ndi mayezedwe amenewa a Cepheid?**

- **CG-** Otenga pansempha akumachuluka kusiyana ndi apa chala ndiye ine monga kholo nkhawa yanga ndiyokuti ndimadabwa kuti koma magazi akupita kuti kapena magazi asalamo mthupi.
- **CG-** I feel that the blood taken from the vein is a lot and I am worried that my child might not have any more blood remaining in her

1. **Kodi mungamve bwanji ngati munthu wina wa mmudzi mwanu ataziwa zotsatira za magazi a mwana wanu atayezedwa kufufuza ngati ali ndi HIV kapena ayi?**

- **CG-** Ine nditha kumva bwino chifukwa ndamva m’mene alili mwana wanga.
- **CG-** I would personally feel good because I would know my child’s status

1. **Kodi muli ndi maganizo kapena nkhawa zina zomwe mungafune kutidziwisa pa nkhani imeneyi**

- **CG-**  Nkhawa yanga ili pomutenga pa nsempha imene ili yopweteka kwa mwana.
- **CG-** My concern is on the vein which blood is taken from which is painful to a child
