# Supplementary material for: ‘If I am on ART, my new-born baby should be put on treatment immediately’: Exploring the acceptability, and appropriateness of Cepheid Xpert HIV-1 Qual assay for early infant diagnosis of HIV in Malawi
Source: PLOS Glob Public Health. 2023 Mar 10;3(3):e0001135. doi: 10.1371/journal.pgph.0001135 (PMC10021387; doi:10.1371/journal.pgph.0001135)
Supplement: S2 File — (ZIP) [file pgph.0001135.s005.zip › transcripts responses chichewa& english/DET012.docx]

**DET0012_CG_F_26.7.18**

1. **Malingana ndi mmene tafotokozera za kayezedwe ka Cepheid, mwana ayenera kutengedwa magazi pachara kapena pa nsempha, inu monga kholo mungamve bwanji kuti mwana wanu ayezedwe magazi kuzera njira zimezi?**

- **CG-** Kumva bwino chifukwa akayezedwa amaziwa zotsala kuti mwana alibwanji mthupi.
- **CG-** I can feel good because when tested you know how the child is

1. **Kwainu monga kholo la mwana wa chichepere, maganizo anu ndi otani pokhuzana ndi mayezedwe a magazi kuti tidziwe kuti mwana ali ndi HIV kapena ayi malingana ndi mmene tafotokozera za kayezedwe ka Cepheid kuti zosatira zimatuluka kwa minitsi 92?**

- **CG-**  Alibe ganizo pa nkhaniyi.
- **CG-** no comment

1. **Kodi njira zimenezi tingazikhazikise bwanji mu zipatala? (tatiwuzani, tiyambe ndi gulu liti la anthu ndipo nchifukwa chani mukuganiza kuti tiyambe ndi gulu limeneli chifukwa chain?**

- **CG-** Munthu akabwera kuchipatala kuti akuzalandira mankhwala azithaso kuuzidwa zokhudzana ndi njilazi, asankha gulu la akulu kuti ndilomwe liziyambilira.
- **CG-** Reach out to people when they come to the hospital and start with the adults.

1. **Kodi tingapange bwanji kuti kuyezesa magazi kwa ana ndi makolo awo kapena anthu owayang’ira zikhale za chinsinsi?**

- **CG-**  Pakukambilana pakati pa dotolo ndi khololo osauza anthu ena.
- **CG-** It should be between the doctor and parents

1. **Kodi makolo angatengepo gawo lanji kuti njira zoyezesera magazi za Cepheid zikhazikisidwe mu chipatala chathu chino cha Mulanje?**

- **CG-** Azibwera kuchipatala kuti azamve kuti njirazi zikhazikitsidwa motani.
- **CG-** They should come to the hospital to learn more about the strategy.

b). **Kodi makolo awuzidwe zotani ndi uphungu wotani kuti amvesese za njira zoyezesera magazi za Cepheid ndi ?**

- **CG-** Awuzidwe mwachikondi komanso modekha.
- **CG-** They should be told with love and care

1. **Kodi azibambo angatengepo gawo lanji kuti njira zoyezesera magazi za Cepheid zikhazikisidwe mu chipatala chathu chino cha Mulanje? Tingawalimbikise bwanji azibambo kuti azitenga nawo gawo mukuyezedwa magazi mu njira za Cepheid?**

- **CG-**  Azibambo azitengedwa ndi azikazi awo kuti azayezetse ndikudziwa zokhudzana ndi njirazi kudzera kuchipatalako. Kuwalimbikitsa kuti akayezetse komanso kuti dziko linavuta.
- **CG-** Men must be taken together with their wives so they can get tested and know more about Cepheid at the hospital. Encouraging them to get tested and that the world has changed.

1. **Kodi anthu a mmudzi mwanu angamve bwanji njira zoyezesera magazi za Cepheid ndi zitakhazikisidwa pa chipatala chanu chaching’ono mmudzi mwanu. Tingatani kuti anthu a mmudzi muno alimbikisidwe kutenga nawo mbali mu njira zoyezetsera magazi za Cepheid?**

- **CG-** Atha kuchilandila momasuka kuti anthu aziwe zotsatira kuwalimbikitsa akabwera kuzawonana ndi dotolo.
- **CG-** They can welcome it. Encourage them to come and meet the doctor for the testing

1. **Kodi inu ndi anthu ena mma midzi mu mumakhala ndi nkhwa zanji zokhuzana ndi kulandila zosatira za magazi mwana akayezedwa kuti tiziwe kuti mwana ali ndi HIV kapena ayi?**

- **CG-**  Nkhawa sikhalapo chifukwa akazapezeka nako amaziwa kuti mwana athandizidwa.
- **CG-** I have no concerns because if found positive, the doctors would help

1. **Kodi mungakhale ndi njira kapena maganizo a momwe tingathandizire kuchepesa nkhawa zokhuzana ndikulandila zotsatira za magazi mwana wayezedwa kuti tidziwe kuti mwana ali ndi HIV kapena ayi?**

- **CG-**  Kukayezetsa kuti munthu usakhale ndi nkhawa.
- **CG-** Getting tested so that you should not have fear

1. **Kuchokera pa nthawi yomwe mwana wanu wayezedwa magazi kuti tidziwe kuti mwana ali ndi HIV kapena ayi, mungapilile nthawi yayitali bwanji kuti mudziwe zosatira**

**Tsiku lomwelo**

- **Three days**

**Miyezi iwiri kapena itatu**

**Fotokozani zifukwa zomwe mungasankhile yankho limeneli**

- **CG-** Kungofuna kudziwa patapita masiku basi.
- **CG-** No reason

1. **Mwana wanu atayezedwa magazi, mungafune kudikila nthawi yayitali bwanji kuti mudziwe kuti mwana ali ndi HIV yomwe yimayambitsa matenda a AIDS?**

**TSiku lomwelo**

- **Three days**

**Miyezi iwiri kapena itatu**

**Fotokozani zifukwa zimene mwasankhila yankho limenelo**

- **CG-**  Kuti akuchipatala akhale ndi nthawi yolongosola zotsatirazo.
- **CG-** So that the hospital can have time to explain the results

1. **Mwana wanu atayezedwa magazi mungafune kudikila nthaawi yayitali bwanji kuti muziwe kuti mwana alibe HIV yomwe imayambitsa matenda a AIDS**

- **Same day**

**Patatha masiku**

**Miyezi iwiri kapena itatu**

**Fotokozani zifukwa zomwe mungasankhile yankho limenelo**

- **CG-** Kuti udziwe mthupi mwa mwana m’mene mulili.
- **CG-** To know the health status of the child

1. **kodi mungafune muwuzidwe zotani ndi uphungu otani kuti inu mupange chisankho choti mwana wanu ayezedwe magazi kuti mudziwe kuti mwana ali ndi HIV yomwe imayambitsa matenda a AIDS kapena ayi? Fotokozani bwino lomwe.**

- **CG-** Palibe maganizo ena aliwonse omwe alinawo.
- **CG-** no idea

1. **Mungafune kuti tikufikileni mu njira yotani kuti tikuwuzeni zimezi ndikukupasani uphungu umenewu wa njira zoyezesera magazi za Cepheid?**

- **CG-**  Kungomvera ndondomeko ndi malangizo omwe akuchipatala anena.
- **CG-** following the doctor’s instructions

1. **Kodi mungathe kuwalimbikisa makolo anzanu kapena owasamalira ana kuti alore ana Awo ayezedwwe magazi kuti aziwe ngati ali ndi HIV yoyambitsa matenda a AIDS kugwilitsa ntchito Cepheid?**

- **CG-**  Eya
- **CG-** yes

**15b) Nkhawa zanu zingakhale zotani ndi mayezedwe amenewa a Cepheid?**

- **CG-**  Palibe nkhawa iliyonse angofuna njirazi zipitilire.
- **CG-** no problem here

1. **Kodi mungamve bwanji ngati munthu wina wa mmudzi mwanu ataziwa zotsatira za magazi a mwana wanu atayezedwa kufufuza ngati ali ndi HIV kapena ayi?**

- **CG-** Pamakhala povuta chifukwa munthu aakufufuzayo siwachibale bola atakhala achibale atha kukusungira chinsinsi.
- **CG-**it can be difficult because someone who you are not related to can not keep a secret at least if it was a relative

1. **Kodi muli ndi maganizo kapena nkhawa zina zomwe mungafune kutidziwisa pa nkhani imeneyi**

- **CG-**  Palibe nkhawa ina iliyonse yomwe ilipo.
- **CG-** no problem with this
