# Supplementary material for: ‘If I am on ART, my new-born baby should be put on treatment immediately’: Exploring the acceptability, and appropriateness of Cepheid Xpert HIV-1 Qual assay for early infant diagnosis of HIV in Malawi
Source: PLOS Glob Public Health. 2023 Mar 10;3(3):e0001135. doi: 10.1371/journal.pgph.0001135 (PMC10021387; doi:10.1371/journal.pgph.0001135)
Supplement: S2 File — (ZIP) [file pgph.0001135.s005.zip › transcripts responses chichewa& english/DET019.docx]

**DET019_CG_F_27.7.18**

1. **Malingana ndi mmene tafotokozera za kayezedwe ka Cepheid, mwana ayenera kutengedwa magazi pachara kapena pa nsempha, inu monga kholo mungamve bwanji kuti mwana wanu ayezedwe magazi kuzera njira zimezi?**

- **CG-** Njira imeneyi ili bwino chifukwa choti akatenga pa nsempha ngati munthu ali ndi matenda amapezeka mwachangu.
- **CG-** it is a good way because when they draw venous blood, the results are faster

1. **Kwainu monga kholo la mwana wa chichepere, maganizo anu ndi otani pokhuzana ndi mayezedwe a magazi kuti tidziwe kuti mwana ali ndi HIV kapena ayi malingana ndi mmene tafotokozera za kayezedwe ka Cephei kuti zosatira zimatuluka kwa minitsi 92?**

- **CG-**  Ukaziwa m’mene mwana alili ndibwino kwambiri chifukwa choti utha kualandira thandizo mwachangu ndikuziwa m’mene ungamusamalilire.
- **CG-** when you know your child’s status, it is very good because you might receive help as soon as possible and you will know how to care for the child.

1. **Kodi njira zimenezi tingazikhazikise bwanji mu zipatala? (tatiwuzani, tiyambe ndi gulu liti la anthu ndipo nchifukwa chani mukuganiza kuti tiyambe ndi gulu limeneli chifukwa chain?**

- **CG-** Ndikuganiza kuti tiyambe mwana chifukwa choti mwana ndinsogoleri wa m’mawa ndiye monga atsogoleri ayenera kuwateteza kuti m’mawa azakhale bwino.
- **CG-** I think we should start with children because they are the leaders of tomorrow and them being leaders need to be protected.

1. **Kodi tingapange bwanji kuti kuyezesa magazi kwa ana ndi makolo awo kapena anthu owayang’ira zikhale za chinsinsi?**

- **CG-** Sibwino kuti wina aziwe chifukwa anthu ena amakhala ochuluka nzeru akhoza kukuwonongera tsogolo.
- **CG-** it is not good that someone else knows because some people have corrupt mindsets which might destroy a child’s future

1. **Kodi makolo angatengepo gawo lanji kuti njira zoyezesera magazi za Cepheid zikhazikisidwe mu chipatala chathu chino cha Mulanje?**

- **CG-** Akuyenera kupita kuchipatala kuti akayezetse zotsatila kuzera njira za Cepheid.
- **CG-** They should go to the hospital for testing using Cepheid methods.

b). **Kodi makolo awuzidwe zotani ndi uphungu wotani kuti amvesese za njira zoyezesera magazi za Cepheid?**

- **CG-** Ife ndiolandira kumva uphunguwo kuchokera kwa inu akuchipatala ndikutiwunikira.
- **CG-** we should receive the guidance and counselling from medical personnel

1. **Kodi azibambo angatengepo gawo lanji kuti njira zoyezesera magazi za Cepheid zikhazikisidwe mu chipatala chathu chino cha Mulanje? Tingawalimbikise bwanji azibambo kuti azitenga nawo gawo mukuyezedwa magazi mu njira za Cepheid?**

- **CG-**  Azibambo akuyenera kutenga gawo kwambiri chifukwa iwowo kwambiri ndi amene amatha kukamutengera mkazi matenda, ndikuwona kuti azimayi akuyenera kutengapo gawo kiuwalimbikitsa azibambo amenewa.
- **CG-**Men must take a huge part because they are the ones that might transmit the virus to the wife and women must encourage their husbands to take part.

1. **Kodi anthu a mmudzi mwanu angamve bwanji njira zoyezesera magazi za Cepheid zitakhazikisidwa pa chipatala chanu chaching’ono mmudzi mwanu. Tingatani kuti anthu a mmudzi muno alimbikisidwe kutenga nawo mbali mu njira zoyezetsera magazi za Cepheid?**

- **CG-** Angazimve bwino chifukwa ku mmidzi kuno mayendedwe amakhala ovuta komanso anthu amakhala ndi mphwayi choncho zinthu izi zizatithandiza.
- **CG-**I would be happy with it because transportation is hard and people are lazy because of that.

1. **Kodi inu ndi anthu ena mma midzi mu mumakhala ndi nkhwa zanji zokhuzana ndi kulandila zosatira za magazi mwana akayezedwa kuti tiziwe kuti mwana ali ndi HIV kapena ayi?**

- **CG-** Ine nkhawa ndilibe chifukwa choti timafuna kuthandizidwa ngati tayezetsa ndikupezeka kuti tili ndi kachilombo, kotero ine nkhawa ndilibe pomuyezetsa mwana.
- **CG-** I have no concerns because we need to be helped and if I am found positive I would also not hesitate but get my children tested

1. **Kodi mungakhale ndi njira kapena maganizo a momwe tingathandizire kuchepesa nkhawa zokhuzana ndikulandila zotsatira za magazi mwana wayezedwa kuti tidziwe kuti mwana ali ndi HIV kapena ayi?**

- **CG-** Ndikungolimbikitsa anthu kuti asamkhale ndi nkhawa komanso azichivomereza.
- **CG-** Encouraging people not be stressed but just accept the results

1. **Kuchokera pa nthawi yomwe mwana wanu wayezedwa magazi kuti tidziwe kuti mwana ali ndi HIV kapena ayi, mungapilile nthawi yayitali bwanji kuti mudziwe zosatira**

- **Tsiku lomwelo**

**Patatha masiku**

**Miyezi iwiri kapena itatu**

**Fotokozani zifukwa zomwe mungasankhile yankho limeneli**

- **CG-**  Ndassankha tsiku lomwero chifukwa choti mwana atapezeka kuti ali ndi matenda athe kuthandizidwa mwachangu.
- **CG-** I choose the same day so that if the child is found positive, we can get assisted immediately

1. **Mwana wanu atayezedwa magazi, mungafune kudikila nthawi yayitali bwanji kuti mudziwe kuti mwana ali ndi HIV yomwe yimayambitsa matenda a AIDS?**

**TSiku lomwelo**

**Patatha masiku**

- **Miyezi iwiri kapena itatu**

**Fotokozani zifukwa zimene mwasankhila yankho limenelo**

- **CG-** Ndasankha mwezi chifukwa choti mwina a dotolo amakhala ndi nthawi yoti atenge kuwona zotsatira.
- **CG-** I choose a month because it gives time for the doctor to verify the results

1. **Mwana wanu atayezedwa magazi mungafune kudikila nthaawi yayitali bwanji kuti muziwe kuti mwana alibe HIV yomwe imayambitsa matenda a AIDS**

- **Tsiku lomwelo**

**Patatha masiku**

**Miyezi iwiri kapena itatu**

**Fotokozani zifukwa zomwe mungasankhile yankho limenelo**

- **CG-**  Chifukwa umayeneleka ukamapita kunyumba uzipita opanda nkhawa ndiwachiyembekezo.
- **CG-** I would prefer going home without any fear or hope

1. **kodi mungafune muwuzidwe zotani ndi uphungu otani kuti inu mupange chisankho choti mwana wanu ayezedwe magazi kuti mudziwe kuti mwana ali ndi HIV yomwe imayambitsa matenda a AIDS kapena ayi? Fotokozani bwino lomwe.**

- **CG-** Ngati umamukonda mwana wako sukuyenera kudikila madotolo akuwuzenso chochita.
- **CG-** if you love your child you do not have to wait for the doctors to tell you what to do

1. **Mungafune kuti tikufikileni mu njira yotani kuti tikuwuzeni zimezi ndikukupasani uphungu umenewu wa njira zoyezesera magazi za Cepheid?**

- **CG-**  Ine ndasankha kuti adokotala azitifikira m’mudzi ndikutiphunzitsa njira zimenezi.
- **CG-** I choose the doctor reaching us in our respective villages and teaching us the strategies

1. **Kodi mungathe kuwalimbikisa makolo anzanu kapena owasamalira ana kuti alore ana Awo ayezedwwe magazi kuti aziwe ngati ali ndi HIV yoyambitsa matenda a AIDS kugwilitsa ntchito Cepheid?**

- **CG-**  Eya
- **CG-**yes

**15b) Nkhawa zanu zingakhale zotani ndi mayezedwe amenewa a Cepheid?**

- **CG-** Ine ndilibe nkhawa chifukwa choti izi ndizothandiza kwa ine ndemwe ndi banja langa.
- **CG-**I have no problems with this because it is helpful to me and my family.

1. **Kodi mungamve bwanji ngati munthu wina wa mmudzi mwanu ataziwa zotsatira za magazi a mwana wanu atayezedwa kufufuza ngati ali ndi HIV kapena ayi?**

- **CG-** Singawone chovuta chilichonse chifukwa choti wina aliyense zimatha kumugwera asakuyembekezera.
- **CG-** I wouldn’t be very sad because this can happen to anyone at any time

1. **Kodi muli ndi maganizo kapena nkhawa zina zomwe mungafune kutidziwisa pa nkhani imeneyi**

- **CG-**  Palibe chilichonse chimene ndachiwona chovuta chifukwa choti ndikukonza nditsogolo la mwana wanga.
- **CG-**I see no problem because it is all for my child’s future
