# Supplementary material for: ‘If I am on ART, my new-born baby should be put on treatment immediately’: Exploring the acceptability, and appropriateness of Cepheid Xpert HIV-1 Qual assay for early infant diagnosis of HIV in Malawi
Source: PLOS Glob Public Health. 2023 Mar 10;3(3):e0001135. doi: 10.1371/journal.pgph.0001135 (PMC10021387; doi:10.1371/journal.pgph.0001135)
Supplement: S2 File — (ZIP) [file pgph.0001135.s005.zip › transcripts responses chichewa& english/DET020.docx]

**DET020_CG_F_27.7.18**

1. **Malingana ndi mmene tafotokozera za kayezedwe ka Cephei , mwana ayenera kutengedwa magazi pachara kapena pa nsempha, inu monga kholo mungamve bwanji kuti mwana wanu ayezedwe magazi kuzera njira zimezi?**

- **CG-** Njilazi ndizabwino chifukwa akufuna kuziwa mmene mwana wawo alili nthupi.
- **CG-** it is a good way because they want to know the status of the child

1. **Kwainu monga kholo la mwana wa chichepere, maganizo anu ndi otani pokhuzana ndi mayezedwe a magazi kuti tidziwe kuti mwana ali ndi HIV kapena ayi malingana ndi mmene tafotokozera za kayezedwe ka Cepheid kuti zosatira zimatuluka kwa minitsi ndipo?**

- **CG-**  Kwawo ndikuthokoza chifukwa cha mayezedwe atsopano amenewa.
- **CG-** I am just grateful to the new testing method

1. **Kodi njira zimenezi tingazikhazikise bwanji mu zipatala? (tatiwuzani, tiyambe ndi gulu liti la anthu ndipo nchifukwa chani mukuganiza kuti tiyambe ndi gulu limeneli chifukwa chain?**

- **CG-** Kumakhala kumvetsa mosiyanasiyana kuti zinthu zikafika pachipatala monga njilazi zabwerera iwowa achimvetsa , gulu la ana ndilomwe liyambilire chifukwa iwowa akuziwa kale mmene alili.
- **CG-** Start with children because it is easy for adults to know their status

1. **Kodi tingapange bwanji kuti kuyezesa magazi kwa ana ndi makolo awo kapena anthu owayang’ira zikhale za chinsinsi?**

- **CG-**  Nde pamafunika ndikuti dokotala aziwe zotsatira zamunthu asauzenso ena ayi zikhale pakati pa dotoloyo ndi wayezedwayo.
- **CG-** it is required that the doctor must know the results of the person tested and tell no one else

1. **Kodi makolo angatengepo gawo lanji kuti njira zoyezesera magazi za Cepheid zikhazikisidwe mu chipatala chathu chino cha Mulanje?**

- **CG-** Akuyenera kupita kukayezetsa, koma kuuzako apafupi zaubwino wa njilazi.
- **CG-** They should also go and get tested and tell people close to them about the new testing methods.

b). **Kodi makolo awuzidwe zotani ndi uphungu wotani kuti amvesese za njira zoyezesera magazi za Cepheid ndi ?**

- **CG-** Alibe ganizo linalililonse pa njirazi.
- **CG-** No comment on this

1. **Kodi azibambo angatengepo gawo lanji kuti njira zoyezesera magazi za Cepheid zikhazikisidwe mu chipatala chathu chino cha Mulanje? Tingawalimbikise bwanji azibambo kuti azitenga nawo gawo mukuyezedwa magazi mu njira za Cepheid?**

- **CG-**  Azibambo akuyenerakubwera ndikuyezetsa kuchipatala kuno.
- **CG-** Men should also come for testing at the hospital

1. **Kodi anthu a mmudzi mwanu angamve bwanji njira zoyezesera magazi za Cepheid zitakhazikisidwa pa chipatala chanu chaching’ono mmudzi mwanu. Tingatani kuti anthu a mmudzi muno alimbikisidwe kutenga nawo mbali mu njira zoyezetsera magazi za Cepheid?**

- **CG-** Achilandila bwino chifukwa chawafikila kuti sakuyeneraso kuyenda mtunda wawutali kuti akadziwe zotsatira zawo.
- **CG-** They can be happy with it because they do not have to travel a long distance just to get tested

1. **Kodi inu ndi anthu ena mma midzi mu mumakhala ndi nkhwa zanji zokhuzana ndi kulandila zosatira za magazi mwana akayezedwa kuti tiziwe kuti mwana ali ndi HIV kapena ayi?**

- **CG-** Siningakhale ndinkhawa chifukwa ndikhala ndikudziwe mmen nthupi mwa mwana mulili.
- **CG-** I wouldn’t be worried because I will know my status

1. **Kodi mungakhale ndi njira kapena maganizo a momwe tingathandizire kuchepesa nkhawa zokhuzana ndikulandila zotsatira za magazi mwana wayezedwa kuti tidziwe kuti mwana ali ndi HIV kapena ayi?**

- **CG-**  Maganizo anga ndi oti ukakhala ndi nkhawa ukuzichedwesa wekha malo moti upita ukaziwe mmen nthupi mwako mulili.
- CG- I think when you are worried of the results you delay yourself in knowing the status of your child

1. **Kuchokera pa nthawi yomwe mwana wanu wayezedwa magazi kuti tidziwe kuti mwana ali ndi HIV kapena ayi, mungapilile nthawi yayitali bwanji kuti mudziwe zosatira**

- **Tsiku lomwelo**

**Patatha masiku**

**Miyezi iwiri kapena itatu**

**Fotokozani zifukwa zomwe mungasankhile yankho limeneli**

- **CG-**  Chifukwa mayendedwe ndiovutilapo kuti ndifike ku chipatala kuno.
- **CG-** Because it is not easy to travel to the hospital

1. **Mwana wanu atayezedwa magazi, mungafune kudikila nthawi yayitali bwanji kuti mudziwe kuti mwana ali ndi HIV yomwe yimayambitsa matenda a AIDS?**

- **TSiku lomwelo**

**Patatha masiku**

**Miyezi iwiri kapena itatu**

**Fotokozani zifukwa zimene mwasankhila yankho limenelo**

- **CG-** Chifukwa mayendedwe ndiovutilapo kuti ndifike ku chipatala kuno.
- **CG-** Because of transportation issues

1. **Mwana wanu atayezedwa magazi mungafune kudikila nthaawi yayitali bwanji kuti muziwe kuti mwana alibe HIV yomwe imayambitsa matenda a AIDS**

- **Tsiku lomwelo**

**Patatha masiku**

**Miyezi iwiri kapena itatu**

**Fotokozani zifukwa zomwe mungasankhile yankho limenelo**

- **CG-** Chifukwa mayendedwe ndiovutilapo kuti ndifike ku chipatala kuno.
- **CG-** Difficulty in transportation to the hospital

1. **kodi mungafune muwuzidwe zotani ndi uphungu otani kuti inu mupange chisankho choti mwana wanu ayezedwe magazi kuti mudziwe kuti mwana ali ndi HIV yomwe imayambitsa matenda a AIDS kapena ayi? Fotokozani bwino lomwe.**

- **CG-**  Kungosatila uphungu omwe a dokotala apeleke wa ubwino woyezetsa.
- **CG-**Following the doctors counselling about the importance of testing

1. **Mungafune kuti tikufikileni mu njira yotani kuti tikuwuzeni zimezi ndikukupasani uphungu umenewu wa njira zoyezesera magazi za Cepheid ?**

- **CG-** Kuwafikila mwa ubwino kuti iwowo amvetsetse za ubwino wa Cepheid.
- CG- Reaching them well so they understand the importance of Cepheid

1. **Kodi mungathe kuwalimbikisa makolo anzanu kapena owasamalira ana kuti alore ana Awo ayezedwwe magazi kuti aziwe ngati ali ndi HIV yoyambitsa matenda a AIDS kugwilitsa ntchito Cepheid?**

- **CG-**  Eya
- **CG-** yes

**15b) Nkhawa zanu zingakhale zotani ndi mayezedwe amenewa a Cepheid?**

- **CG-** Alibepo nkhawa chifukwa akufuna kudziwa zotsatira zamwana.
- **CG-** no problem with this because I would want to know the results of the child

1. **Kodi mungamve bwanji ngati munthu wina wa mmudzi mwanu ataziwa zotsatira za magazi a mwana wanu atayezedwa kufufuza ngati ali ndi HIV kapena ayi?**

- **CG-** Ndikhoza kupwetekeka mu mtima chifukwa anzanga atha kumandiseka kamba ka zotsatira.
- **CG-** it would break my heart because my friends would laugh at my child.

1. **Kodi muli ndi maganizo kapena nkhawa zina zomwe mungafune kutidziwisa pa nkhani imeneyi**

- **CG-**  Alibepo ganizo lililonse chifukwa amvesesa za ubwino wa njirazi.
- **CG-** no thoughts on this, I have understood the importance of the test.
