# Supplementary material for: ‘If I am on ART, my new-born baby should be put on treatment immediately’: Exploring the acceptability, and appropriateness of Cepheid Xpert HIV-1 Qual assay for early infant diagnosis of HIV in Malawi
Source: PLOS Glob Public Health. 2023 Mar 10;3(3):e0001135. doi: 10.1371/journal.pgph.0001135 (PMC10021387; doi:10.1371/journal.pgph.0001135)
Supplement: S2 File — (ZIP) [file pgph.0001135.s005.zip › transcripts responses chichewa& english/DET021.docx]

**DET021_CG_F_27.7.18**

**She had not answer FROM QUE.1**

1. **Malingana ndi mmene tafotokozera za kayezedwe ka Cepheid, mwana ayenera kutengedwa magazi pachara kapena pa nsempha, inu monga kholo mungamve bwanji kuti mwana wanu ayezedwe magazi kuzera njira zimezi?**

- **CG-** atha kumva bwino chifukwa akufuna kuziwa mene nthupi mwa mwana mulili
- **CG-** I would feel good knowing my child’s status

1. **Kwainu monga kholo la mwana wa chichepere, maganizo anu ndi otani pokhuzana ndi mayezedwe a magazi kuti tidziwe kuti mwana ali ndi HIV kapena ayi malingana ndi mmene tafotokozera za kayezedwe ka Cepheid kuti zosatira zimatuluka kwa minitsi 92?**

- **CG-**  alibe yankho pokhudzana ndifusoli
- **CG-** No idea

1. **Kodi njira zimenezi tingazikhazikise bwanji mu zipatala? (tatiwuzani, tiyambe ndi gulu liti la anthu ndipo nchifukwa chani mukuganiza kuti tiyambe ndi gulu limeneli chifukwa chain?**

- **CG-** ana akuyenela kuyambirila chifukwa sangakwanitse kupita okha kuchipatala
- **CG-** it should start with kids because they cannot go to for the test alone

1. **Kodi tingapange bwanji kuti kuyezesa magazi kwa ana ndi makolo awo kapena anthu owayang’ira zikhale za chinsinsi?**

CG- makamaka alibe yankho pankhaniyi

CG- I have no thoughts

1. **Kodi makolo angatengepo gawo lanji kuti njira zoyezesera magazi za Cepheid zikhazikisidwe mu chipatala chathu chino cha Mulanje?**

- **CG-** gawo lomwe angalimbikise ndiloti apitilize asasiyile panjila

b). **Kodi makolo awuzidwe zotani ndi uphungu wotani kuti amvesese za njira zoyezesera magazi za Cepheid?**

- **CG-** zawavuta kuyankha
- **CG-** I have not answer

1. **Kodi azibambo angatengepo gawo lanji kuti njira zoyezesera magazi za Cepheid zikhazikisidwe mu chipatala chathu chino cha Mulanje? Tingawalimbikise bwanji azibambo kuti azitenga nawo gawo mukuyezedwa magazi mu njira za Cepheid?**

- **CG-**  azibambo alimbikise madokotala kuti asasiye njilazi, komaso kuwalimbikisa kuti atenge mwana ndikukamuyezetsa
- **CG-** Men should encourage the doctors towards these ways and encourage them to get their child tested

1. **Kodi anthu a mmudzi mwanu angamve bwanji njira zoyezesera magazi za Cepheid zitakhazikisidwa pa chipatala chanu chaching’ono mmudzi mwanu. Tingatani kuti anthu a mmudzi muno alimbikisidwe kutenga nawo mbali mu njira zoyezetsera magazi za Cepheid?**

- **CG-** Zitha kuwasangalasa chifukwa anthu mmudzi amaona kutalika pomwe zitabwela pachipatala chaching’ono sangaone kutalika
- **CG-** it can make them happy because people in the village find the distance too much so if it is available in their local health Centre it would be easier

1. **Kodi inu ndi anthu ena mma midzi mu mumakhala ndi nkhwa zanji zokhuzana ndi kulandila zosatira za magazi mwana akayezedwa kuti tiziwe kuti mwana ali ndi HIV kapena ayi?**

- **CG-** nkhawa imakhalapo kuti akapezeka ndimatenda moyo wake umakhala pachiophyezo koma ngati sanawapeze matenda kuziwa mene ungamusamalire
- **CG-** Concerns comes that if found positive, the life of my child may be in danger

1. **Kodi mungakhale ndi njira kapena maganizo a momwe tingathandizire kuchepesa nkhawa zokhuzana ndikulandila zotsatira za magazi mwana wayezedwa kuti tidziwe kuti mwana ali ndi HIV kapena ayi?**

**CG-**  Munthu oti wapezeka ndimatenda pamafunika osamusala kucheza naye, kusewela naye ndikumupasa malangizo oti aziteteze kuti asafalitse

**CG-** we should not discriminate HIV infected children by playing with them and giving them advice so that they should not spread it.

1. **Kuchokera pa nthawi yomwe mwana wanu wayezedwa magazi kuti tidziwe kuti mwana ali ndi HIV kapena ayi, mungapilile nthawi yayitali bwanji kuti mudziwe zosatira**

**Tsiku lomwelo**

**Patatha masiku**

**Miyezi iwiri kapena itatu**

**Fotokozani zifukwa zomwe mungasankhile yankho limeneli**

- **CG-** ukaziwa tsiku lomwe chimakhala chosangalasa kwambiri
- **CG-** when you get the results on the same day it makes one happier.

1. **Mwana wanu atayezedwa magazi, mungafune kudikila nthawi yayitali bwanji kuti mudziwe kuti mwana ali ndi HIV yomwe yimayambitsa matenda a AIDS?**

**TSiku lomwelo**

**Patatha masiku**

**Miyezi iwiri kapena itatu**

**Fotokozani zifukwa zimene mwasankhila yankho limenelo**

- **CG-** palibe mayankho omwe anenela tsiku lomwelo
- **CG-** no reason for picking the same day

1. **Mwana wanu atayezedwa magazi mungafune kudikila nthaawi yayitali bwanji kuti muziwe kuti mwana alibe HIV yomwe imayambitsa matenda a AIDS**

**Tsiku lomwelo**

**Patatha masiku**

**Miyezi iwiri kapena itatu**

**Fotokozani zifukwa zomwe mungasankhile yankho limenelo**

Tsiku lomwelo kuti akaziwa zotsatila ukaziwe mene ungamusamalilire

CG- Same day so that I should know how tocare for my child

1. **kodi mungafune muwuzidwe zotani ndi uphungu otani kuti inu mupange chisankho choti mwana wanu ayezedwe magazi kuti mudziwe kuti mwana ali ndi HIV yomwe imayambitsa matenda a AIDS kapena ayi? Fotokozani bwino lomwe.**

- **CG-** Ufuna kuuzidwa zoona zenizeni zamene mwana alili
- **CG-** to know the actual truth about the child’s health

1. **Mungafune kuti tikufikileni mu njira yotani kuti tikuwuzeni zimezi ndikukupasani uphungu umenewu wa njira zoyezesera magazi za Cepheid?**

- **CG- K**uwapeza mmudzi mwawo ndikuwauza mene magazi alili
- **CG-** finding them in their villages

1. **Kodi mungathe kuwalimbikisa makolo anzanu kapena owasamalira ana kuti alore ana Awo ayezedwwe magazi kuti aziwe ngati ali ndi HIV yoyambitsa matenda a AIDS kugwilitsa ntchito Cepheid?**

- **CG-**  Eya
- **CG-** yes

**15b) Nkhawa zanu zingakhale zotani ndi mayezedwe amenewa a ndi Cepheid?**

**CG-** Palibe nkwawa inailiyonse

**CG-** no concerns

1. **Kodi mungamve bwanji ngati munthu wina wa mmudzi mwanu ataziwa zotsatira za magazi a mwana wanu atayezedwa kufufuza ngati ali ndi HIV kapena ayi?**

- **CG-** Sangamve bwino chifukwa popita koyezedwa umafuna akave zotsatira okha osati ndimunthuso wina
- **CG-**I would not feel good because results are confidential

1. **Kodi muli ndi maganizo kapena nkhawa zina zomwe mungafune kutidziwisa pa nkhani imeneyi**

- **CG-** Alibe maganizo alionse kapena nkhawa
- **CG-** no concerns
