# Supplementary material for: ‘If I am on ART, my new-born baby should be put on treatment immediately’: Exploring the acceptability, and appropriateness of Cepheid Xpert HIV-1 Qual assay for early infant diagnosis of HIV in Malawi
Source: PLOS Glob Public Health. 2023 Mar 10;3(3):e0001135. doi: 10.1371/journal.pgph.0001135 (PMC10021387; doi:10.1371/journal.pgph.0001135)
Supplement: S2 File — (ZIP) [file pgph.0001135.s005.zip › transcripts responses chichewa& english/DET023.docx]

**DET023_CG_F_27.7.18**

1. **Malingana ndi mmene tafotokozera za kayezedwe ka Cepheid, mwana ayenera kutengedwa magazi pachara kapena pa nsempha, inu monga kholo mungamve bwanji kuti mwana wanu ayezedwe magazi kuzera njira zimezi?**

- **CG-** Ndingamve bwino chifukwa ndi tsogolo la mwanayo.
- **CG-** I would feel good because it is the child’s future

1. **Kwainu monga kholo la mwana wa chichepere, maganizo anu ndi otani pokhuzana ndi mayezedwe a magazi kuti tidziwe kuti mwana ali ndi HIV kapena ayi malingana ndi mmene tafotokozera za kayezedwe ka Cepheid kuti zosatira zimatuluka kwa minitsi 92?**

- **CG-**  Ndingachilandile bwino chifukwa aziziwa mmene mwana wawo alili ndikumveraso zonena za dotolo.
- **CG-** I would be okay with it and I would and I would follow Doctors advise

1. **Kodi njira zimenezi tingazikhazikise bwanji mu zipatala? (tatiwuzani, tiyambe ndi gulu liti la anthu ndipo nchifukwa chani mukuganiza kuti tiyambe ndi gulu limeneli chifukwa chain?**

- **CG-** MA dokotala akhazikitse mmene iwowo akuganizila, ayambe kuyezedwa ndi akulu chifukwa iwowo ndiamene amapatsila ana matenda.
- **CG-** It should be according to the doctors and it should start with the adults because they are the ones that transmit the virus

1. **Kodi tingapange bwanji kuti kuyezesa magazi kwa ana ndi makolo awo kapena anthu owayang’ira zikhale za chinsinsi?**

- **CG-**  Kubwera kuchipatala mayi bambo ndi mwana osatiso pakhale wina ayi.
- **CG-** Only the parents and the child should go to the hospital not accompanied by anyone else

1. **Kodi makolo angatengepo gawo lanji kuti njira zoyezesera magazi za Cepheid zikhazikisidwe mu chipatala chathu chino cha Mulanje?**

- **CG-**  zakuziwa mmene angachitire kuti njirazi zikhazikitsidwe.
- **CG-** I do not know

b). **Kodi makolo awuzidwe zotani ndi uphungu wotani kuti amvesese za njira zoyezesera magazi za Cephei?**

- **CG-** Kungowathandiza kuti amvetsetse komanso kuwuzidwa ubwino wa njirazi.
- **CG-** Helping them understand and know the importance of this method .

1. **Kodi azibambo angatengepo gawo lanji kuti njira zoyezesera magazi za Cepheid zikhazikisidwe mu chipatala chathu chino cha Mulanje? Tingawalimbikise bwanji azibambo kuti azitenga nawo gawo mukuyezedwa magazi mu njira za Cepheid?**

- **CG-**  Azibambo azithaso kubwera kuzayezetsa komanso kuzamva za uphungu ku chipatala.
- **CG-** Even men can come for testing and also get counselling from the hospital.

1. **Kodi anthu a mmudzi mwanu angamve bwanji njira zoyezesera magazi za Cepheid zitakhazikisidwa pa chipatala chanu chaching’ono mmudzi mwanu. Tingatani kuti anthu a mmudzi muno alimbikisidwe kutenga nawo mbali mu njira zoyezetsera magazi za Cepheid?**

- **CG-** Anthu atha kuchilandira bwino chifukwa akufuna atetezedwe kumatendawa
- **CG-** other people might receive it well because they would want to be protected from the virus.

1. **Kodi inu ndi anthu ena mma midzi mu mumakhala ndi nkhwa zanji zokhuzana ndi kulandila zosatira za magazi mwana akayezedwa kuti tiziwe kuti mwana ali ndi HIV kapena ayi?**

- **ANS-**  Sakhala ndi nkhawa chifukwa akayezedwa amaziwa kuti adotolo atha kuwateteza malingana ndi zosatirazo.
- **CG-** I would not be worried because I know I would be helped regardless of the results

1. **Kodi mungakhale ndi njira kapena maganizo a momwe tingathandizire kuchepesa nkhawa zokhuzana ndikulandila zotsatira za magazi mwana wayezedwa kuti tidziwe kuti mwana ali ndi HIV kapena ayi?**

- **CG-**  Palibepo nkhwa chifukwa ukapezeka nawo matenda umalandira chithandizo, nde akuwona kuti kupita kuchipatalako ndi njira imene ingathese nkhawa.
- CG- I would not be worried because when you are found with the virus you get the requried help

1. **Kuchokera pa nthawi yomwe mwana wanu wayezedwa magazi kuti tidziwe kuti mwana ali ndi HIV kapena ayi, mungapilile nthawi yayitali bwanji kuti mudziwe zosatira**

- **Tsiku lomwelo**

**Patatha masiku**

**Miyezi iwiri kapena itatu**

**Fotokozani zifukwa zomwe mungasankhile yankho limeneli**

- **CG-**  Ndizafuna kuziwa tsiku lomwero chifukwa choti ndichimene ndabwerera kuno kuti ndimve, kuti mwana wanga athandizidwe ngati atapezeka ndi kachilombo.
- **CG-** Same day because I want my child to be assisted immediately

1. **Mwana wanu atayezedwa magazi, mungafune kudikila nthawi yayitali bwanji kuti mudziwe kuti mwana ali ndi HIV yomwe yimayambitsa m atenda a AIDS?**

- **TSiku lomwelo**

**Patatha masiku**

**Miyezi iwiri kapena itatu**

**Fotokozani zifukwa zimene mwasankhila yankho limenelo**

- **CG-**  Ndasankha tsiku lomwero chifukwa kwathu ndikutali sindingathe kubwerelanso.
- **CG-** I choose the same day because I stay very far from the hospital

1. **Mwana wanu atayezedwa magazi mungafune kudikila nthaawi yayitali bwanji kuti muziwe kuti mwana alibe HIV yomwe imayambitsa matenda a AIDS**

- **Tsiku lomwelo**

**Patatha masiku**

**Miyezi iwiri kapena itatu**

**Fotokozani zifukwa zomwe mungasankhile yankho limenelo**

1. **kodi mungafune muwuzidwe zotani ndi uphungu otani kuti inu mupange chisankho choti mwana wanu ayezedwe magazi kuti mudziwe kuti mwana ali ndi HIV yomwe imayambitsa matenda a AIDS kapena ayi? Fotokozani bwino lomwe.**

- **CG-**  Apatsidwe uphungu woti ngati mwana wapezeka ndi HIV alandile chithandizo.
- **CG-** They should receive counselling of what to do if the child is found Positive

1. **Mungafune kuti tikufikileni mu njira yotani kuti tikuwuzeni zimezi ndikukupasani uphungu umenewu wa njira zoyezesera magazi za Cepheid?**

- **CG-** Alibe ganizo linalililonse
- **CG-** no comment on this

1. **Kodi mungathe kuwalimbikisa makolo anzanu kapena owasamalira ana kuti alore ana Awo ayezedwwe magazi kuti aziwe ngati ali ndi HIV yoyambitsa matenda a AIDS kugwilitsa ntchito Cepheid?**

- **CG-**  Eya
- **CG-** yes

**15b) Nkhawa zanu zingakhale zotani ndi mayezedwe amenewa a Cepheid?**

- **CG-** Alibe nkhawa ina iliyonse.
- **CG-**has no problem with this

1. **Kodi mungamve bwanji ngati munthu wina wa mmudzi mwanu ataziwa zotsatira za magazi a mwana wanu atayezedwa kufufuza ngati ali ndi HIV kapena ayi?**

- **CG-** sangamve bwino chifukwa zikufunika zikhala pakati pa dokotala ndi iwowo.
- **CG-** I would not feel good because it is only supposed between the doctor and me.

1. **Kodi muli ndi maganizo kapena nkhawa zina zomwe mungafune kutidziwisa pa nkhani imeneyi**

- **CG-**  Nkhawa ndiyoti zotsatira sanawuzidwe chiyezesereni cha dzulo
- **CG-** I have not yet received my results from yesterdays test
