# Supplementary material for: ‘If I am on ART, my new-born baby should be put on treatment immediately’: Exploring the acceptability, and appropriateness of Cepheid Xpert HIV-1 Qual assay for early infant diagnosis of HIV in Malawi
Source: PLOS Glob Public Health. 2023 Mar 10;3(3):e0001135. doi: 10.1371/journal.pgph.0001135 (PMC10021387; doi:10.1371/journal.pgph.0001135)
Supplement: S2 File — (ZIP) [file pgph.0001135.s005.zip › transcripts responses chichewa& english/DET024.docx]

**DET024_CG_F_27.7.18**

1. **Malingana ndi mmene tafotokozera za kayezedwe ka Cepheid, mwana ayenera kutengedwa magazi pachara kapena pa nsempha, inu monga kholo mungamve bwanji kuti mwana wanu ayezedwe magazi kuzera njira zimezi?**

- **CG-** Mwana ayenera kuyezedwa pa nsempha chifukwa choti ndi mwana nde pachala sangapeze magazi ambiri.
- **CG-** Blood sample of a child need to been drawn from the vein because you can not find enough blood on the finger

1. **Kwainu monga kholo la mwana wa chichepere, maganizo anu ndi otani pokhuzana ndi mayezedwe a magazi kuti tidziwe kuti mwana ali ndi HIV kapena ayi malingana ndi mmene tafotokozera za kayezedwe ka Cepheid kuti zosatira zimatuluka kwa minitsi 92?**

- **CG-**  Mwana ayenera kuyezedwa pompo ndikuziwa zosatira chifukwa choti kudikila mpaka mwanayo afike zaka zingapo zingakhale zovuta chifukwa akhonza kuyamba kudwara.
- **CG-** the child should be immediately tested and results should be out because waiting for the child to grow would be a bit hard because he/she might get sick

1. **Kodi njira zimenezi tingazikhazikise bwanji mu zipatala? (tatiwuzani, tiyambe ndi gulu liti la anthu ndipo nchifukwa chani mukuganiza kuti tiyambe ndi gulu limeneli chifukwa chain?**

- **CG-**  Uthengawu ukuyenera kufikila makolo, mujyambile makolo chifukwa makolo ndiamene angathe kutenga mwana ndikumupitisa kuchipatala.
- **CG-** this message must reach parents and we should start with parents because they are the ones who can take their child to the hospital

1. **Kodi tingapange bwanji kuti kuyezesa magazi kwa ana ndi makolo awo kapena anthu owayang’ira zikhale za chinsinsi?**

- **CG-**  Kungomuwuza kholo la mwana opanda wina aliyense komanso a dokotala akuyenera kusunga chinsinsi.
- **CG-** Telling the child’s parent and no one else

1. **Kodi makolo angatengepo gawo lanji kuti njira zoyezesera magazi za Cepheid zikhazikisidwe mu chipatala chathu chino cha Mulanje?**

- **CG-** Uthengawu kungowumvetsetsa komanso kuwuza anzathu.
- **CG-** understanding and telling our friends the message

b). **Kodi makolo awuzidwe zotani ndi uphungu wotani kuti amvesese za njira zoyezesera magazi za Cepheid ndi ?**

- **CG-** Atiwuze njira zomwe tingagwiritse ntchito komanso atipase ndondomeko zosamalilira ana.
- **CG-** should tell us the ways which we can use and also on how we can care for our child.

1. **Kodi azibambo angatengepo gawo lanji kuti njira zoyezesera magazi za Cepheid zikhazikisidwe mu chipatala chathu chino cha Mulanje? Tingawalimbikise bwanji azibambo kuti azitenga nawo gawo mukuyezedwa magazi mu njira za Cepheid?**

- **CG-**  Tikuyenera kuwafotokozera ndikuwawuza azipita kukayezetsa, pokayezetsa magazi tikuyenera kunyamulana ndi azibambo ndikuwawuza zakuwopsa kwa matendawa.
- **CG-** we need to explain to them and tell them to get tested and when getting tested we need to go with our husbands and tell them the dangers of HIV.

1. **Kodi anthu a mmudzi mwanu angamve bwanji njira zoyezesera magazi za Cepheid ndi zitakhazikisidwa pa chipatala chanu chaching’ono mmudzi mwanu. Tingatani kuti anthu a mmudzi muno alimbikisidwe kutenga nawo mbali mu njira zoyezetsera magazi za Cepheid?**

- **CG-** Ndilibe ganizo lililonse.
- **CG-** No thoughts on this

1. **Kodi inu ndi anthu ena mma midzi mu mumakhala ndi nkhwa zanji zokhuzana ndi kulandila zosatira za magazi mwana akayezedwa kuti tiziwe kuti mwana ali ndi HIV kapena ayi?**

- **CG-**  Ndimakhala ndi nkhawa chifukwa chokuti timakhala sitinamve kanthu .
- **CG-** I have concerns because I have never done this test for my child

**Kodi mungakhale ndi njira kapena maganizo a momwe tingathandizire kuchepesa nkhawa zokhuzana ndikulandila zotsatira za magazi mwana wayezedwa kuti tidziwe kuti mwana ali ndi HIV kapena ayi?**

- **CG-**  Aliyense amakhala ndi nkhawa, kuthesa kwake ndikudziwa zotsatira basi.
- **CG-** Everyone has fear and the only way to get lid of it is knowing the results

1. **Kuchokera pa nthawi yomwe mwana wanu wayezedwa magazi kuti tidziwe kuti mwana ali ndi HIV kapena ayi, mungapilile nthawi yayitali bwanji kuti mudziwe zosatira**

- **Tsiku lomwelo**

**Patatha masiku**

**Miyezi iwiri kapena itatu**

**Fotokozani zifukwa zomwe mungasankhile yankho limeneli**

- **CG-**  Ngati wapezeka nako ukuyenera kuvomereza mwachangu ndikumuteteza.
- **CG-** if found positive, you need to take it in quickly and protect the child

1. **Mwana wanu atayezedwa magazi, mungafune kudikila nthawi yayitali bwanji kuti mudziwe kuti mwana ali ndi HIV yomwe yimayambitsa matenda a AIDS?**

- **TSiku lomwelo**

**Patatha masiku**

**Miyezi iwiri kapena itatu**

**Fotokozani zifukwa zimene mwasankhila yankho limenelo**

- **CG-** Nditsiku limene ndikuyembekezera zotsatira kuti ndiziwe kuti thupi la mwan lilibwanji.
- **CG-** it is the day I am expecting the results of my child

1. **Mwana wanu atayezedwa magazi mungafune kudikila nthaawi yayitali bwanji kuti muziwe kuti mwana alibe HIV yomwe imayambitsa matenda a AIDS**

- **Tsiku lomwelo**

**Patatha masiku**

**Miyezi iwiri kapena itatu**

**Fotokozani zifukwa zomwe mungasankhile yankho limenelo**

- **CG-**  Ndifuna ndiziwe ngati alinako kapena ayi ndikumuteteza.
- **CG-** I need know if my child is positive or not

1. **kodi mungafune muwuzidwe zotani ndi uphungu otani kuti inu mupange chisankho choti mwana wanu ayezedwe magazi kuti mudziwe kuti mwana ali ndi HIV yomwe imayambitsa matenda a AIDS kapena ayi? Fotokozani bwino lomwe.**

- **CG-** Kuwudzidwa zakuwopsa kwa matenda amenewa.
- **CG-** Being told the dangers of this disease

1. **Mungafune kuti tikufikileni mu njira yotani kuti tikuwuzeni zimezi ndikukupasani uphungu umenewu wa njira zoyezesera magazi za Cepheid?**

- **CG-**  kaya kuzera kusikero kaya kutiyendera mukhoza kutifikila.
- **CG-** At antenatal clinic or visiting us

1. **Kodi mungathe kuwalimbikisa makolo anzanu kapena owasamalira ana kuti alore ana Awo ayezedwwe magazi kuti aziwe ngati ali ndi HIV yoyambitsa matenda a AIDS kugwilitsa ntchito Cepheid?**

- **CG-**  Eya
- **CG-** Yes

**15b) Nkhawa zanu zingakhale zotani ndi mayezedwe amenewa a Cepheid?**

- **CG-** Pakuphonyesa kumuyeza mwana chifukwa amataya magazi ambiri, zimenezi zingatipatse nkhawa.
- **CG-** if mistakes are made the child might lose a lot of blood which strikes fear in our hearts

1. **Kodi mungamve bwanji ngati munthu wina wa mmudzi mwanu ataziwa zotsatira za magazi a mwana wanu atayezedwa kufufuza ngati ali ndi HIV kapena ayi?**

- **CG-** Zingakhale zowawa chifukwa aliyense amafuna chinsinsi.
- **CG-** it would be painful because everyone wants to keep this a secret

1. **Kodi muli ndi maganizo kapena nkhawa zina zomwe mungafune kutidziwisa pa nkhani imeneyi**

- **CG-**  Ndilibe nkhawa iliyonse pa nkhani imeneyi.
- **CG-** no problem
