# Supplementary material for: ‘If I am on ART, my new-born baby should be put on treatment immediately’: Exploring the acceptability, and appropriateness of Cepheid Xpert HIV-1 Qual assay for early infant diagnosis of HIV in Malawi
Source: PLOS Glob Public Health. 2023 Mar 10;3(3):e0001135. doi: 10.1371/journal.pgph.0001135 (PMC10021387; doi:10.1371/journal.pgph.0001135)
Supplement: S2 File — (ZIP) [file pgph.0001135.s005.zip › transcripts responses chichewa& english/DET025.docx]

**DET025_CG_F_27.7.18**

1. **Malingana ndi mmene tafotokozera za kayezedwe ka Cepheid, mwana ayenera kutengedwa magazi pachara kapena pa nsempha, inu monga kholo mungamve bwanji kuti mwana wanu ayezedwe magazi kuzera njira zimezi?**

- **CG-** Angamve bwino chifukwa akupanga tsogolo la mwana wawo.
- **CG-** I would be okay with it because it is my child’s future

1. **Kwainu monga kholo la mwana wa chichepere, maganizo anu ndi otani pokhuzana ndi mayezedwe a magazi kuti tidziwe kuti mwana ali ndi HIV kapena ayi malingana ndi mmene tafotokozera za kayezedwe ka Cepheid kuti zosatira zimatuluka kwa minitsi 92?**

- **CG-**  Maganizo awo ndi abwino chifukwa zithandiza kuziwa ngati mwana ali ndi matenda kapena ayi.
- **CG-** it is a good idea because It will help me know if my child has the virus or not.

1. **Kodi njira zimenezi tingazikhazikise bwanji mu zipatala? (tatiwuzani, tiyambe ndi gulu liti la anthu ndipo nchifukwa chani mukuganiza kuti tiyambe ndi gulu limeneli chifukwa chain?**

- **CG-**  Tiwadziwise kudzera ku ma Ward a ana ndikuwafotokozera ubwino wa njirazi, komanso tiyambilire ana chifukwa mwana atha kutengera mu njira zosiyanasiyana sinanga mwana ndi mwana.
- **CG-** Using peadiatrics wards, explain the importance of the methods. Start with children

1. **Kodi tingapange bwanji kuti kuyezesa magazi kwa ana ndi makolo awo kapena anthu owayang’ira zikhale za chinsinsi?**

- **CG-** Chinsinsi chili ndi mwini mwana.
- **CG-** The secret is between the mother and child.

1. **Kodi makolo angatengepo gawo lanji kuti njira zoyezesera magazi za Cepheid zikhazikisidwe mu chipatala chathu chino cha Mulanje?**

- **CG-** Atenge gawo powayezetsera anawa kuzera munjira za Cepheid kapena .
- **CG-** Taking part by getting their children tested using Cepheid

b). **Kodi makolo awuzidwe zotani ndi uphungu wotani kuti amvesese za njira zoyezesera magazi za Cepheid?**

- **CG-** Alandile uphungu wowalimbikitsa zaubwino wa njirazi.
- **CG-** Counselling concerning the importance of this method

1. **Kodi azibambo angatengepo gawo lanji kuti njira zoyezesera magazi za Cepheid zikhazikisidwe mu chipatala chathu chino cha Mulanje? Tingawalimbikise bwanji azibambo kuti azitenga nawo gawo mukuyezedwa magazi mu njira za Cepheid?**

- **CG-**  Akafuna kuyezetsa azibwera kuchipatala kuzaziwa mmene alili nthupi.
- **CG-** When they need to be tested, they should go to the hospital

1. **Kodi anthu a mmudzi mwanu angamve bwanji njira zoyezesera magazi za Cepheid zitakhazikisidwa pa chipatala chanu chaching’ono mmudzi mwanu. Tingatani kuti anthu a mmudzi muno alimbikisidwe kutenga nawo mbali mu njira zoyezetsera magazi za Cepheid?**

- **CG-** Amva bwino chifukwa iwonso akuyenera kuyezetsa sinanga zafika pafupi, kuwawuza misonkhano kuti akayezetse.
- **CG-** They would like it and they would be motivated to go for the test

1. **Kodi inu ndi anthu ena mma midzi mu mumakhala ndi nkhwa zanji zokhuzana ndi kulandila zosatira za magazi mwana akayezedwa kuti tiziwe kuti mwana ali ndi HIV kapena ayi?**

- **CG-**  Nkhawa ikhalapo makamaka kuti zotsatila zibwere zotani.
- **CG-** I would be worried because I am un sure of the results

1. **Kodi mungakhale ndi njira kapena maganizo a momwe tingathandizire kuchepesa nkhawa zokhuzana ndikulandila zotsatira za magazi mwana wayezedwa kuti tidziwe kuti mwana ali ndi HIV kapena ayi?**

- **CG-**  Kulimba mtima ngati kuti zotsatira zikuwonetsa kuti ali nako amusamalire mwanayo.
- **CG-** We should just be strong because if found positive we would get assisted

1. **Kuchokera pa nthawi yomwe mwana wanu wayezedwa magazi kuti tidziwe kuti mwana ali ndi HIV kapena ayi, mungapilile nthawi yayitali bwanji kuti mudziwe zosatira**

- **Tsiku lomwelo**

**Patatha masiku**

**Miyezi iwiri kapena itatu**

**Fotokozani zifukwa zomwe mungasankhile yankho limeneli**

- **CG-**  Chifukwa choti azilimba mtima ndizosatilazo.
- **CG-** Because I want to know and accept the results

1. **Mwana wanu atayezedwa magazi, mungafune kudikila nthawi yayitali bwanji kuti mudziwe kuti mwana ali ndi HIV yomwe yimayambitsa matenda a AIDS?**

- **TSiku lomwelo**

**Patatha masiku**

**Miyezi iwiri kapena itatu**

**Fotokozani zifukwa zimene mwasankhila yankho limenelo**

- **CG-** Kupangila mayendedwe nde ndibwino kuti adziwe tsiku lomwero.
- **CG-** I stay very far, so I want to know today

1. **Mwana wanu atayezedwa magazi mungafune kudikila nthaawi yayitali bwanji kuti muziwe kuti mwana alibe HIV yomwe imayambitsa matenda a AIDS**

- **Tsiku lomwelo**

**Patatha masiku**

**Miyezi iwiri kapena itatu**

**Fotokozani zifukwa zomwe mungasankhile yankho limenelo**

- **CG-** Kupangila mayendedwe nde ndibwino kuti adziwe tsiku lomwero.
- **CG**- Considering transport issues same day results would be better

1. **kodi mungafune muwuzidwe zotani ndi uphungu otani kuti inu mupange chisankho choti mwana wanu ayezedwe magazi kuti mudziwe kuti mwana ali ndi HIV yomwe imayambitsa matenda a AIDS kapena ayi? Fotokozani bwino lomwe.**

- **CG-** Kuwalimbikitsa kuti alandire chithandizo akapezeka ndi matenda kapena kuwathandiza ndi malangizo ngati alibe kuti akamusamale bwanji mwana.
- **CG-** How to take care of child if positive or negative

1. **Mungafune kuti tikufikileni mu njira yotani kuti tikuwuzeni zimezi ndikukupasani uphungu umenewu wa njira zoyezesera magazi za Cepheid?**

- **CG-**  Njira ina iliyonse yomwe achipatatala akhonza.
- **CG-** Any means the medical team might use

1. **Kodi mungathe kuwalimbikisa makolo anzanu kapena owasamalira ana kuti alore ana Awo ayezedwwe magazi kuti aziwe ngati ali ndi HIV yoyambitsa matenda a AIDS kugwilitsa ntchito Cepheid?**

- **CG-**  Eya
- **CG-** yes

**15b) Nkhawa zanu zingakhale zotani ndi mayezedwe amenewa a Cepheid?**

- **CG-**  Nkhawa zibwera kuti potenga magazi pansempha kuti sampweteka mwanayo.
- **CG-** My concern is that when drawing blood, it might hurt my child

1. **Kodi mungamve bwanji ngati munthu wina wa mmudzi mwanu ataziwa zotsatira za magazi a mwana wanu atayezedwa kufufuza ngati ali ndi HIV kapena ayi?**

- **CG-** Sangamve bwino kuti munthu winayo akamve azilengeza za mmene mwana wawoyo alili.
- **CG-** Wouldn’t like it seeing another person talking about my child’s HIV status

1. **Kodi muli ndi maganizo kapena nkhawa zina zomwe mungafune kutidziwisa pa nkhani imeneyi**

- **CG-** Nkhawa ndiyoti sakudziwa mmene mwana wawo alili nthupi makamaka zotsatira za mwanayo ndi zomwe zikuwapasa nkhawa.
- **CG-** I only worried because I do not know the status of my child.
