# Supplementary material for: ‘If I am on ART, my new-born baby should be put on treatment immediately’: Exploring the acceptability, and appropriateness of Cepheid Xpert HIV-1 Qual assay for early infant diagnosis of HIV in Malawi
Source: PLOS Glob Public Health. 2023 Mar 10;3(3):e0001135. doi: 10.1371/journal.pgph.0001135 (PMC10021387; doi:10.1371/journal.pgph.0001135)
Supplement: S2 File — (ZIP) [file pgph.0001135.s005.zip › transcripts responses chichewa& english/DET026.docx]

**DET026_CG_F_27.7.18**

1. **Malingana ndi mmene tafotokozera za kayezedwe ka Cepheid, mwana ayenera kutengedwa magazi pachara kapena pa nsempha, inu monga kholo mungamve bwanji kuti mwana wanu ayezedwe magazi kuzera njira zimezi?**

- **CG-** Sindingamve bwino ndi njira yapansempha komabe ndikufuna kuti mwana athandizidwe, ndiye ndikungoyenera kuvomereza.
- **CG-** I wouldn’t feel good with the ways but I still want my child to be helped so I would comply.

1. **Kwainu monga kholo la mwana wa chichepere, maganizo anu ndi otani pokhuzana ndi mayezedwe a magazi kuti tidziwe kuti mwana ali ndi HIV kapena ayi malingana ndi mmene tafotokozera za kayezedwe ka Cepheid kuti zosatira zimatuluka kwa minitsi 92?**

- **CG-**  Ndiyabwino chifukwa chokuti zinthu ziziyenda mwansanga sichifukwa chokuti nthawi yakale kunalibe njirazi.
- **CG-** It is good because things are moving faster rather than in the old days when we didn’t have this way.

1. **Kodi njira zimenezi tingazikhazikise bwanji mu zipatala? (tatiwuzani, tiyambe ndi gulu liti la anthu ndipo nchifukwa chani mukuganiza kuti tiyambe ndi gulu limeneli chifukwa chain?**

- **CG-** Tizikhazikise kuti anthu azilandila zinthu mwansanga, tiyambilire chifukwa choti mwana akhoza kumanyentchera ife osaziwa kuti mwana akudwara chain.
- **CG-** We should establish that people should be receiving things quickly and start with children because we would just see signs of malnutrition not knowing what may be wrong with them.

1. **Kodi tingapange bwanji kuti kuyezesa magazi kwa ana ndi makolo awo kapena anthu owayang’ira zikhale za chinsinsi?**

- **CG-**  Munthu mukamafuna kumuyeza muzimutengera kukachipinda.
- **CG-** Testing should take place in a confined room

1. **Kodi makolo angatengepo gawo lanji kuti njira zoyezesera magazi za Cepheid zikhazikisidwe mu chipatala chathu chino cha Mulanje?**

- **CG-**  Kuwalimbikitsa anthu obweresa chithandizo kuti chibwere msanga.
- **CG-** Encouraging the helping organizations to come and assist

b). **Kodi makolo awuzidwe zotani ndi uphungu wotani kuti amvesese za njira zoyezesera magazi za Cepheid ?**

- **CG-** Akuyenera kutilangiza njira zotsatilira njirazi.
- **CG-** we need to receive counselling.

1. **Kodi azibambo angatengepo gawo lanji kuti njira zoyezesera magazi za Cepheid zikhazikisidwe mu chipatala chathu chino cha Mulanje? Tingawalimbikise bwanji azibambo kuti azitenga nawo gawo mukuyezedwa magazi mu njira za Cepheid?**

- **CG-**  Akamva azibwera kuzayezetsa mwachangu, ndikuziwa mmene thupi mwawo mmene muliri.
- **CG-** when they understand they should also come for testing to know their bodies status

1. **Kodi anthu a mmudzi mwanu angamve bwanji njira zoyezesera magazi za Cepheid zitakhazikisidwa pa chipatala chanu chaching’ono mmudzi mwanu. Tingatani kuti anthu a mmudzi muno alimbikisidwe kutenga nawo mbali mu njira zoyezetsera magazi za Cepheid?**

- **CG-** Amva bwino chifukwa chipatala chili pafupi ndiye sangayende nthawi yayitali kuti akaziwe kuti mthupi mwawo muli bwanji, kuwawunikila ubwino oyezetsa.
- **CG-** They would like it and they would be encouraged to come for the test

1. **Kodi inu ndi anthu ena mma midzi mu mumakhala ndi nkhwa zanji zokhuzana ndi kulandila zosatira za magazi mwana akayezedwa kuti tiziwe kuti mwana ali ndi HIV kapena ayi?**

- **CG-**  Ine nkhawa sindingakhale nayo chifukwa zinthuzoi ndizothandiza ife tomwe.
- **CG-** I have no concerns because it is helping us the people.

1. **Kodi mungakhale ndi njira kapena maganizo a momwe tingathandizire kuchepesa nkhawa zokhuzana ndikulandila zotsatira za magazi mwana wayezedwa kuti tidziwe kuti mwana ali ndi HIV kapena ayi?**

- **CG-** Kumakhala mmagulu kumakambilana za muliri wavutawu, kuti tigonjetse ndikuchita masewero kuti apezeka nawo asamadandaure.
- **CG-** Forming groups where we can discuss issues of HIV/AIDS

1. **Kuchokera pa nthawi yomwe mwana wanu wayezedwa magazi kuti tidziwe kuti mwana ali ndi HIV kapena ayi, mungapilile nthawi yayitali bwanji kuti mudziwe zosatira**

- **Tsiku lomwelo**

**Patatha masiku**

**Miyezi iwiri kapena itatu**

**Fotokozani zifukwa zomwe mungasankhile yankho limeneli**

- **CG-**  Chifukwa choti zotsatoira zikatuluka pompo nkhawa sukhala nayo.
- **CG-** Because I would not have worries on the same day results.

1. **Mwana wanu atayezedwa magazi, mungafune kudikila nthawi yayitali bwanji kuti mudziwe kuti mwana ali ndi HIV yom we yimayambitsa matenda a AIDS?**

- **TSiku lomwelo**

**Patatha masiku**

**Miyezi iwiri kapena itatu**

**Fotokozani zifukwa zimene mwasankhila yankho limenelo**

- **CG-**  Ukuyenera kuziwa tsiku lomwelo kuti ulandile thandizo ngati pakuyenera kuteero.
- **CG-** you need to know on the same day so u can receive help

1. **Mwana wanu atayezedwa magazi mungafune kudikila nthaawi yayitali bwanji kuti muziwe kuti mwana alibe HIV yomwe imayambitsa matenda a AIDS**

- **Tsiku lomwelo**

**Patatha masiku**

**Miyezi iwiri kapena itatu**

**Fotokozani zifukwa zomwe mungasankhile yankho limenelo**

- **CG-**  Ndasankha tsiku lomwero chifukwa choti mtima umagunda ukakhala sunamve zosatira.
- **CG-** Same day because I would not be at ease if I was to wait longer

1. **kodi mungafune muwuzidwe zotani ndi uphungu otani kuti inu mupange chisankho choti mwana wanu ayezedwe magazi kuti mudziwe kuti mwana ali ndi HIV yomwe imayambitsa matenda a AIDS kapena ayi? Fotokozani bwino lomwe.**

- **CG-** Kuwonana ndi adokotala kuti akulangize njira zabwino zotsatira.
- **CG-** Meeting the doctor to tell us the correct order to follow things.

1. **Mungafune kuti tikufikileni mu njira yotani kuti tikuwuzeni zimezi ndikukupasani uphungu umenewu wa njira zoyezesera magazi za Cepheid?**

- **CG-**  Popangisa msonkhano ndikutiwuza kudzera mu wailesi.
- **CG-** by hosting conventions and using radios.

1. **Kodi mungathe kuwalimbikisa makolo anzanu kapena owasamalira ana kuti alore ana Awo ayezedwwe magazi kuti aziwe ngati ali ndi HIV yoyambitsa matenda a AIDS kugwilitsa ntchito Cepheid?**

- **CG-**  Eya
- **CG-** yes

**15b) Nkhawa zanu zingakhale zotani ndi mayezedwe amenewa a Cepheid?**

- **CG-**  Ndilibe nkhawa ikliyonse pa nkhani imeneyi.
- **CG-** no problem with this issue.

1. **Kodi mungamve bwanji ngati munthu wina wa mmudzi mwanu ataziwa zotsatira za magazi a mwana wanu atayezedwa kufufuza ngati ali ndi HIV kapena ayi?**

- **CG-** Ndingadandaule chifukwa munthu umakhala ndi chitozo
- **CG-** I would be sad because I would become a laughing stock

1. **Kodi muli ndi maganizo kapena nkhawa zina zomwe mungafune kutidziwisa pa nkhani imeneyi**

- **CG-**  Ndilibe nkhawa kwanga ndikungonyadila kuti apitilize.
- **CG-** I have no problem with this but rather joy so they should continue.
