# Supplementary material for: ‘If I am on ART, my new-born baby should be put on treatment immediately’: Exploring the acceptability, and appropriateness of Cepheid Xpert HIV-1 Qual assay for early infant diagnosis of HIV in Malawi
Source: PLOS Glob Public Health. 2023 Mar 10;3(3):e0001135. doi: 10.1371/journal.pgph.0001135 (PMC10021387; doi:10.1371/journal.pgph.0001135)
Supplement: S2 File — (ZIP) [file pgph.0001135.s005.zip › transcripts responses chichewa& english/DET027.docx]

**DET027_CG_F_27_30.7.18**

1. **Malingana ndi mmene tafotokozera za kayezedwe ka Cepheid, mwana ayenera kutengedwa magazi pachara kapena pa nsempha, inu monga kholo mungamve bwanji kuti mwana wanu ayezedwe magazi kuzera njira zimezi?**

- **CG-** Atha kumva bwino kuti aziwe mmene nthupi mwa mwana mulili.
- **CG-** I would feel good knowing the status of my child

1. **Kwainu monga kholo la mwana wa chichepere, maganizo anu ndi otani pokhuzana ndi mayezedwe a magazi kuti tidziwe kuti mwana ali ndi HIV kapena ayi malingana ndi mmene tafotokozera za kayezedwe ka Cepheid kuti zosatira zimatuluka kwa minitsi 92?**

- **CG-**  Maganizo awo ndiabwino kuti aziziwa mmene mwana alili nthupi mwachangu.
- **CG-** it is a good idea knowing the child’s status

1. **Kodi njira zimenezi tingazikhazikise bwanji mu zipatala? (tatiwuzani, tiyambe ndi gulu liti la anthu ndipo nchifukwa chani mukuganiza kuti tiyambe ndi gulu limeneli chifukwa chain?**

- **CG-**  Ma nurse akuyenera kufotokozera zaubwino wanjirazi, komanso ana ndamene akuyenera kuyamba chifukwa mayezedwe awo pakali pano kilube.
- **CG-** Nurses need to explain the importance’s of this testing method and they also need to start because we do not have testing methods like this

1. **Kodi tingapange bwanji kuti kuyezesa magazi kwa ana ndi makolo awo kapena anthu owayang’ira zikhale za chinsinsi?**

- **CG-**  Kufunika kukhala pawiriwiri pakati pa dokotala ndi owezedwayo kuyi zikhale zachinsinsi.
- **CG-** It is supposed to be between the doctor and the one getting tested.

1. **Kodi makolo angatengepo gawo lanji kuti njira zoyezesera magazi za Cepheid zikhazikisidwe mu chipatala chathu chino cha Mulanje?**

- **CG-** Azitenga ana kukayezetsa chifukwa kumangomusunga mwana sungadziwe mmen alili nthupi.
- **CG-** They should be taking their children to get tested

b). **Kodi makolo awuzidwe zotani ndi uphungu wotani kuti amvesese za njira zoyezesera magazi za Cepheid?**

- **CG-** Kumwera malangizo ochokera kwa ma dotolo akamapeleka uphungu okhuidza zinjilazi.
- **CG-** Following the doctors advise

1. **Kodi azibambo angatengepo gawo lanji kuti njira zoyezesera magazi za Cepheid ndi zikhazikisidwe mu chipatala chathu chino cha Mulanje? Tingawalimbikise bwanji azibambo kuti azitenga nawo gawo mukuyezedwa magazi mu njira za Cepheid ndi ?**

- **CG-**  Azimayi akuyenera kuwalimbikitsa amuna awo kuti akayezetse ndikudziwa mmene alili nthupi.
- **CG-** Women need to encourage their husbands to get tested

1. **Kodi anthu a mmudzi mwanu angamve bwanji njira zoyezesera magazi za Cepheid zitakhazikisidwa pa chipatala chanu chaching’ono mmudzi mwanu. Tingatani kuti anthu a mmudzi muno alimbikisidwe kutenga nawo mbali mu njira zoyezetsera magazi za Cepheid?**

- **CG-** Atha kumva bwino chifukwa saziyenda ulendo wautali kukayezetsa, achipatala akuyenela kuuza amfumu kuti awuze anthu a m’mudzi mwawo zamayezedwe a Cepheid.
- **CG-** They would like it because they would not have to walk for a long distance now. You need to go through the village chief to reach out to a lot more people

1. **Kodi inu ndi anthu ena mma midzi mu mumakhala ndi nkhwa zanji zokhuzana ndi kulandila zosatira za magazi mwana akayezedwa kuti tiziwe kuti mwana ali ndi HIV kapena ayi?**

- **CG-**  Nkhawa imakhala yoti mwana wawo ayezedwe ndikuziwa zotsatira.
- **CG-** Stress comes about because of fear of knowing the results

1. **Kodi mungakhale ndi njira kapena maganizo a momwe tingathandizire kuchepesa nkhawa zokhuzana ndikulandila zotsatira za magazi mwana wayezedwa kuti tidziwe kuti mwana ali ndi HIV kapena ayi?**

- **CG-** Kuti nkhawa isakhalepo akuyenera kutenga mwana kukamuyezetsa ndikudziwa zotsatila.
- **CG-** To reduce fear, people just need to get their children tested so they can know their status and accept it.

1. **Kuchokera pa nthawi yomwe mwana wanu wayezedwa magazi kuti tidziwe kuti mwana ali ndi HIV kapena ayi, mungapilile nthawi yayitali bwanji kuti mudziwe zosatira**

- **Tsiku lomwelo**

**Patatha masiku**

**Miyezi iwiri kapena itatu**

**Fotokozani zifukwa zomwe mungasankhile yankho limeneli**

- **CG-**  Chifukwa akapda kumva tsiku lomwero akhala ndi nkhawa yayikulu.
- **CG-** I need to hear them on the same day to prevent stress

1. **Mwana wanu atayezedwa magazi, mungafune kudikila nthawi yayitali bwanji kuti mudziwe kuti mwana ali ndi HIV yomwe yimayambitsa matenda a AIDS?**

- **TSiku lomwelo**

**Patatha masiku**

**Miyezi iwiri kapena itatu**

**Fotokozani zifukwa zimene mwasankhila yankho limenelo**

- **CG-**

1. **Mwana wanu atayezedwa magazi mungafune kudikila nthaawi yayitali bwanji kuti muziwe kuti mwana alibe HIV yomwe imayambitsa matenda a AIDS**

**Tsiku lomwelo**

**Patatha masiku**

**Miyezi iwiri kapena itatu**

**Fotokozani zifukwa zomwe mungasankhile yankho limenelo**

- **CG-** Alibe yankho lililonse.
- **CG-** No thoughts on this

1. **kodi mungafune muwuzidwe zotani ndi uphungu otani kuti inu mupange chisankho choti mwana wanu ayezedwe magazi kuti mudziwe kuti mwana ali ndi HIV yomwe imayambitsa matenda a AIDS kapena ayi? Fotokozani bwino lomwe.**

- **CG-** Awuzidwe ubwino woyezetsa HIV yomwe imayambitsa EDZI.
- **CG-**They should be taught the importance of HIV testing

1. **Mungafune kuti tikufikileni mu njira yotani kuti tikuwuzeni zimezi ndikukupasani uphungu umenewu wa njira zoyezesera magazi za Cepheid?**

- **CG-**  Kungomvera malangizo akuchipatala.
- **CG-** listening to the hospital’s advice

1. **Kodi mungathe kuwalimbikisa makolo anzanu kapena owasamalira ana kuti alore ana Awo ayezedwwe magazi kuti aziwe ngati ali ndi HIV yoyambitsa matenda a AIDS kugwilitsa ntchito Cepheid?**

- **CG-**  Eya
- **CG-** yes

**15b) Nkhawa zanu zingakhale zotani ndi mayezedwe amenewa a Cepheid?**

- **CG-**  Alipobe nkhawa chifukwa akufuna mwana athandizidwe.
- **CG-** no worries because its her child getting help

1. **Kodi mungamve bwanji ngati munthu wina wa mmudzi mwanu ataziwa zotsatira za magazi a mwana wanu atayezedwa kufufuza ngati ali ndi HIV kapena ayi?**

- **CG-** Sangamve bwino chifukwa munthu azilalika za zotsatira zawo.
- **CG- I** would not be happy seeing someone preach about the results

1. **Kodi muli ndi maganizo kapena nkhawa zina zomwe mungafune kutidziwisa pa nkhani imeneyi**

- **CG-** Nkhawa alibe koma kwawo ndikusangalala ndi njilazi kuti ndizabwino.
- **CG-** only joy brought about because of this new development
