# Supplementary material for: ‘If I am on ART, my new-born baby should be put on treatment immediately’: Exploring the acceptability, and appropriateness of Cepheid Xpert HIV-1 Qual assay for early infant diagnosis of HIV in Malawi
Source: PLOS Glob Public Health. 2023 Mar 10;3(3):e0001135. doi: 10.1371/journal.pgph.0001135 (PMC10021387; doi:10.1371/journal.pgph.0001135)
Supplement: S2 File — (ZIP) [file pgph.0001135.s005.zip › transcripts responses chichewa& english/DET028.docx]

**DET028_CG_F_30.7.18**

**ALIBE GANIZO LILILONSE FROM QUE.4**

1. **Malingana ndi mmene tafotokozera za kayezedwe ka Cepheid, mwana ayenera kutengedwa magazi pachara kapena pa nsempha, inu monga kholo mungamve bwanji kuti mwana wanu ayezedwe magazi kuzera njira zimezi?**

- **CG-** Ndingamve bwino chifukwa choti ndidziwa m’mene alili mwachangu.
- **CG-** I would feel good because I would know how my child is quickly.

1. **Kwainu monga kholo la mwana wa chichepere, maganizo anu ndi otani pokhuzana ndi mayezedwe a magazi kuti tidziwe kuti mwana ali ndi HIV kapena ayi malingana ndi mmene tafotokozera za kayezedwe ka Cepheid kuti zosatira zimatuluka kwa minitsi 92?**

- **CG-**  Maganizo anga ndi abwino kwambiri chifukwa ndadziwa m’men mwana wanga alili.
- **CG-** I think it very good because I’ll now know the status of my child

1. **Kodi njira zimenezi tingazikhazikise bwanji mu zipatala? (tatiwuzani, tiyambe ndi gulu liti la anthu ndipo nchifukwa chani mukuganiza kuti tiyambe ndi gulu limeneli chifukwa chain?**

- **CG-** Apa ndilibe ganizo, Tiyambire ana chifukwa ana ndi atsogoleri a mawa.
- **CG-** no comment but we should start with children because they are the leaders of tomorrow

1. **Kodi tingapange bwanji kuti kuyezesa magazi kwa ana ndi makolo awo kapena anthu owayang’ira zikhale za chinsinsi?**

- **CG-** kungomuuza kholo lamwana opanda wina aliyense komaso adokotala akuyenera kusunga chinsinsi
- **CG-** Only give the results to the parents

1. **Kodi makolo angatengepo gawo lanji kuti njira zoyezesera magazi za Cepheid zikhazikisidwe mu chipatala chathu chino cha Mulanje?**

- **CG-** uthengawu kungowumvetsetsa kumanso kuwuza anzathu
- **CG-** understanding the message and telling our friends

b). **Kodi makolo awuzidwe zotani ndi uphungu wotani kuti amvesese za njira zoyezesera magazi za Cepheid?**

- **CG-** atiwuza njira zomwe tingagwilitse ntchito komanso atipase ndondomeko losamalira ana
- **CG-** We should be told how to take care of our child

1. **Kodi azibambo angatengepo gawo lanji kuti njira zoyezesera magazi za Cepheid zikhazikisidwe mu chipatala chathu chino cha Mulanje? Tingawalimbikise bwanji azibambo kuti azitenga nawo gawo mukuyezedwa magazi mu njira za Cepheid?**

- **CG-**  tikuyenera kuwafotokozera ndikuwawuza azipita kukayezetsa
- **CG-** we need to explain and tell them to get tested
- Pokayezetsa magazi tikuyenera kunyamukana ndi azibambo ndikukunyamulana ndi azibambo ndikuwawuza zakuophya kwamatendawa

CG- When we come with for the test we should bring husbands along and tell them the importance of the test

1. **Kodi anthu a mmudzi mwanu angamve bwanji njira zoyezesera magazi za Cepheid ndi zitakhazikisidwa pa chipatala chanu chaching’ono mmudzi mwanu. Tingatani kuti anthu a mmudzi muno alimbikisidwe kutenga nawo mbali mu njira zoyezetsera magazi za Cepheid?**

- **CG-** ndilibe ganizo lililonse
- **CG-** no comment on this

1. **Kodi inu ndi anthu ena mma midzi mu mumakhala ndi nkhwa zanji zokhuzana ndi kulandila zosatira za magazi mwana akayezedwa kuti tiziwe kuti mwana ali ndi HIV kapena ayi?**

- **CG-** ndimakhala ndi nkhawa chifukwa choti timakhara chifukwa choti timakhala tilibe ganizo lilonse chifukwa choti pamakhala sitinamve kanthu
- **CG-** I get worried when I have not gone for any testing

1. **Kodi mungakhale ndi njira kapena maganizo a momwe tingathandizire kuchepesa nkhawa zokhuzana ndikulandila zotsatira za magazi mwana wayezedwa kuti tidziwe kuti mwana ali ndi HIV kapena ayi?**

- **CG-** Aliyense amakhala ndikhawa kuthesa kwake ndikuziwa zotsatira basi
- **CG-** Everyone has stress but knowing the results is the only help.

1. **Kuchokera pa nthawi yomwe mwana wanu wayezedwa magazi kuti tidziwe kuti mwana ali ndi HIV kapena ayi, mungapilile nthawi yayitali bwanji kuti mudziwe zosatira**

**Tsiku lomwelo**

**Patatha masiku**

**Miyezi iwiri kapena itatu**

**Fotokozani zifukwa zomwe mungasankhile yankho limeneli**

- **CG-** Ngati wapezeka nako uyenera kuvomele za mwachangu ndikumuteteza
- **CG-** if found positive, quickly accept it so you can protect the child.

1. **Mwana wanu atayezedwa magazi, mungafune kudikila nthawi yayitali bwanji kuti mudziwe kuti mwana ali ndi HIV yomwe yimayambitsa matenda a AIDS?**

**TSiku lomwelo**

**Patatha masiku**

**Miyezi iwiri kapena itatu**

**Fotokozani zifukwa zimene mwasankhila yankho limenelo**

- **CG-** Ndi Tsiku limene ndikuyembekezera zotsatira ndikuti ndiziwe kuti thupi muli bwanji
- **CG-** I want to get results same day

1. **Mwana wanu atayezedwa magazi mungafune kudikila nthaawi yayitali bwanji kuti muziwe kuti mwana alibe HIV yomwe imayambitsa matenda a AIDS**

**Tsiku lomwelo**

**Patatha masiku**

**Miyezi iwiri kapena itatu**

**Fotokozani zifukwa zomwe mungasankhile yankho limenelo**

**CG: -** Ndifuna ndiziwe ngati alinako kapena ayi ndikuteteza

**CG-** Because I need to start thinking of what to do next

1. **kodi mungafune muwuzidwe zotani ndi uphungu otani kuti inu mupange chisankho choti mwana wanu ayezedwe magazi kuti mudziwe kuti mwana ali ndi HIV yomwe imayambitsa matenda a AIDS kapena ayi? Fotokozani bwino lomwe.**

- **CG-** Kuwuzidwa zakuwophya kwa matenda amenewa
- **CG-** being told the dangers of the virus

1. **Mungafune kuti tikufikileni mu njira yotani kuti tikuwuzeni zimezi ndikukupasani uphungu umenewu wa njira zoyezesera magazi za Cepheid?**

- **CG-** Kaya kuzera kusikero kaya kutiyendale mukhonza kutifikira
- **CG-** Antenatal Clinic or at our home

1. **Kodi mungathe kuwalimbikisa makolo anzanu kapena owasamalira ana kuti alore ana Awo ayezedwwe magazi kuti aziwe ngati ali ndi HIV yoyambitsa matenda a AIDS kugwilitsa ntchito Cepheid?**

- **CG-** Ayi
- **CG-** yes

**15b) Nkhawa zanu zingakhale zotani ndi mayezedwe amenewa a Cepheid?**

- **CG-** Pakuphonyesa kumuyeza chifukwa amataya magazi ambiri zimenezi zingapase nkhawa
- **CG-** Losing blood in the process of blood draw

1. **Kodi mungamve bwanji ngati munthu wina wa mmudzi mwanu ataziwa zotsatira za magazi a mwana wanu atayezedwa kufufuza ngati ali ndi HIV kapena ayi?**

- **CG-** Zingakhale zowawa chifukwa choti aliyense amafuna chinsinsi
- **CG-** it would be painful because everyone wants to keep their secrets

1. **Kodi muli ndi maganizo kapena nkhawa zina zomwe mungafune kutidziwisa pa nkhani imeneyi**

- **CG-** Ndilibe nkhawa ili yonse pankhani imeneyi
- **CG-** no objections on this
