# Supplementary material for: ‘If I am on ART, my new-born baby should be put on treatment immediately’: Exploring the acceptability, and appropriateness of Cepheid Xpert HIV-1 Qual assay for early infant diagnosis of HIV in Malawi
Source: PLOS Glob Public Health. 2023 Mar 10;3(3):e0001135. doi: 10.1371/journal.pgph.0001135 (PMC10021387; doi:10.1371/journal.pgph.0001135)
Supplement: S2 File — (ZIP) [file pgph.0001135.s005.zip › transcripts responses chichewa& english/DET030.docx]

**DET030_CG_F_30.7.18**

1. **Malingana ndi mmene tafotokozera za kayezedwe ka Cepheid, mwana ayenera kutengedwa magazi pachara kapena pa nsempha, inu monga kholo mungamve bwanji kuti mwana wanu ayezedwe magazi kuzera njira zimezi?**

- **CG-** Sindingamve bwino ndi njira yapansempha komabe sindikufuna kuti mwana athandizidwe ndiye ndikungoyenera kuvomeleza
- **CG-** I would not be confortable with venous blood draw but I would accept it because I want my child to be helped.

1. **Kwainu monga kholo la mwana wa chichepere, maganizo anu ndi otani pokhuzana ndi mayezedwe a magazi kuti tidziwe kuti mwana ali ndi HIV kapena ayi malingana ndi mmene tafotokozera za kayezedwe ka Cepheid kuti zosatira zimatuluka kwa minitsi 92?**

- **CG-**  NDiyabwino chifukwa choti zinthu ziziyenda mwansanga sichifukwa choti nthawi yakale kunalibe njirazi
- **CG-** I like this method because it is faster than the past methods

1. **Kodi njira zimenezi tingazikhazikise bwanji mu zipatala? (tatiwuzani, tiyambe ndi gulu liti la anthu ndipo nchifukwa chani mukuganiza kuti tiyambe ndi gulu limeneli chifukwa chain?**

- **CG-** Tizikhazikitse kuti anthu azilandira zinthu mwansanga
- Tiyambire ana chifukwa choti mwana akhonza kumanyetchera ife osaziwa kuti akudwala
- It needs to be implemented firstly to children because sometimes they get malnourished and we do not know what they are suffering from

1. **Kodi tingapange bwanji kuti kuyezesa magazi kwa ana ndi makolo awo kapena anthu owayang’ira zikhale za chinsinsi?**

- **CG-**  Munthu ukamafuna kumuyeza muzimutengera kuchipinda
- **CG-** The process needs to be done in a private room

1. **Kodi makolo angatengepo gawo lanji kuti njira zoyezesera magazi za Cepheid zikhazikisidwe mu chipatala chathu chino cha Mulanje?**

- **CG-** Kuwalimbikitsa anthu obwelesa chithandizo kuti chibwere nsanga.
- **CG-** Encouraging the sponsors that they should help out in setting up this methods

b). **Kodi makolo awuzidwe zotani ndi uphungu wotani kuti amvesese za njira zoyezesera magazi za Cepheid?**

- **CG-** Akuyenera kutilangiza njira zotsatira njirazi.
- **CG**- They need to be advised on the whole process on the test

1. **Kodi azibambo angatengepo gawo lanji kuti njira zoyezesera magazi za Cepheid zikhazikisidwe mu chipatala chathu chino cha Mulanje? Tingawalimbikise bwanji azibambo kuti azitenga nawo gawo mukuyezedwa magazi mu njira za Cepheid?**

- **CG-**  Akamva azibwera kuzayezetsa mwachangu ndikuziwa mmene nthupi mwawo mulili
- **CG-** They should be encouraged to be coming for testing in early stages

1. **Kodi anthu a mmudzi mwanu angamve bwanji njira zoyezesera magazi za Cepheid zitakhazikisidwa pa chipatala chanu chaching’ono mmudzi mwanu. Tingatani kuti anthu a mmudzi muno alimbikisidwe kutenga nawo mbali mu njira zoyezetsera magazi za Cepheid?**

- **CG-** Amva bwino chifukwa chipatala chili pafupi ndisayenda nthawi yayitali kuti akaziwe zanthupi mwake kuwawunikira ubwino woyezetsa
- **CG-** They would like it because the hospital is really close and they would not need to walk for a long distance to get tested

1. **Kodi inu ndi anthu ena mma midzi mu mumakhala ndi nkhwa zanji zokhuzana ndi kulandila zosatira za magazi mwana akayezedwa kuti tiziwe kuti mwana ali ndi HIV kapena ayi?**

- **CG-**  Ine nkhawa sindingakhale nayo chifukwa zinthuzi ndizothandiza ife tomwe
- **CG-** I do not have any worry because I know my child will get helped regardless of the results

1. **Kodi mungakhale ndi njira kapena maganizo a momwe tingathandizire kuchepesa nkhawa zokhuzana ndikulandila zotsatira za magazi mwana wayezedwa kuti tidziwe kuti mwana ali ndi HIV kapena ayi?**

- **CG-** Kumakhara mmagulu kumakambilana za mulili wavutawa kuti tigonjese ndikuchita masewero kuti anthu omwe apezeka nako asamadandaule
- **CG-** joining different groupings and sharing HIV related issues and performing plays/dramas so as to entertain and motivate the infected

1. **Kuchokera pa nthawi yomwe mwana wanu wayezedwa magazi kuti tidziwe kuti mwana ali ndi HIV kapena ayi, mungapilile nthawi yayitali bwanji kuti mudziwe zosatira**

**Tsiku lomwelo**

**Patatha masiku**

- **2-3 months**

**Fotokozani zifukwa zomwe mungasankhile yankho limeneli**

- **CG-**  Chifukwa cholti zotsatira zikatuluka pompo nkhawa sukhala nayo.
- **CG-** Because when you get your results immediately you do not become worried

1. **Mwana wanu atayezedwa magazi, mungafune kudikila nthawi yayitali bwanji kuti mudziwe kuti mwana ali ndi HIV yomwe yimayambitsa matenda a AIDS?**

- **Same day**

**Patatha masiku**

**Miyezi iwiri kapena itatu**

**Fotokozani zifukwa zimene mwasankhila yankho limenelo**

- **CG-**Ukuyenera kuziwa tsiku lomwelo kuti ulandile thandizo ngati pakuyeneleka kutero.
- **CG-** Same day because my child need to get help at the same time too

1. **Mwana wanu atayezedwa magazi mungafune kudikila nthaawi yayitali bwanji kuti muziwe kuti mwana alibe HIV yomwe imayambitsa matenda a AIDS**

- **Same day**

**Patatha masiku**

**Miyezi iwiri kapena itatu**

**Fotokozani zifukwa zomwe mungasankhile yankho limenelo**

- **CG-** Ndasankha tsiku lomwero chifukwa choti mtima umagunda ukakhala sunamve zotsatira
- **CG-**  Because when you have not heard the results your heart is not at ease

1. **kodi mungafune muwuzidwe zotani ndi uphungu otani kuti inu mupange chisankho choti mwana wanu ayezedwe magazi kuti mudziwe kuti mwana ali ndi HIV yomwe imayambitsa matenda a AIDS kapena ayi? Fotokozani bwino lomwe.**

- **CG-**Kuwonana ndi adokotala kuti akalangize njira zabwino zotsatira
- **CG-** Meeting the doctor that he/she should give proper advise

1. **Mungafune kuti tikufikileni mu njira yotani kuti tikuwuzeni zimezi ndikukupasani uphungu umenewu wa njira zoyezesera magazi za Cepheid?**

- **CG-**  Popangisa nsonkhano ndikutiwuza kuzera mu wailesi
- **CG-** Organising meetings and through the radio

1. **Kodi mungathe kuwalimbikisa makolo anzanu kapena owasamalira ana kuti alore ana Awo ayezedwwe magazi kuti aziwe ngati ali ndi HIV yoyambitsa matenda a AIDS kugwilitsa ntchito Cepheid?**

- **CG-**  Eya
- **CG-** Yes

**15b) Nkhawa zanu zingakhale zotani ndi mayezedwe amenewa a Cepheid?**

- **CG-** Alibe nkhawa ina iliyonse.
- **CG-** I have no problem with it

1. **Kodi mungamve bwanji ngati munthu wina wa mmudzi mwanu ataziwa zotsatira za magazi a mwana wanu atayezedwa kufufuza ngati ali ndi HIV kapena ayi?**

- **CG-** Ndingadandaule chifukwa munthu umakhala ndi chitonzo
- **CG-** I can like that because of fear of discrimination

1. **Kodi muli ndi maganizo kapena nkhawa zina zomwe mungafune kutidziwisa pa nkhani imeneyi**

- **CG-**  Ndilibe nkhawa kwanga ndikungonyadira kuti apitilize
- **CG-** I have no problem with it, I am just glad and wish that this method continues
