# Supplementary material for: ‘If I am on ART, my new-born baby should be put on treatment immediately’: Exploring the acceptability, and appropriateness of Cepheid Xpert HIV-1 Qual assay for early infant diagnosis of HIV in Malawi
Source: PLOS Glob Public Health. 2023 Mar 10;3(3):e0001135. doi: 10.1371/journal.pgph.0001135 (PMC10021387; doi:10.1371/journal.pgph.0001135)
Supplement: S2 File — (ZIP) [file pgph.0001135.s005.zip › transcripts responses chichewa& english/DET031.docx]

**DET031_CG_F_30.7.18**

1. **Malingana ndi mmene tafotokozera za kayezedwe ka Cepheid, mwana ayenera kutengedwa magazi pachara kapena pa nsempha, inu monga kholo mungamve bwanji kuti mwana wanu ayezedwe magazi kuzera njira zimezi?**

- **CG-** Ine ndingamve bwino chifukwa choti akayezedwa ndidziwa mmene alili.
- **CG-** I would feel good because I will know how the child is.

1. **Kwainu monga kholo la mwana wa chichepere, maganizo anu ndi otani pokhuzana ndi mayezedwe a magazi kuti tidziwe kuti mwana ali ndi HIV kapena ayi malingana ndi mmene tafotokozera za kayezedwe ka Cepheid kuti zosatira zimatuluka kwa minitsi 92?**

- **CG-**  Maganizo awo ndiabwino kuti aziziwa mene mwana alili nthupi mwachangu
- **CG-** It is a good idea to know the child’s status within a short time.

1. **Kodi njira zimenezi tingazikhazikise bwanji mu zipatala? (tatiwuzani, tiyambe ndi gulu liti la anthu ndipo nchifukwa chani mukuganiza kuti tiyambe ndi gulu limeneli chifukwa chain?**

- **CG-** Ma nurse akuyenela kufotokozela azimayi pamene abwela ndi ana kuchipatala zaubwino wanjirazi komaso ana ndamene akuyenera kuyamba chifukwa mayezedwe awo pakali pano kulibe
- **CG-** The nurses needs to explain to the women when they come to the hospital. It needs to start with the children

1. **Kodi tingapange bwanji kuti kuyezesa magazi kwa ana ndi makolo awo kapena anthu owayang’ira zikhale za chinsinsi?**

- **CG-** Kufunika kukhala pawiriwiri pakati pa doctor ndi woyezetsayo kuti zikhale zachinsinsi.
- **CG-** It should only be between the doctor and parents

1. **Kodi makolo angatengepo gawo lanji kuti njira zoyezesera magazi za Cepheid zikhazikisidwe mu chipatala chathu chino cha Mulanje?**

- **CG-**  Azitenga ana kukayezetsa chifukwa kumangomusunga mwana sungadziwe mene alili nthupi
- **CG-** They should be taking their children for testing without which they would know nothing.

b). **Kodi makolo awuzidwe zotani ndi uphungu wotani kuti amvesese za njira zoyezesera magazi za Cepheid ?**

- **CG-** Kumvela malangizo ochokela kwa ma dotolo akamapeleka uphungu
- **CG-** listening to the doctor during Guidance and counselling

1. **Kodi azibambo angatengepo gawo lanji kuti njira zoyezesera magazi za Cepheid zikhazikisidwe mu chipatala chathu chino cha Mulanje? Tingawalimbikise bwanji azibambo kuti azitenga nawo gawo mukuyezedwa magazi mu njira za Cepheid?**

- **CG-**  Azimayi akuyenela kuwalimbikitsa amuna awo kuti akayezetse ndikuziwa mene alili nthupi
- **CG-** Women need to encourage their husbands to get tested

1. **Kodi anthu a mmudzi mwanu angamve bwanji njira zoyezesera magazi za Cepheid zitakhazikisidwa pa chipatala chanu chaching’ono mmudzi mwanu. Tingatani kuti anthu a mmudzi muno alimbikisidwe kutenga nawo mbali mu njira zoyezetsera magazi za Cepheid?**

- **CG-** Atha kumva bwino chifukwa saziyenda ulendo wautali kukayezetsa achipatala akuyenela kuuza amfumu kuti auze anthu ammudzi mwawo zamayezedwe a Cepheid
- **CG-** They can like it because of the short distance. They need to go through the village chief

1. **Kodi inu ndi anthu ena mma midzi mu mumakhala ndi nkhwa zanji zokhuzana ndi kulandila zosatira za magazi mwana akayezedwa kuti tiziwe kuti mwana ali ndi HIV kapena ayi?**

- **CG-** Nkhawa imakhala yoti mwana wawo ayezedwe ndikuziwa zotsatila
- **CG-** I am worried of the results of my child

1. **Kodi mungakhale ndi njira kapena maganizo a momwe tingathandizire kuchepesa nkhawa zokhuzana ndikulandila zotsatira za magazi mwana wayezedwa kuti tidziwe kuti mwana ali ndi HIV kapena ayi?**

- **CG-**  Kuti nkhawa isakhalepo akuyenela kutenga mwana kukamuyezetsa ndikuziwa zotsatila
- **CG-** We need to get children tested so that we should not be in doubts

1. **Kuchokera pa nthawi yomwe mwana wanu wayezedwa magazi kuti tidziwe kuti mwana ali ndi HIV kapena ayi, mungapilile nthawi yayitali bwanji kuti mudziwe zosatira**

- **Tsiku lomwelo**

**Patatha masiku**

**Miyezi iwiri kapena itatu**

**Fotokozani zifukwa zomwe mungasankhile yankho limeneli**

- **CG-** Chifukwa akapanda kumva tsiku lomwelo akhala ndi nkhawa yaikulu
- **CG-** Because if Ithey do not hear the results the same day Iwill be very stressed

1. **Mwana wanu atayezedwa magazi, mungafune kudikila nthawi yayitali bwanji kuti mudziwe kuti mwana ali ndi HIV yomwe yimayambitsa matenda a AIDS?**

- **TSiku lomwelo**

**Patatha masiku**

**Miyezi iwiri kapena itatu**

**Fotokozani zifukwa zimene mwasankhila yankho limenelo**

- **CG-** Alibe ganizo.
- **CG-** no comment

1. **Mwana wanu atayezedwa magazi mungafune kudikila nthaawi yayitali bwanji kuti muziwe kuti mwana alibe HIV yomwe imayambitsa matenda a AIDS**

- **Tsiku lomwelo**

**Patatha masiku**

**Miyezi iwiri kapena itatu**

**Fotokozani zifukwa zomwe mungasankhile yankho limenelo**

- **CG-** Alibe ganizo.
- **CG-** no comment

1. **kodi mungafune muwuzidwe zotani ndi uphungu otani kuti inu mupange chisankho choti mwana wanu ayezedwe magazi kuti mudziwe kuti mwana ali ndi HIV yomwe imayambitsa matenda a AIDS kapena ayi? Fotokozani bwino lomwe.**

- **CG-** Auzidwe ubwino woyezetsa HIV yomwe imayambitsa AIDS.
- **CG-** importance of HIV and AIDs testing

1. **Mungafune kuti tikufikileni mu njira yotani kuti tikuwuzeni zimezi ndikukupasani uphungu umenewu wa njira zoyezesera magazi za Cepheid?**

- **CG-** Kangomvela malangizo akuchipatala.
- **CG-** following the counselling

1. **Kodi mungathe kuwalimbikisa makolo anzanu kapena owasamalira ana kuti alore ana Awo ayezedwwe magazi kuti aziwe ngati ali ndi HIV yoyambitsa matenda a AIDS kugwilitsa ntchito Cepheid?**

- **CG-**  Eya
- **CG-** yes

**15b) Nkhawa zanu zingakhale zotani ndi mayezedwe amenewa a Cepheid?**

- **CG-**  Ine ndilibe nkhawa chifukwa choti ndimafuna ndidziwe mmene mwana alili.
- **CG-** I have no stress because I want to know how the child is

1. **Kodi mungamve bwanji ngati munthu wina wa mmudzi mwanu ataziwa zotsatira za magazi a mwana wanu atayezedwa kufufuza ngati ali ndi HIV kapena ayi?**

- **CG-** Sangamve bwino chifukwa munthuyo azilalika za ma results awo
- **CG-** wouldn’t feel good hearing someone preach the results

1. **Kodi muli ndi maganizo kapena nkhawa zina zomwe mungafune kutidziwisa pa nkhani imeneyi**

- **CG-** NKhawa alibe koma kwawo ndikusangalala ndi njilazi kuti ndizabwino
- **CG-** no problem but just joy from the new method
