# Supplementary material for: ‘If I am on ART, my new-born baby should be put on treatment immediately’: Exploring the acceptability, and appropriateness of Cepheid Xpert HIV-1 Qual assay for early infant diagnosis of HIV in Malawi
Source: PLOS Glob Public Health. 2023 Mar 10;3(3):e0001135. doi: 10.1371/journal.pgph.0001135 (PMC10021387; doi:10.1371/journal.pgph.0001135)
Supplement: S2 File — (ZIP) [file pgph.0001135.s005.zip › transcripts responses chichewa& english/DET033.docx]

**DET033_CG_F_30.7.18**

1. **Malingana ndi mmene tafotokozera za kayezedwe ka Cepheid, mwana ayenera kutengedwa magazi pachara kapena pa nsempha, inu monga kholo mungamve bwanji kuti mwana wanu ayezedwe magazi kuzera njira zimezi?**

- **CG-** Ndingamve bwino chifukwa choti ngati angapezeke nako akhonza kuthandizidwa mwachangu
- **CG-** I would feel okay with it because if found positive my child will be helped.

1. **Kwainu monga kholo la mwana wa chichepere, maganizo anu ndi otani pokhuzana ndi mayezedwe a magazi kuti tidziwe kuti mwana ali ndi HIV kapena ayi malingana ndi mmene tafotokozera za kayezedwe ka Cepheid kuti zosatira zimatuluka kwa minitsi 92?**

- **CG-**  Ndili osangalala Kamba koti njira zimenezi zikufuna zikhazikitsidwe chifukwa choti zitithandiza tonse.
- **CG-** I am happy because this new method will help us all

1. **Kodi njira zimenezi tingazikhazikise bwanji mu zipatala? (tatiwuzani, tiyambe ndi gulu liti la anthu ndipo nchifukwa chani mukuganiza kuti tiyambe ndi gulu limeneli chifukwa chain?**

- **CG-**  Kukhazikitsa kwake ndikosafuna kwa munthu achimvetsa chifukwa choti njira zimenezi ndizothandiza ndipo mukuyenera kutifikira kusikelo kapena kupangiaa msonkhano muyambirer onse chifukwa zinthu zimayambira kwa kholo
- **CG-** You need to reach out to us through the antenatal clinic or conventions and it needs to start with adults so that we can know if it is okay for the children

1. **Kodi tingapange bwanji kuti kuyezesa magazi kwa ana ndi makolo awo kapena anthu owayang’ira zikhale za chinsinsi?**

- **CG-**  Chinsinsi ndichofunikira ndipo tikuyenera kuyamba kusunga tokha chinsinsi ndi madotolo
- **CG-** Parents and doctors are responsible in keeping privacy of the child

1. **Kodi makolo angatengepo gawo lanji kuti njira zoyezesera magazi za Cepheidzikhazikisidwe mu chipatala chathu chino cha Mulanje?**

- **CG-** Pamenepo ndilibe ganizo
- **CG-** I have no idea

b). **Kodi makolo awuzidwe zotani ndi uphungu wotani kuti amvesese za njira zoyezesera magazi za Cepheid?**

- **CG-** Mukuyenera kutiwuza kuyipa ndi ubwino wako ndipo mukunera kutiyankhura mwa chiondi ngati mmene mwatifikilamu
- **CG-** Tell us the importance and the disadvantages of this method and talk to us with love and respect

1. **Kodi azibambo angatengepo gawo lanji kuti njira zoyezesera magazi za Cepheid zikhazikisidwe mu chipatala chathu chino cha Mulanje? Tingawalimbikise bwanji azibambo kuti azitenga nawo gawo mukuyezedwa magazi mu njira za Cepheid?**

- **CG-**  Azibambo akungoyenera kuzayetsetsa chifukwa iwowonso ndiokhuzidwa ine ndiwafotokozere ubwino wakayetsa ndikuti akuyenera kutero kuti moyo wawo ukhale wanthanzi
- **CG-** Men need to take part by also going for the test

1. **Kodi anthu a mmudzi mwanu angamve bwanji njira zoyezesera magazi za Cepheid zitakhazikisidwa pa chipatala chanu chaching’ono mmudzi mwanu. Tingatani kuti anthu a mmudzi muno alimbikisidwe kutenga nawo mbali mu njira zoyezetsera magazi za Cepheid?**

- **CG-** Anthu ammudzi mwathu akhonza kumva bwino chifukwa choti njirazi zizakhala zili pafupi ndipo zithandiza ife tomwe
- **CG-** They would be happy that such an important thing is very close to them

1. **Kodi inu ndi anthu ena mma midzi mu mumakhala ndi nkhwa zanji zokhuzana ndi kulandila zosatira za magazi mwana akayezedwa kuti tiziwe kuti mwana ali ndi HIV kapena ayi?**

- **CG-**  Njirazi zithandiza ife tomwe komabe ndimakhala ndi nkhawa chifukwa choti mwana atati apezeke ndi matenda ndichinthu chovuta chifukwa choti mwana sayankhula
- **CG-** This method is really important but I am only worried that my child could be found positive

1. **Kodi mungakhale ndi njira kapena maganizo a momwe tingathandizire kuchepesa nkhawa zokhuzana ndikulandila zotsatira za magazi mwana wayezedwa kuti tidziwe kuti mwana ali ndi HIV kapena ayi?**

- **CG-**

1. **Kuchokera pa nthawi yomwe mwana wanu wayezedwa magazi kuti tidziwe kuti mwana ali ndi HIV kapena ayi, mungapilile nthawi yayitali bwanji kuti mudziwe zosatira**

- **Tsiku lomwelo**

**Patatha masiku**

**Miyezi iwiri kapena itatu**

**Fotokozani zifukwa zomwe mungasankhile yankho limeneli**

- **CG-** Chifukwa choti ndizafuna kuti ndiziwe mmene alili ndikuziwa mmene ndi ngamusamalile
- **CG-** I should know how my child is and how I can care for her

1. **Mwana wanu atayezedwa magazi, mungafune kudikila nthawi yayitali bwanji kuti mudziwe kuti mwana ali ndi HIV yomwe yimayambitsa matenda a AIDS?**

- **TSiku lomwelo**

**Patatha masiku**

**Miyezi iwiri kapena itatu**

**Fotokozani zifukwa zimene mwasankhila yankho limenelo**

- **CG-** Chifukwa choti sinanga zinthuzi zipita ku laboratory ndiye zikukhonza kutengako nthawi yochepa
- **CG-** Same day because I believe when a test is performed in the laboratory, it does not take too long

1. **Mwana wanu atayezedwa magazi mungafune kudikila nthaawi yayitali bwanji kuti muziwe kuti mwana alibe HIV yomwe imayambitsa matenda a AIDS**

- **Tsiku lomwelo**

**Patatha masiku**

**Miyezi iwiri kapena itatu**

**Fotokozani zifukwa zomwe mungasankhile yankho limenelo**

- **CG-**  Chifukwa choti ndifuna ndiziwe mmene mwana alili
- **CG –** Because I want to know my child’s status

1. **kodi mungafune muwuzidwe zotani ndi uphungu otani kuti inu mupange chisankho choti mwana wanu ayezedwe magazi kuti mudziwe kuti mwana ali ndi HIV yomwe imayambitsa matenda a AIDS kapena ayi? Fotokozani bwino lomwe.**

- **CG-** Akuyenera kutilimbikitsa pamayezetsedwe anenawa kuti tiziwe mmene alili ndikutilangiza njira zake
- **CG-** They need to encourage us to get tested and explain the whole procedure

1. **Mungafune kuti tikufikileni mu njira yotani kuti tikuwuzeni zimezi ndikukupasani uphungu umenewu wa njira zoyezesera magazi za Cepheid?**

- **CG-**  Malingana ndikayendedwe ndikhonza kusankha kuti mutifikile kuchipatala konkuno patifikira kuana.
- **CG-** Reach out to us when we come here the hospital

1. **Kodi mungathe kuwalimbikisa makolo anzanu kapena owasamalira ana kuti alore ana Awo ayezedwwe magazi kuti aziwe ngati ali ndi HIV yoyambitsa matenda a AIDS kugwilitsa ntchito Cepheid?**

- **CG-**  Eya
- **CG-**yes

**15b) Nkhawa zanu zingakhale zotani ndi mayezedwe amenewa a ndi Cepheid?**

- **CG-** Mkhawa yanga inali pomutenga magazi chifukwa njirayi ndiyowawa kwa mwana.
- **CG-** My worry arises on the process of drawing blood from the child

1. **Kodi mungamve bwanji ngati munthu wina wa mmudzi mwanu ataziwa zotsatira za magazi a mwana wanu atayezedwa kufufuza ngati ali ndi HIV kapena ayi?**

- **CG-** Ndilibe ganizo lili lonse
- **CG-** no comment

1. **Kodi muli ndi maganizo kapena nkhawa zina zomwe mungafune kutidziwisa pa nkhani imeneyi**

- **CG-** Alibe nkhawa kapena ganizo lililonse.
- **CG-** no concerns
