# Supplementary material for: ‘If I am on ART, my new-born baby should be put on treatment immediately’: Exploring the acceptability, and appropriateness of Cepheid Xpert HIV-1 Qual assay for early infant diagnosis of HIV in Malawi
Source: PLOS Glob Public Health. 2023 Mar 10;3(3):e0001135. doi: 10.1371/journal.pgph.0001135 (PMC10021387; doi:10.1371/journal.pgph.0001135)
Supplement: S2 File — (ZIP) [file pgph.0001135.s005.zip › transcripts responses chichewa& english/DET034.docx]

**DET034_CG_F_30.7.18**

1. **Malingana ndi mmene tafotokozera za kayezedwe ka Cepheid, mwana ayenera kutengedwa magazi pachara kapena pa nsempha, inu monga kholo mungamve bwanji kuti mwana wanu ayezedwe magazi kuzera njira zimezi?**

- **CG-** Atha kumva bwino kuti aziwe mene nthupi mwa mwana mulili chifukwa atha kumangosunga mwana asali bwino nthupi.
- **CG-** I would feel good knowing the HIV status of my child

1. **Kwainu monga kholo la mwana wa chichepere, maganizo anu ndi otani pokhuzana ndi mayezedwe a magazi kuti tidziwe kuti mwana ali ndi HIV kapena ayi malingana ndi mmene tafotokozera za kayezedwe ka Cepheid kuti zosatira zimatuluka kwa minitsi 30 ndi njira ya ndipo 92 ya Cepheid?**

- **CG-**  NJilazi ndizabwino chifukwa akufuna kuziwa mene nthupi mulili
- **CG-** it’s a good method since all they want to know is their status

1. **Kodi njira zimenezi tingazikhazikise bwanji mu zipatala? (tatiwuzani, tiyambe ndi gulu liti la anthu ndipo nchifukwa chani mukuganiza kuti tiyambe ndi gulu limeneli chifukwa chain?**

- **CG-** Kupita ma mdela osiyana siyana ndikuwauza, komaso tikuyenela kuyamba ndi ana chifukwa ana ndiachichepele sangathe kukayezetsa okha
- **CG-** Going in different areas and telling people. Start with children.

1. **Kodi tingapange bwanji kuti kuyezesa magazi kwa ana ndi makolo awo kapena anthu owayang’ira zikhale za chinsinsi?**

- **CG-** Zimatengela kwa kholo kuwauzauza anthu mene mwana alili kapena kungosungila wekha mumtima
- **CG-** it depends on the parent telling people or not about the child’s status

1. **Kodi makolo angatengepo gawo lanji kuti njira zoyezesera magazi za Cepheid zikhazikisidwe mu chipatala chathu chino cha Mulanje?**

- **CG-** kuti kholo lomwe lamva za ceipheid akauzeso azawo kuti apite kuchipatala kukayezetsa
- **CG-** The parents that have heard about Ceiphed should also tell their friends

b). **Kodi makolo awuzidwe zotani ndi uphungu wotani kuti amvesese za njira zoyezesera magazi za Cepheid?**

- **CG-** Akuyenela kufotokozeledwa mene njilazi zikuyendela makamaka zaubwino kapena kuipa kwa njilazi
- **CG-** They need to be explain the whole procedure and the advantages and disadvantages of this method

1. **Kodi azibambo angatengepo gawo lanji kuti njira zoyezesera magazi za Cepheid zikhazikisidwe mu chipatala chathu chino cha Mulanje? Tingawalimbikise bwanji azibambo kuti azitenga nawo gawo mukuyezedwa magazi mu njira za Cepheid?**

- **CG-**  Azibambo auze akazi awo kuti akayezetse komaso ayendeledwe kuti tikawalimbikitse za njila ya Cepheid.
- **CG-** Men should encourage their wives to get tested

1. **Kodi anthu a mmudzi mwanu angamve bwanji njira zoyezesera magazi za Cepheid zitakhazikisidwa pa chipatala chanu chaching’ono mmudzi mwanu. Tingatani kuti anthu a mmudzi muno alimbikisidwe kutenga nawo mbali mu njira zoyezetsera magazi za Cepheid?**

- **CG-** Atha kumva bwino kuti zawafupikila komaso kuwapitila mudzi ndikupangisa msonkhano.
- **CG-** They can like that this test is very close to them

1. **Kodi inu ndi anthu ena mma midzi mu mumakhala ndi nkhwa zanji zokhuzana ndi kulandila zosatira za magazi mwana akayezedwa kuti tiziwe kuti mwana ali ndi HIV kapena ayi?**

- **CG-** Nkhawa imakhalapo kuti chifukwa sudziwa mmene mwana alili nthupi.
- **CG-** I would get worried because we are not sure of the health status of the child

1. **Kodi mungakhale ndi njira kapena maganizo a momwe tingathandizire kuchepesa nkhawa zokhuzana ndikulandila zotsatira za magazi mwana wayezedwa kuti tidziwe kuti mwana ali ndi HIV kapena ayi?**

- **CG-** Alangizidwe kuti asakhale ndi nkhawa pamene zotsatila zisanatuluke
- **CG-** We should get proper counselling before the results

1. **Kuchokera pa nthawi yomwe mwana wanu wayezedwa magazi kuti tidziwe kuti mwana ali ndi HIV kapena ayi, mungapilile nthawi yayitali bwanji kuti mudziwe zosatira**

**Tsiku lomwelo**

**Patatha masiku**

- **Miyezi iwiri kapena itatu**

**Fotokozani zifukwa zomwe mungasankhile yankho limeneli**

- **CG-**  Alibe ganizo lomwe asakhila miyezi iwiri.
- **CG-** no reason for choosing 2 months

1. **Mwana wanu atayezedwa magazi, mungafune kudikila nthawi yayitali bwanji kuti mudziwe kuti mwana ali ndi HIV yomwe yimayambitsa matenda a AIDS?**

**TSiku lomwelo**

**Patatha masiku**

- **Miyezi iwiri kapena itatu**

**Fotokozani zifukwa zimene mwasankhila yankho limenelo**

- **CG-** Alibeso ganizo lomwe asakhila patatha miyezi iwiri
- **CG-** no reason for choosing 3months

1. **Mwana wanu atayezedwa magazi mungafune kudikila nthaawi yayitali bwanji kuti muziwe kuti mwana alibe HIV yomwe imayambitsa matenda a AIDS**

**Tsiku lomwelo**

**Patatha masiku**

**Miyezi iwiri kapena itatu**

**Fotokozani zifukwa zomwe mungasankhile yankho limenelo**

- **CG-** Alibe yankho linalililonse
- **CG-** no answer

1. **kodi mungafune muwuzidwe zotani ndi uphungu otani kuti inu mupange chisankho choti mwana wanu ayezedwe magazi kuti mudziwe kuti mwana ali ndi HIV yomwe imayambitsa matenda a AIDS kapena ayi? Fotokozani bwino lomwe.**

- **CG-** Achipatala afotokozele anthu zaubwino wakuyezetsa
- **CG-** The hospital personel should explain the importance of this test

1. **Mungafune kuti tikufikileni mu njira yotani kuti tikuwuzeni zimezi ndikukupasani uphungu umenewu wa njira zoyezesera magazi za Cepheid?**

- **CG-**  NJila iliyonse yomwe achipatala angapange
- **CG-** In any way the hospital is comfortable with

1. **Kodi mungathe kuwalimbikisa makolo anzanu kapena owasamalira ana kuti alore ana Awo ayezedwwe magazi kuti aziwe ngati ali ndi HIV yoyambitsa matenda a AIDS kugwilitsa ntchito Cepheid?**

- **CG-**  Eya
- **CG-** yes

**15b) Nkhawa zanu zingakhale zotani ndi mayezedwe amenewa a Cepheid?**

- **CG-**  Nkhawa palibe ndi mayezedwawa.
- **CG-** no problem with the testing method

1. **Kodi mungamve bwanji ngati munthu wina wa mmudzi mwanu ataziwa zotsatira za magazi a mwana wanu atayezedwa kufufuza ngati ali ndi HIV kapena ayi?**

- **CG-** atha kukhumudwa chifukwa munthuyo sakuyenda kumva nawo zotsatilazo
- **CG-** I would be sad because these results are supposed to be confidential

1. **Kodi muli ndi maganizo kapena nkhawa zina zomwe mungafune kutidziwisa pa nkhani imeneyi**

- **CG-** Alibe nkhawa pokhuzana mmayedwe a ceipheid
- **CG-** I have no concerns
