# Supplementary material for: ‘If I am on ART, my new-born baby should be put on treatment immediately’: Exploring the acceptability, and appropriateness of Cepheid Xpert HIV-1 Qual assay for early infant diagnosis of HIV in Malawi
Source: PLOS Glob Public Health. 2023 Mar 10;3(3):e0001135. doi: 10.1371/journal.pgph.0001135 (PMC10021387; doi:10.1371/journal.pgph.0001135)
Supplement: S2 File — (ZIP) [file pgph.0001135.s005.zip › transcripts responses chichewa& english/DET035.docx]

**DET035_CG_F_30.7.18**

1. **Malingana ndi mmene tafotokozera za kayezedwe ka Cepheid, mwana ayenera kutengedwa magazi pachara kapena pa nsempha, inu monga kholo mungamve bwanji kuti mwana wanu ayezedwe magazi kuzera njira zimezi?**

- **CG-** INe ndingamve bwino chifukwa choti akayezedwa ndiziwa mmene alili.
- **CG-** I would feel good because after being tested I will know how my child is

1. **Kwainu monga kholo la mwana wa chichepere, maganizo anu ndi otani pokhuzana ndi mayezedwe a magazi kuti tidziwe kuti mwana ali ndi HIV kapena ayi malingana ndi mmene tafotokozera za kayezedwe ka Cepheid kuti zosatira zimatuluka kwa minitsi 92?**

- **CG-**  Ndili osangalala chifukwa choti njira zimenezi zakhazidwa ndipo ndili okondwela chifukwa chotiganizira ife paumoyo wa ana athu
- **CG-** I am happy because this method has been established and I am excited because it is benefiting our wellbeing.

1. **Kodi njira zimenezi tingazikhazikise bwanji mu zipatala? (tatiwuzani, tiyambe ndi gulu liti la anthu ndipo nchifukwa chani mukuganiza kuti tiyambe ndi gulu limeneli chifukwa chain?**

- **CG-** Mukuyenera inu madotolo kutifotokozera ndikufikira mwachangu ndipo musachedwe muyambire ana chifukwa anandiofunika chifukwa choti ndiatsogoleri ammawa
- **CG-** Doctors should explain and reach us as soon as possible and it should start with Children because they are the leaders of tomorrow.

1. **Kodi tingapange bwanji kuti kuyezesa magazi kwa ana ndi makolo awo kapena anthu owayang’ira zikhale za chinsinsi?**

- **CG-**  Otithandiza ndinu adotolo potisungira chinsinsi
- **CG-** You as Doctros are the ones to help us with this by keeping it private

1. **Kodi makolo angatengepo gawo lanji kuti njira zoyezesera magazi za Cepheid zikhazikisidwe mu chipatala chathu chino cha Mulanje?**

- **CG-** Tikuyenera kuwafikira anzathu zanjirazi kuti nawonso azisatire.
- **CG-** We need to reach out to our friends to explain to them about this method

b). **Kodi makolo awuzidwe zotani ndi uphungu wotani kuti amvesese za njira zoyezesera magazi za Cepheid?**

- **CG-** Ali kholo lanzeru akuyenera kuchilandira chifukwa ndizofunika kwa ife tomwe
- **CG-** A wise parent should welcome it because it is benefiting us

1. **Kodi azibambo angatengepo gawo lanji kuti njira zoyezesera magazi za Cepheid zikhazikisidwe mu chipatala chathu chino cha Mulanje? Tingawalimbikise bwanji azibambo kuti azitenga nawo gawo mukuyezedwa magazi mu njira za Cepheid?**

- **CG-** Azibambo nawoso akuyenera kuchimva magazi tikuyenera kuwalangiza kuti akuyenera kuzayezetsa ndikuziwa zathupi mwawo.
- **CG-** We need to encourage men to come and get tested too.

1. **Kodi anthu a mmudzi mwanu angamve bwanji njira zoyezesera magazi za Cepheid zitakhazikisidwa pa chipatala chanu chaching’ono mmudzi mwanu. Tingatani kuti anthu a mmudzi muno alimbikisidwe kutenga nawo mbali mu njira zoyezetsera magazi za Cepheid?**

- **CG-** Angamve bwino chifukwa choti njira ngati zimene kaleli tinalibe alangizi akuyenera kutengapo gawo powalangiza anthu pakayezedwe kamagazi.
- **CG-** They would like this and the health advisors should be the ones to come to us at the village to tell us about this method

1. **Kodi inu ndi anthu ena mma midzi mu mumakhala ndi nkhwa zanji zokhuzana ndi kulandila zosatira za magazi mwana akayezedwa kuti tiziwe kuti mwana ali ndi HIV kapena ayi?**

- **CG-** Umadaula komabe umayenera kuchilandira ndikuthandizira kuti mwana akahale ndi thanzi
- **CG-** I would be sad but I would need to accept it for the child to grow to healthy

1. **Kodi mungakhale ndi njira kapena maganizo a momwe tingathandizire kuchepesa nkhawa zokhuzana ndikulandila zotsatira za magazi mwana wayezedwa kuti tidziwe kuti mwana ali ndi HIV kapena ayi?**

- CG- Otithandiza ndinu achipatala kutithandizira chifukwa ife sitingakhale opanda nkhawa.
- CG- The ones who are supposed to help us are medical personnel because we cannot stay without worrying.

1. **Kuchokera pa nthawi yomwe mwana wanu wayezedwa magazi kuti tidziwe kuti mwana ali ndi HIV kapena ayi, mungapilile nthawi yayitali bwanji kuti mudziwe zosatira**

- **Same day**

**Patatha masiku**

**Miyezi iwiri kapena itatu**

**Fotokozani zifukwa zomwe mungasankhile yankho limeneli**

- **CG-**  Chifukwa adotolo atiuza kuti zotsatila tidikile 1 or 2hrs
- **CG-** its because the doctor told us to wait for the results for 1 to 2hrs

1. **Mwana wanu atayezedwa magazi, mungafune kudikila nthawi yayitali bwanji kuti mudziwe kuti mwana ali ndi HIV yomwe yimayambitsa matenda a AIDS?**

- **Same day**

**Patatha masiku**

**Miyezi iwiri kapena itatu**

**Fotokozani zifukwa zimene mwasankhila yankho limenelo**

- **CG-**

1. **Mwana wanu atayezedwa magazi mungafune kudikila nthaawi yayitali bwanji kuti muziwe kuti mwana alibe HIV yomwe imayambitsa matenda a AIDS**

- **Same day**

**Patatha masiku**

**Miyezi iwiri kapena itatu**

**Fotokozani zifukwa zomwe mungasankhile yankho limenelo**

- **CG-**

1. **kodi mungafune muwuzidwe zotani ndi uphungu otani kuti inu mupange chisankho choti mwana wanu ayezedwe magazi kuti mudziwe kuti mwana ali ndi HIV yomwe imayambitsa matenda a AIDS kapena ayi? Fotokozani bwino lomwe.**

- **CG-** Kutifikira kuchipatala kuno iyi ndinjira yapafupi chifukwa choti anthu amanyalanyaza
- **CG-** Reaching us here at the hospital

1. **Mungafune kuti tikufikileni mu njira yotani kuti tikuwuzeni zimezi ndikukupasani uphungu umenewu wa njira zoyezesera magazi za Cepheid?**

- **CG-** Mutifikire kuzera mmafumu izi ndizachidure
- **CG-** Use the village chief

1. **Kodi mungathe kuwalimbikisa makolo anzanu kapena owasamalira ana kuti alore ana Awo ayezedwwe magazi kuti aziwe ngati ali ndi HIV yoyambitsa matenda a AIDS kugwilitsa ntchito Cepheid?**

- **CG-**  Eya
- **CG-** yes

**15b) Nkhawa zanu zingakhale zotani ndi mayezedwe amenewa a Cepheid?**

- **CG-** Ine ndilibe nkhawa chifukwa choti ndimafuna ndiziwe mmene alili.
- **CG-** I have no objections because I want to know how my child is.

1. **Kodi mungamve bwanji ngati munthu wina wa mmudzi mwanu ataziwa zotsatira za magazi a mwana wanu atayezedwa kufufuza ngati ali ndi HIV kapena ayi?**

- **CG-** Ndingamve bwino chifukwa chinsinsi masiku ano kulibe aliyense akhonza kutenga nthendayi
- **CG-** I would be okay with it because HIV is common these days and anyone can have it.

1. **Kodi muli ndi maganizo kapena nkhawa zina zomwe mungafune kutidziwisa pa nkhani imeneyi**

- **CG-** Ine ndingokulimbikitsani kuti mupitilize chifukwa njirazi zilibwino ndithu
- **CG-** I would recommend that you should continue with this method because it sounds really good.
