# Supplementary material for: ‘If I am on ART, my new-born baby should be put on treatment immediately’: Exploring the acceptability, and appropriateness of Cepheid Xpert HIV-1 Qual assay for early infant diagnosis of HIV in Malawi
Source: PLOS Glob Public Health. 2023 Mar 10;3(3):e0001135. doi: 10.1371/journal.pgph.0001135 (PMC10021387; doi:10.1371/journal.pgph.0001135)
Supplement: S2 File — (ZIP) [file pgph.0001135.s005.zip › transcripts responses chichewa& english/DET037.docx]

**DET037_CG_F_30.7.18**

1. **Malingana ndi mmene tafotokozera za kayezedwe ka Cepheid, mwana ayenera kutengedwa magazi pachara kapena pa nsempha, inu monga kholo mungamve bwanji kuti mwana wanu ayezedwe magazi kuzera njira zimezi?**

- **CG-** Atha kumva bwino chifukwa akufuna kudziwa chowonadi mmene mwana alili nthupi.
- **CG-** I would feel good because I would know the status of my child.

1. **Kwainu monga kholo la mwana wa chichepere, maganizo anu ndi otani pokhuzana ndi mayezedwe a magazi kuti tidziwe kuti mwana ali ndi HIV kapena ayi malingana ndi mmene tafotokozera za kayezedwe ka Cepheid kuti zosatira zimatuluka kwa minitsi 92?**

- **CG-**  Maganizo awo ndiokondwa chifukwa njirazi kunalibe.
- **CG-** I am happy with it because this method wasn’t available before

1. **Kodi njira zimenezi tingazikhazikise bwanji mu zipatala? (tatiwuzani, tiyambe ndi gulu liti la anthu ndipo nchifukwa chani mukuganiza kuti tiyambe ndi gulu limeneli chifukwa chain?**

- **CG-**  Amene wabwera ku chipatala akuyenera kukapeleka uphungu kwa anzawo komanso akufunika kuyambira ndi ana, chifukwa makolo omwe amathawa kusikelo akakhala ndi mimba.
- **CG-** The one that has come to the hospital should be responsible to tell others and it needs to start with children.

1. **Kodi tingapange bwanji kuti kuyezesa magazi kwa ana ndi makolo awo kapena anthu owayang’ira zikhale za chinsinsi?**

- **CG-** Chinsinsi chikuyenera kukhala ndi mwini zotsatirayo.
- **CG-** the secret should be with the owner of the test results

1. **Kodi makolo angatengepo gawo lanji kuti njira zoyezesera magazi za Cepheid zikhazikisidwe mu chipatala chathu chino cha Mulanje?**

**CG-** Atengepo udindo kupita kpkayezetsa.

**CG-** take part by getting tested

b). **Kodi makolo awuzidwe zotani ndi uphungu wotani kuti amvesese za njira zoyezesera magazi za Cepheid?**

- **CG-** Awuzidwe za ndondomeko zimenezi.
- **CG-** they should be told about the whole procedures

1. **Kodi azibambo angatengepo gawo lanji kuti njira zoyezesera magazi za Cepheid zikhazikisidwe mu chipatala chathu chino cha Mulanje? Tingawalimbikise bwanji azibambo kuti azitenga nawo gawo mukuyezedwa magazi mu njira za Cepheid?**

- **CG-**  Azibambo azibwera kuchipatala ndi ma banja awokuzayezetsa , komanso tiowalimbikitse powanyengerera iweyo monga mayi wapakhomo.
- **CG-** Men should be coming to the hospital with families to get tested and we should motivate them as a woman

1. **Kodi anthu a mmudzi mwanu angamve bwanji njira zoyezesera magazi za Cepheid zitakhazikisidwa pa chipatala chanu chaching’ono mmudzi mwanu. Tingatani kuti anthu a mmudzi muno alimbikisidwe kutenga nawo mbali mu njira zoyezetsera magazi za Cepheid?**

- **CG-** Atha kumva bwin ochifukwa aziziwa mmene alili, koma tifalitse uthenga pochititsa msonkhano kuti aliyense azimvere yekha.
- **CG-** They can feel good about it and we need to reach out to everyone through convections

1. **Kodi inu ndi anthu ena mma midzi mu mumakhala ndi nkhwa zanji zokhuzana ndi kulandila zosatira za magazi mwana akayezedwa kuti tiziwe kuti mwana ali ndi HIV kapena ayi?**

- **CG-** Nkhawa imakhalapo yoti mwana afa kwa osachimvetsa, koma kwa ochimvetsa amatsatira njira ndikuwona mmene mwana angamusamalalire .
- **CG-** Fear of death arises to those who do not understand and accept it but those who understand and follow instructions find a way to care for the young one.

1. **Kodi mungakhale ndi njira kapena maganizo a momwe tingathandizire kuchepesa nkhawa zokhuzana ndikulandila zotsatira za magazi mwana wayezedwa kuti tidziwe kuti mwana ali ndi HIV kapena ayi?**

- **CG-** Pemphero ndilomwe lingathese nkhawa komanso kumwa mankhwala mwa ndondomeko.
- **CG-** Prayer is the only thing that can take away the stress and following the prescription when taking the medicine.

1. **Kuchokera pa nthawi yomwe mwana wanu wayezedwa magazi kuti tidziwe kuti mwana ali ndi HIV kapena ayi, mungapilile nthawi yayitali bwanji kuti mudziwe zosatira**

- **Tsiku lomwelo**

**Patatha masiku**

**Miyezi iwiri kapena itatu**

**Fotokozani zifukwa zomwe mungasankhile yankho limeneli**

- **CG-** Kuti akamapita kwawo aziwe mmene mwana alili, kuti ngati kuli kulandila chithandizo alandire pompo.
- **CG-** That when I am going home I should know the status and if there is need I should get the help I require.

1. **Mwana wanu atayezedwa magazi, mungafune kudikila nthawi yayitali bwanji kuti mudziwe kuti mwana ali ndi HIV yomwe yimayambitsa matenda a AIDS?**

- **TSiku lomwelo**

**Patatha masiku**

**Miyezi iwiri kapena itatu**

**Fotokozani zifukwa zimene mwasankhila yankho limenelo**

- **CG-**  Amafuna adziwe tsiku lomwero kuti akhale omasuka.
- **CG-** Same day results so that they can be free

1. **Mwana wanu atayezedwa magazi mungafune kudikila nthaawi yayitali bwanji kuti muziwe kuti mwana alibe HIV yomwe imayambitsa matenda a AIDS**

- **Tsiku lomwelo**

**Patatha masiku**

**Miyezi iwiri kapena itatu**

**Fotokozani zifukwa zomwe mungasankhile yankho limenelo**

- **CG-**  Chifukwa akhala osangalala ndinyengo yomwe mwana wawo ali.
- **CG-** Because they would be happy with the condition their child is in.

1. **kodi mungafune muwuzidwe zotani ndi uphungu otani kuti inu mupange chisankho choti mwana wanu ayezedwe magazi kuti mudziwe kuti mwana ali ndi HIV yomwe imayambitsa matenda a AIDS kapena ayi? Fotokozani bwino lomwe.**

- **CG-** Ma dokotala akuyenera kuwuza anthu akabwera ku chipatala.
- **CG-** Doctors should tell people at the hospitals

1. **Mungafune kuti tikufikileni mu njira yotani kuti tikuwuzeni zimezi ndikukupasani uphungu umenewu wa njira zoyezesera magazi za Cepheid?**

- **CG-** Tiwafikile mu zipatala zazing’ono kuti chifikire aliyense kuti achimvese ndikuzayezetsa.
- **CG-** This must reach local health Centre’s so that everyone can understand it.

1. **Kodi mungathe kuwalimbikisa makolo anzanu kapena owasamalira ana kuti alore ana Awo ayezedwwe magazi kuti aziwe ngati ali ndi HIV yoyambitsa matenda a AIDS kugwilitsa ntchito Cepheid?**

- **CG-**  Eya
- **CG-** yes

**15b) Nkhawa zanu zingakhale zotani ndi mayezedwe amenewa a Cepheid?**

- **CG-**  Alibe nkhawa iliyonse kamba ka njirazi.
- **CG-** I have no concerns

1. **Kodi mungamve bwanji ngati munthu wina wa mmudzi mwanu ataziwa zotsatira za magazi a mwana wanu atayezedwa kufufuza ngati ali ndi HIV kapena ayi?**

- **CG-** Sadabdaula chifukwa akhaka wamva za mmaluwa chowona sakhala akuchidziwa.
- **CG-** I would not have worries because they would just be rumors and not the truth.

1. **Kodi muli ndi maganizo kapena nkhawa zina zomwe mungafune kutidziwisa pa nkhani imeneyi**

- **CG-** Ganizo lawo ndiloti njilazi zifike mu zipatala zazing’ono kuti omwe sanamve amve kuti nawo athe kuyezetsa ana awo.
- **CG-** I think these new methods should also reach local health Centre’s and clinics so that those who couldn’t access the information can also get tested.
