# Supplementary material for: ‘If I am on ART, my new-born baby should be put on treatment immediately’: Exploring the acceptability, and appropriateness of Cepheid Xpert HIV-1 Qual assay for early infant diagnosis of HIV in Malawi
Source: PLOS Glob Public Health. 2023 Mar 10;3(3):e0001135. doi: 10.1371/journal.pgph.0001135 (PMC10021387; doi:10.1371/journal.pgph.0001135)
Supplement: Attachment — Submitted filename: Response to Reviewers_Rebuttal _letter_11.9.22.docx [file pgph.0001135.s006.docx]

**‘If I am on ART, my new-born baby should be put on treatment immediately’: Exploring the acceptability , and appropriateness of Cepheid Xpert HIV-1 Qual assay for early infant diagnosis of HIV in Malawi.**

**Manuscript submitted for review to PLOS Global Public Health**

**Author’s response to reviewer’s comments**

Dear Dr Lopez-Varela

We thank Reviewers 1 and 2 for their supportive and constructive comments to help strengthen this article. We detail our comments below and refer to the page numbers of the manuscript file with tracked changes to view these edits.

a. The major changes in this revised manuscript are as follows:

2. **Materials and Methods**

Removed table 1

3. **Results**
   a. Table 1 is now in this section

We include our responses to editorial and formatting requests on the following pages.

We wish to resubmit this research article titled **‘If I am on ART, my new-born baby should be put on treatment immediately’: Exploring the acceptability, and appropriateness of Cepheid Xpert HIV-1 Qual assay for early infant diagnosis of HIV in Malawi**. and thank you for your consideration of this manuscript.

MNN¥

Yours sincerely,

Maggie Nyirenda-Nyang’wa

**Editorial requests, formatting amendments and authors comments and revisions**

**Comment:**

Journal Requirements:

1. Please amend your detailed Financial Disclosure statement. This is published with the article. It must therefore be completed in full sentences and contain the exact wording you wish to be published.

a. Please clarify all sources of funding (financial or material support) for your study. List the grants (with grant number) or organizations (with url) that supported your study, including funding received from your institution.

b. State the initials, alongside each funding source, of each author to receive each grant.
c. State what role the funders took in the study. If the funders had no role in your study, please state: “The funders had no role in study design, data collection and analysis, decision to publish, or preparation of the manuscript.”

**Response:**

**MNN, McClay Foundation, 20 Seagoe Industrial Estate, Craigavon, Belfast, Northern Ireland. BT63 5QD via Queens University Belfast. Grant number R2167Cii**

**MNN, Fogarty International Center-funded Malawi HIV implementation Research Scientist Training program (D43 TW010060).**

**MNN, Adalma Foundation via Liverpool School of Tropical Medicine (no grant number or url available)**

**“All the funders had no role in study design, data collection and analysis, decision to publish, or preparation of the manuscript.”**

**Comment:**

2. Please ensure that the Title in your manuscript file and the Title provided in your online submission form are the same.

**Response: The title in the manuscript file and the title provided in the online submission form are now the same**

**‘If I am on ART, my new-born baby should be put on treatment immediately’: Exploring the acceptability, and appropriateness of Cepheid Xpert HIV-1 Qual assay for early infant diagnosis of HIV in Malawi.**

**Comment:**

3. We have noticed that you have uploaded Supporting Information files, but you have not included a list of legends. Please add a full list of legends for your Supporting Information files after the references list.

**Response: We have now added a list of legends for the Supporting Information files**

1. **Interview guide for caregivers**
2. **Interview guide for health workers**
3. **Acceptability Transcripts folder contains Caregivers transcripts both in Chichewa and English and health workers transcripts in Chichewa.**
4. **Chichewa Transcripts folder contains Caregivers transcripts in Chichewa**
5. **Coding book**

Please review your reference list to ensure that it is complete and correct. If you have cited papers that have been retracted, please include the rationale for doing so in the manuscript text, or remove these references and replace them with relevant current references. Any changes to the reference list should be mentioned in the rebuttal letter that accompanies your revised manuscript. If you need to cite a retracted article, indicate the article’s retracted status in the References list and also include a citation and full reference for the retraction notice.

**Thank you for the feedback that we should add new references , we removed references**

**a) Mary-Ann Davies, Jorge Pinto. Targeting 90–90–90 – don't leave children and adolescents behind. J Int AIDS Soc. 2015; 18(7Suppl 6): 20745 which was published in 2015 (Old version reference number 1),**

b) The 2015-16 Malawi Demographic and Health Survey (2015-16 MDHS).[ Available from <http://www.nsomalawi.mw/index.php?option=com_content&view=article&id=222&Itemid=108>. ( Accessed 3.3.2019) ( Old version reference number 19) and HIV Rates By Country 2021 (worldpopulationreview.com) (Accessed 13.3.2019) ( Old version reference number 22). **We have now added** **a new reference in the updated manuscript number 2** - UNAIDS Malawi fact sheets published in 2021 which has the latest data on Malawi.

**We also added references**

4- **Gulia et al and 5-Creek et al which discuss persistence of maternal antibodies and seroreversion time in HIV-1-uninfected children born to HIV-1-infected mothers.**

4.	Gulia J, Kumwenda N, Li Q, Taha TE. HIV seroreversion time in HIV-1-uninfected children born to HIV-1-infected mothers in Malawi. JAIDS Journal of Acquired Immune Deficiency Syndromes. 2007;46(3):332-7.

5.	Creek TL, Sherman GG, Nkengasong J, Lu L, Finkbeiner T, Fowler MG, et al. Infant human immunodeficiency virus diagnosis in resource-limited settings: issues, technologies, and country experiences. American journal of obstetrics and gynecology. 2007;197(3):S64-S71.

8. Cepheid Xpert® HIV-1 Qual assay Brochure [Available from: <https://cepheid.widen.net/content/qhuclnz714/pdf/Cepheid-Xpert-HIV-1-Qual-Brochure-CE-IVD-3053-English.pdf?u=bk12m>. **in addition to the WHO Prequalification of In Vitro Diagnostics PUBLIC REPORT as it is easy to understand**.

10- Nyirenda-Nyang'wa M, Manthalu G, Arnold M, Nkhoma D, Hosseinipour MC, Chagomerana M, et al. Costing and cost-effectiveness of Cepheid Xpert HIV-1 Qual Assay using whole blood protocol versus PCR by Abbott Systems in Malawi. Journal of Global Health Economics and Policy. 2022;2:e2022013. **This Costing and cost-effectiveness of Cepheid Xpert HIV-1 Qual Assay also highlights the performance and feasibilty aspects of this test but was only published in August 2022.**

Please note that **Chikaonda et al and Rosenberg et al** are the same published manuscript but when searched, currently it comes up as Rosenberg et al.

The rest of the references have not been changed.

**Reviewer 1**

**Comment:**

Abstract:

Word missing: “The qualitative [study/analysis/component] was nested within a larger diagnostic study…”

**Response: Thank you for pointing this out. We have addressed and added “study” (see page 2 line 47.**

**Comment:**

“Data was coded..” Should be data were coded

**Response: Thank you for pointing this out.** **We have corrected this (see page 2 line 50).**

**Comment:**

Background

Page 3: Authors mention internalised stigma but could also relate to other forms of stigma – anticipated stigma.

**Response: Thank you for this suggestion. We have now removed internalised (see page 3 line 98).**

**Comment:**

Line 101 – 104: The authors mention a list of patient/caregiver centred fears/concerns. Are there any studies/data related to health worker concerns? For instance resources, training, work load etc.

**Response: In this paper we discuss health worker concerns regarding resources (see page 16 lines 470-472 -504), training (see page 16 lines 473-474),**

**Workload (see page 16 lines 466),**

**We have added findings from other studies regarding health worker concerns towards XpertHIV e.g. resources, training, workload etc. (see page 19 lines 554-556) - Opollo et al states frequent power fluctuations required battery power back-up.**

**Comment:**

Line 142 – 147: Consider moving this paragraph and the table into the results/findings section.

**Response: Thank you for the suggestion. We have moved the paragraph and table into the results/findings section. (See pages 6-7 lines 188 to 203).**

**Comment:**

Table 1: Domestic work and housewife are grouped together. Should these not be reported as distinct categories? Is this income generating domestic work?

**Response: Thank you for the questions and feedback. We have now removed Domestic work and replaced with (unemployed) (See pages 7 lines 197).**

**Comment:**

Methods: How did discussion guides differ between health workers and caregivers?

**Response: Thank you for prompting us to be more explicit about the difference between discussion guides for health workers and caregivers.**

**The interview topic guides which lists the questions asked during the interviews to caregivers and health workers respectively are provided in the Supplementary Information.**

**The main difference was that the health workers guides focussed on the implementation of the intervention. The questions were around implementation -health workers interaction with the technology, how it works, its robustness, and how they perceive acceptability of the intervention both for the implementers and users. (See pages 5 lines 139-145).**

**Some questions included**

**how they felt as they delivered XpertHIV testing -are testing procedures easy to follow?**

**their interaction with care givers when taking blood, their general impression of parents and care givers as their children are having blood taken?**

**whether other health workers would be interested in XpertHIV testing, there will be any extra demand on the health services, time need to carry out XpertHIV test,**

**would ministry of health afford, be interested in adopting and scaling out XpertHIV? What would be the barriers and enablers?**

**Why women have a lot more confidence in hospital staff? Why caregivers especially women sometimes hardly explain answers, or their answers are very short.**

**Testing of HIV negative women whose partners /husbands are HIV infected partners.**

**For the users (parents /caregivers)**

**The focus was on how they perceive EID-HIV and their interaction with XpertHIV using whole blood as they receive the intervention.**

**Some of the questions included**

**Concerns that the community might have related to HIV testing and receipt of HIV test results of their child/ward.**

**Information they would want to be given to make an informed decision to accept that their ward should get an HIV test or not; How would they want to be approached and given the information.**

**Issues of confidentiality -how they would feel if the community learnt about HIV test results for their child/ward?**

**Perceptions about turn-around time to receiving test results.**

**How parents/caregivers felt when their ward underwent XpertHIV whole blood testing, their thoughts about the testing processes and testing environment, the role of parents and guardians in decision making for their wards to infants and children to undergo testing.**

**Their main concerns and worries towards XpertHIV whole blood testing- would they encourage other parents/guardians to allow their children to be tested for HIV using XpertHIV whole blood?**

**Suggestions or ideas for addressing possible community concerns about XpertHIV whole blood testing.**

**whether they would recommend XpertHIV whole blood testing implementation. If yes, who should be targeted and why?**

**How would their community feel about XpertHIV whole blood testing, what could be done to encourage community members to participate in XpertHIV whole blood testing**.

**Comment:**

Ethics:

A minor point: In the inclusion section, the authors note that participants had to be either over 18 years old or emancipated minors. In the ethics sections, the authors note that informed consent was obtained from the parent/guardian of each participant under 18 years of age. This seems to be contradictory – either participants are over 18 or emancipated, so no consent from guardians would be required.

**Response: Thank you for the suggestion. We have removed emancipated minors. (See page 5 line 133).**

**Comment:**

Results:

It would be interesting to note how the diagnostics process was described and explained to caregivers. Understanding of the test and knowledge of HIV and risk to infants could all impact the acceptability of the diagnostic test, as the authors briefly note in lines 271 – 274.

**Response: The diagnostic process was described and provided in the patient information sheet and consent forms. This information was verbally read out and discussed with the participants. XpertHIV testing procedures were discussed in detail.**

**At the end of the interview, participants were encouraged to ask questions and taking into consideration the level of literacy in our country, Malawi, we provided both written and verbal information so that the participants were able to read and also verbally understand procedures involved in diagnostics, expectations and risks of the XpertHIV test.**

**One of the sections was as follows**

**What will happen to my child or ward if they take part in XpertHIV testing?**

**A research nurse will collect some blood to be tested for HIV which will go through routine testing and a point of care test that has recently been developed called Cepheid HIV 1 Qual (XpertHIV) which is not currently used in Malawi but the instruments for this test are readily available in Malawi. There will be some discomfort from the prick from the needle as we get some blood from the vein or foot. The blood will then be processed by a laboratory technician and results will be given out on the same day but where results are equivocal, we will do more tests whose confirmatory results may take up to 3 days.**

**There is power in knowing one’s HIV status. If you or your child is found to be HIV negative, we cannot promise that the study will help them individually but the information we get will help us to improve this test which will help other children in future who will require HIV diagnosis.**

**If you or your child is found to be HIV positive, we will link them to HIV care services so that they can start antiretroviral therapy and will be followed up in clinic.**

**Comment:**

Line 316: “Genderred” should be ‘gendered’

**Response: We have deleted extra” r” from Genderred (see page 10 line 296.**

**Comment:**

Line 332: Were there any mention of men refusing to have children tested for HIV?

**Response: No, there was no mention of men refusing to have children tested for HIV**

**What was noted was that the infants and children came to seek health care at the facility with their mothers/ female caregivers. Only 3% of the caregivers were men which shows how caregiving aspect is gendered.**

**That is why there was mention that women need support from male partners.**

**Comment:**

Line 362: Write out TAT first time used

**Response: We have now written out turn-around time (TAT) (see Page 12 line 342).**

**Comment:**

Line 433: This is an interesting finding and has the potential to act as barrier to future uptake. Did any of the caregivers mention what they think happened to the blood (sinister use)?

**Response: No, whatever the caregivers felt was not mentioned, just fears of what happened to the blood taken.**

**In our discussion, we discussed fears of blood sucking, which are not confined to this group but broader context of the Malawian society.**

**Comment:**

Conclusion

The authors made note in the methods section how participants provided input on how to convey information on the diagnostic test. There is scope to include how the caregivers would like the information to be shared in future. What should be emphasised and how should caregivers be informed/prepared? The authors can consider expanding on this in the conclusion section.

**Response: The majority of the caregivers suggested that information regarding XpertHIV testing should be shared via the radio. Though the caregivers did not suggest or mention specific mechanism /methodologies of communicating or sharing information other than radio (very few people have radios or listen to them for a long time) and is not targeted, yet this information needs to be targeted at point of care.**

**We think information given to women requires future research to be conducted. The research should be context specific and can be used to inform women about technologies such as XpertHIV when they need to use them.**

**The caregivers emphasised the need for pre and post counselling to prepare them for XpertHIV testing, acceptance of test results and linkage to HIV care if their infants and children are diagnosed with HIV infection. This has been stated in the “Discussion” section.**

Reviewer #2:

**Comment:**

Line 43 – I suggest using EID-HIV FOR Early infant diagnosis of HIV

**Response: We agree with the comment made by Reviewer 2 and have changed EID to EID-HIV. (see page 2 line 43 ).**

**Comment:**

Line 196 – I suggest sharing the coding book for didactic purposes since the article is likely to be read by a variety of people including beginners in qualitative research analysis.

**Response: Thank you for pointing this out.**

**We truly appreciate the comments regarding the need to provide the coding book so that people including beginners in qualitative research analysis can understand the coding and theme analysis. We have now provided the coding book as supporting information however the interview topic guides which list the questions asked during the interviews are provided in the Supporting Information (Items 1 and 2).**

**The themes which emerged from the data analysis are those which are presented as headings and described in the Results section of the manuscript.**

**Comment:**

Line 219 – please remove the “now” in the sentence.

**Response: We have removed “now”. (See page 6 line 181).**

**Comment:**

The Ethics statement is incomplete regarding the mothers and caregivers as well as the health care providers please include.

**Response: We have now added health care providers to the ethics statement. (See page 6 line 184).**

**Comment:**

Line 465 – “nurse” is lowercase,

**Response: We have addressed this and put capital N (see page 16 line 477).**

**Comment:**

Line 474 – “nurse” is sentence case

Please revise and standardize the presentation of quotes and participants in brackets.

**Response: Thank you for pointing this out.**

**We have worked on the quotes of participants in brackets and removed spaces where they existed.**

**Comment:**

Line 585 – I do not see the sample of nurses as a limitation since it was very well justified why this number.

I would suggest the authors to include a recommendation section since there is a possibility of scale-up of the intervention. The recommendations would be useful to other sites nationally to consider and improve the implementation.

**Response: We agree with the comment made by Reviewer 2 that the sample of nurses is not a limitation since it was very well justified why this number. We removed this as a limitation (see page 19 line 568-572).**

**We have added recommendation section as suggested.**

**Recommendation (see page 20 lines 595 to 605)**

**This intervention is important for scale up however governments, policy makers and programme planners should have information about caregivers’ and healthcare providers’ experiences and engagement with XpertHIV using a whole-blood-sample and why it’s necessary to improve TAT as per findings of this qualitative study. There should also be enough heath workers in paediatric outpatient setting and wards where HIV testing of infants and children occurs.**

**Since this was a process evaluation of implementation research on XpertHIV using whole blood, we made recommendations in the main paper.**

**More implementation studies to assess engagement with XpertHIV in other sites nationally and in the region should be conducted whilst scaling-up this intervention.**
