# Supplementary material for: ‘If I am on ART, my new-born baby should be put on treatment immediately’: Exploring the acceptability, and appropriateness of Cepheid Xpert HIV-1 Qual assay for early infant diagnosis of HIV in Malawi
Source: PLOS Glob Public Health. 2023 Mar 10;3(3):e0001135. doi: 10.1371/journal.pgph.0001135 (PMC10021387; doi:10.1371/journal.pgph.0001135)
Supplement: Attachment — Submitted filename: Response to Reviewers_Rebuttal _letter_05.01.23.docx [file pgph.0001135.s007.docx]

**‘If I am on ART, my new-born baby should be put on treatment immediately’: Exploring the acceptability, and appropriateness of Cepheid Xpert HIV-1 Qual assay for early infant diagnosis of HIV in Malawi.**

**Manuscript submitted for review to PLOS Global Public Health**

**Author’s response to reviewer’s comments**

Dear Dr Gladys Ganal Paguio

Thank your for your supportive and constructive comments to help strengthen this article. We detail our comments below and refer to the page numbers of the manuscript file with tracked changes to view these edits.

We include our responses to editorial and formatting requests on the following page.

We wish to resubmit this research article titled **‘If I am on ART, my new-born baby should be put on treatment immediately’: Exploring the acceptability, and appropriateness of Cepheid Xpert HIV-1 Qual assay for early infant diagnosis of HIV in Malawi**. and thank you for your consideration of this manuscript.

Yours sincerely,

MNN¥

Maggie Nyirenda-Nyang’wa

**Editorial requests, formatting amendments and authors comments and revisions**

Journal Requirements:

**Comment:**

1. Please amend your detailed Financial Disclosure statement. This is published with the article. It must therefore be completed in full sentences and contain the exact wording you wish to be published.

a. State the initials, alongside each funding source, of each author to receive each grant.

b. If any authors received a salary from any of your funders, please state which authors and which funders.

If you did not receive any funding for this study, please simply state: “The authors received no specific funding for this work.”

**Response:**

Thank you, this has now been addressed on clean copy page 21 lines 637 to 645 and the manuscript with tracked changes page 21 lines 633 to 641.

It states

Maggie Nyirenda - Nyang'wa (MNN) received funding from the following :

i) McClay Foundation, 20 Seagoe Industrial Estate, Craigavon, Belfast, Northern Ireland. BT63 5QD via Queens University Belfast - Grant number R2167Cii

ii) Fogarty International Center-funded Malawi HIV implementation Research Scientist Training program- Grant number (D43 TW010060).

ii) Adalma Foundation via Liverpool School of Tropical Medicine (no grant number or url available)

“All the funders had no role in study design, data collection and analysis, decision to publish, or preparation of the manuscript.”

**Comment:**

2. We do not publish any copyright or trademark symbols that usually accompany proprietary names, eg ©, ®, or ™ (e.g. next to drug or reagent names). Please remove all instances of trademark/copyright symbols throughout the text, including ® on pages 22 and 23.

**Response:**

Thank you for this information. From the clean copy, we have now removed

®from reference 8 on pages 22 line 696

®from reference 12 on pages 23 line 711

®from reference 14 on pages 23 line 717

In response to reviewers

**Reviewer** #**1**

The authors have addressed all of the comments. A few (very minor) suggested edits:

**Comment:**

Throughout the text, please refer to mothers or children “living with HIV rather than “HIV-positive”.

**Response:**

Thank you for this suggestion. We have now removed “HIV-positive” and replaced with “living with HIV (See the following pages and lines in the clean copy)

page 9 line 243

page 9 line 253

**Comment:**

Line 232: "Written informed consent was obtained from the parent/guardian of each participant under 18 years of age and health care providers. " Does participants refer to the children or the caregivers? Or does it refer to caregivers/mothers who are under the age of 18? If so, did they complete assent as well?

**Response:**

Thank you for prompting us to be more explicit about the participants and consent for this study. This qualitative study investigated healthcare providers and caregivers’ perspectives on the acceptability and appropriateness of Cepheid Xpert HIV-1 Qual assay for early infant diagnosis of HIV. The caregivers/mothers and healthcare providers median age and ranges were 25 years (IQR: 22 -34) and 33 yrs (IQR: 23 -58) respectively. They were all above18 years therefore they all completed consent forms. We have clarified this now on page 6 line 183-184.

**Comment:**

Line 350 typo: "approval from their male partner’s" - should be "male partners"

**Response:**

We have deleted the apostrophe from “partner’s". (See page 11 line 304 in the clean copy.).

Reviewer #2:

**Comment:**

I delighted to see that the comments and suggestions from my first review were agreed by the authors and included.
I have no further comments.

**Response:**

Thank you
